# Identification of active compounds in Ophiopogonis Radix from different geographical origins by UPLC-Q/TOF-MS combined with GC-MS approaches

**DOI:** 10.1515/biol-2022-0096

**Published:** 2022-08-12

**Authors:** Xiaoyu Zha, Gaowen Li, Ling Zhang, Qun Chen, Qing Xia

**Affiliations:** Department of Pharmacology, Ningbo College of Health Science, Rd. Xuefu 51#, Yinzhou District, 315100 Ningbo, Zhejiang, China

**Keywords:** Ophiopogonis Radix, Maidong, UPLC-Q/TOF-MS, GC-MS, LC-MS

## Abstract

Ophiopogonis Radix, also known as Maidong in Chinese, is largely produced in the Sichuan and Zhejiang provinces: “Chuan-maidong (CMD)” and “Zhe-maidong (ZMD),” respectively. This study aimed to distinguish and evaluate the quality of CMD and ZMD. In this study, the tubers of CMD and ZMD were investigated using UPLC-Q/TOF-MS, GC-MS, and LC-MS methods, respectively. Overall, steroidal saponins, homoisoflavonoids, amino acids, and nucleosides were quickly identified. Furthermore, multivariate statistical analysis revealed that CMD and ZMD could be separated. Moreover, CMD showed higher levels of 4-aminobutanoic acid, glycine, l-proline, monoethanolamine, and serine than ZMD. Besides, the levels of chlorogenic acid, traumatic acid, cytidine, cadaverine, pyridoxine 5-phosphate, glutinone, and pelargonidin 3-*O*-(6-*O*-malonyl-β-d-glucoside) were remarkably higher in ZMD than in CMD. Furthermore, these different constituents were mainly associated with galactose metabolism; starch and sucrose metabolism; cysteine and methionine metabolism; valine, leucine, and isoleucine biosynthesis; and glycerophospholipid metabolism. In general, these results showed many differences between the bioactive chemical constituents of *Ophiopogon japonicus* from different production areas, where ZMD performed better in the quality assessment than CMD, and that UPLC-Q/TOF-MS, GC-MS, and LC-MS are effective methods to discriminate medicinal herbs from different production areas.

## Graphical abstract

**Figure:**
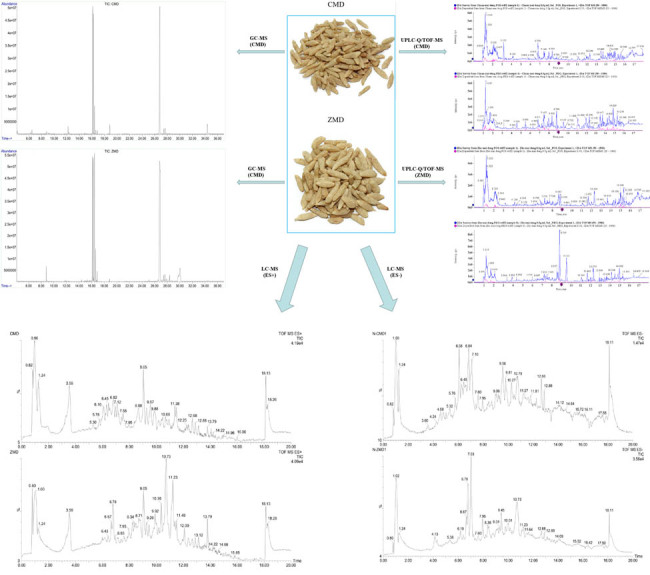

## 1 Introduction

Ophiopogonis Radix (known as Maidong), the root tuber of *Ophiopogon japonicus* Ker-Gawl, belongs to the family Liliaceae and is the most widely used traditional Chinese medicine (TCM) in the Chinese Pharmacopoeia [1]. According to TCM theory, Maidong nourishes yin, moistens the lungs, promotes body fluid production, eases the mind, and clears away heart fires [2,3]. It has been employed to control diabetes and its complications [4], radiation pneumonitis [5], atherosclerotic coronary heart disease, and viral myocarditis [6]. Additionally, modern phytochemical studies have suggested that Maidong is rich in various biologically active compounds, including steroidal saponins, amino acids, homoisoflavonoids, polysaccharides, and nucleosides, which have beneficial immunomodulatory, anti-inflammatory, central nervous system protective, antioxidative, and anti-apoptosis effects [7–9]. Although several studies on the chemical components of Maidong have been reported, these studies were performed with a single analytical technique and are not comprehensive [10,11]. Therefore, to the best of our knowledge, there is still a lack of information on the comprehensive chemical constituents of Maidong determined by using a multidimensional assessment approach.

At present, the cultivation regions of Ophiopogonis Radix are mainly concentrated in the Sichuan (mainly Santai County) and Zhejiang provinces (mainly the city of Cixi) of China. Ophiopogonis Radix from Sichuan and Zhejiang provinces is popularly called Chuan-maidong (CMD) and Zhe-maidong (ZMD), respectively, but ZMD is generally considered superior to Ophiopogonis Radix cultivated in other provinces [10]. Currently, it is generally accepted that the quantity and pharmacological effects of tubers on Maidong in different areas are controlled by environmental conditions and endogenous factors [11]. A study by Lu et al. found that the chemical constituents of CMD and ZMD differed much from each other according to high-performance liquid chromatography-mass spectrometry (LC-MS) with multivariate statistical analysis [12]. With the structural transformation of economic development, the cultivation of ZMD has drastically decreased in Zhejiang Province in recent years. Sichuan has now become the primary place of MD production [10,12]. In addition, CMD and ZMD are difficult to distinguish based on their appearance, which has also made quality control of Maidong challenging. Considering these findings, further study of the compositional distinction between Maidong tubers grown in Sichuan and Zhejiang provinces remains limited. Differences between the chemical constituents of CMD and ZMD have been reported by using LC coupled with evaporative light scattering detection, gas chromatography (GC) coupled with MS, or LC coupled with MS [2,10,13]. Because of the complexity of chemical constituents, those studies only assessed saponins, polysaccharides, or homoisoflavonoids.

Thus, this study aimed to comprehensively characterize the bioactive constituents of CMD and ZMD from the two producing areas and investigate their metabolic pathway. In this study, CMD and ZMD tubers were collected, and the chemical information of multiple bioactive constituents was characterized by using ultra-performance liquid chromatography-quadrupole-time-of-flight mass spectrometry (UPLC-Q/TOF-MS), as well as GC-MS and LC-MS methods with multivariate statistical analysis, including principal component analysis (PCA) and orthogonal partial least squares-discriminate analysis (OPLS-DA). Furthermore, the pathways of significantly different chemical constituents were identified to reveal the potential biological events occurring between CMD and ZMD. Therefore, the results of this study might provide a guide for a comprehensive evaluation and quality control, as well as a study on the mechanism of Ophiopogonis Radix.

## 2 Materials and methods

### 2.1 Chemicals and reagents

Ultrapure water was prepared by a Milli-Q system (Milford, MA, USA). Acetonitrile and methanol (HPLC grade) were produced by Merck (Darmstadt, Germany). Bis(trimethylsilyl)trifluoroacetamide was obtained from CNW Technologies (Shanghai, China). dl-*o*-Chlorophenylalanine was purchased from GL Biochem (Shanghai) Ltd (Shanghai, China). All the other chemicals and solvents were of analytical grade (purity (S98%) for GC/LC use.

### 2.2 Plant materials

CMD and ZMD at the same growth stage were collected from the market as mature plants in the cities of Cixi (Zhejiang, China) and Mianyang (Sichuan, China) in May 2020, respectively, including six batches of ZMD and six batches of CMD samples. All the samples were authenticated by Professor Qing Xia, Ningbo College of Health & Science, Ningbo, Zhejiang, China.

### 2.3 Sample preparation for UPLC-Q/TOF-MS analysis

The aim of the present study was to thoroughly evaluate the polysaccharides and saponins of CMD and ZMD in water extract solutions. Briefly, the dried tubers of CMD and ZMD were ground and passed through a standard 60-mesh filter. The obtained powder (3.0 g) was accurately weighed into a conical flask, immersed in 200 mL of distilled water for 30 min, and boiled for 90 min. Then, the liquid extract obtained was concentrated to 10 g by using rotating evaporation (JC-ZF-1L, Qingdao Juchuang Times Environmental Protection Technology Co., Ltd, China). The obtained liquid extract was dissolved in methanol at a weight ratio of 1:1 and centrifuged at a speed of 14,000 rpm for 20 min before UPLC-Q/TOF-MS analysis.

### 2.4 Sample preparation for GC-MS analysis

Additionally, approximately 50 mg of the dried tubers of CMD and ZMD were used for the extraction procedure. Briefly, CMD and ZMD were mixed with 800 μL of methanol containing an internal standard (2.8 mg/mL dl-*o*-Chlorophenylalanine). Then, all samples were ground to a fine powder using a grinding mill operated at 65 Hz for 120 s. The samples were ultrasonicated at 4 kHz in an ice bath for 30 min and then centrifuged at 12,000 rpm at 4°C for 10 min. Subsequently, 200 μL of the supernatant was evaporated to dryness at room temperature. After that, the samples were derivatized by shaking with 30 μL of methoxyamine hydrochloride (20 mg/mL) in pyridine for 90 min at 37°C. The samples were then trimethylsilylated by adding 30 μL of bis(trimethylsilyl)trifluoroacetamide and incubated for 1 h at 70°C. After the reaction was complete, the samples were incubated for 1 h at room temperature. Finally, 200 μL of the supernatant was transferred to a vial for GC-MS analysis. The mix of all extract solutions was used as a control sample (QC).

### 2.5 Sample preparation for LC-MS analysis

Furthermore, approximately 50 mg of the dried tubers of CMD and ZMD were applied for the extraction procedure. Briefly, CMD and ZMD were extracted with 800 μL of methanol containing dl-*o*-Chlorophenylalanine (2.8 mg/mL) to investigate flavonoids. All samples were ground to a fine powder using a grinding mill operated at 65 Hz for 120 s. The samples were ultrasonicated at 40 kHz in an ice bath for 30 min and then centrifuged at 12,000 rpm at 4°C for 15 min. After that, 200 μL of the supernatant was transferred to a vial for LC-MS analysis. The mix of all extract solutions was used as QC.

### 2.6 UPLC-Q/TOF-MS analysis and MS conditions

UPLC-Q/TOF-MS analysis was performed on a Waters ACQUITY UPLC I-Class PLUS system (Waters Corporation, Milford, MA, USA) coupled with hybrid quadrupole time-of-flight tandem mass spectrometer (SCIEX X-500R, SCIEX, Framingham, MA, USA) equipped with TurboIonSpray sources and a Turbo ion spray interface. Briefly, chromatographic separation was performed on a Waters UPLC BEH C_18_ column (100 mm × 2.1 mm, 1.7 µm particle size) at 40°C with a flow rate of 0.3 mL/min, and the injection volume was 3 μL. The mobile phase was composed of 0.1% ammonium formate in acetonitrile (A) and 0.1% formic acid aqueous solution (B) and introduced under the following gradient conditions: 0–12 min, 99% A–50% A; 12–14.5 min, 50–15% A; 14.5–15 min, 15–1% A; 15–18 min, 1% A; 18–18.1 min, 1% B–99% A; and 18.1–21 min, 99% A. TOF MS was performed using a Turbo Ion Spray ion source and ESI positive (+) and negative (−) ion scanning modes. The MS analysis conditions were as follows: source temperature: 600°C; nebulizing gas (N_2_): 55 psi; drying gas (N_2_): 45 psi; curtain gas (CUR): 35 psi; IonSpray Voltage Floating: 5,500 V/−4,500 V; TOF MS scan *m*/*z* range: 100–1,500 Da; TOF-MS/MS scan *m*/*z* range: 25–1,500 Da; TOF MS scan accumulation time: 0.25 s/spectra; and product ion scan accumulation time: 0.035 s/spectra. MS uses information-dependent acquisition and high sensitivity mode.

### 2.7 GC-MS analysis

An Agilent 6890A/5973C GC-MS system and a DB-5MS fused-silica capillary column (30 m × 0.25 mm × 0.25 μm, Agilent J&W Scientific, USA) were used for analysis. The injector temperature was 280°C. The temperature program used was as follows: the column temperature was held at 70°C for 2 min, increased by 10°C  to 200°C, increased by 5°C  to 280°C and held there for 6 min. The ion source and quadrupole rod temperatures were 230 and 150°C, respectively. The column effluent was fully scanned in the mass range of 50–550 *m*/*z*. The data were subjected to feature extraction and preprocessed with the XCMS package in R software (version 4.0.5, https://www.r-project.org/) and then normalized and edited into a two-dimensional data matrix by Excel 2010 software; data included the retention time (RT), the mass-to-charge ratio, observations (samples), and peak intensity.

### 2.8 LC-MS analysis

LC-MS was performed using an ACQUITYTM UPLC-QTOF platform (Waters, Wexford, Ireland) with a Waters ACQUITY UPLC HSS T3 column (2.1 mm × 100 mm, 1.8 μm). The mobile phases consisted of 0.1% aqueous formic acid (v/v) (A) and acetonitrile (B), and were introduced under the following gradient elution conditions: 0% B at 0–1 min, 0–20% B at 1–2 min, 20–50% B at 2–12 min, 50–95% B at 12–15 min, and 95–100% B at 15–20 min. The flow rate was set at 0.35 mL/min, and the column temperature was maintained at 40°C. The injection volume was 6 μL. The electrospray ionization source was set in both ESI (+) and ESI (–) ionization modes. The parameters were set as follows: source and desolvation temperatures: 120 and 350°C, respectively; desolvation gas (N_2_) flow: 600 L/h; capillary voltages: 1.4 kV for ESI (+) and 1.3 kV for ESI (–); sampling cone: 40 V for ESI (+) and 23 V for ESI (–); cone gas (N_2_) flow: 50 L/h; collision energy: 10–40 V; ion energy: 1 V; scan time: 0.03 s; and interscan time: 0.02 s. The mass range scanned was 50–1,500 *m*/*z*. MS data were collected with MassLynx 4.1 software.

### 2.9 Data analysis

For UPLC-Q/TOF-MS analysis, the data were processed using SCIEX OS software with multiple confidence criteria, including quality accuracy, RT, isotopes, and matching use of compound libraries. In this study, the TCM MS/MS Library, which contains secondary data for more than 1,500 Chinese herbal medicines, was used to identify the target constituents based on the first-order accurate mass number, isotope distribution ratio, and MS/MS of the compounds. For GC-MS analysis, a total of 1,060 features were collected in this experiment, and the data were imported into SIMCA-P (version 13.0, Umetrics AB, Sweden) software for PCA and OPLS-DA. For LC-MS analysis, the data were first transformed to CDF files by CDFbridge and input into the XCMS package in R software and then normalized and edited into a two-dimensional data matrix by Excel 2007 software. A total of 1,712 features in ESI (+) ionization mode and 1,138 features in ESI (–) ionization mode were collected in this experiment, and the data were imported into SIMCA-P software to perform PCA and OPLS-DA.

## 3 Results

### 3.1 UPLC/Q-TOF MS analysis of chemical constituents of CMD and ZMD

**Figure 1:**
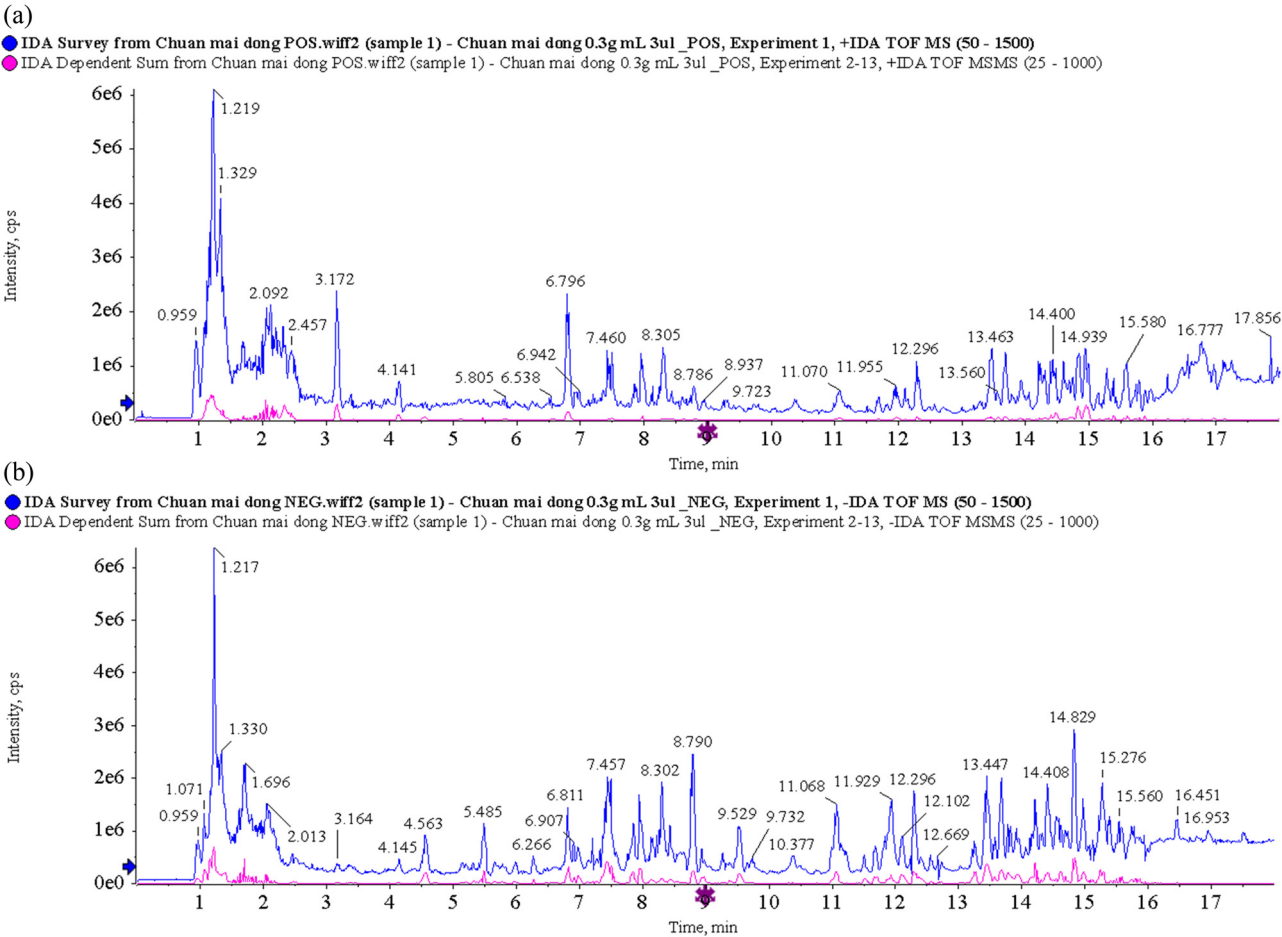
Total ion chromatogram of CMD obtained by UPLC/Q-TOF MS analysis in (a) positive ion mode and (b) negative ion mode.

**Figure 2:**
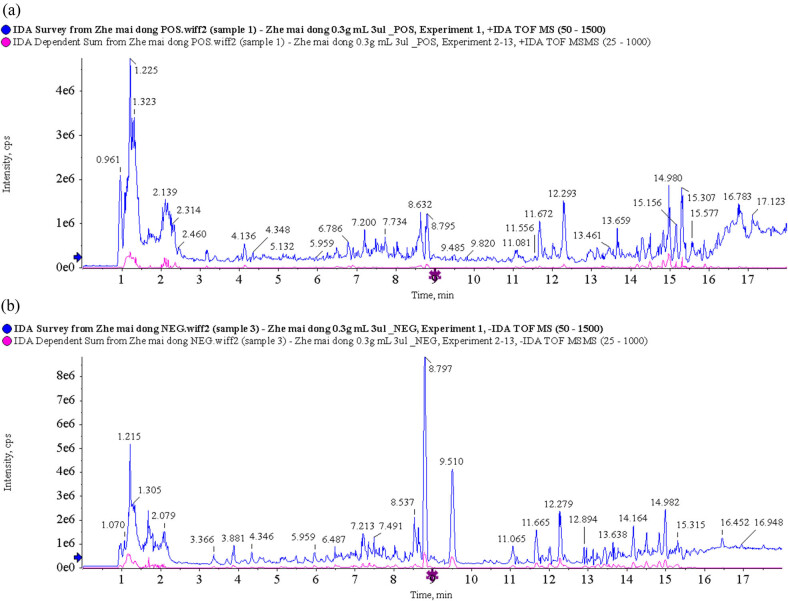
Total ion chromatogram of ZMD obtained by UPLC/Q-TOF MS analysis in (a) positive ion mode and (b) negative ion mode.

By using UPLC/Q-TOF MS analysis, CMD and ZMD could be analyzed within 21 min and exhibited some major peaks in the total ion chromatography, as shown in Figures 1 and 2, respectively. According to the TCM MS/MS Library in SCIEX OS software, the chemical constituents were identified qualitatively. As a result, a total of 26 chemical constituents of CMD were identified in positive ion mode and 33 chemical constituents of CMD were identified in negative ion mode in UPLC/Q-TOF MS analysis. Furthermore, a total of 33 chemical constituents of ZMD were identified in positive ion mode and 39 chemical constituents of ZMD were identified in negative ion mode in UPLC/Q-TOF MS analysis. Most of these chemical constituents were steroidal saponins, amino acids, homoisoflavonoids, polysaccharides, and nucleosides. Additionally, our UPLC/Q-TOF MS analysis revealed that the dried tubers of both CMD and ZMD contained methylophiopogonanone A, methylophiopogonanone B, methylophiopogonone A, ophiopogonin D, ophiopogonin D′, ophiopogonanone C, ophiopogonanone E, and ruscogenin. Detailed information on the identified chemical constituents is listed in Tables 1–4. Also, the MS fragmentation pathways for different chemical constituents of CMD and ZMD in positive ion mode or negative ion mode are shown in Tables S1 and S2, respectively.

**Table 1:** Putative identification of CMD in positive ion mode

No.	Component name	Area	RT	Formula	Precursor mass	Found at mass	Mass error (ppm)
1	l(+)-Arginine	7,402,000	1.14	C_6_H_14_N_4_O_2_	175.119	175.1187	−1.5
2	Trigonelline	253,900	1.21	C_7_H_7_NO_2_	138.055	138.0551	1
3	Proline	433,800	1.24	C_5_H_9_NO_2_	116.071	116.0707	0.4
4	Glutamic acid	187,400	1.3	C_5_H_9_NO_4_	148.06	148.0606	0.8
5	Betaine	140,700	1.37	C_5_H_11_NO_2_	118.086	118.0863	0.6
6	Nicotinic acid	155,300	1.71	C_6_H_5_NO_2_	124.039	124.0394	0.9
7	Nicotinamide	235,400	1.79	C_6_H_6_N_2_O	123.055	123.0554	0.9
8	Adenosine	2,131,000	2.36	C_10_H_13_N_5_O_4_	268.104	268.1038	−0.9
9	Cordycepin	43,090	2.42	C_10_H_13_N_5_O_3_	252.109	252.1093	0.8
10	Guanosine	207,900	2.46	C_10_H_13_N_5_O_5_	284.099	284.0992	0.7
11	Phenylalanine	1,719,000	3.17	C_9_H_11_NO_2_	166.086	166.0863	0.3
12	Cinnamic acid	48,090	3.18	C_9_H_8_O_2_	149.06	149.0598	0.6
13	4-Hydroxybenzoic acid	12,960	4.73	C_7_H_6_O_3_	139.039	139.039	0.4
14	Esculetin	23,820	5.13	C_9_H_6_O_4_	179.034	179.034	0.6
15	Hyperin	4,621	6.5	C_21_H_20_O_12_	465.103	465.1034	1.4
16	Syringaldehyde	3,442	6.56	C_9_H_10_O_4_	183.065	183.065	−1.1
17	Luteoloside	2,638	6.67	C_21_H_20_O_11_	449.108	449.1091	2.8
18	Isoferulic acid	3,834	6.74	C_10_H_10_O_4_	195.065	195.0652	0.1
19	Narirutin	2,831	7.08	C_27_H_32_O_14_	581.186	581.1873	1.4
20	Neohesperidin	4,766	7.5	C_28_H_34_O_15_	611.197	611.1977	1.1
21	Tiliroside	4,904	8.91	C_30_H_26_O_13_	595.145	595.145	0.7
22	Calycosin-7-*O*-glucoside	6,176	9.42	C_22_H_22_O_10_	447.129	447.1284	−0.4
23	Nobiletin	31,320	12.77	C_21_H_22_O_8_	403.139	403.1388	0.1
24	Ophiopogonin D′	877,800	14.83	C_44_H_70_O_16_	855.474	855.4731	−0.6
25	Ruscogenin	178,800	14.85	C_27_H_42_O_4_	431.316	431.3154	−0.5
26	Ophiopogonanone C	3,902	15.06	C_20_H_20_O_6_	357.133	357.134	1.9

**Table 2:** Putative identification of CMD in negative ion mode

No.	Component name	Area	RT	Formula	Precursor mass	Found at mass	Mass error (ppm)
1	Histidine	17,540	1.09	C_6_H_9_N_3_O_2_	154.062	154.0621	−0.7
2	l(+)-Arginine	165,100	1.1	C_6_H_14_N_4_O_2_	173.104	173.1044	0.2
3	Glutamic acid	57,600	1.14	C_5_H_9_NO_4_	146.046	146.046	0.6
4	d-(+)-Mannose	786,300	1.22	C_6_H_12_O_6_	179.056	179.0562	0.4
5	l-Malic acid	3,632,000	1.38	C_4_H_6_O_5_	133.014	133.0144	0.9
6	Citric acid	9,523,000	1.9	C_6_H_8_O_7_	191.02	191.0198	0.3
7	Succinic acid	58,120	2.29	C_4_H_6_O_4_	117.019	117.0193	0
8	Leucine	473,700	2.47	C_6_H_13_NO_2_	130.087	130.0873	0
9	Guanosine	335,800	2.47	C_10_H_13_N_5_O_5_	282.084	282.0844	0.1
10	Gallic acid	10,950	2.65	C_7_H_6_O_5_	169.014	169.0142	−0.3
11	Phenylalanine	592,600	3.18	C_9_H_11_NO_2_	164.072	164.0717	−0.1
12	Vanillic acid	119,600	3.37	C_8_H_8_O_4_	167.035	167.035	0.2
13	l-Tryptophan	383,500	4.16	C_11_H_12_N_2_O_2_	203.083	203.0827	0.7
14	4-Hydroxybenzoic acid	36,400	4.63	C_7_H_6_O_3_	137.024	137.0245	0.9
15	4-*O*-caffeoyl quinic acid	21,560	4.8	C_16_H_18_O_9_	353.088	353.0881	0.8
16	Esculetin	151,700	5.13	C_9_H_6_O_4_	177.019	177.0195	0.8
17	Caffeic acid	154,600	5.21	C_9_H_8_O_4_	179.035	179.0351	0.6
18	Shikimic acid	548,900	5.49	C_7_H_10_O_5_	173.046	173.0454	−0.9
19	Ellagic acid	28,660	6.35	C_14_H_6_O_8_	300.999	300.999	0.1
20	Hyperin	34,590	6.51	C_21_H_20_O_12_	463.088	463.0875	−1.4
21	Narirutin	8,615	7.08	C_27_H_32_O_14_	579.172	579.1718	−0.3
22	Neohesperidin	16,980	7.5	C_28_H_34_O_15_	609.182	609.1827	0.4
23	Tiliroside	16,580	8.92	C_30_H_26_O_13_	593.13	593.1292	−1.4
24	Calycosin-7-*o*-glucoside	4,825	9.43	C_22_H_22_O_10_	491.119	491.1188	−1.4
25	Gracillin	14,970	10.78	C_45_H_72_O_17_	929.475	929.473	−2.3
26	Liriope muscari baily saponins C	165,000	13.46	C_44_H_70_O_17_	869.454	869.4514	−3
27	Methylophiopogonone A	386,000	14.71	C_19_H_16_O_6_	339.087	339.0864	−2.9
28	Ophiopogonin D	3,242,000	14.83	C_44_H_70_O_16_	899.465	899.4623	−2.6
29	Methylophiopogonanone B	2,266,000	14.99	C_19_H_20_O_5_	327.124	327.1228	−3
30	Liriopesides B	181,500	15.12	C_39_H_62_O_12_	767.422	767.4199	−3.1
31	Gingerglycolipid B	5,459	15.2	C_33_H_58_O_14_	723.381	723.3788	−2.8
32	Corosolic acid	3,440	15.55	C_30_H_48_O_4_	471.348	471.3459	−4.4
33	Ophiopogonanone C	75,350	15.72	C_19_H_16_O_7_	355.082	355.0814	−2.7

**Table 3:** Putative identification of ZMD in positive ion mode

No.	Component name	Area	RT	Formula	Precursor mass	Found at mass	Mass error (ppm)
1	l(+)-Arginine	4,301,000	1.11	C_6_H_14_N_4_O_2_	175.119	175.1189	−0.5
2	Trigonelline	207,100	1.2	C_7_H_7_NO_2_	138.055	138.0551	1.2
3	Proline	2,757,000	1.22	C_5_H_9_NO_2_	116.071	116.0706	0
4	Nicotinic acid	56,410	1.72	C_6_H_5_NO_2_	124.039	124.0395	1.6
5	Nicotinamide	208,200	1.8	C_6_H_6_N_2_O	123.055	123.0553	0.4
6	Adenosine	926,700	2.35	C_10_H_13_N_5_O_4_	268.104	268.104	−0.2
7	Cordycepin	41,280	2.42	C_10_H_13_N_5_O_3_	252.109	252.1096	1.9
8	Isoleucine	280,500	2.46	C_6_H_13_NO_2_	132.102	132.1019	−0.2
9	Guanosine	90,750	2.47	C_10_H_13_N_5_O_5_	284.099	284.0995	1.9
10	Phenylalanine	285,000	3.17	C_9_H_11_NO_2_	166.086	166.0863	0.4
11	Chlorogenic acid	57,310	4.64	C_16_H_18_O_9_	355.102	355.1031	2
12	4-Hydroxybenzoic acid	14,360	4.73	C_7_H_6_O_3_	139.039	139.0391	0.9
13	Daphnetin	124,300	5.13	C_9_H_6_O_4_	179.034	179.0339	−0.1
14	Esculetin	124,300	5.13	C_9_H_6_O_4_	179.034	179.0339	−0.1
15	Rutin	71,010	6.3	C_27_H_30_O_16_	611.161	611.1621	2.3
16	Hyperin	297,200	6.5	C_21_H_20_O_12_	465.103	465.1034	1.4
17	Isoscopoletin	42,290	6.69	C_10_H_8_O_4_	193.05	193.0498	1.6
18	Isoferulic acid	2,997	6.74	C_10_H_10_O_4_	195.065	195.0657	2.4
19	Luteoloside	1,829	6.96	C_21_H_20_O_11_	449.108	449.1097	4.1
20	Narirutin	15,920	7.08	C_27_H_32_O_14_	581.186	581.1875	1.8
21	Luteolin	107,100	7.14	C_15_H_10_O_6_	287.055	287.0553	1
22	Genistein	31,520	7.38	C_21_H_20_O_10_	433.113	433.1134	1
23	Hesperidin	55,530	7.48	C_28_H_34_O_15_	611.197	611.1975	0.7
24	Pratensein-7-*O*-glucoside	4,385	7.7	C_22_H_22_O_11_	463.123	463.1243	1.8
25	Tiliroside	190,600	8.91	C_30_H_26_O_13_	595.145	595.1444	−0.4
26	Calycosin-7-*O*-glucoside	42,280	9.42	C_22_H_22_O_10_	447.129	447.1293	1.5
27	Farrerol	249,800	10.35	C_17_H_16_O_5_	301.107	301.1068	−0.8
28	Patchouli alcohol	611,500	12.24	C_15_H_24_	205.195	205.1949	−0.7
29	Nobiletin	99,200	12.77	C_21_H_22_O_8_	403.139	403.1389	0.3
30	Diosgenin	38,270	14.75	C_27_H_42_O_3_	415.321	415.3208	0.2
31	Ruscogenin	6,144	14.83	C_27_H_42_O_4_	431.316	431.3175	4.4
32	Ophiopogonanone C	41,460	15.1	C_20_H_20_O_6_	357.133	357.1333	0
33	Liriopesides B	256,700	15.11	C_39_H_62_O_12_	723.431	723.4309	−0.7

**Table 4:** Putative identification of ZMD in negative ion mode

No.	Component name	Area	RT	Formula	Precursor mass	Found at mass	Mass error (ppm)
1	Histidine	11,660	1.08	C_6_H_9_N_3_O_2_	154.062	154.0622	−0.1
2	Arginine	165,500	1.09	C_6_H_14_N_4_O_2_	173.104	173.1044	0.1
3	d-(+)-Mannose	376,700	1.19	C_6_H_12_O_6_	179.056	179.056	−0.8
4	l-Malic acid	2,191,000	1.35	C_4_H_6_O_5_	133.014	133.0143	0.2
5	Fungitetraose	329,200	1.79	C_24_H_42_O_21_	665.215	665.2146	0
6	Citric acid	7,804,000	1.94	C_6_H_8_O_7_	191.02	191.0196	−0.5
7	Succinic acid	21,110	2.31	C_4_H_6_O_4_	117.019	117.0192	−0.9
8	Adenine	10,630	2.37	C_5_H_5_N_5_	134.047	134.0473	0.3
9	Guanosine	115,800	2.48	C_10_H_13_N_5_O_5_	282.084	282.0845	0.3
10	Gallic acid	54,280	2.66	C_7_H_6_O_5_	169.014	169.0142	0
11	Phenylalanine	70,380	3.19	C_9_H_11_NO_2_	164.072	164.0718	0.8
12	Vanillic acid	228,400	3.37	C_8_H_8_O_4_	167.035	167.035	0.3
13	Hydroxytyrosol	11690	3.69	C_8_H_10_O_3_	153.056	153.0559	1
14	l-Tryptophan	250,300	4.16	C_11_H_12_N_2_O_2_	203.083	203.0826	0.1
15	Salidroside	31,980	4.17	C_14_H_20_O_7_	299.114	299.1138	0.7
16	4-Hydroxybenzoic acid	92,120	4.63	C_7_H_6_O_3_	137.024	137.0244	0.1
17	4-*O*-caffeoyl quinic acid	264,700	4.8	C_16_H_18_O_9_	353.088	353.0879	0.1
18	Esculetin	430,000	5.14	C_9_H_6_O_4_	177.019	177.0193	−0.3
19	Caffeic acid	309,100	5.21	C_9_H_8_O_4_	179.035	179.0348	−0.8
20	Eleutheroside E	14,750	5.88	C_34_H_46_O_18_	787.267	787.2658	−1
21	Rutin	202,800	6.3	C_27_H_30_O_16_	609.146	609.1456	−0.9
22	Hyperin	2,004,000	6.51	C_21_H_20_O_12_	463.088	463.0879	−0.7
23	Astragalin	12,120	6.96	C_21_H_20_O_11_	447.093	447.0931	−0.4
24	Specnuezhenide	872,500	7.04	C_31_H_42_O_17_	685.235	685.2342	−1
25	Narirutin	43,210	7.08	C_27_H_32_O_14_	579.172	579.1709	−1.8
26	Dicaffeoylquinic acid	34,110	7.49	C_25_H_24_O_12_	515.119	515.1189	−1.2
27	Hesperidin	161,500	7.49	C_28_H_34_O_15_	609.182	609.1816	−1.5
28	Quercetin	16,350	9.1	C_15_H_10_O_7_	301.035	301.035	−1.3
29	Calycosin-7-*o*-glucoside	20,440	9.43	C_22_H_22_O_10_	491.119	491.1178	−3.5
30	Apigenin	6,976	10.1	C_15_H_10_O_5_	269.046	269.0448	−2.9
31	Butylparaben	2,508	13.24	C_11_H_14_O_3_	193.087	193.0869	−0.7
32	Liriope muscari baily saponins C	5,340	13.53	C_44_H_70_O_17_	869.454	869.4516	−2.8
33	Asiatic acid	31,510	14.06	C_30_H_48_O_5_	487.343	487.3414	−3
34	Methylophiopogonone A	264,600	14.71	C_19_H_16_O_6_	339.087	339.0865	−2.7
35	Ophiopogonin D	47,450	14.83	C_44_H_70_O_16_	899.465	899.463	−1.7
36	Gingerglycolipid B	5,698	15.21	C_33_H_58_O_14_	723.381	723.3777	−4.3
37	Corosolic acid	13,550	15.55	C_30_H_48_O_4_	471.348	471.3464	−3.3
38	Ophiopogonanone C	201,100	15.72	C_19_H_16_O_7_	355.082	355.0815	−2.2
39	Oleanolic acid	20,510	16.77	C_30_H_48_O_3_	455.353	455.3524	−1.4

### 3.2 GC-MS analysis of chemical constituents of CMD and ZMD

**Figure 3:**
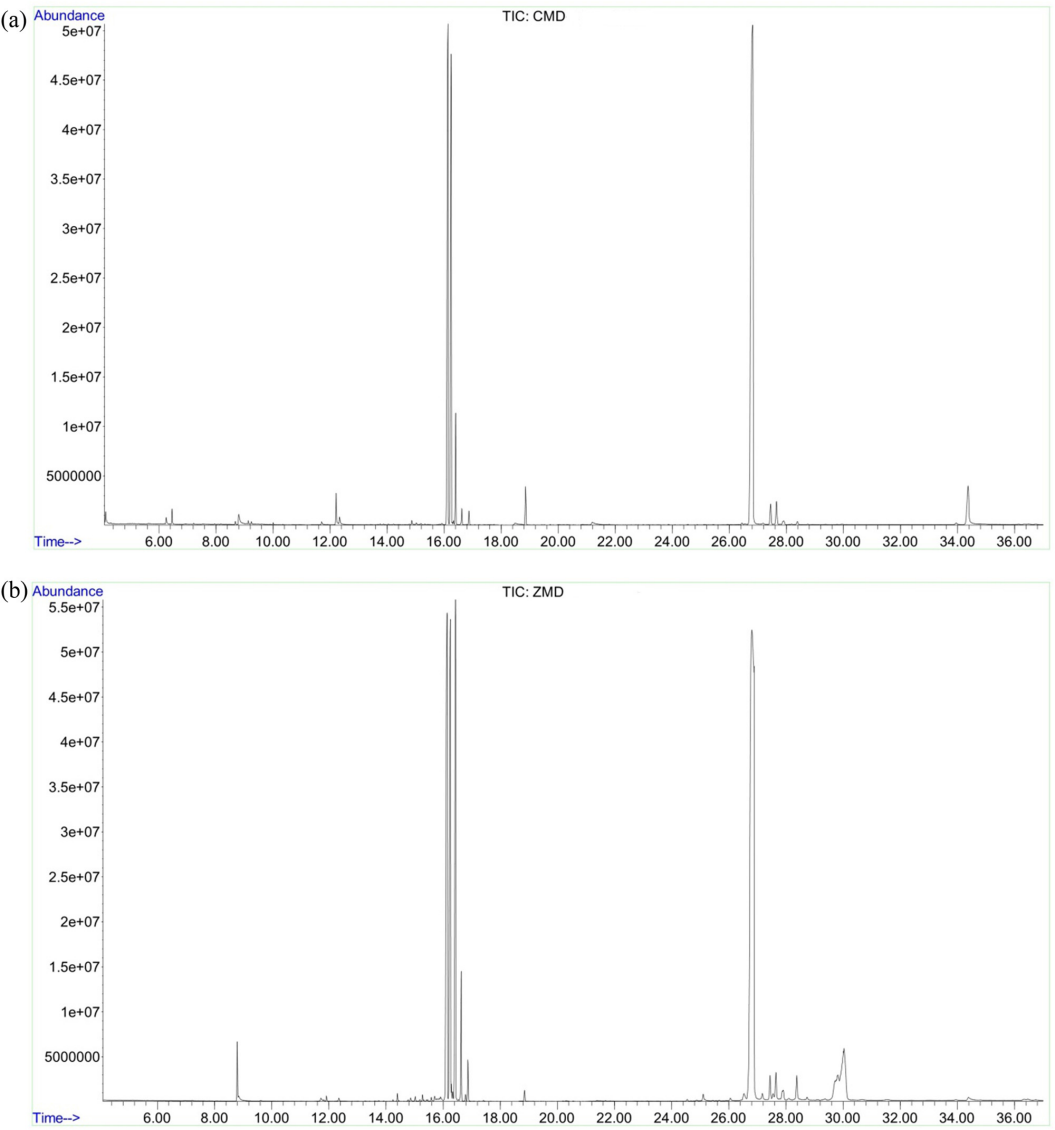
GC-MS chromatographs of CMD extract (a) and ZMD extract (b).

**Figure 4:**
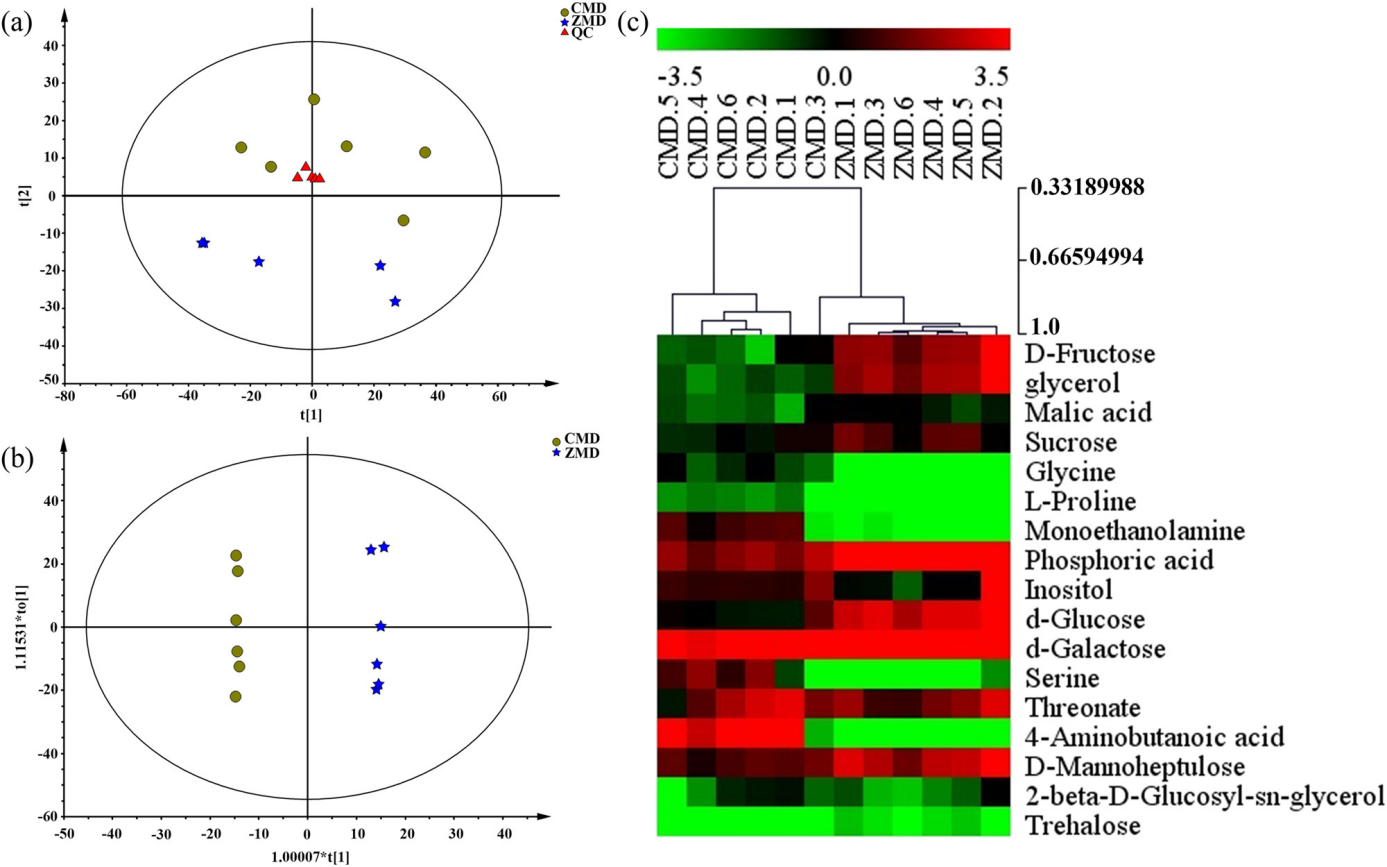
Multivariate statistical analysis of CMD and ZMD samples using GC-MS analysis: (a) PCA score plots for CMD, ZMD, and QC samples, (b) OPLS-DA score plots for CMD and ZMD, and (c) heatmap plot for the different chemical constituents of CMD and ZMD.

The total ion chromatograms of CMD and ZMD are shown in Figure 3. PCA and OPLS-DA were used to realize the CMD and ZMD clusters. In addition, the PCA score plot exhibited a relatively tight clustering of the QC samples, which confirmed the reliability of the MS data. As shown in Figure 4a, the CMD and ZMD groups were clearly separated in the PCA score plot (*R* ^2^ *X* = 0.864, *Q* ^2^ = 0.651) with four PCs. Meanwhile, an OPLS-DA model was established (*R* ^2^ *X* = 0.912, *R* ^2^ *Y* = 0.998, and *Q* ^2^ = 0.936) and showed clear discrimination between CMD and ZMD groups (Figure 4b). A heatmap plot was generated to further characterize the significant differences. Variables with variable importance in the projection (VIP) values larger than 1 were considered to be potential chemical constituents, and 17 chemical constituents were selected (Figure 4c and Table S3).

### 3.3 Pathway enrichment of different chemical constituents in GC-MS analysis

To explore the roles of different chemical constituents based on GC-MS analysis, the different chemical constituents were imported into MetaboAnalyst 5.0 (https://www.metaboanalyst.ca/), a comprehensive platform dedicated to metabolomics data analysis via a user-friendly, web-based interface [14]. The impact value threshold was set to 0.1, and pathways with an impact value greater than the threshold were considered potential target pathways. As shown in Table 5, the therapeutic effect of CMD and ZMD was probably associated with galactose metabolism; starch and sucrose metabolism; cyanoamino acid metabolism; methane metabolism; aminoacyl-tRNA biosynthesis; glycine, serine, and threonine metabolism; arginine and proline metabolism; amino sugar and nucleotide sugar metabolism; sulfur metabolism; and glycerolipid metabolism (Table 5).

**Table 5:** Pathways of significantly different chemical constituents in GC-MS analysis

No.	Pathway name	Metabolite	KEGG ID
1	Galactose metabolism	Glycerol	C00116
		d-Galactose	C00124
		d-Glucose	C00031
		Sucrose	C00089
2	Starch and sucrose metabolism	Trehalose	C01083
		beta-d-Fructose	C02336
		d-Glucose	C00031
		Sucrose	C00089
3	Cyanoamino acid metabolism	Glycine	C00037
		l-Serine	C00065
4	Methane metabolism	Glycine	C00037
		l-Serine	C00065
5	Aminoacyl-tRNA biosynthesis	Glycine	C00037
		l-Serine	C00065
		l-Proline	C00148
6	Glycine, serine, and threonine metabolism	l-Serine	C00065
		Glycine	C00037
7	Arginine and proline metabolism	Gamma-aminobutyric acid	C00334
		l-Proline	C00148
8	Amino sugar and nucleotide sugar metabolism	d-Galactose	C00124
		beta-d-Fructose	C02336
9	Sulfur metabolism	l-Serine	C00065
10	Glycerolipid metabolism	Glycerol	C00116
11	Sphingolipid metabolism	l-Serine	C00065
12	Nitrogen metabolism	Glycine	C00037
13	Glyoxylate and dicarboxylate metabolism	l-Malic acid	C00149
14	Butanoate metabolism	Gamma-aminobutyric acid	C00334
15	Citrate cycle (TCA cycle)	l-Malic acid	C00149
16	Carbon fixation in photosynthetic organisms	l-Malic acid	C00149
17	Pyruvate metabolism	l-Malic acid	C00149
18	Alanine, aspartate, and glutamate metabolism	Gamma-aminobutyric acid	C00334
19	Glycerophospholipid metabolism	Ethanolamine	C00189
20	Glutathione metabolism	Glycine	C00037
21	Cysteine and methionine metabolism	l-Serine	C00065

### 3.4 LC-MS analysis of chemical constituents of CMD and ZMD

**Figure 5:**
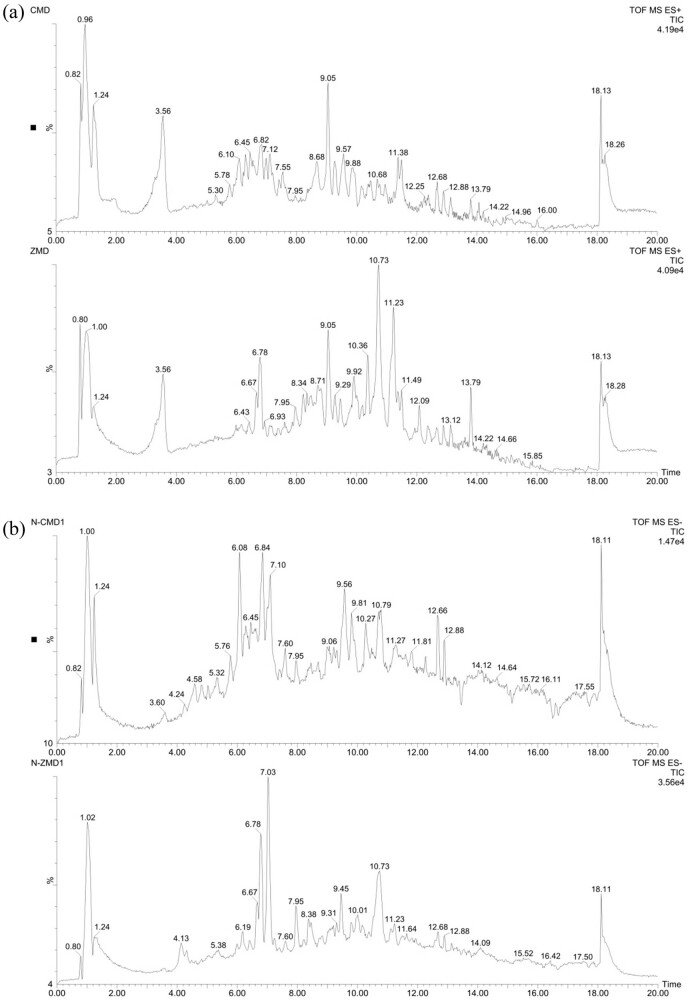
Base peak chromatograms of CMD and ZMD obtained by LC-MS in positive mode (a) and negative mode (b).

**Figure 6:**
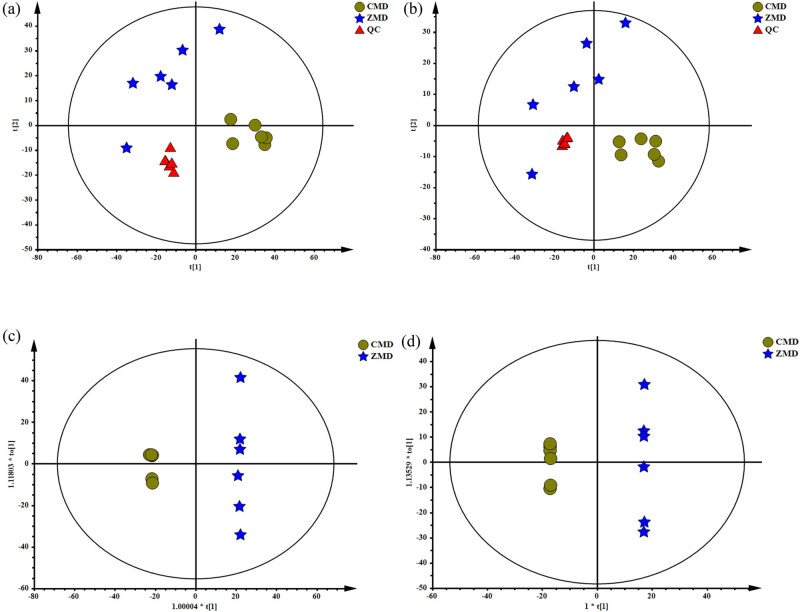
PCA and OPLS-DA score plots of CMD and ZMD samples in (a and c) positive ion mode and (b and d) negative ion mode.

**Figure 7:**
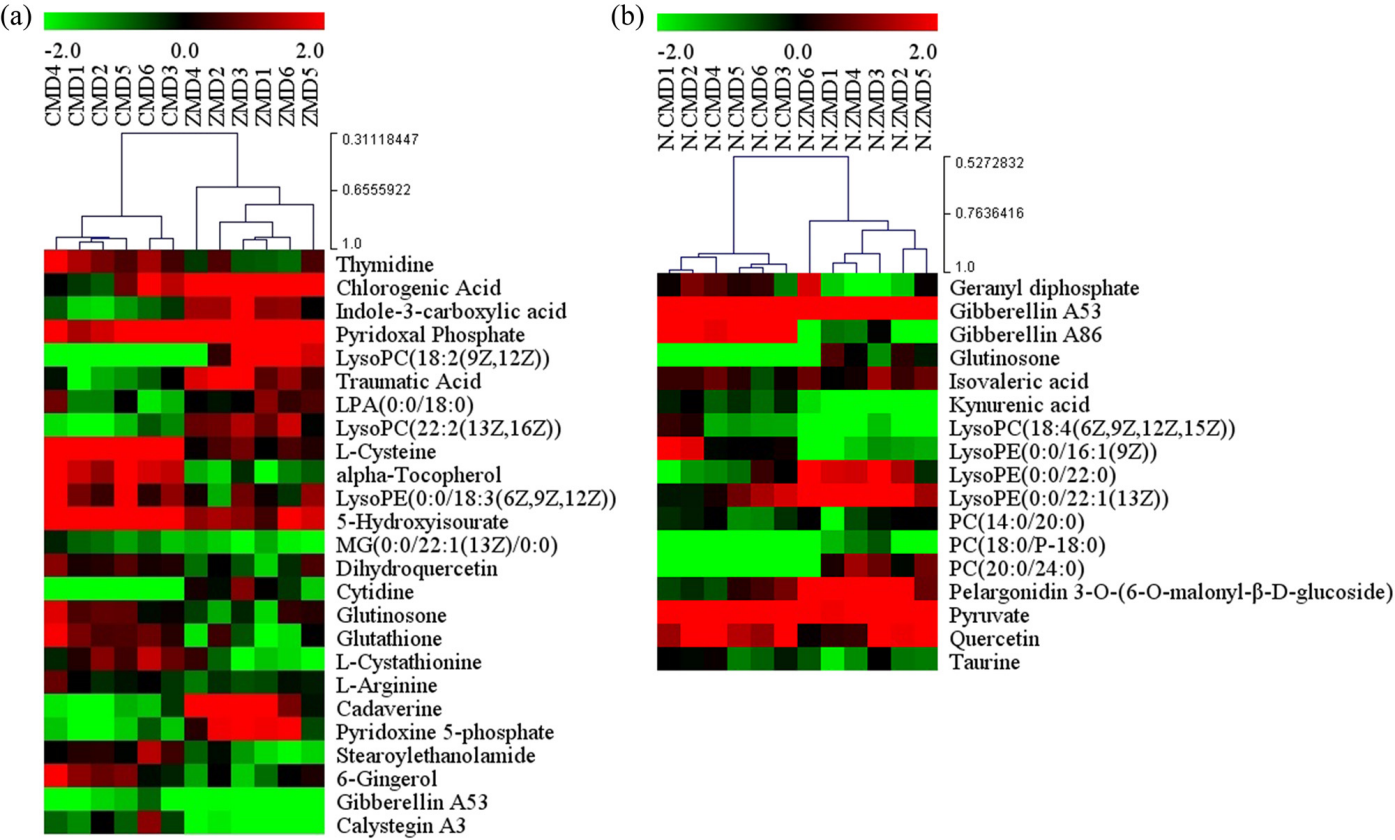
Heatmap plot for the different chemical constituents between CMD and ZMD using LC-MS analysis in (a) positive ion mode and (b) negative ion mode.

The CMD and ZMD extracts were also analyzed by LC-MS in both positive and negative ion modes. The base peak chromatograms of LC-MS are shown in Figure 5a and b. As shown in Figure 6a, the CMD and ZMD groups were also clearly separated in the PCA score plot (*R* ^2^ *X* = 0.59, *Q* ^2^ = 0.303) with three PCs. Then, the OPLS-DA model was established (*R* ^2^ *X* = 0.658, *R* ^2^ *Y* = 1, and *Q* ^2^ = 0.892) in positive ion mode (Figure 6c). And, an OPLS-DA model was established (*R* ^2^ *X* = 0.763, *R* ^2^ *Y* = 1, and *Q* ^2^ = 0.93) in negative ion mode (Figure 6d), and both showed clear discrimination of the CMD and ZMD groups in negative ion mode (*R* ^2^ *X* = 0.63, *Q* ^2^ = 0.279; Figure 6b) with three PCs. Furthermore, we found 25 differences in the chemical constituents of CMD and ZMD in positive ion mode (Figure 7a and Table S4) and a total of 17 differences in the chemical constituents of CMD and ZMD in negative ion mode (Figure 7b and Table S5).

### 3.5 Pathway enrichment of different chemical constituents in LC-MS analysis

Similarly, metabolic pathways of different chemical constituents were analyzed in LC-MS analysis using the integrated web-based tool MetaboAnalyst. In our study, the 25 different chemical constituents between CMD and ZMD in the positive ion mode were mainly associated with tropane, piperidine, and pyridine alkaloid biosynthesis; sulfur metabolism; stilbenoid, diarylheptanoid, and gingerol biosynthesis; and cysteine and methionine metabolism (Table 6). Moreover, the 17 differences in the chemical constituents of CMD and ZMD in the negative ion mode were related to valine, leucine, and isoleucine biosynthesis; diterpenoid biosynthesis; pantothenate and CoA biosynthesis; flavonoid biosynthesis; glycolysis or gluconeogenesis; carbon fixation in photosynthetic organisms; and glycerophospholipid metabolism (Table 7). These data suggested that there were many differences between CMD and ZMD. Additionally, CMD and ZMD likely act to nourish yin through all three signaling pathways.

**Table 6:** Pathways of significantly different chemical constituents (ESI+)

No.	Pathway name	Metabolite	KEGG ID
1	Tropane, piperidine, and pyridine alkaloid biosynthesis	Cadaverine	C01672
2	Sulfur metabolism	l-Cysteine	C00097
3	Stilbenoid, diarylheptanoid, and gingerol biosynthesis	Chlorogenic acid	C00852
4	Cysteine and methionine metabolism	l-Cysteine	C00097
		l-Cystathionine	C02291
5	Glycerophospholipid metabolism	Phosphatidate	C00416
		2-Lysophosphatidylcholine	C04230
6	Phenylpropanoid biosynthesis	Chlorogenic acid	C00852
7	Diterpenoid biosynthesis	Gibberellin A53	C06094
8	Arginine and proline metabolism	l-Arginine	C00062
9	Thiamine metabolism	l-Cysteine	C00097
10	Aminoacyl-tRNA biosynthesis	l-Arginine	C00062
		l-Cysteine	C00097
11	Flavonoid biosynthesis	Taxifolin	C01617
		Chlorogenic acid	C00852
12	Purine metabolism	5-Hydroxyisourate	C11821
13	Glutathione metabolism	l-Cysteine	C00097
		Cadaverine	C01672
		Glutathione	C00051
14	Glycerolipid metabolism	Phosphatidate	C00416
15	Pyrimidine metabolism	Cytidine	C00475
		Thymidine	C00214
16	Ubiquinone and other terpenoid-quinone biosynthesis	Alpha-tocopherol	C02477
17	Vitamin B6 metabolism	Pyridoxal 5′-phosphate	C00018
		Pyridoxine 5′-phosphate	C00627

**Table 7:** Pathways of significantly different chemical constituents (ESI−)

No.	Pathway name	Metabolite	KEGG ID
1	Valine, leucine, and isoleucine biosynthesis	Pyruvic acid	C00022
2	Butanoate metabolism	Pyruvic acid	C00022
3	Pyruvate metabolism	Pyruvic acid	C00022
4	Diterpenoid biosynthesis	Gibberellin A53	C06094
5	Pantothenate and CoA biosynthesis	Pyruvic acid	C00022
6	Flavonoid biosynthesis	Quercetin	C00389
7	Glycolysis or gluconeogenesis	Pyruvic acid	C00022
8	Carbon fixation in photosynthetic organisms	Pyruvic acid	C00022
9	Glycerophospholipid metabolism	2-Lysophosphatidylcholine	C04230
		Phosphatidylcholine	C00157
10	Alanine, aspartate, and glutamate metabolism	Pyruvic acid	C00022
11	Citrate cycle (TCA cycle)	Pyruvic acid	C00022
12	C5-branched dibasic acid metabolism	Pyruvic acid	C00022
13	Terpenoid backbone biosynthesis	Pyruvic acid	C00022
		Geranyl-PP	C00341
14	Flavone and flavonol biosynthesis	Quercetin	C00389
15	Glycine, serine, and threonine metabolism	Pyruvic acid	C00022
16	Monoterpenoid biosynthesis	Geranyl-PP	C00341
17	Taurine and hypotaurine metabolism	Taurine	C00245
18	Cysteine and methionine metabolism	Pyruvic acid	C00022

## 4 Discussion

The tubers of Ophiopogonis Radix (Maidong in Chinese) are an important Chinese herb and functional health food. However, the quality of CMD and ZMD remains to be distinguished and evaluated. In this respect, UPLC-Q/TOF-MS provides accurate structural information about bioactive compounds for the identification of TCM [15]. In addition, metabolomics provides new insights into understanding global metabolic changes and the multiple related biochemical pathways of altered metabolites [16,17]. GC-MS and LC-MS have become two of the most commonly used high-throughput technologies in metabolomics research due to their high sensitivity and favorable reproducibility [16,18,19]. Due to the complexity of chemical components in MD, it is difficult for traditional methods to thoroughly isolate trace ingredients with a single method. Therefore, multiple analytical platforms are needed.

In this study, efficient and reliable methods based on UPLC-Q/TOF-MS, GC-MS, and LC-MS analyses were used to identify the bioactive chemical constituents in CMD and ZMD. For UPLC-Q/TOF-MS analysis, UPLC-Q/TOF-MS technology has greatly improved the speed of analysis and detection in plants [20]. Overall, a total of 59 and 72 chemical constituents were quickly identified in CMD and ZMD, respectively, including steroidal saponins, homoisoflavonoids, amino acids, and nucleosides. Among them, isoleucine, chlorogenic acid, daphnetin, rutin, isoscopoletin, luteolin, genistein, hesperidin, pratense-7-*O*-glucoside, farrerol, patchouli alcohol, diosgenin, arginine, fungitetraose, adenine, hydroxytyrosol, salidroside, eleutheroside E, astragalin, specnuezhenide, dicaffeoylquinic acid, quercetin, apigenin, butylparaben, asiatic acid, and oleanolic acid existed only in ZMD, while glutamic acid, betaine, cinnamic acid, syringaldehyde, neohesperidin, leucine, shikimic acid, ellagic acid, gracillin, and methylophiopogonanone B existed only in CMD. In general, these results showed that there were many differences between the bioactive chemical constituents of Ophiopogonis Radix from different production areas.

Metabolomics can help to assess the physiological state of an organism in diverse biochemical events [21]. Previously, Lyu et al. reported that *O. japonicas* from Zhejiang and Sichuan can clearly be separated by using UPLC/Q-TOF MS-based metabolome analysis where CMD showed higher level steroidal saponins, and ZMD had higher contents of homoisoflavonoids specifically [20]. Similarly,i this study, for GC-MS and LC-MS-based metabolome analyses, the PCA results showed that the CMD and ZMD samples were divided into two clusters and indicated that metabolite profiling by GC-MS and LC-MS also contributes to discriminating CMD and ZMD samples from different geographical origins. Moreover, the OPL-DA and VIP values revealed that the bioactive chemical constituents in CMD and ZMD were significantly different. Among them, 4-aminobutanoic acid, glycine, l-proline, monoethanolamine, and serine showed higher levels in CMD according to the results of GC-MS analysis. In addition, the contents of chlorogenic acid, traumatic acid, cytidine, cadaverine, pyridoxine 5-phosphate, glutinone, and pelargonidin 3-*O*-(6-*O*-malonyl-β-d-glucoside) were remarkably higher than those in CMD. Moreover, these different constituents were mainly associated with multiple metabolic pathways, such as galactose metabolism; starch and sucrose metabolism; cysteine and methionine metabolism; valine, leucine, and isoleucine biosynthesis; and glycerophospholipid metabolism. Significantly, galactose is crucial for human metabolism, with an established role in energy delivery and the galactosylation of complex molecules [22]. Additionally, sucrose plays a central role in the control of carbon flux in the biosynthesis of different storage reserves [23]. Xu et al. showed that methionine restriction, a dietary regimen that protects against metabolic diseases and aging, represses cancer growth and improves cancer therapy [24]. Interestingly, leucine and isoleucine reduced body weight and white adipose tissue weight by regulating lipid metabolism-related genes in high-fat diet-induced obese mice [25]. Overall, the bioactive chemical constituents in CMD and ZMD are involved in diverse metabolic pathways with different pharmacological effects. However, there are some limitations to this study. The number of samples is too small to be representative for multivariate statistical analysis. The sample size should be expanded for further study. In further research, we will focus on the molecular mechanisms of different chemical constituents in Maidong, which are critical for developing Maidong for pharmacology and clinical uses.

## 5 Conclusion

In summary, UPLC-Q/TOF-MS, GC-MS, and LC-MS analyses combined with multivariate statistical analysis could provide basic information for the discrimination and quality evaluation of Ophiopogonis Radix from two different production areas. These findings suggested that the ZMD samples showed higher levels of one type of bioactive chemical constituent than the CMD samples, demonstrating that the geographical area influenced the accumulation of bioactive constituents. This study also lays foundations for future studies on the quantitative analysis of the 12 bioactive chemical constituents between CMD and ZMD and their relevant metabolic pathways, which will contribute to increasing the understanding of the pharmacodynamic effects and improve the development of Ophiopogonis Radix in TCM.

## Supplementary Material

Supplementary Figure
