# Supplementary material for: Identification of active compounds in Ophiopogonis Radix from different geographical origins by UPLC-Q/TOF-MS combined with GC-MS approaches
Source: Open Life Sci. 2022 Aug 12;17(1):865–80. doi: 10.1515/biol-2022-0096 (PMC9375982; doi:10.1515/biol-2022-0096)
Supplement: Supplementary Figure [file biol-2022-0096-sm.pdf]

Table S1 MS fragmentation pathways for representative compounds of CMD in UPLC-Q/TOF-MS analysis

MS fragmentation pathways for different chemical constituents of CMD in positive ion mode

精氨酸L(+)-Arginine

**Retention Time:** 1.14 minutes<br>**Precursor m/z :** 175.1190<br>**Fit (%)** 100.0% **RFit (%)** 98.1%	**Exp RT:** 1.12 minutes<br>**Analyte Name:**<br>精氨酸L(+)-Arginine
	**Collision Energy = 35 ± 15 eV**

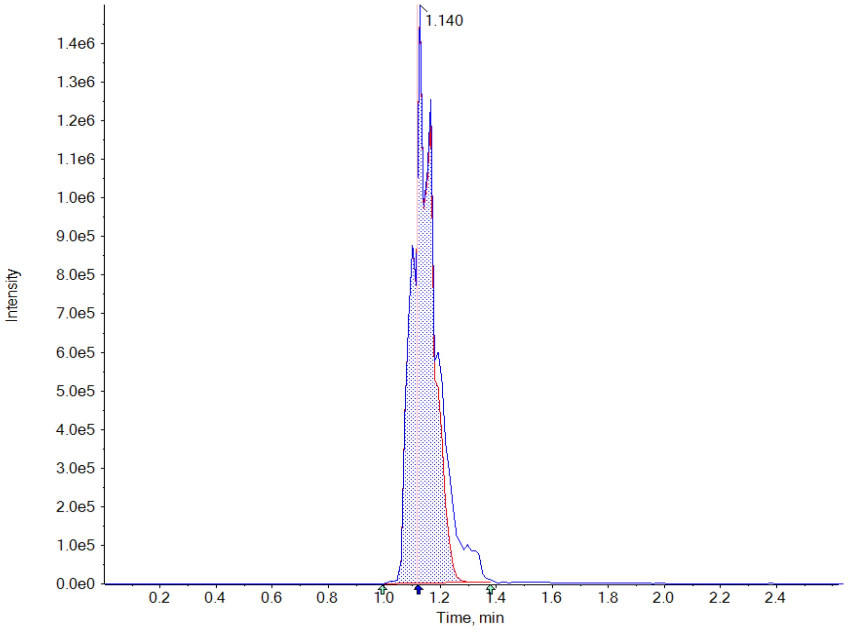

Acquired / Library MSMS

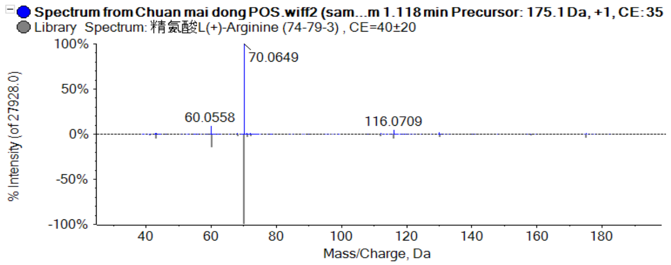

Acquired / Theoretical MS

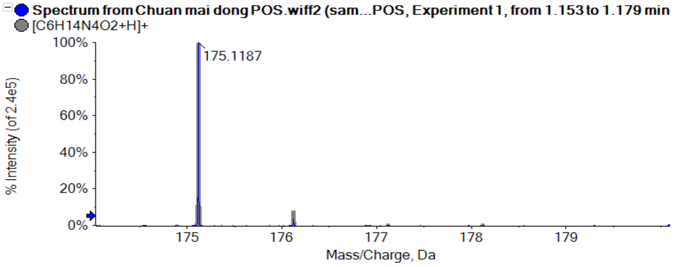

胡芦巴碱Trigonelline

Retention Time: 1.21 minutes
Precursor m/z : 138.0550
Fit (%) 99.8% **RFit (%)** 99.0%

Exp RT: 1.20 minutes
Analyte Name:
胡芦巴碱Trigonelline

Collision Energy = 35 ± 15 eV

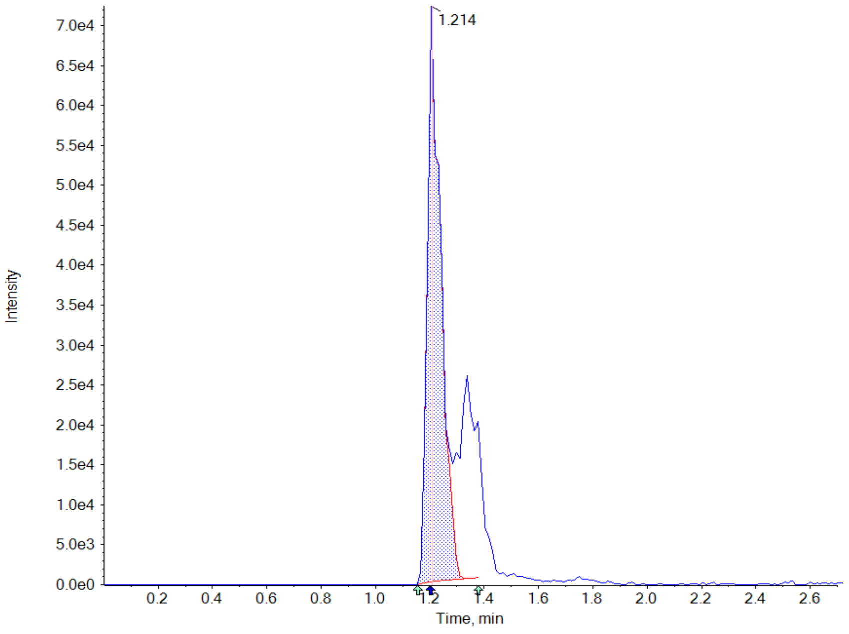

Acquired / Library MSMS

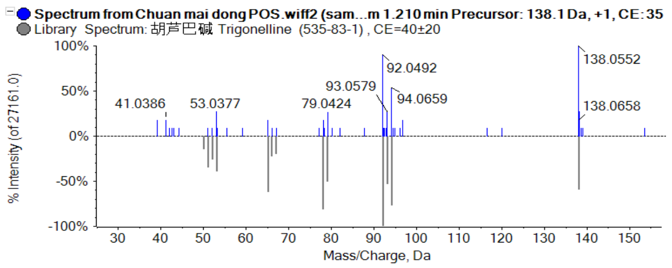

Acquired / Theoretical MS

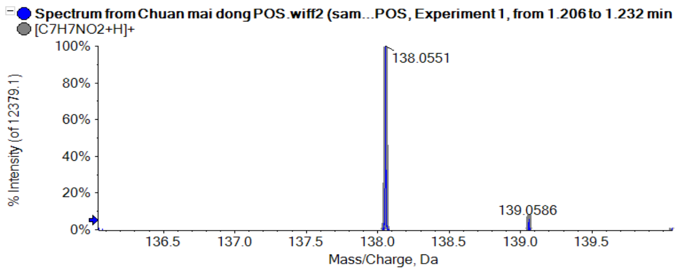

脯氨酸Proline

Retention Time: 1.24 minutes **Exp RT:** 1.22 minutes

Precursor m/z : 116.0706 **Analyte Name:**

Fit (%) 98.5% **RFit (%)** 100.0% 脯氨酸Proline

Collision Energy = 35 ± 15 eV

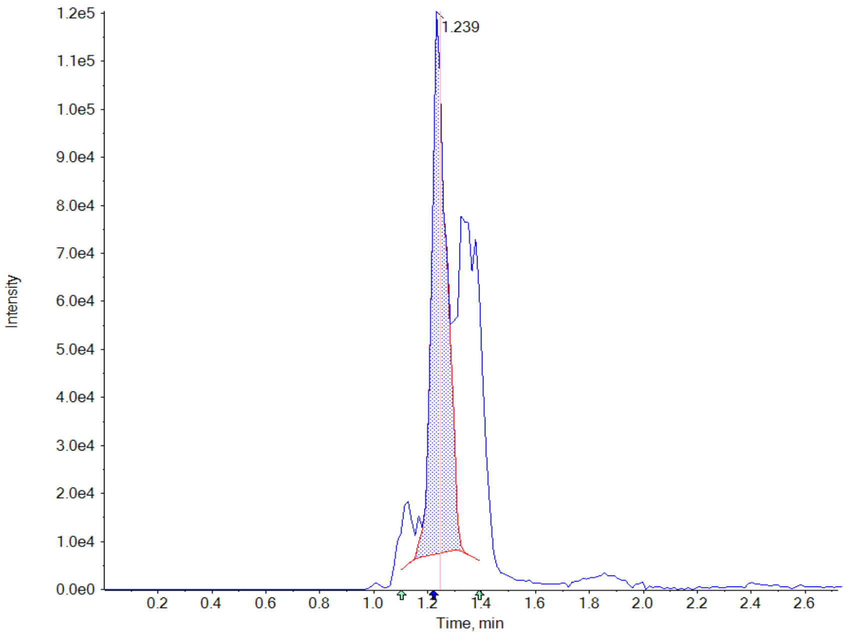

Acquired / Library MSMS

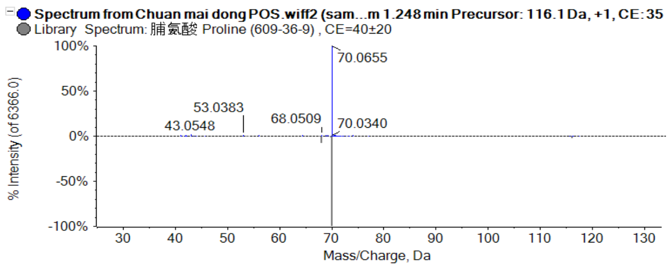

Acquired / Theoretical MS

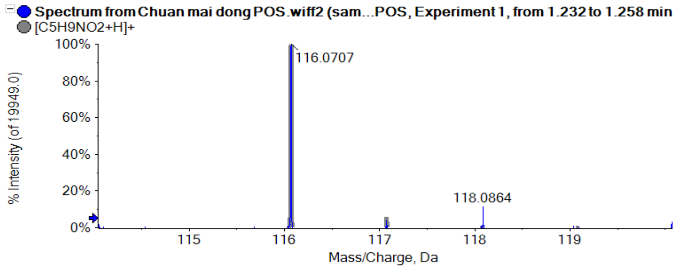

谷氨酸Glutamic acid

Retention Time: 1.30 minutes
Precursor m/z : 148.0604
Fit (%) 100.0% **RFit (%)** 92.0%

Exp RT: 1.16 minutes
Analyte Name:
谷氨酸Glutamic acid

Collision Energy = 35 ± 15 eV

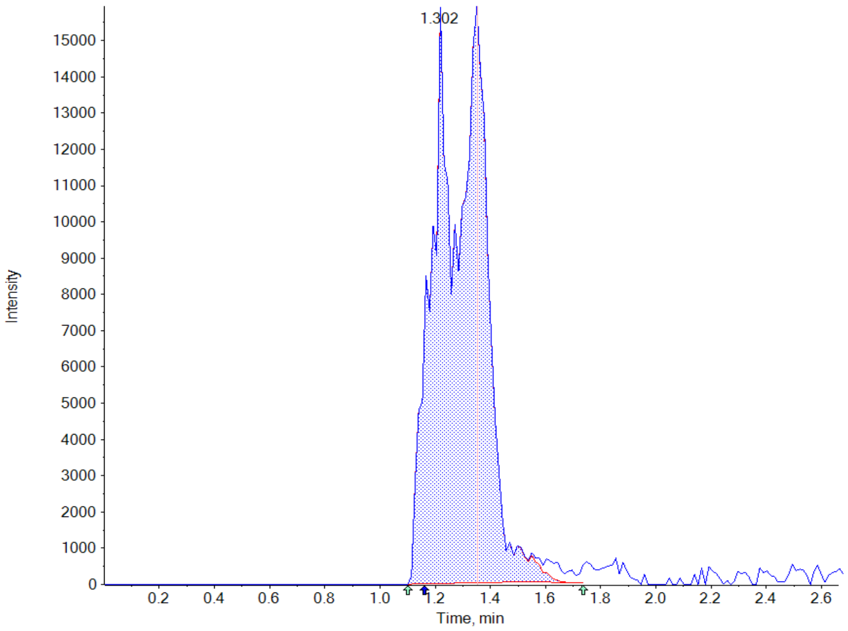

Acquired / Library MSMS

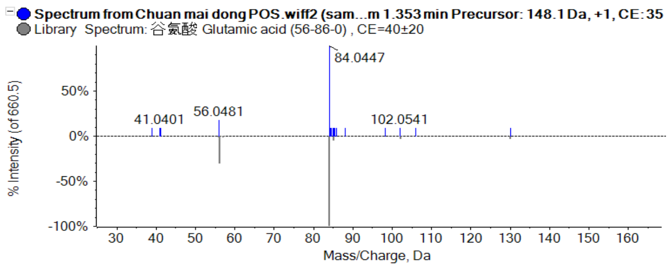

Acquired / Theoretical MS

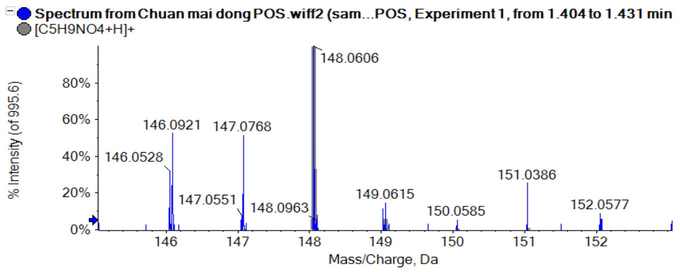

甜菜碱Betaine

Retention Time: 1.19 minutes **Exp RT:** 1.16 minutes

Precursor m/z : 118.0863 **Analyte Name:** 甜菜碱Betaine

Fit (%) 100.0% **RFit (%)** 96.6%

Collision Energy = 35 ± 15 eV

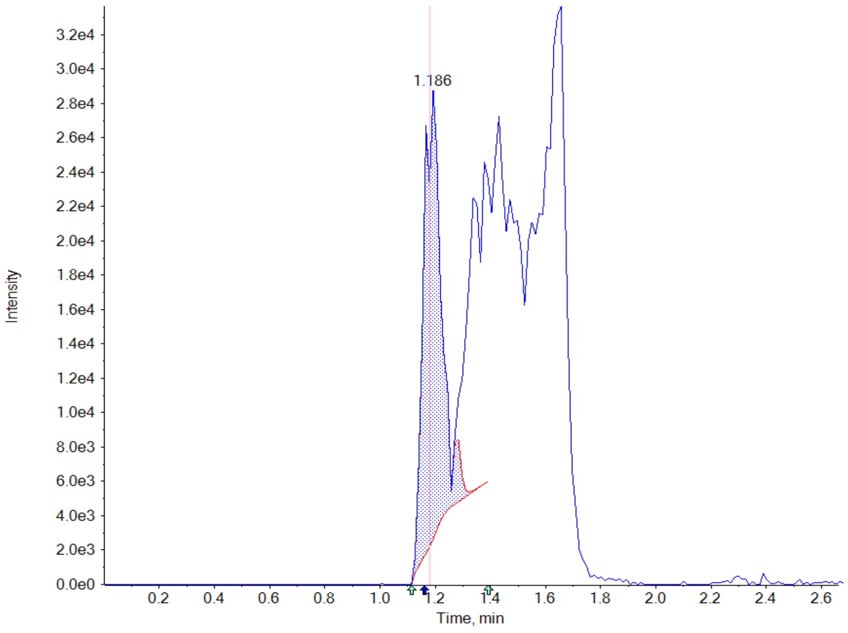

Acquired / Library MSMS

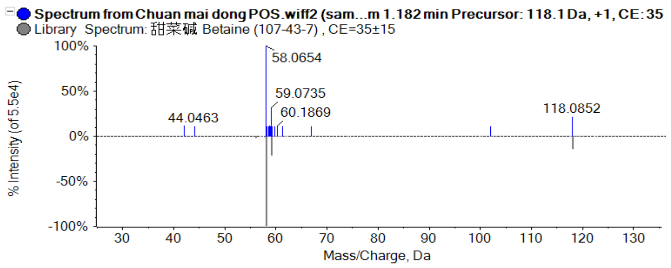

Acquired / Theoretical MS

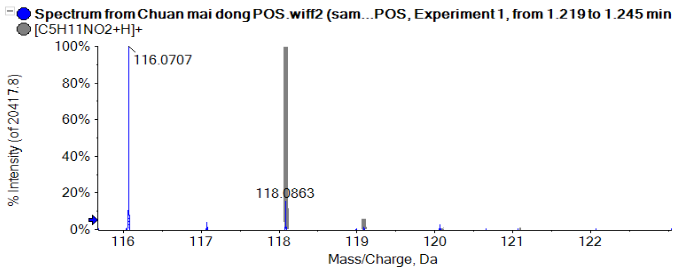

烟酸Nicotinic acid

Retention Time: 1.71 minutes
Precursor m/z : 124.0393
Fit (%) 97.2% **RFit (%)** 99.9%

Exp RT: 1.73 minutes
Analyte Name:
烟酸Nicotinic acid

Collision Energy = 35 ± 15 eV

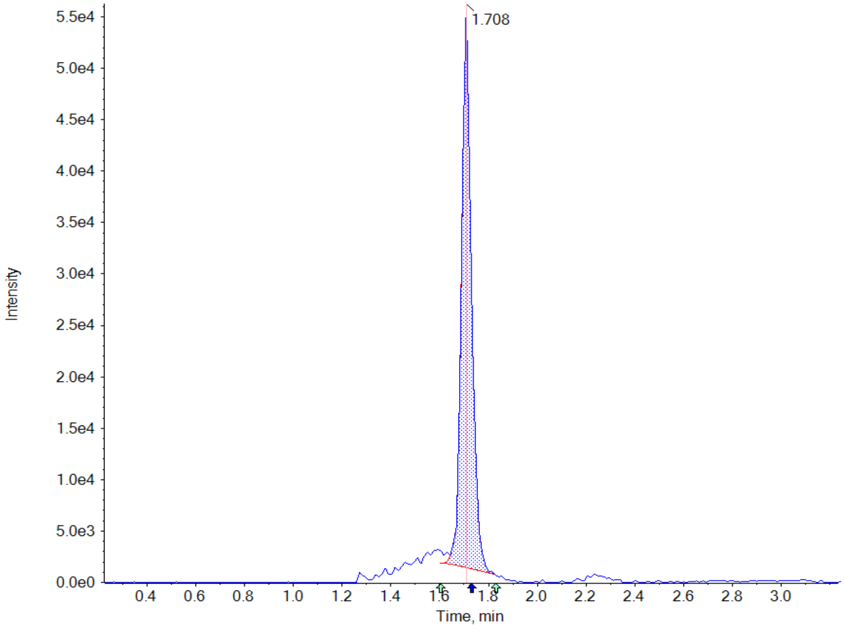

Acquired / Library MSMS

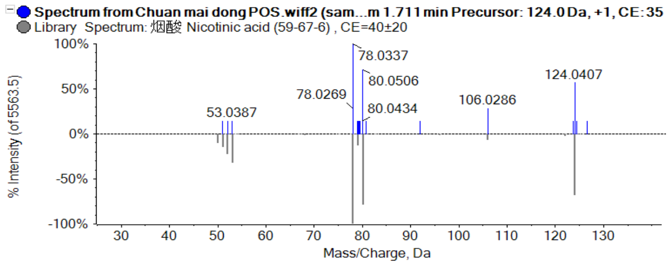

Acquired / Theoretical MS

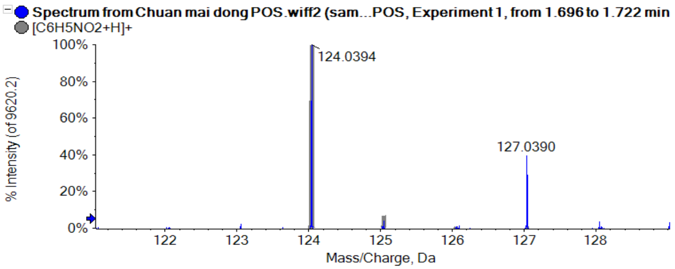

烟酰胺Nicotinamide

Retention Time: 1.79 minutes
Precursor m/z : 123.0553
Fit (%) 99.7% **RFit (%)** 98.8%

Exp RT: 1.81 minutes
Analyte Name:
烟酰胺Nicotinamide

Collision Energy = 35 ± 15 eV

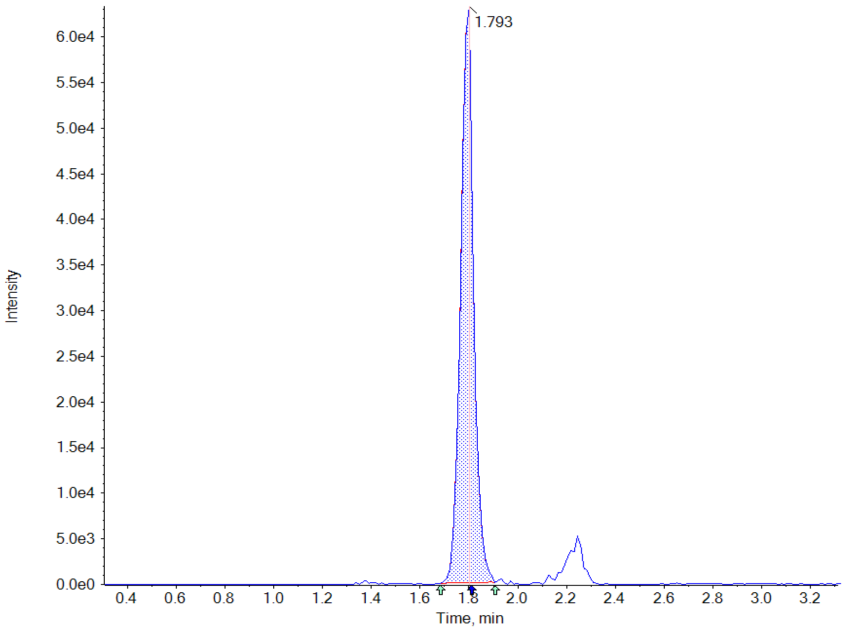

Acquired / Library MSMS

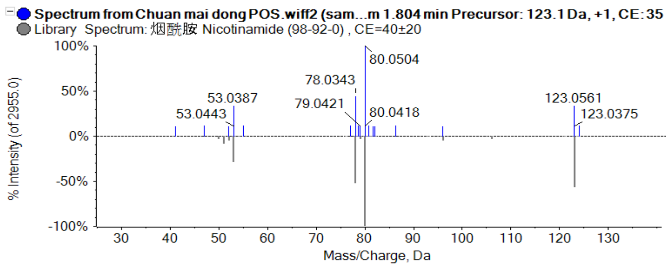

Acquired / Theoretical MS

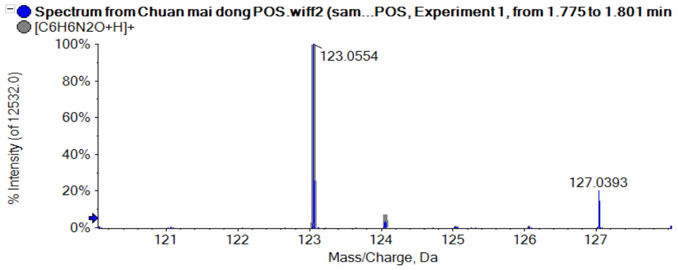

腺苷Adenosine

**Retention Time:** 2.36 minutes	**Exp RT:** 2.42 minutes
**Precursor m/z :** 268.1040	**Analyte Name:**
**Fit (%)** 98.0% **RFit (%)** 100.0%	腺苷Adenosine

Collision Energy = 35 ± 15 eV

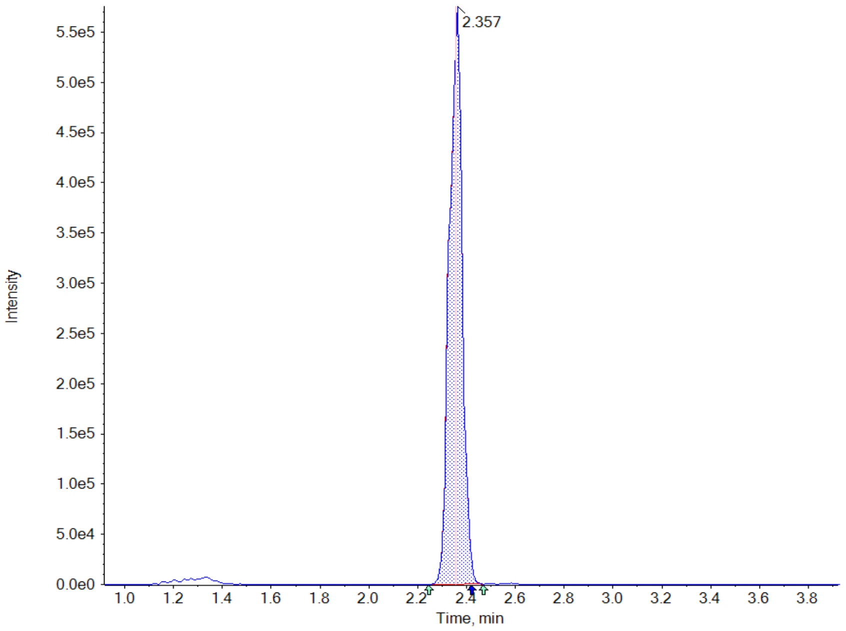

Acquired / Library MSMS

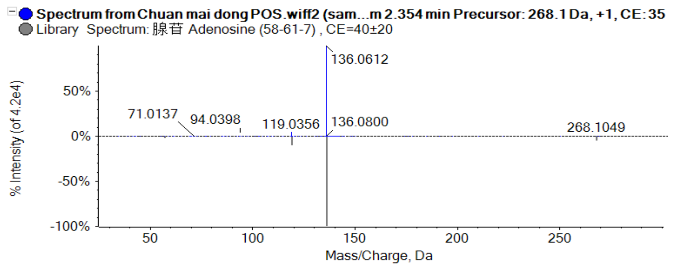

Acquired / Theoretical MS

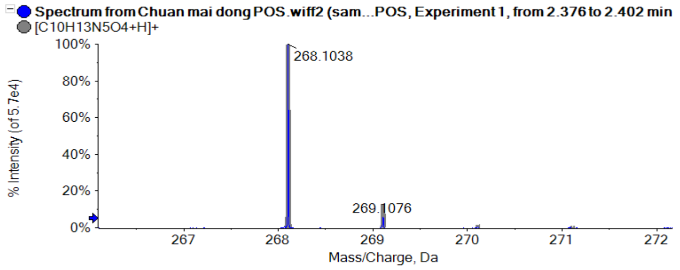

虫草素Cordycepin

Retention Time: 2.42 minutes
Precursor m/z : 252.1091
Fit (%) 98.5% **RFit (%)** 100.0%

Exp RT: 2.42 minutes
Analyte Name:
虫草素Cordycepin

Collision Energy = 35 ± 15 eV

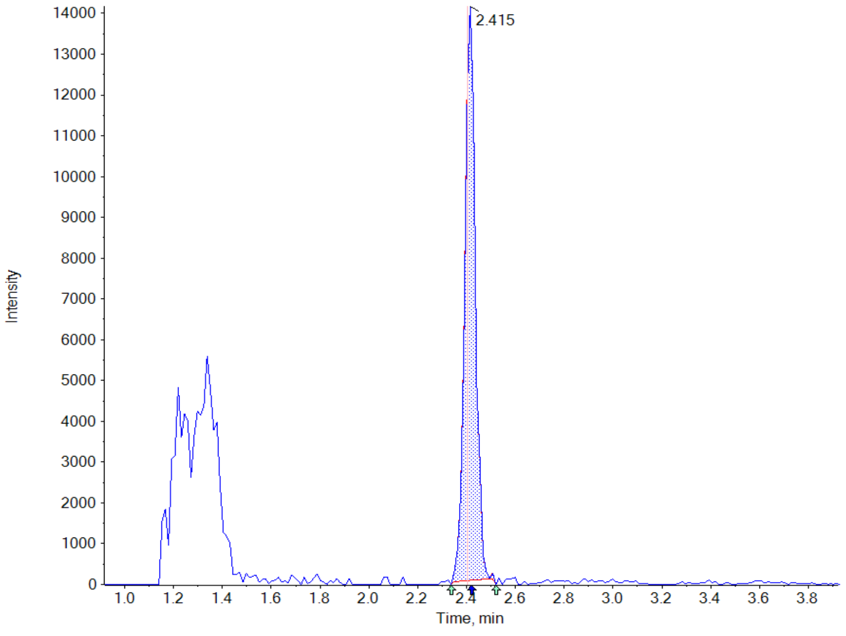

Acquired / Library MSMS

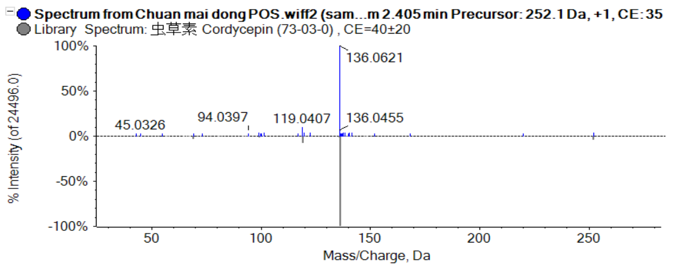

Acquired / Theoretical MS

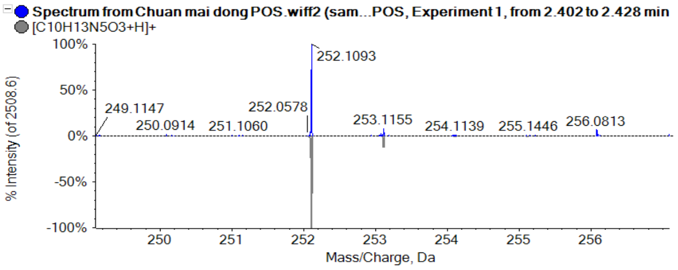

鸟苷Guanosine

Retention Time: 2.46 minutes
Precursor m/z : 284.0989
Fit (%) 99.1% **RFit (%)** 100.0%

Exp RT: 2.56 minutes
Analyte Name:
鸟苷Guanosine

Collision Energy = 35 ± 15 eV

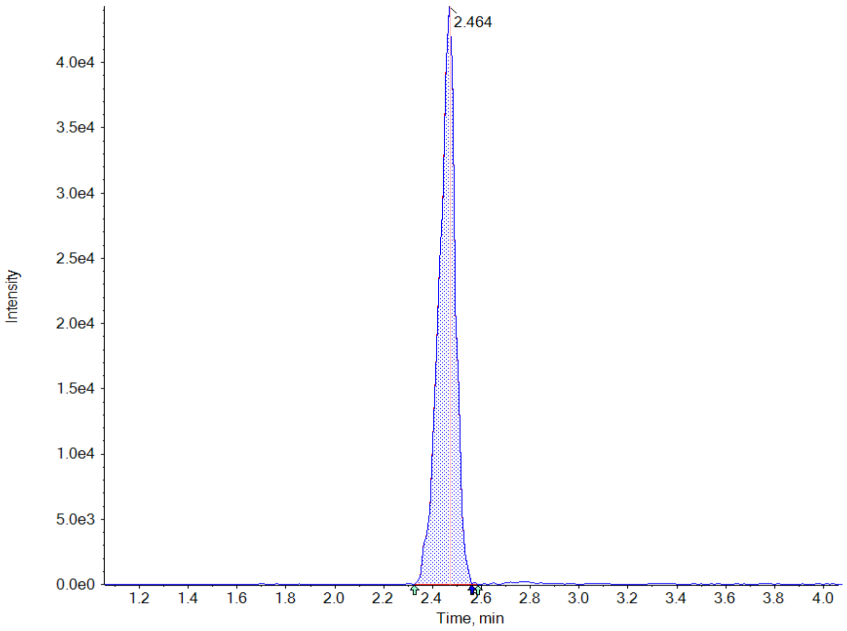

Acquired / Library MSMS

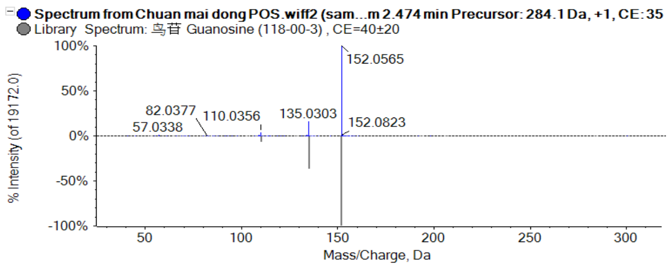

Acquired / Theoretical MS

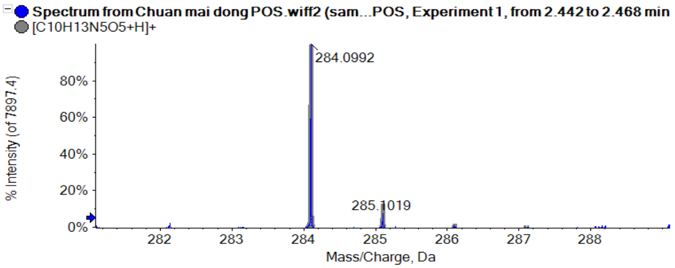

苯丙氨酸Phenprobamate

**Retention Time:** 3.17 minutes<br>**Precursor m/z :** 166.0863<br>**Fit (%)** 99.7% **RFit (%)** 100.0%	**Exp RT:** 3.31 minutes<br>**Analyte Name:**<br>苯丙氨酸Phenprobamate

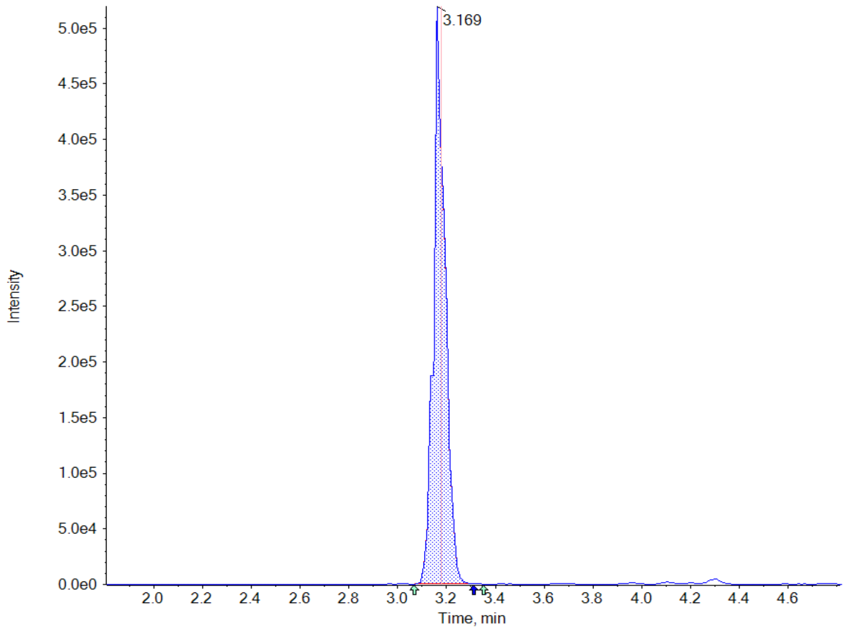

Collision Energy = 35 ± 15 eV

Acquired / Library MSMS

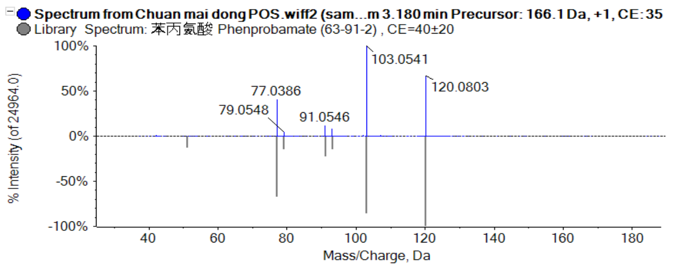

Acquired / Theoretical MS

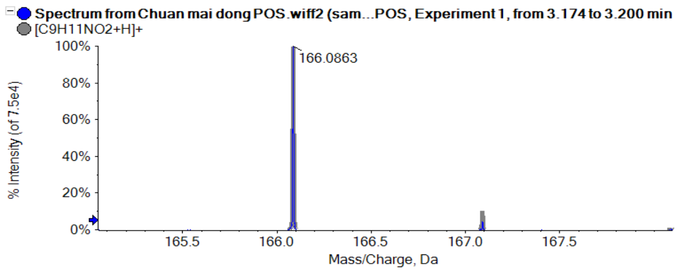

肉桂酸Cinnamic acid

Retention Time: 3.18 minutes
Precursor m/z : 149.0597
Fit (%) 96.8% **RFit (%)** 91.9%

Exp RT: 3.31 minutes
Analyte Name:
肉桂酸Cinnamic acid

Collision Energy = 35 ± 15 eV

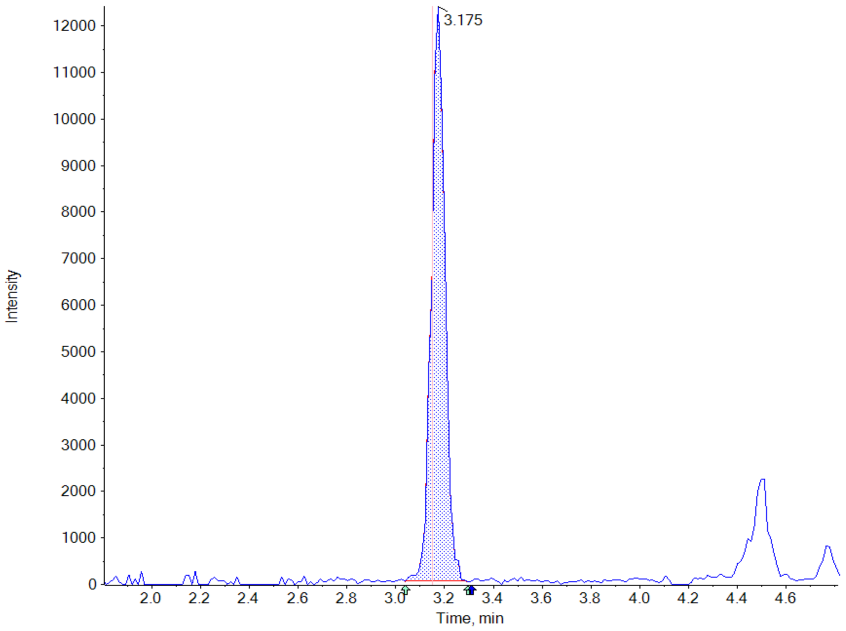

Acquired / Library MSMS

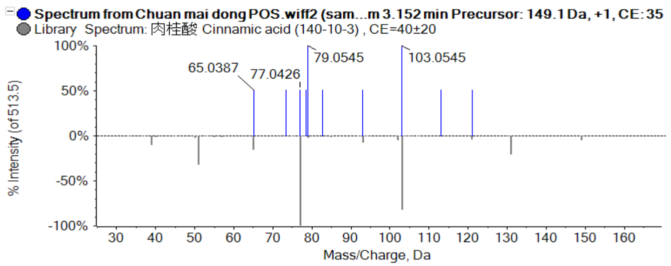

Acquired / Theoretical MS

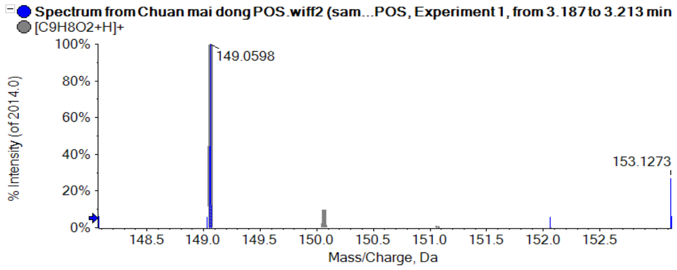

对羟基苯甲酸4-Hydroxybenzoic acid

Retention Time: 4.73 minutes

Precursor m/z : 139.0390

Fit (%) 76.9% **RFit (%)** 98.6%

Exp RT: 4.81 minutes

Analyte Name: 对羟基苯甲酸4-Hydroxybenzoic acid

Collision Energy = 35 ± 15 eV

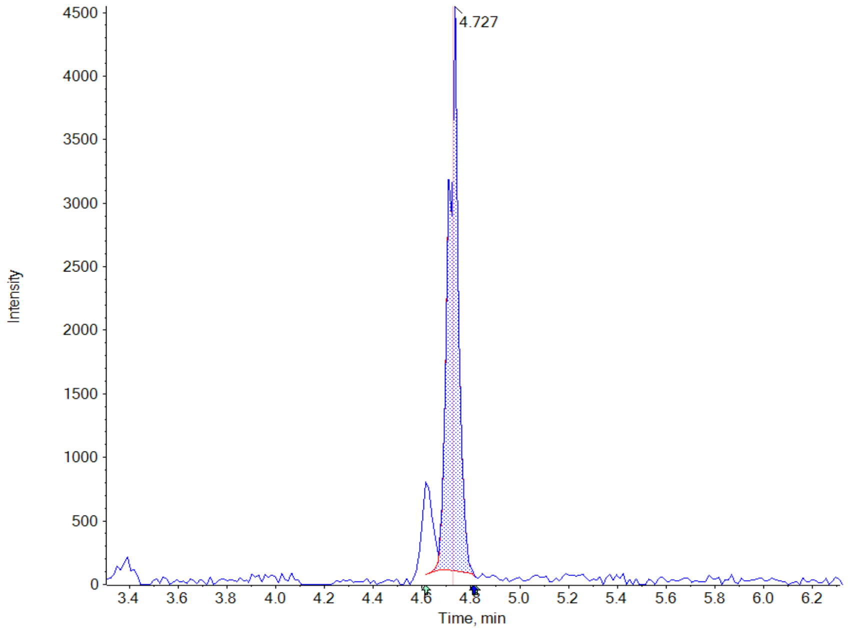

Acquired / Library MSMS

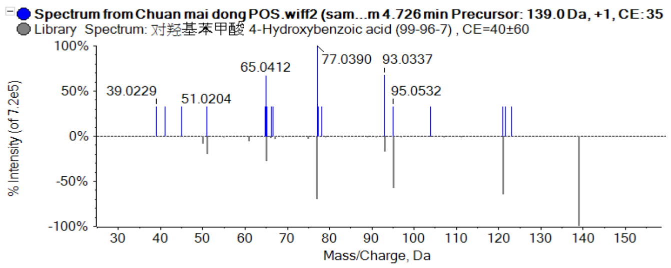

Acquired / Theoretical MS

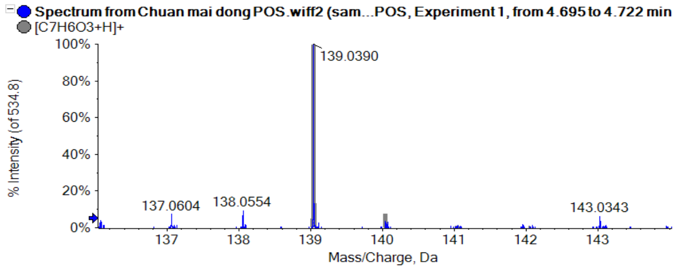

秦皮乙素Esculetin

Retention Time: 5.13 minutes **Exp RT:** 5.32 minutes

Precursor m/z : 179.0339 **Analyte Name:**

Fit (%) 93.9% **RFit (%)** 100.0% 秦皮乙素Esculetin

Collision Energy = 35 ± 15 eV

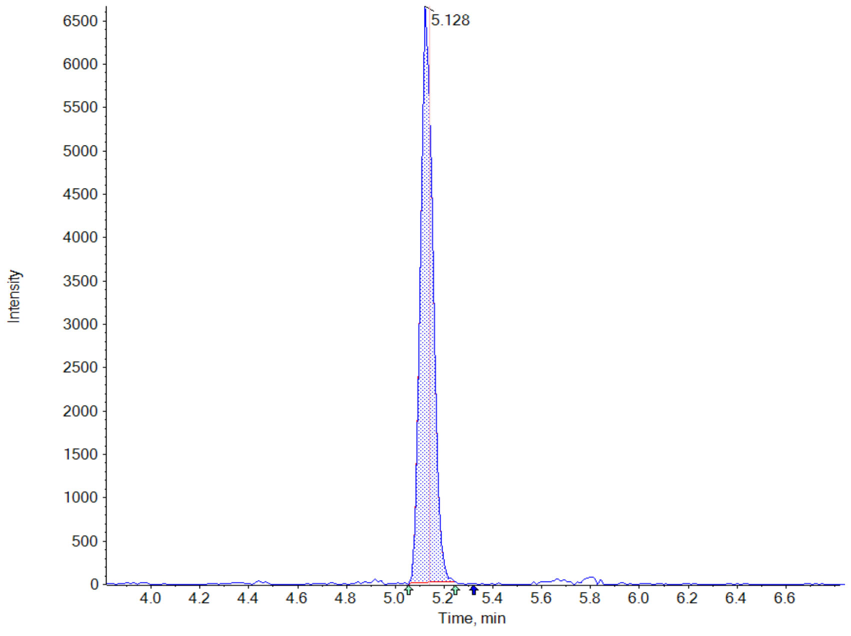

Acquired / Library MSMS

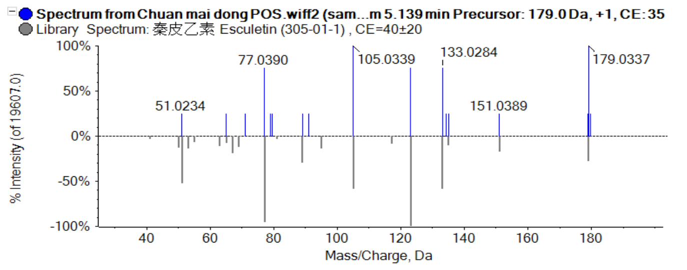

Acquired / Theoretical MS

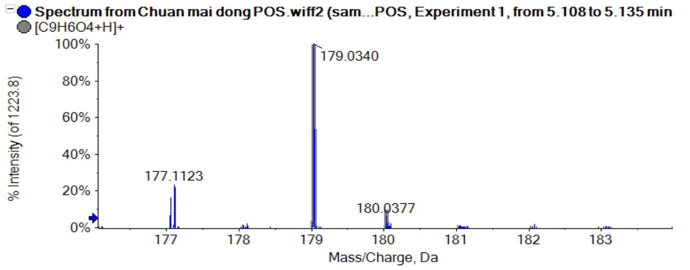

金丝桃苷Hyperin

Retention Time: 6.50 minutes
Precursor m/z : 465.1028
Fit (%) 100.0% **RFit (%)** 100.0%

Exp RT: 6.50 minutes
Analyte Name:
金丝桃苷Hyperin

Collision Energy = 35 ± 15 eV

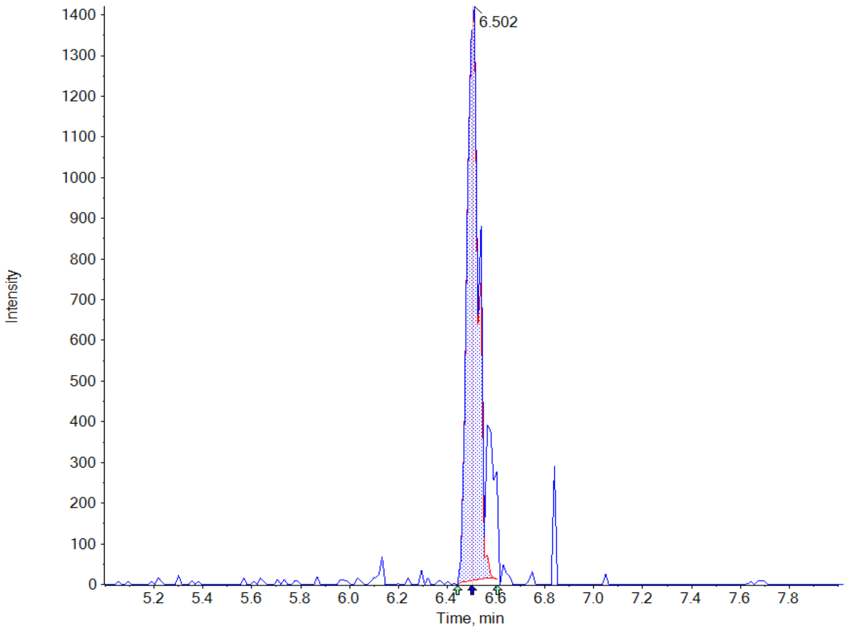

Acquired / Library MSMS

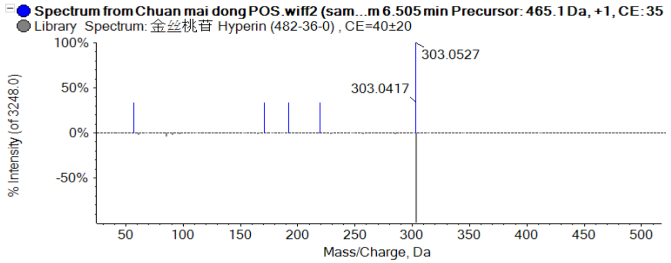

Acquired / Theoretical MS

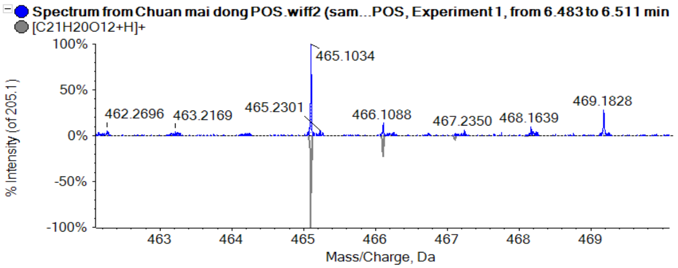

丁香醛Syringaldehyde

Retention Time: 6.56 minutes
Precursor m/z : 183.0652
Fit (%) 96.7% **RFit (%)** 97.1%

Exp RT: 6.56 minutes
Analyte Name:
丁香醛Syringaldehyde

Collision Energy = 35 ± 15 eV

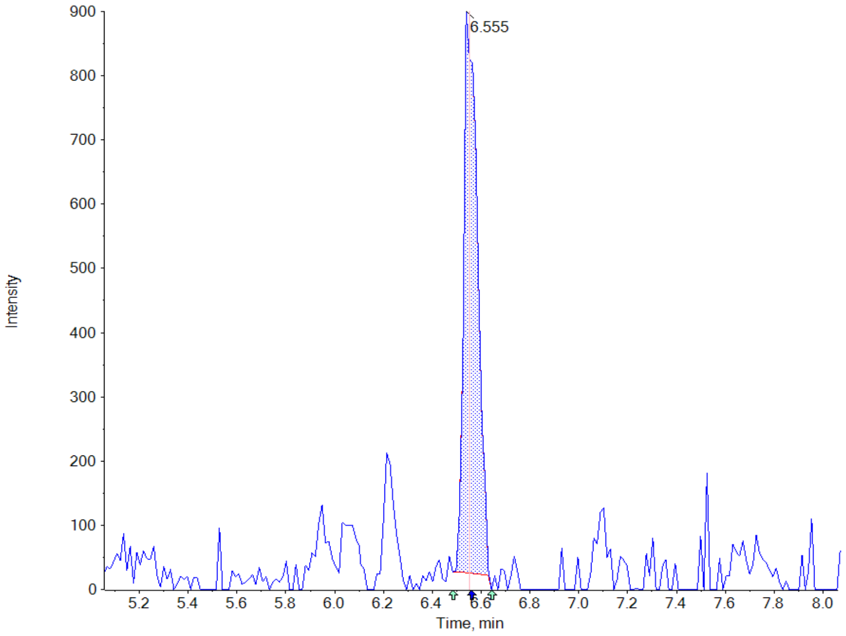

Acquired / Library MSMS

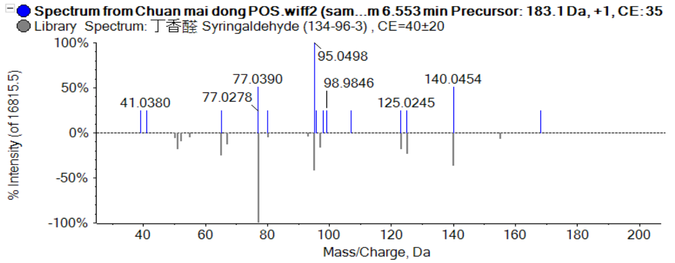

Acquired / Theoretical MS

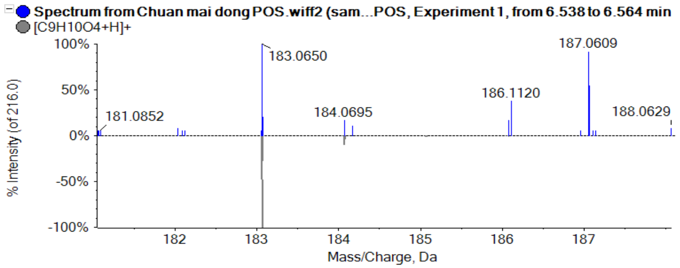

木犀草苷Luteoloside

Retention Time: 6.67 minutes
Precursor m/z : 449.1078
Fit (%) 100.0% **RFit (%)** 100.0%

Exp RT: 6.91 minutes
Analyte Name: 木犀草苷Luteoloside

Collision Energy = 35 ± 15 eV

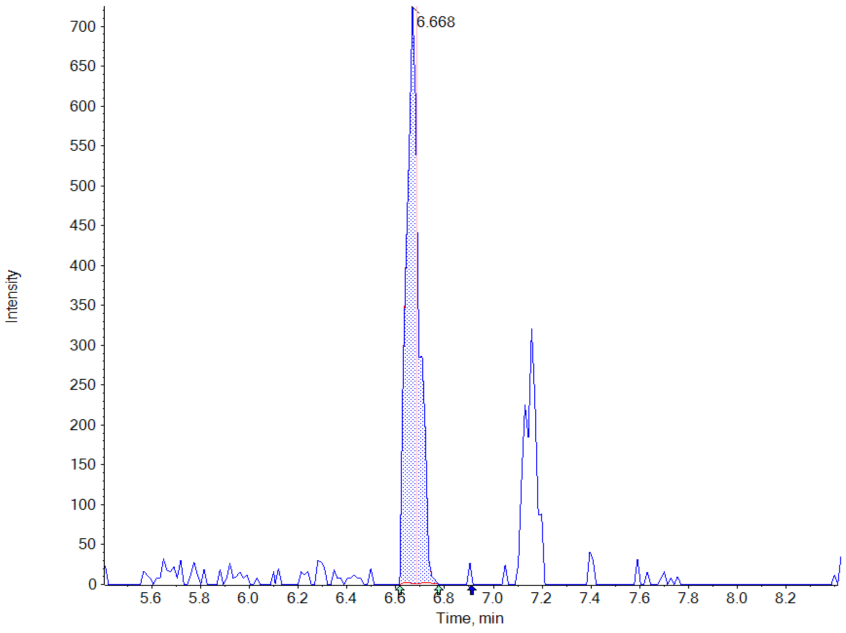

Acquired / Library MSMS

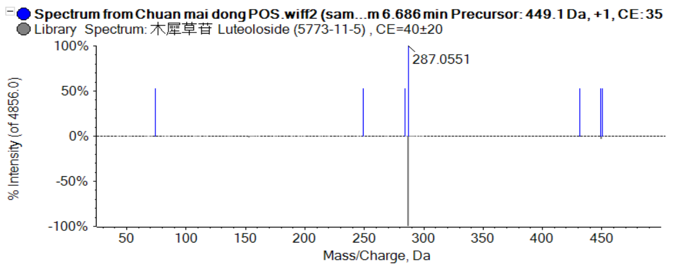

Acquired / Theoretical MS

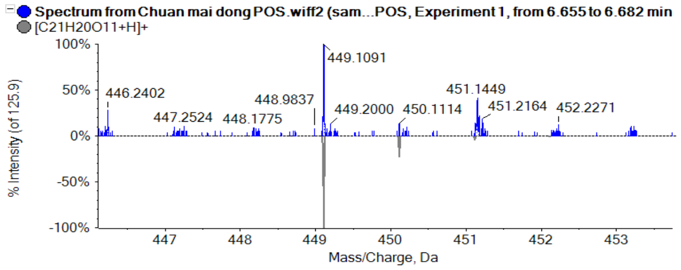

异阿魏酸Isoferulic acid

Retention Time: 6.74 minutes
Precursor m/z : 195.0652
Fit (%) 99.2% **RFit (%)** 100.0%

Exp RT: 6.75 minutes
Analyte Name:
异阿魏酸Isoferulic acid

Collision Energy = 35 ± 15 eV

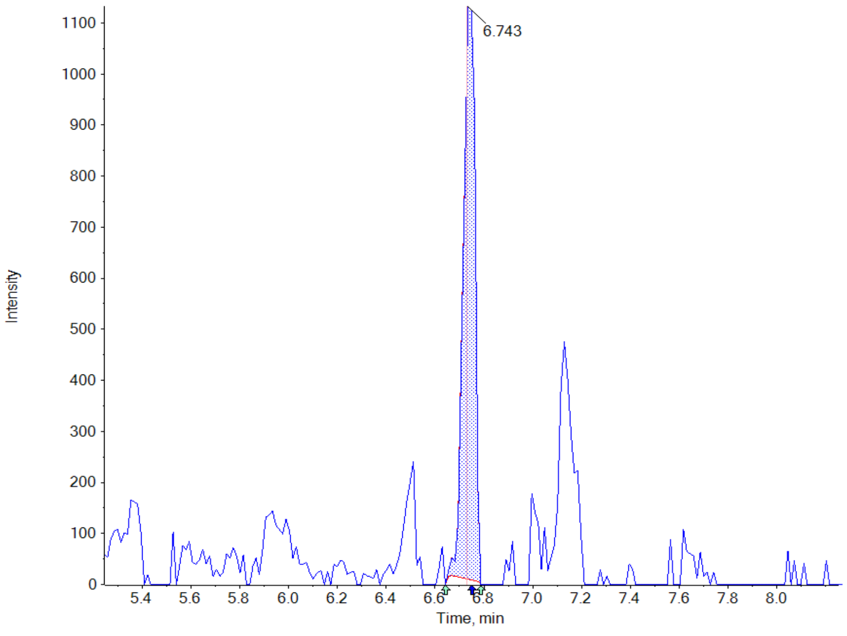

Acquired / Library MSMS

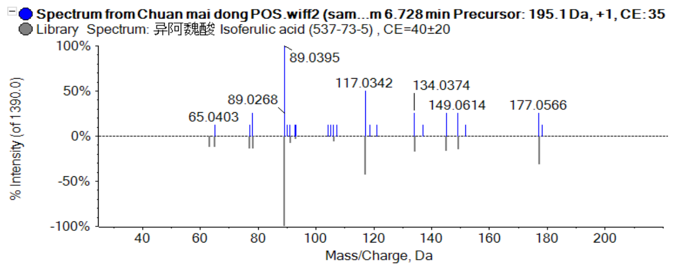

Acquired / Theoretical MS

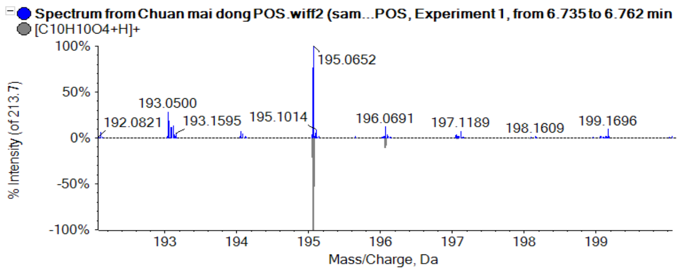

芸香柚皮苷Narirutin

Retention Time: 7.08 minutes

Exp RT: 7.10 minutes

Precursor m/z : 581.1865

Analyte Name: 芸香柚皮苷Narirutin

Fit (%) 93.9% **RFit (%)** 99.7%

Collision Energy = 35 ± 15 eV

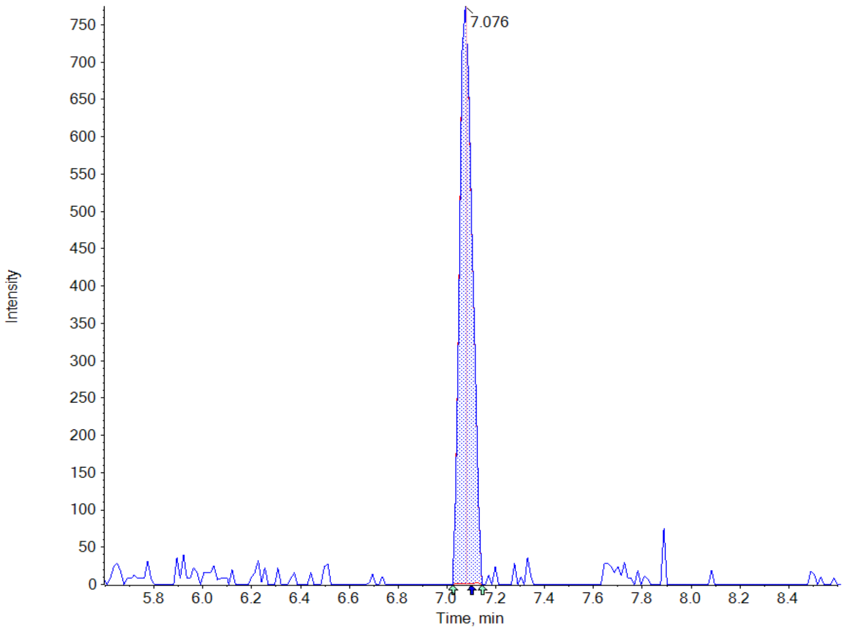

Acquired / Library MSMS

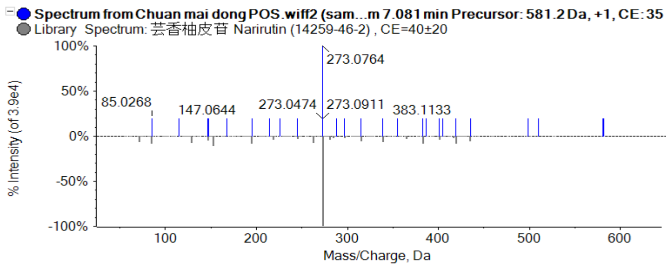

Acquired / Theoretical MS

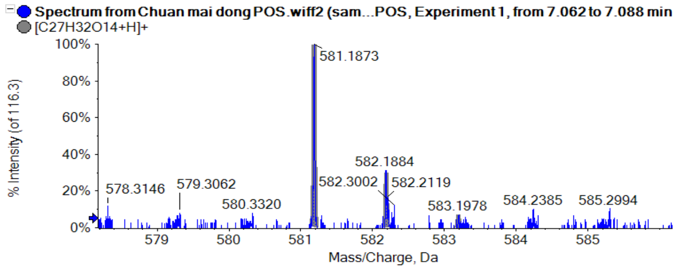

新橙皮苷Neohesperidin

Retention Time: 7.50 minutes
Precursor m/z : 611.1970
Fit (%) 96.7% **RFit (%)** 91.7%

Exp RT: 7.48 minutes
Analyte Name:
新橙皮苷Neohesperidin

Collision Energy = 35 ± 15 eV

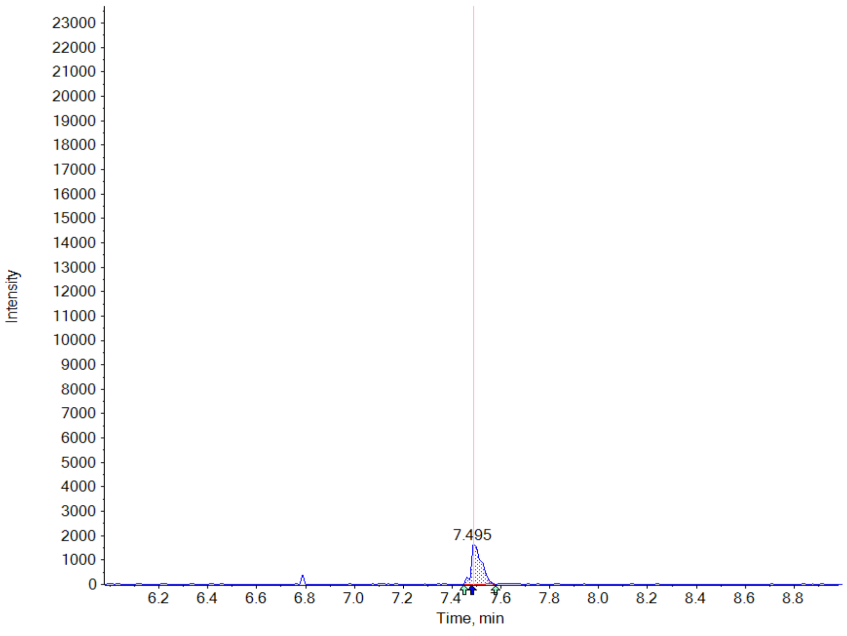

Acquired / Library MSMS

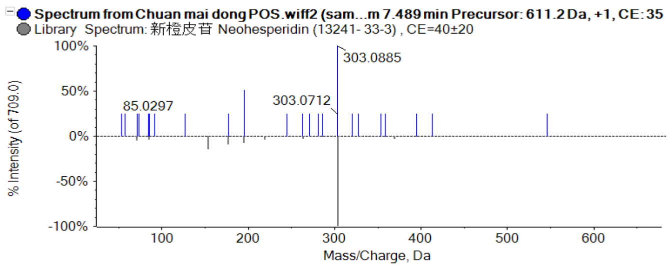

Acquired / Theoretical MS

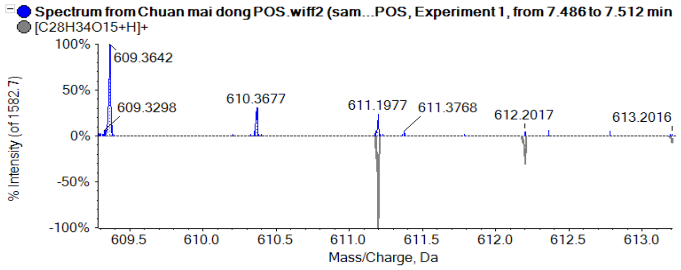

椴树苷Tiliroside

Retention Time: 8.91 minutes **Exp RT:** 8.90 minutes

Precursor m/z : 595.1446 **Analyte Name:**

Fit (%) 100.0% **RFit (%)** 99.6% 椴树苷Tiliroside

Collision Energy = 35 ± 15 eV

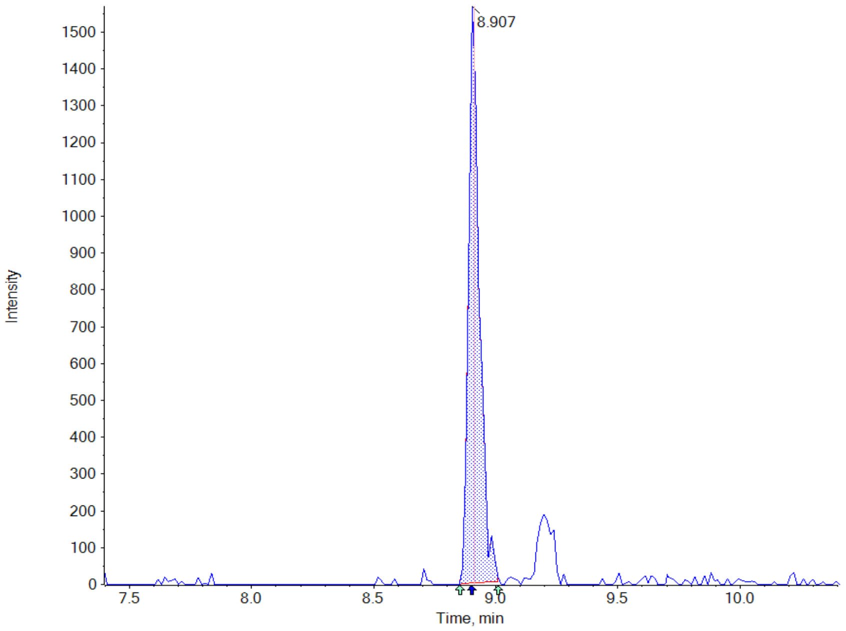

Acquired / Library MSMS

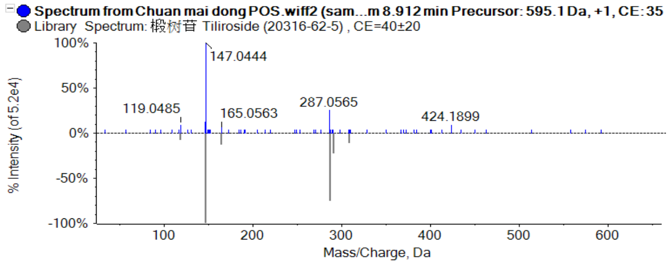

Acquired / Theoretical MS

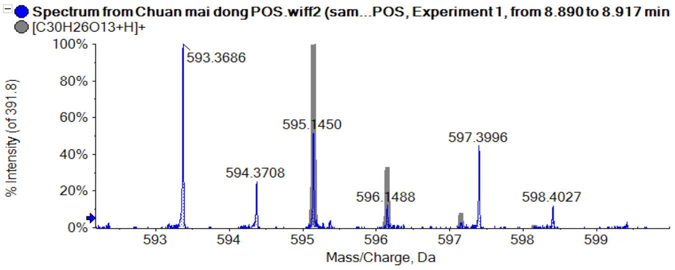

毛蕊异黄酮苷Calycosin-7-O-glucoside

Retention Time: 9.42 minutes
Precursor m/z : 447.1286
Fit (%) 98.2% **RFit (%)** 100.0%

Exp RT: 9.42 minutes
Analyte Name: 毛蕊异黄酮苷Calycosin-7-O-glucoside

Collision Energy = 35 ± 15 eV

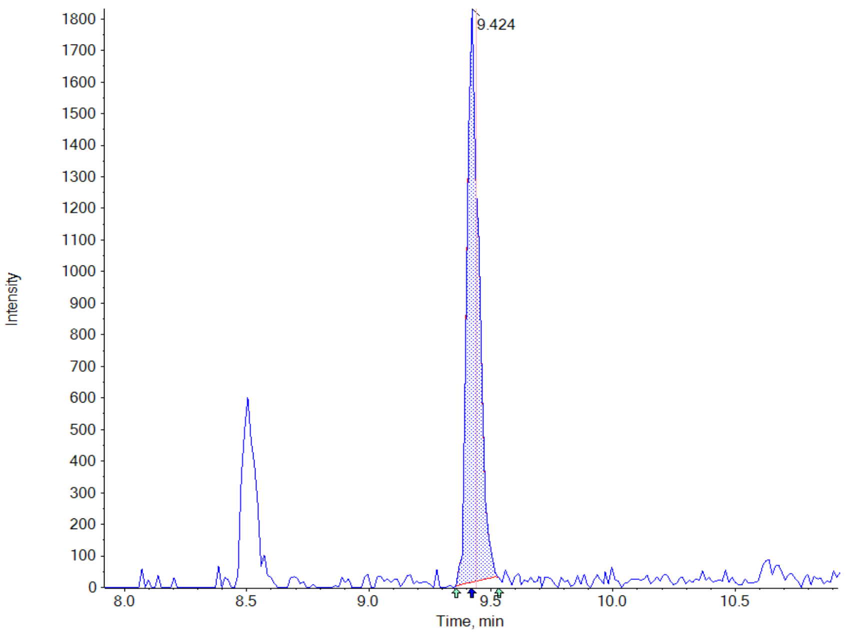

Acquired / Library MSMS

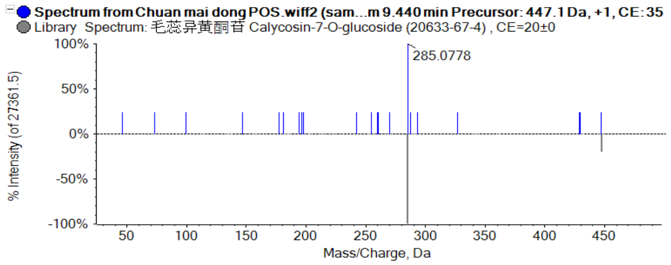

Acquired / Theoretical MS

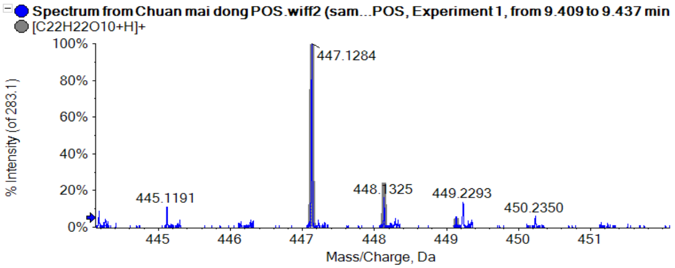

川陈皮素Nobiletin

Retention Time: 12.77 minutes
Precursor m/z : 403.1387
Fit (%) 88.7% **RFit (%)** 97.0%

Exp RT: 12.77 minutes
Analyte Name:
川陈皮素Nobiletin

Collision Energy = 35 ± 15 eV

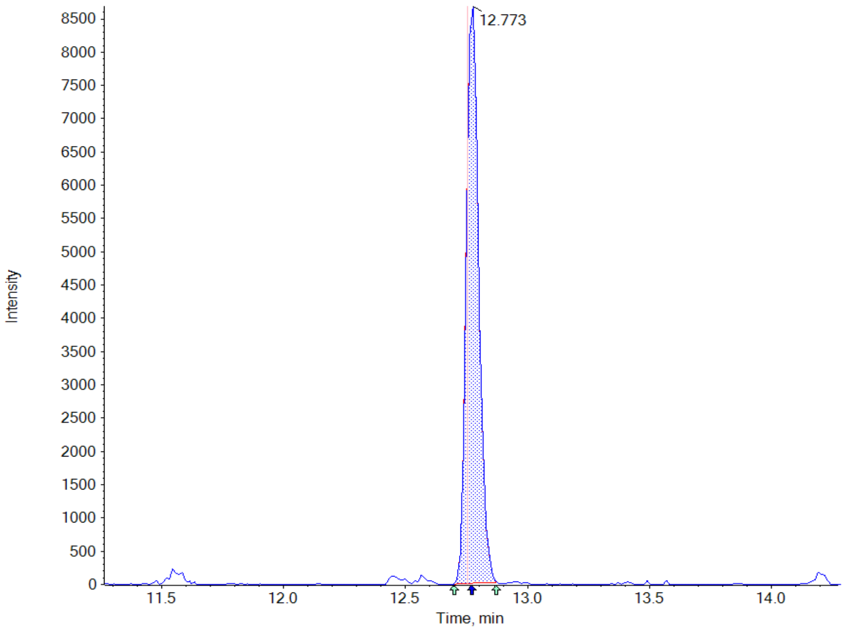

Acquired / Library MSMS

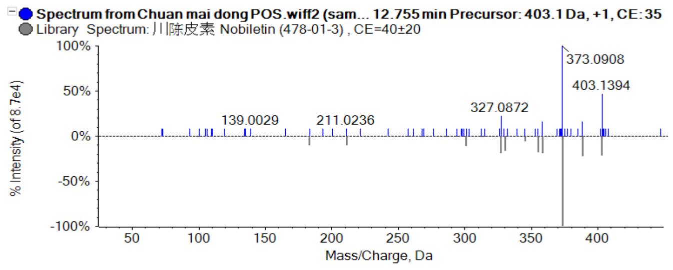

Acquired / Theoretical MS

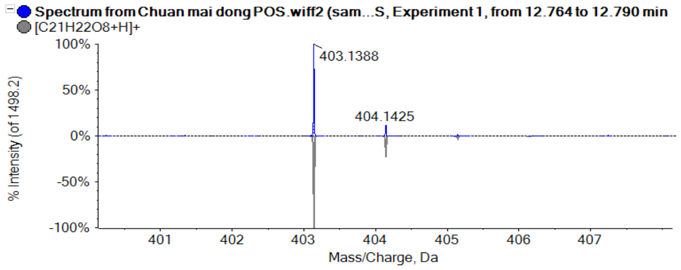

麦冬皂苷D' Ophiopogonin D'

Retention Time: 14.83 minutes
Precursor m/z : 855.4737
Fit (%) 94.1% **RFit (%)** 99.9%

Exp RT: 14.84 minutes
Analyte Name:
麦冬皂苷D' Ophiopogonin D'

Collision Energy = 35 ± 15 eV

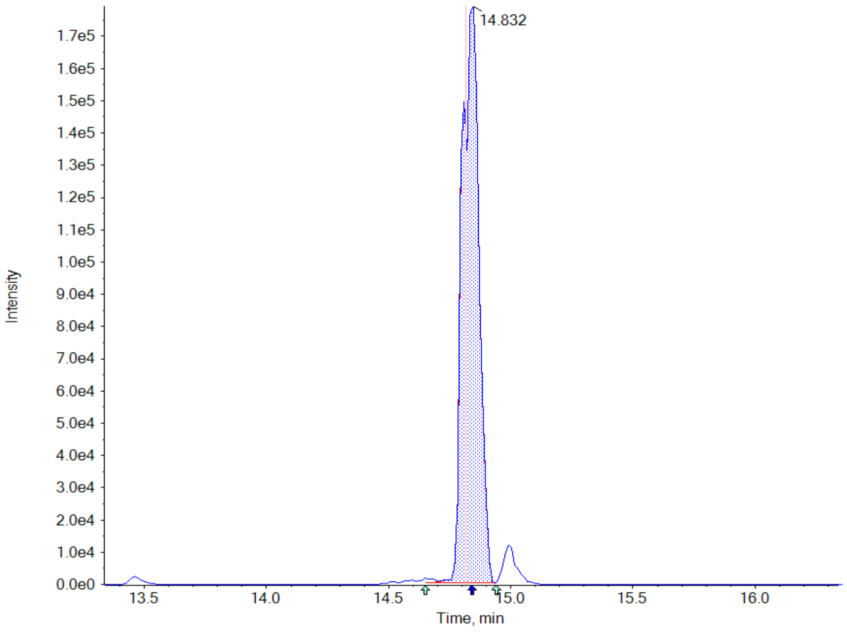

Acquired / Library MSMS

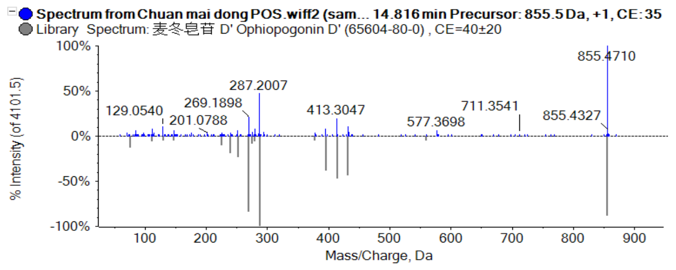

Acquired / Theoretical MS

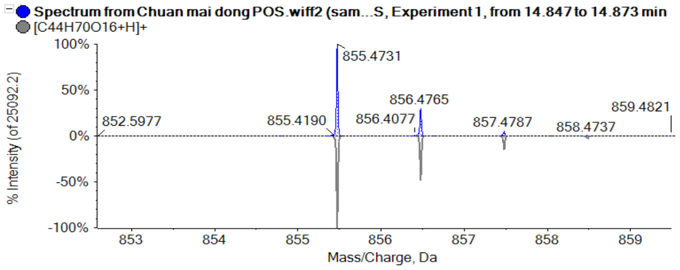

鲁斯可皂苷元Ruscogenin

Retention Time: 14.85 minutes **Exp RT:** 14.85 minutes

Precursor m/z : 431.3156 **Analyte Name:**

Fit (%) 99.9% **RFit (%)** 91.8% 鲁斯可皂苷元Ruscogenin

Collision Energy = 35 ± 15 eV

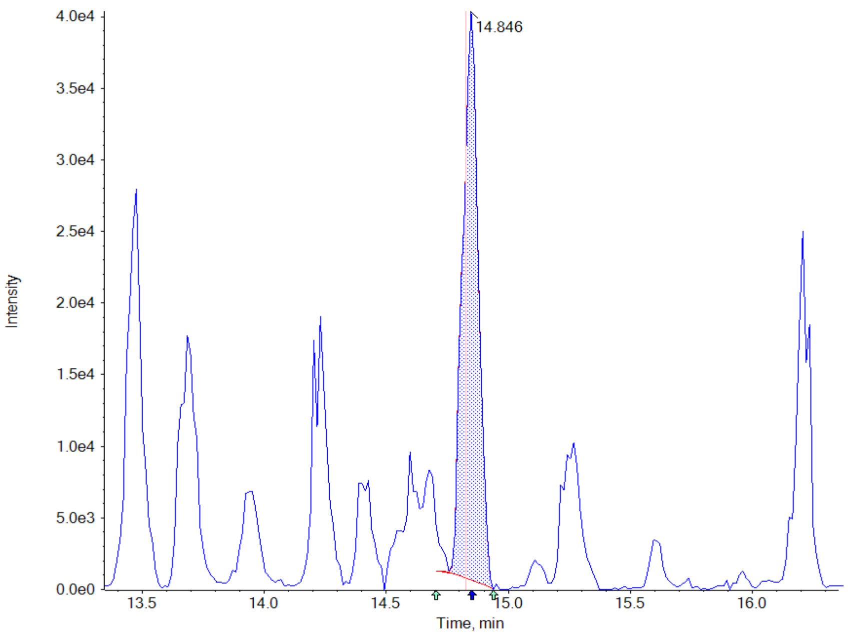

Acquired / Library MSMS

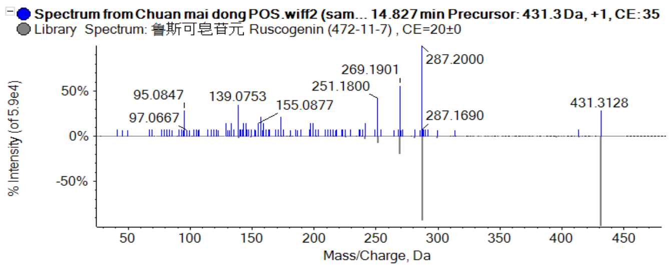

Acquired / Theoretical MS

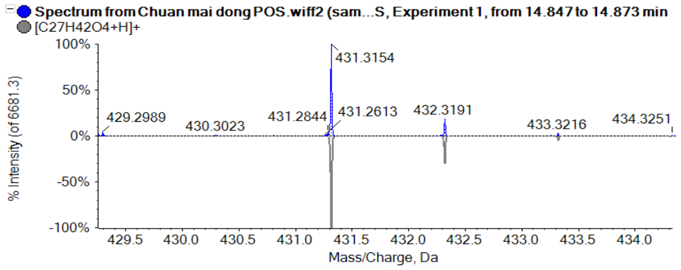

MS fragmentation pathways for different chemical constituents of CMD in negative ion mode

组氨酸Histidine

Retention Time: 1.09 minutes **Exp RT:** 1.09 minutes

Precursor m/z : 154.0622 **Analyte Name:**

Fit (%) 93.0% **RFit (%)** 89.5% 组氨酸Histidine

Collision Energy = 35 ± 15 eV

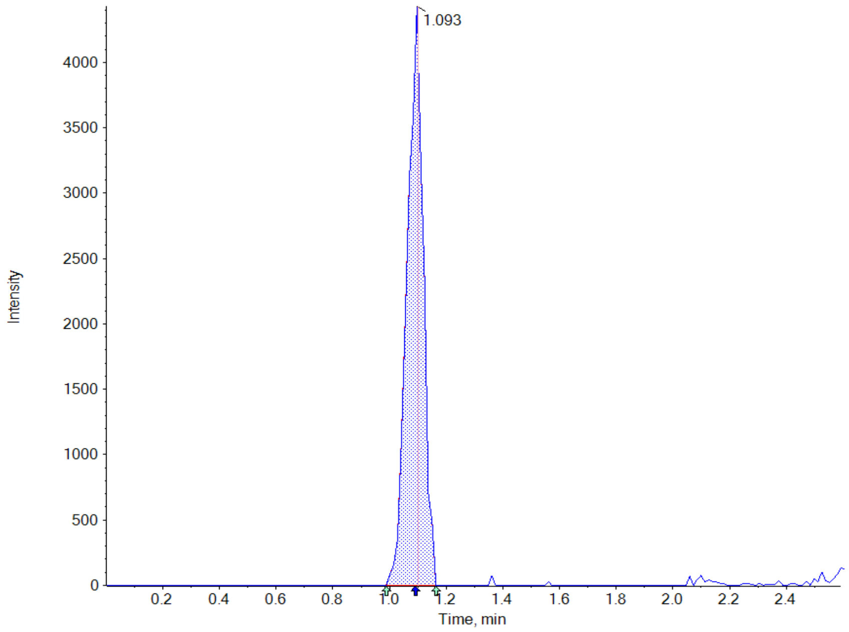

Acquired / Library MSMS

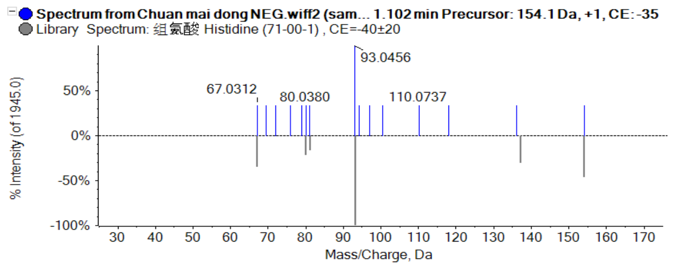

Acquired / Theoretical MS

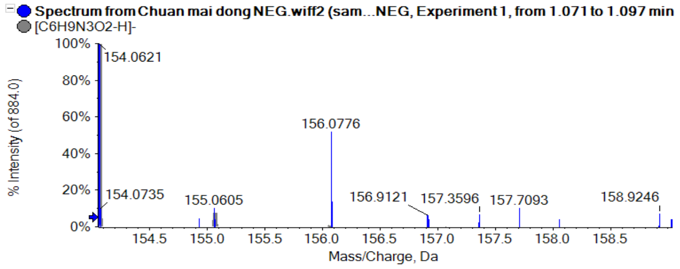

精氨酸L(+)-Arginine

**Retention Time:** 1.10 minutes	**Exp RT:** 1.09 minutes
**Precursor m/z :** 173.1044	**Analyte Name:**
**Fit (%)** 100.0% **RFit (%)** 94.9%	精氨酸L(+)-Arginine

Collision Energy = 35 ± 15 eV

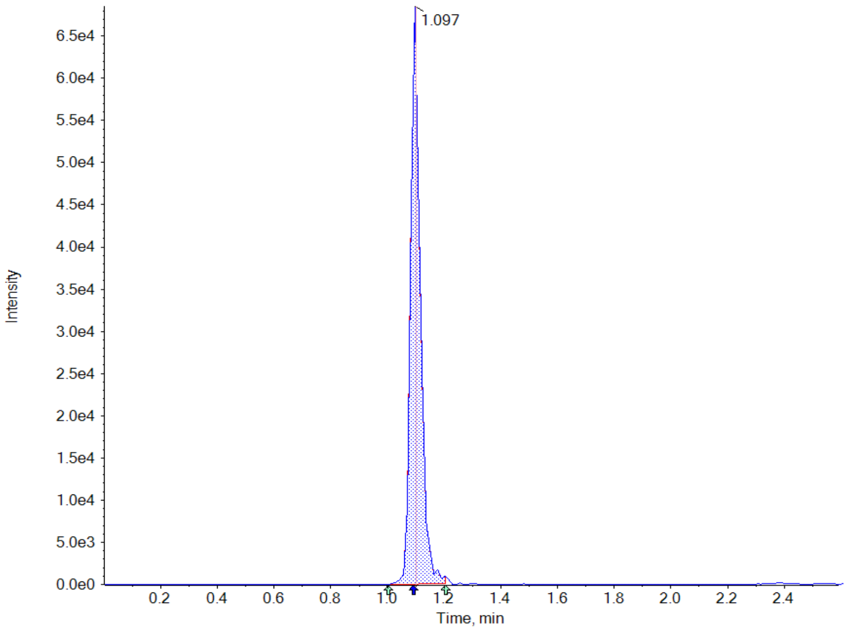

Acquired / Library MSMS

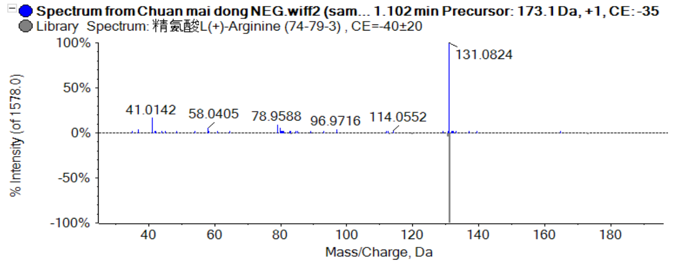

Acquired / Theoretical MS

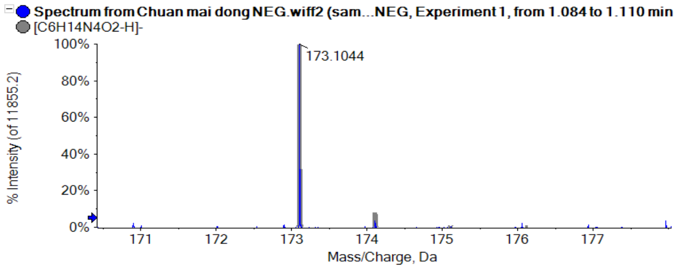

谷氨酸Glutamic acid

**Retention Time:** 1.14 minutes	**Exp RT:** 1.15 minutes
**Precursor m/z :** 146.0459	**Analyte Name:**
**Fit (%)** 98.4% **RFit (%)** 99.2%	谷氨酸Glutamic acid

Collision Energy = 35 ± 15 eV

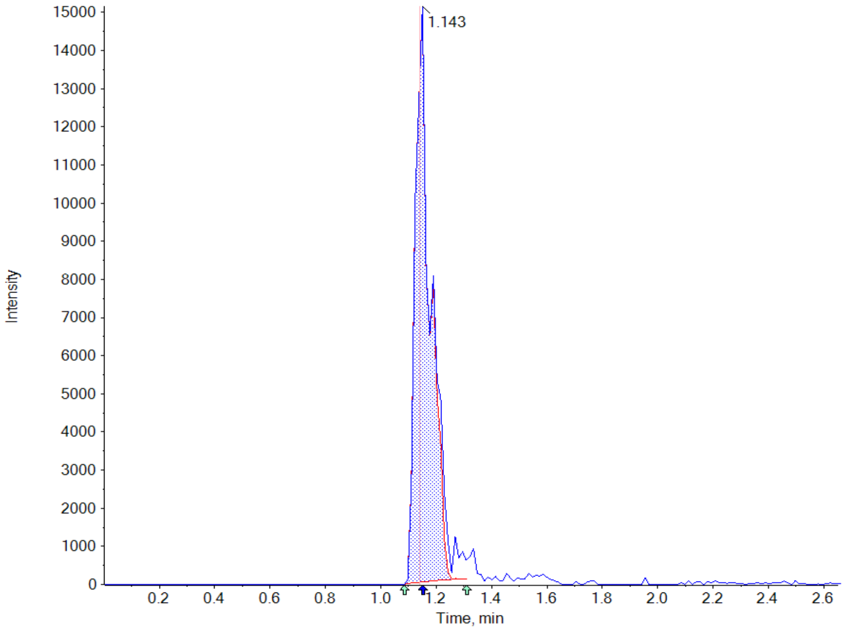

Acquired / Library MSMS

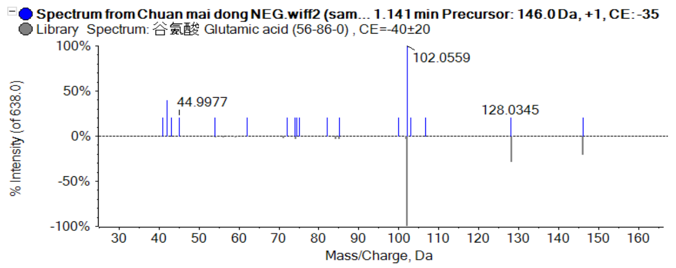

Acquired / Theoretical MS

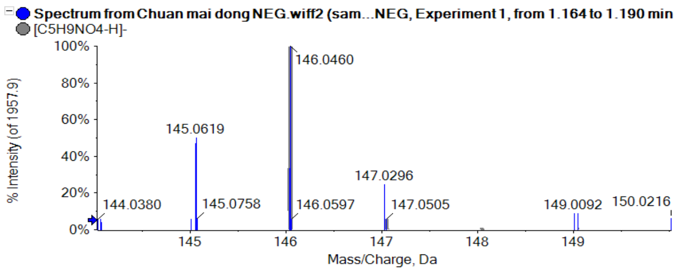

D-甘露糖D-(+)-Mannose

Retention Time: 1.22 minutes **Exp RT:** 1.21 minutes

Precursor m/z : 179.0561 **Analyte Name:**

Fit (%) 97.5% **RFit (%)** 97.1% D-甘露糖D-(+)-Mannose

Collision Energy = 35 ± 15 eV

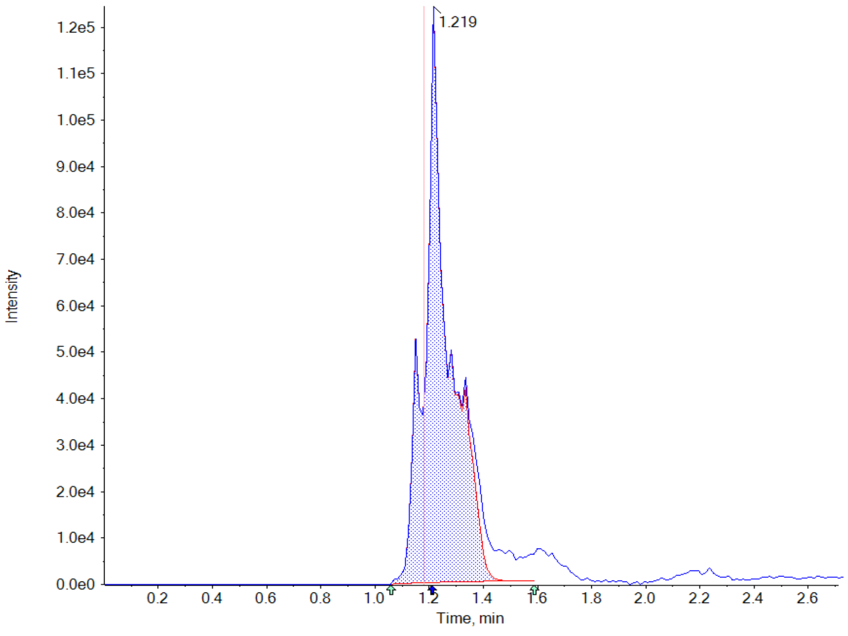

Acquired / Library MSMS

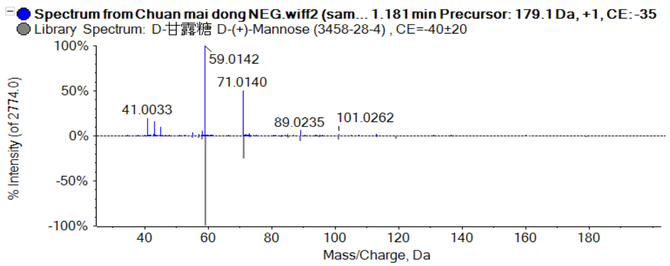

Acquired / Theoretical MS

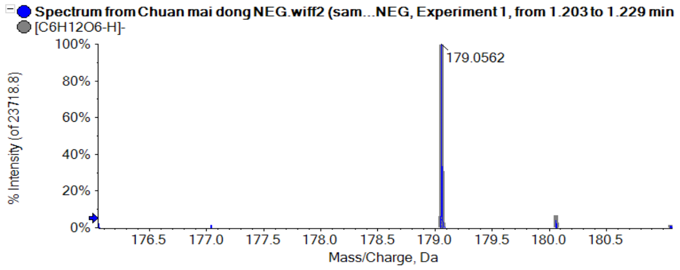

L-苹果酸L-Malic acid

Retention Time: 1.38 minutes
Precursor m/z : 133.0142
Fit (%) 96.3% **RFit (%)** 87.5%

Exp RT: 1.34 minutes
Analyte Name:
L-苹果酸L-Malic acid

Collision Energy = 35 ± 15 eV

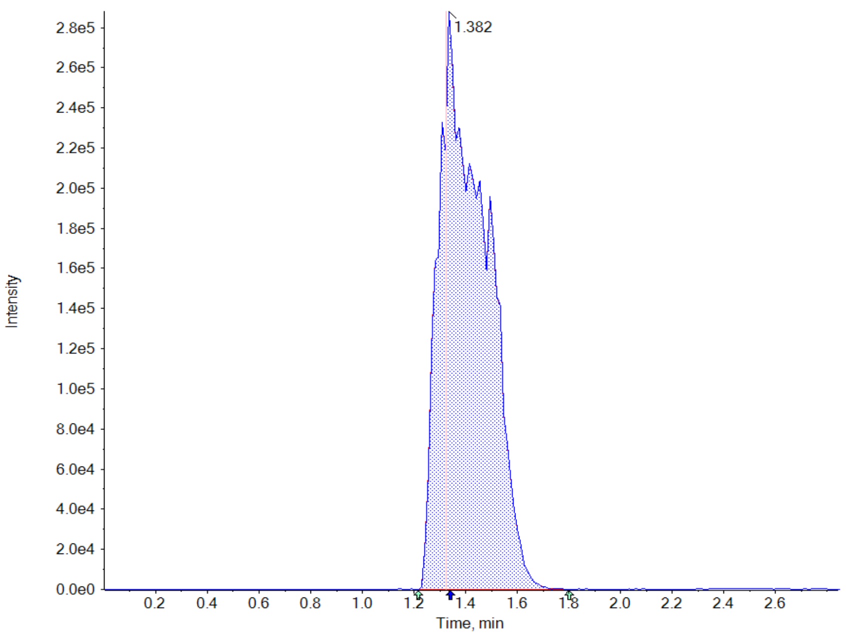

Acquired / Library MSMS

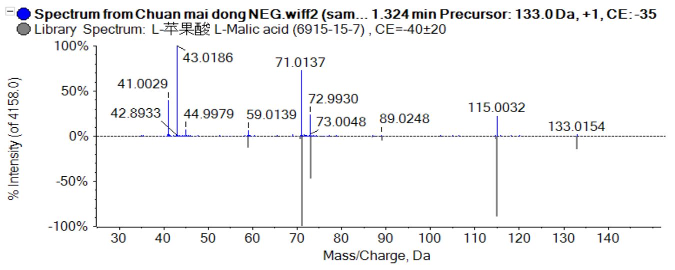

Acquired / Theoretical MS

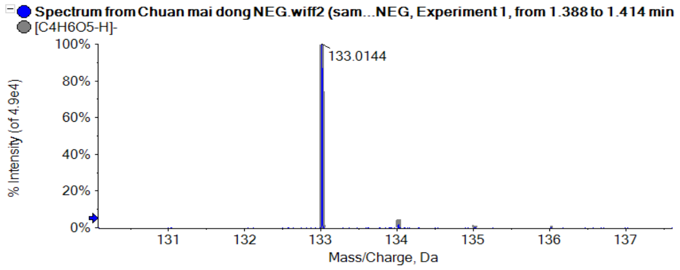

柠檬酸Citric acid

Retention Time: 1.90 minutes
Precursor m/z : 191.0197
Fit (%) 99.3% **RFit (%)** 99.6%

Exp RT: 1.94 minutes
Analyte Name:
柠檬酸Citric acid

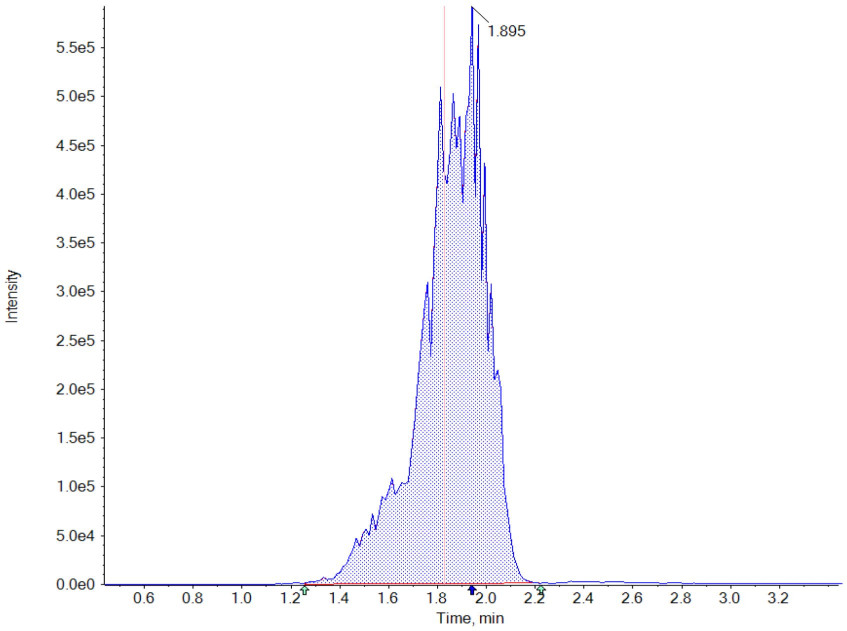

Collision Energy = 35 ± 15 eV

Acquired / Library MSMS

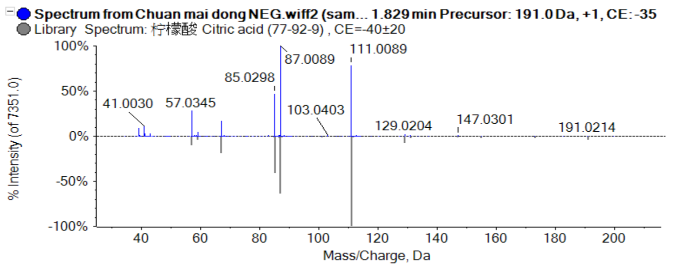

Acquired / Theoretical MS

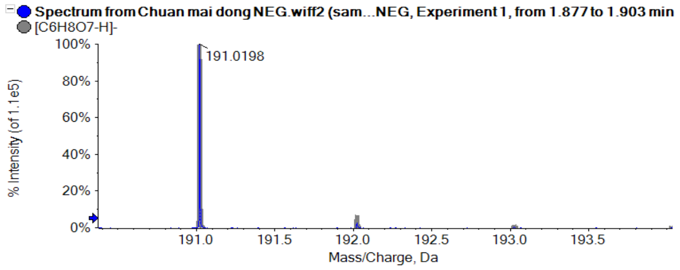

琥珀酸Succinic acid

Retention Time: 2.29 minutes
Precursor m/z : 117.0193
Fit (%) 99.1% **RFit (%)** 95.4%

Exp RT: 2.30 minutes
Analyte Name:
琥珀酸Succinic acid

Collision Energy = 35 ± 15 eV

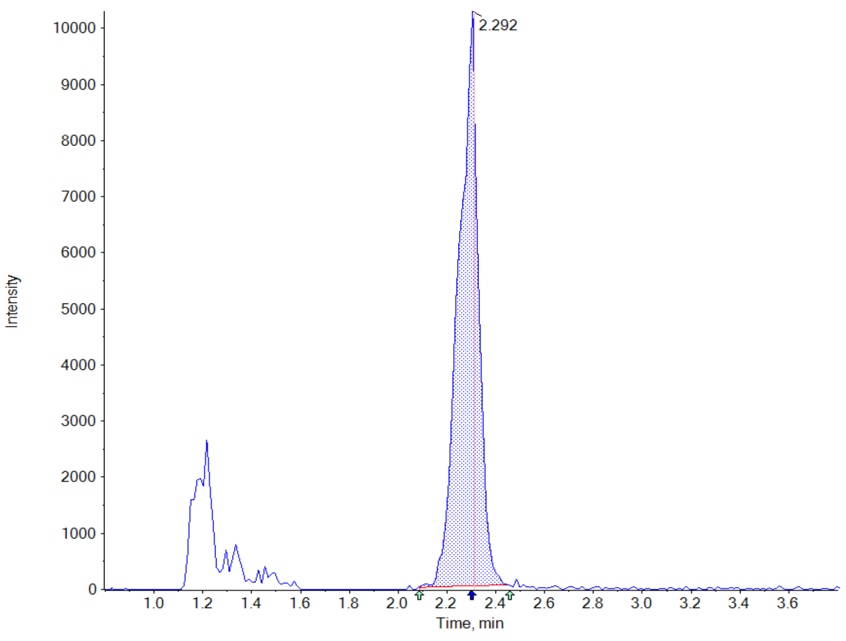

Acquired / Library MSMS

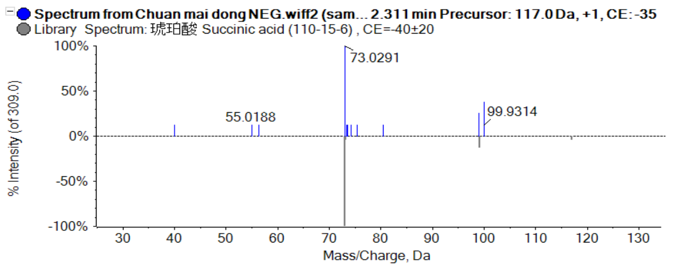

Acquired / Theoretical MS

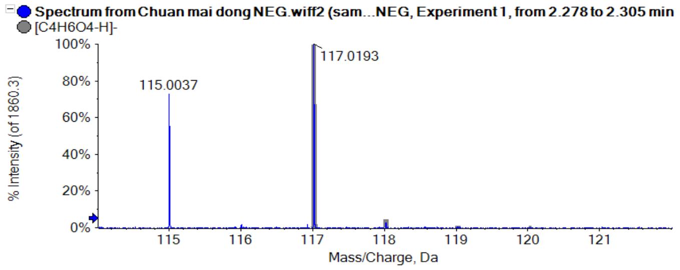

亮氨酸Leucine

Retention Time: 2.47 minutes **Exp RT:** 2.47 minutes
Precursor m/z : 130.0874 **Analyte Name:**
Fit (%) 100.0% **RFit (%)** 100.0% 亮氨酸Leucine

Collision Energy = 35 ± 15 eV

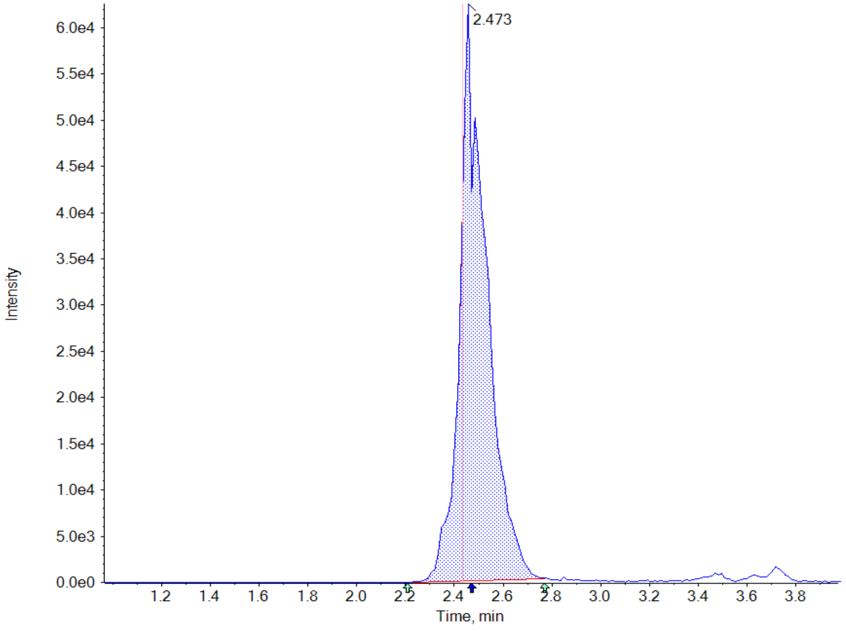

Acquired / Library MSMS

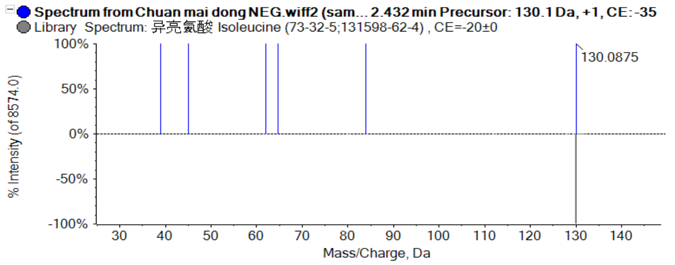

Acquired / Theoretical MS

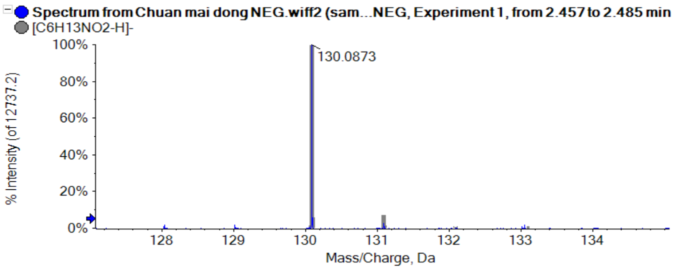

鸟苷Guanosine

**Retention Time:** 2.47 minutes	**Exp RT:** 2.48 minutes
**Precursor m/z :** 282.0844	**Analyte Name:**
**Fit (%)** 99.2% **RFit (%)** 100.0%	鸟苷Guanosine

Collision Energy = 35 ± 15 eV

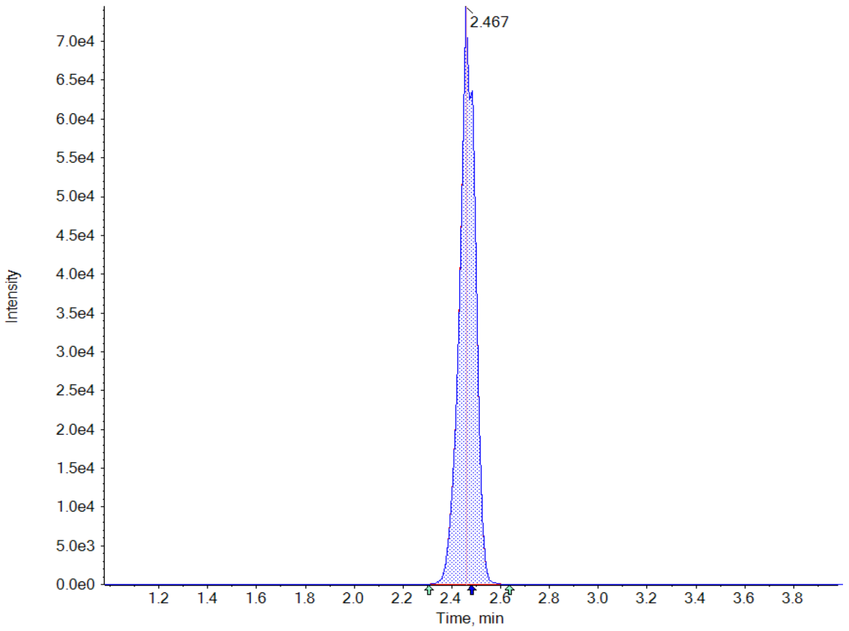

Acquired / Library MSMS

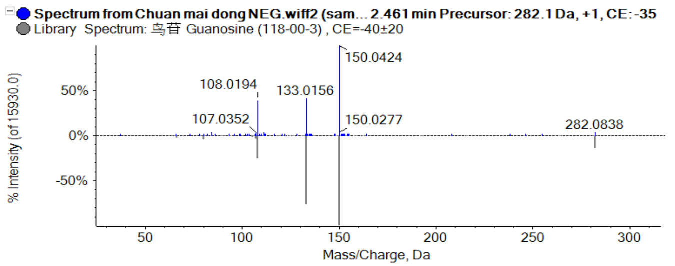

Acquired / Theoretical MS

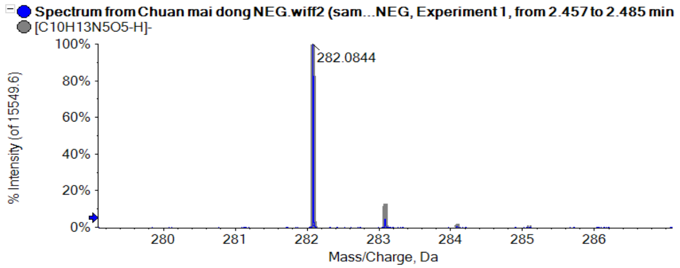

没食子酸Gallic acid

Retention Time: 2.65 minutes
Precursor m/z : 169.0142
Fit (%) 90.6% **RFit (%)** 100.0%

Exp RT: 2.66 minutes
Analyte Name:
没食子酸Gallic acid

Collision Energy = 35 ± 15 eV

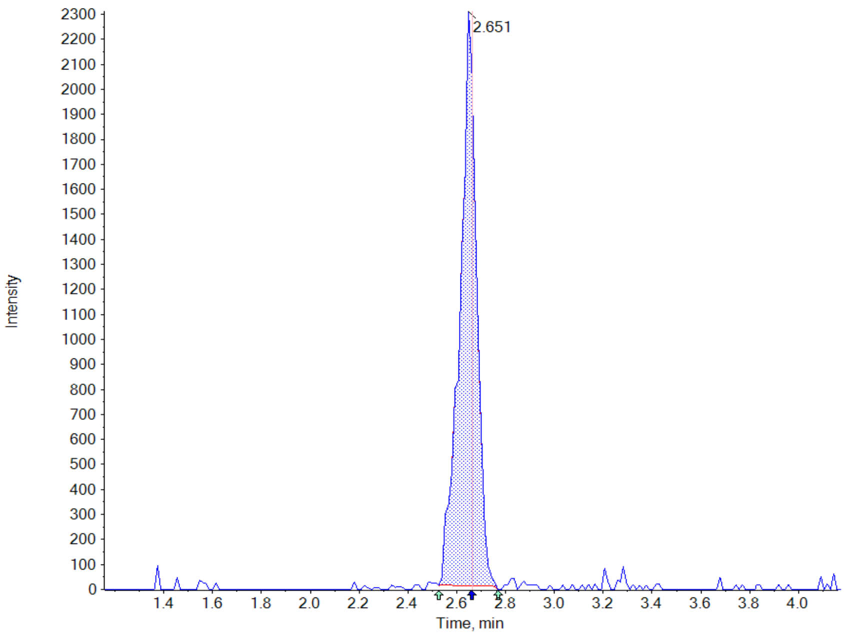

Acquired / Library MSMS

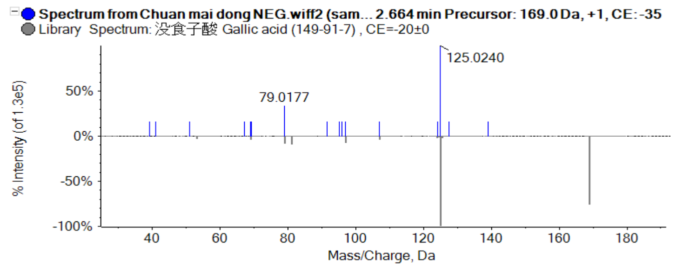

Acquired / Theoretical MS

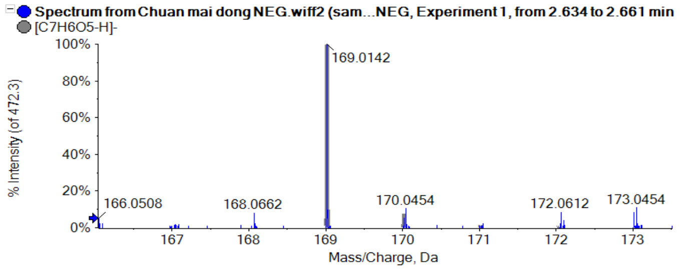

苯丙氨酸Phenprobamate

**Retention Time:** 3.18 minutes	**Exp RT:** 3.18 minutes
**Precursor m/z :** 164.0717	**Analyte Name:**
**Fit (%)** 97.9% **RFit (%)** 98.0%	苯丙氨酸Phenprobamate

Collision Energy = 35 ± 15 eV

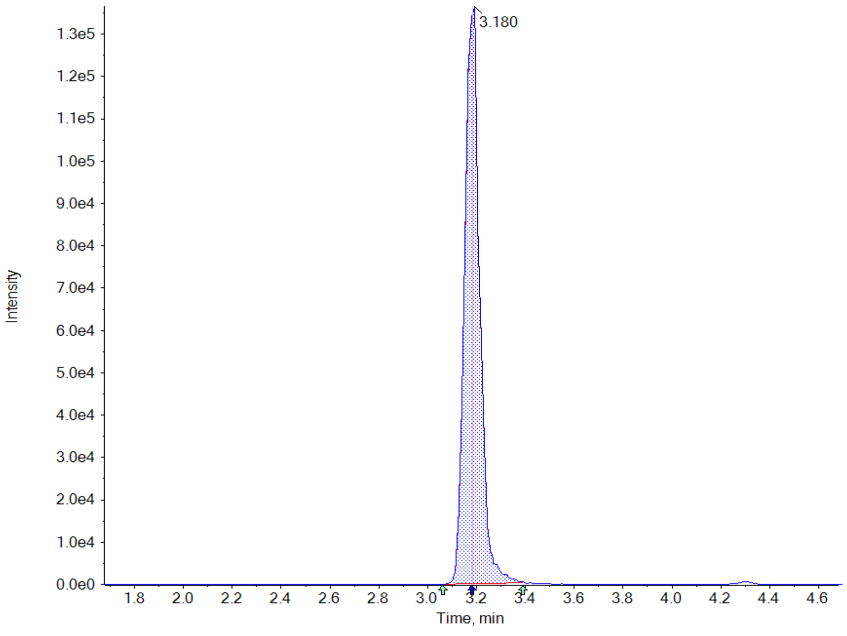

Acquired / Library MSMS

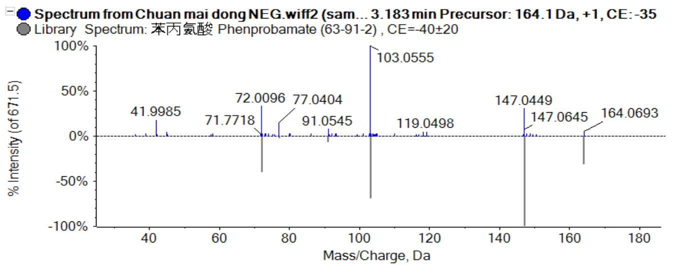

Acquired / Theoretical MS

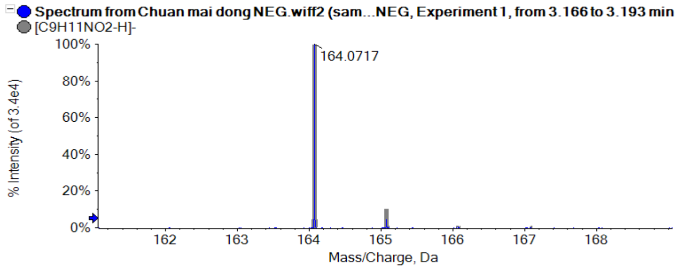

香草酸Vanillic acid

Retention Time: 3.37 minutes
Precursor m/z : 167.0350
Fit (%) 71.6% **RFit (%)** 100.0%

Exp RT: 3.37 minutes
Analyte Name:
香草酸Vanillic acid

Collision Energy = 35 ± 15 eV

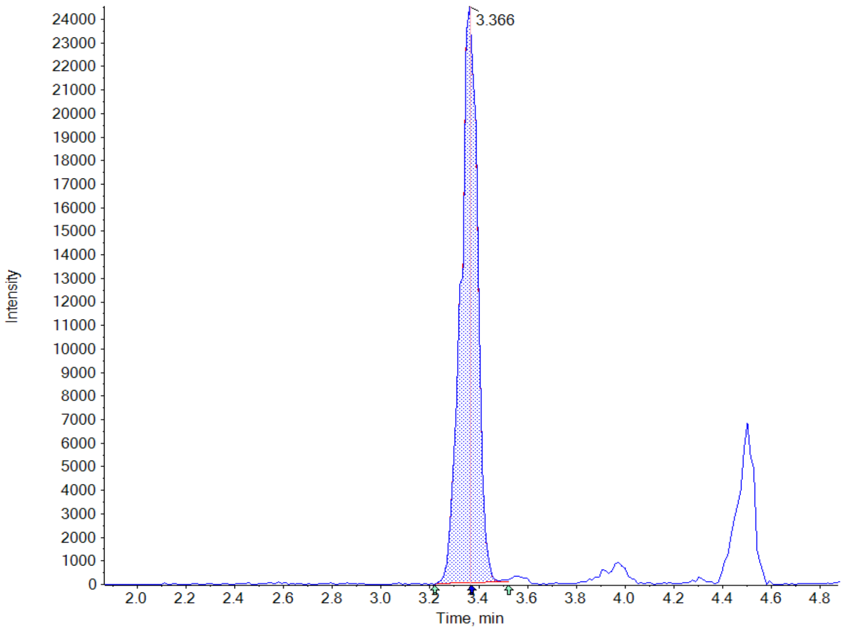

Acquired / Library MSMS

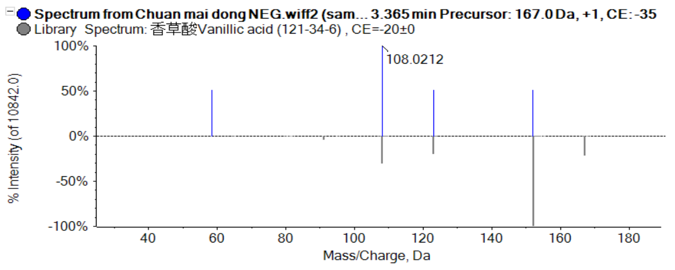

Acquired / Theoretical MS

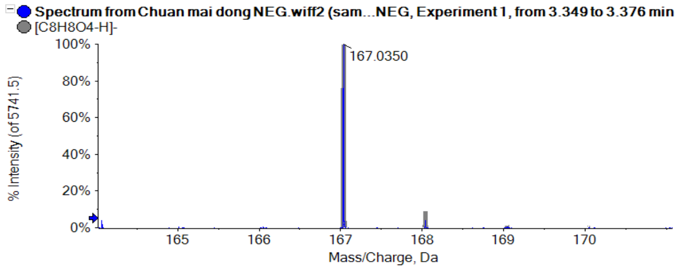

色氨酸L-Tryptophan

Retention Time: 4.16 minutes
Precursor m/z : 203.0826
Fit (%) 96.7% **RFit (%)** 100.0%

Exp RT: 4.16 minutes
Analyte Name:
色氨酸L-Tryptophan

Collision Energy = 35 ± 15 eV

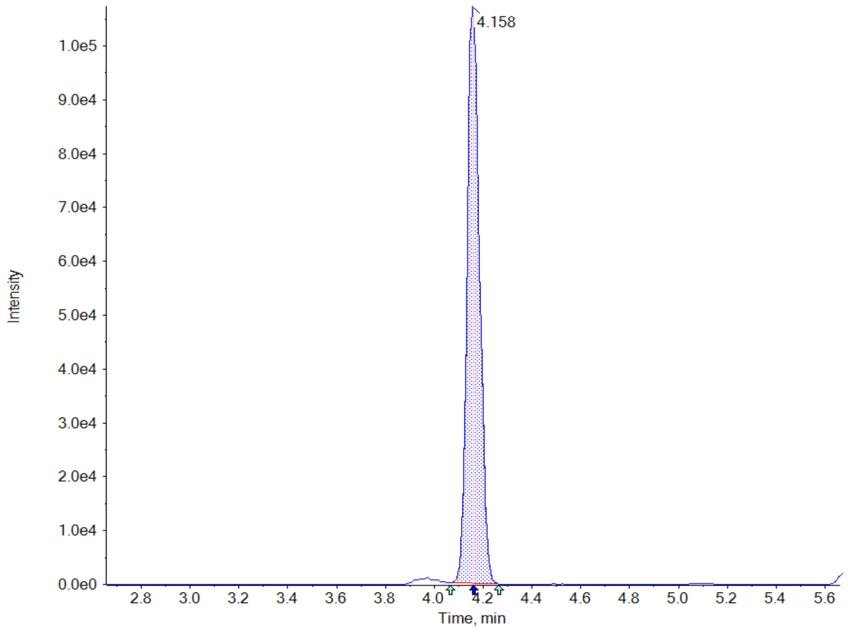

Acquired / Library MSMS

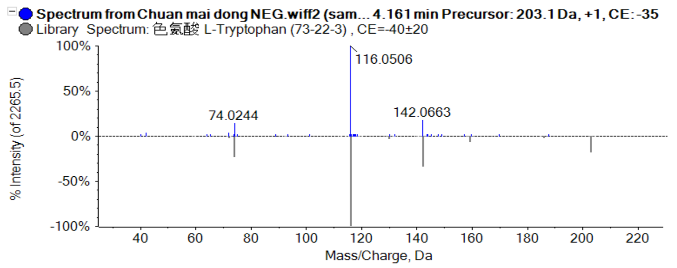

Acquired / Theoretical MS

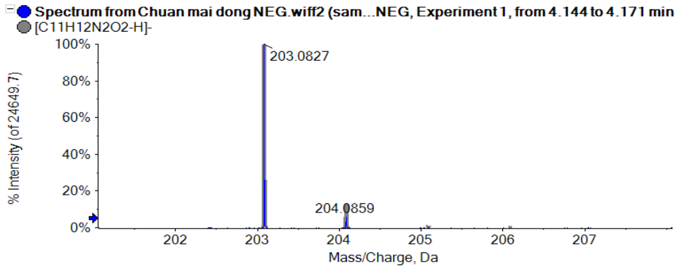

对羟基苯甲酸4-Hydroxybenzoic acid

Retention Time: 4.63 minutes **Exp RT:** 4.63 minutes

Precursor m/z : 137.0244 **Analyte Name:**

Fit (%) 100.0% **RFit (%)** 100.0% 对羟基苯甲酸4-Hydroxybenzoic acid

Collision Energy = 35 ± 15 eV

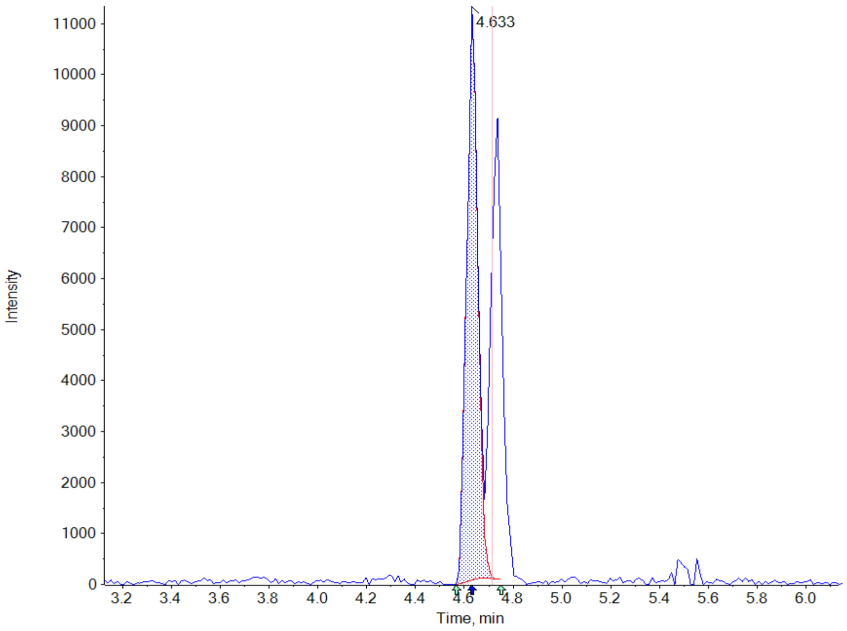

Acquired / Library MSMS

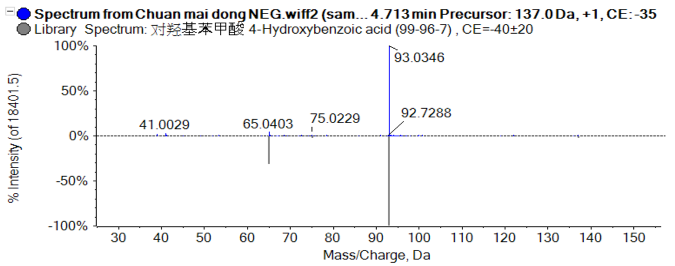

Acquired / Theoretical MS

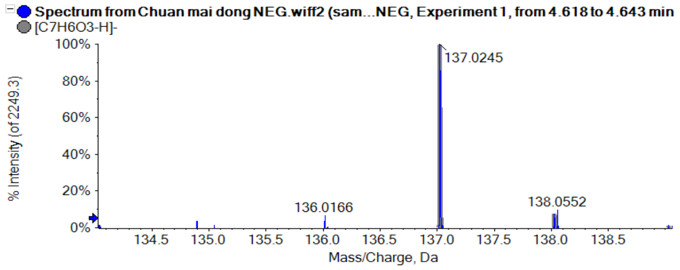

隐绿原酸(5-咖啡酰奎宁酸) 4-O-Caffeoyl Quinic acid

Retention Time: 4.80 minutes
Precursor m/z : 353.0878
Fit (%) 98.3% **RFit (%)** 99.3%

Exp RT: 4.80 minutes
Analyte Name:
隐绿原酸(5-咖啡酰奎宁酸) 4-O-Caffeoyl Quinic acid

Collision Energy = 35 ± 15 eV

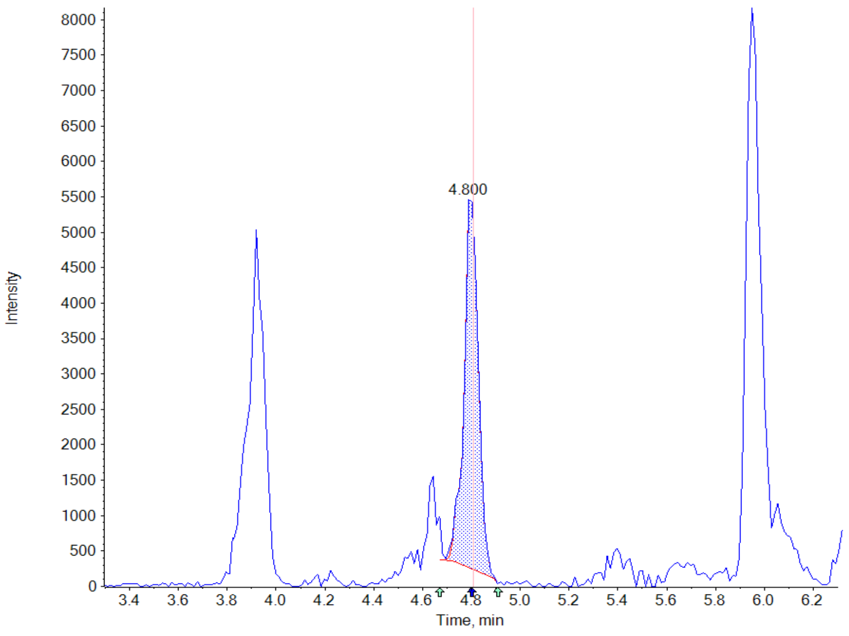

Acquired / Library MSMS

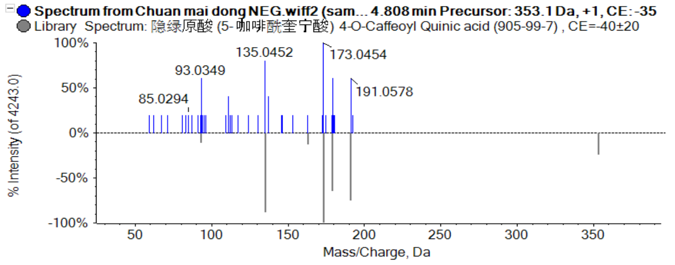

Acquired / Theoretical MS

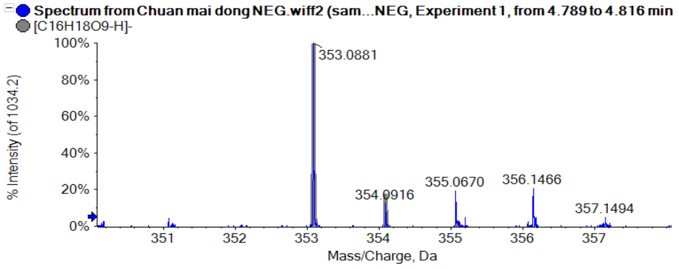

秦皮乙素Esculetin

Retention Time: 5.13 minutes
Precursor m/z : 177.0193
Fit (%) 96.9% **RFit (%)** 99.4%

Exp RT: 5.14 minutes
Analyte Name:
秦皮乙素Esculetin

Collision Energy = 35 ± 15 eV

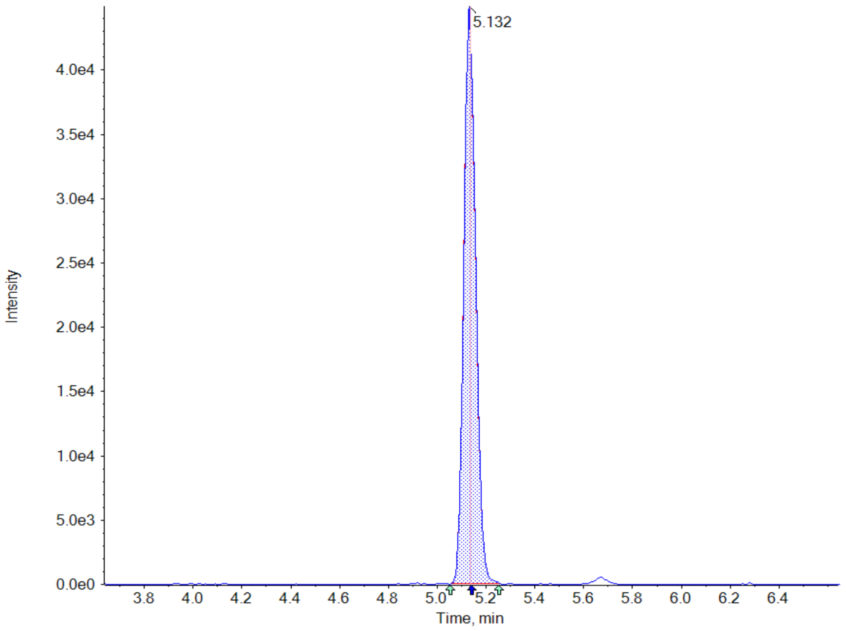

Acquired / Library MSMS

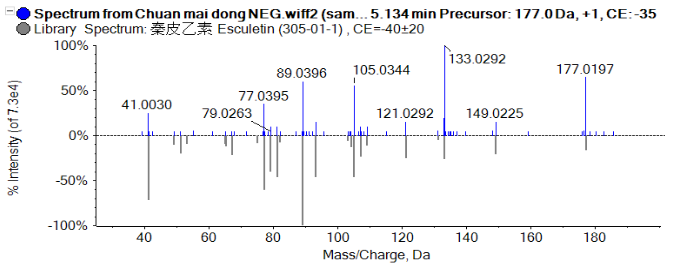

Acquired / Theoretical MS

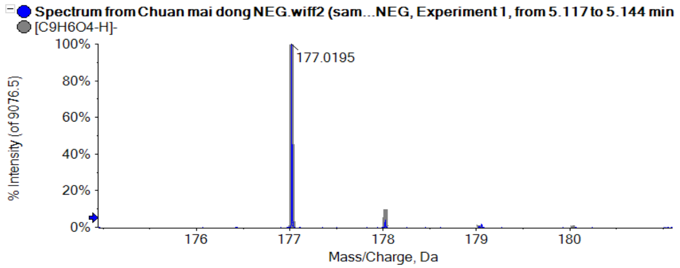

咖啡酸Caffeic acid

Retention Time: 5.21 minutes
Precursor m/z : 179.0350
Fit (%) 82.6% **RFit (%)** 100.0%

Exp RT: 5.21 minutes
Analyte Name:
咖啡酸Caffeic acid

Collision Energy = 35 ± 15 eV

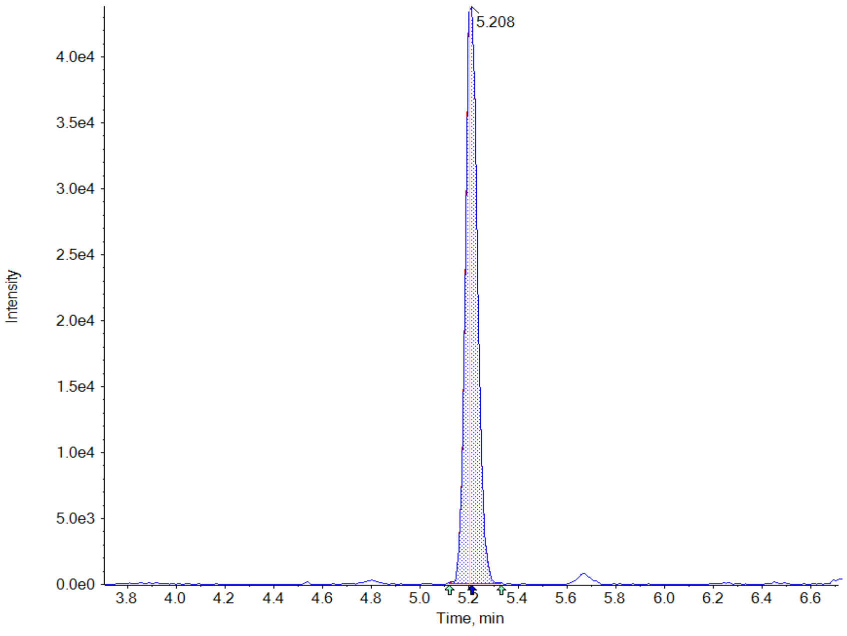

Acquired / Library MSMS

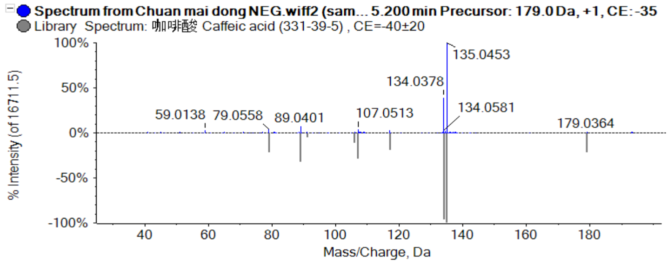

Acquired / Theoretical MS

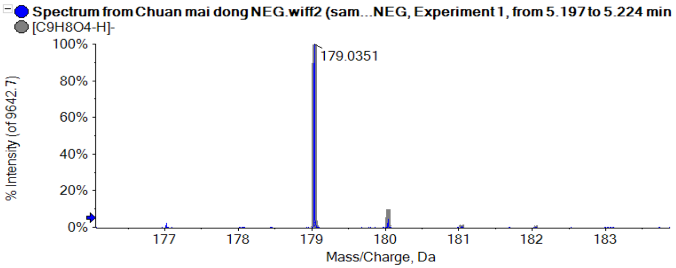

莽草酸Shikimic acid

Retention Time: 5.49 minutes
Precursor m/z : 173.0455
Fit (%) 71.1% **RFit (%)** 100.0%

Exp RT: 5.49 minutes
Analyte Name:
莽草酸Shikimic acid

Collision Energy = 35 ± 15 eV

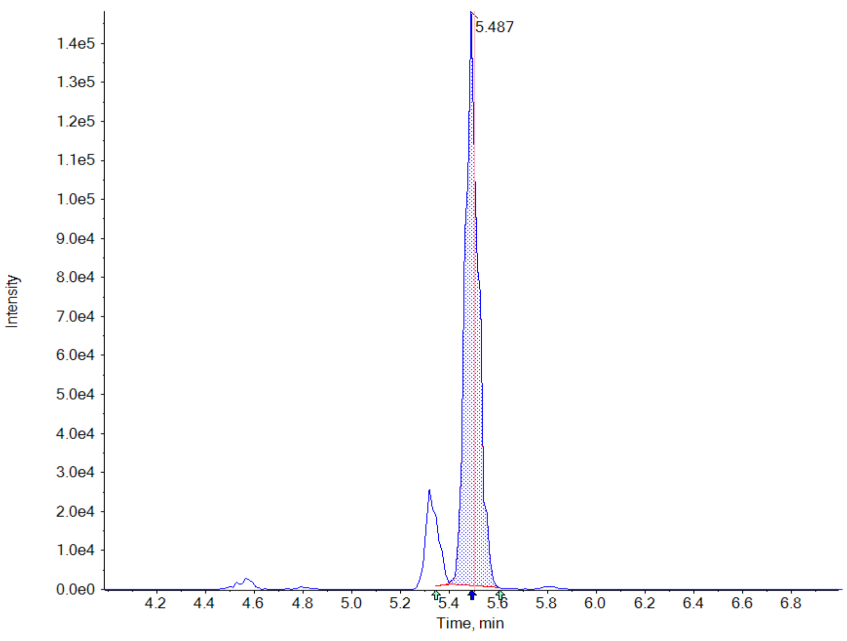

Acquired / Library MSMS

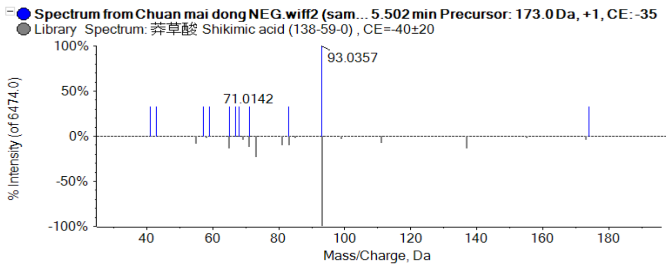

Acquired / Theoretical MS

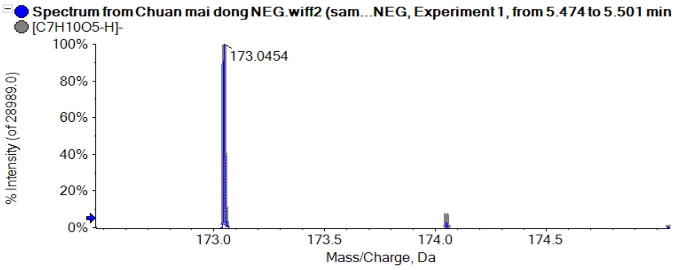

鞣花酸Ellagic Acid

Retention Time: 6.35 minutes
Precursor m/z : 300.9990
Fit (%) 100.0% **RFit (%)** 87.8%

Exp RT: 6.35 minutes
Analyte Name:
鞣花酸Ellagic Acid

Collision Energy = 35 ± 15 eV

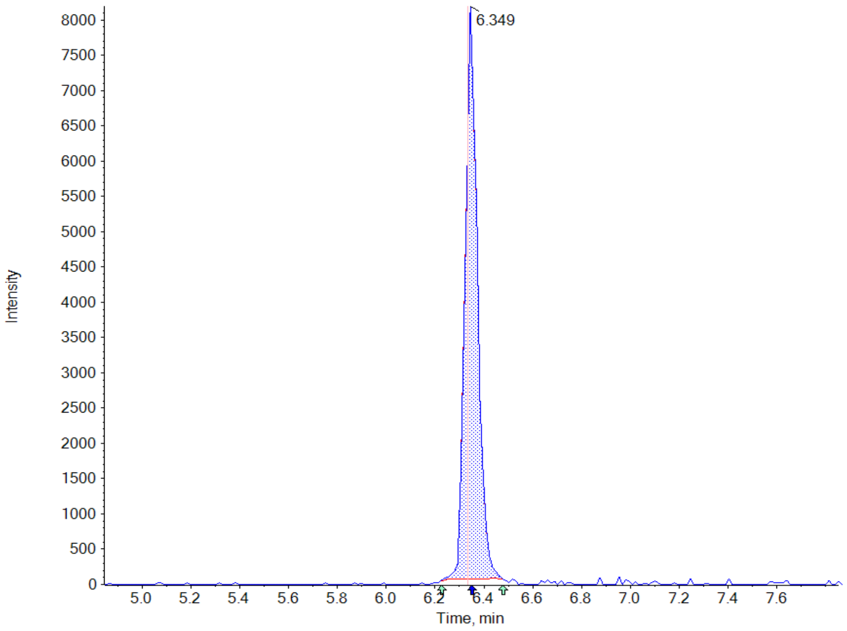

Acquired / Library MSMS

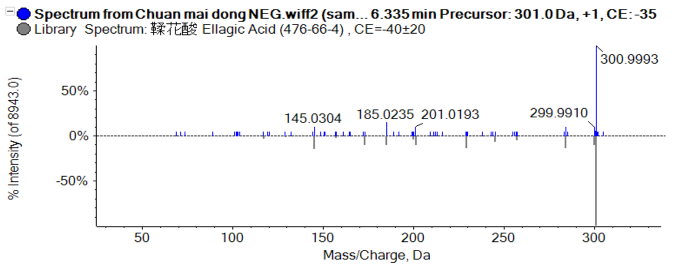

Acquired / Theoretical MS

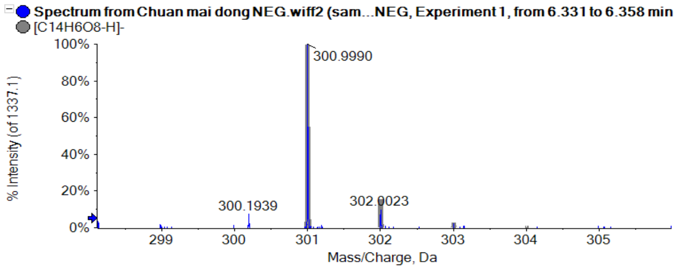

金丝桃苷Hyperin

Retention Time: 6.51 minutes
Precursor m/z : 463.0882
Fit (%) 99.4% **RFit (%)** 99.9%

Exp RT: 6.50 minutes
Analyte Name:
金丝桃苷Hyperin

Collision Energy = 35 ± 15 eV

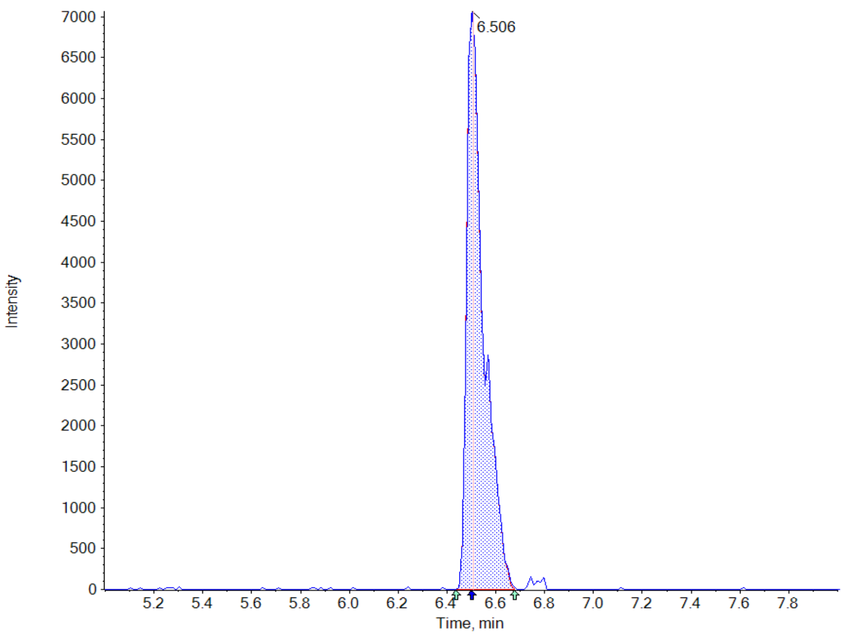

Acquired / Library MSMS

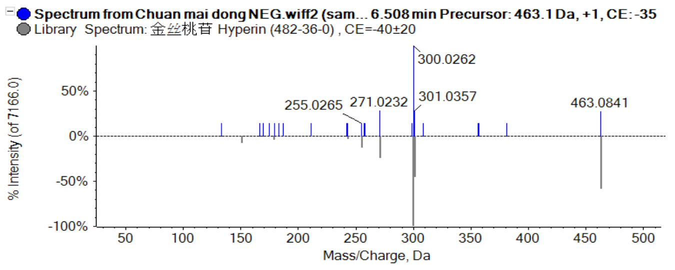

Acquired / Theoretical MS

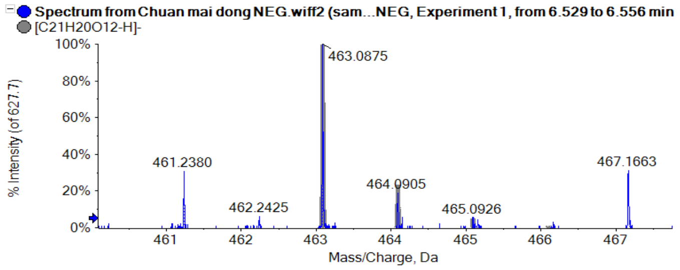

芸香柚皮苷Narirutin

Retention Time: 7.08 minutes
Precursor m/z : 579.1719
Fit (%) 93.2% **RFit (%)** 99.9%

Exp RT: 7.08 minutes
Analyte Name:
芸香柚皮苷Narirutin

Collision Energy = 35 ± 15 eV

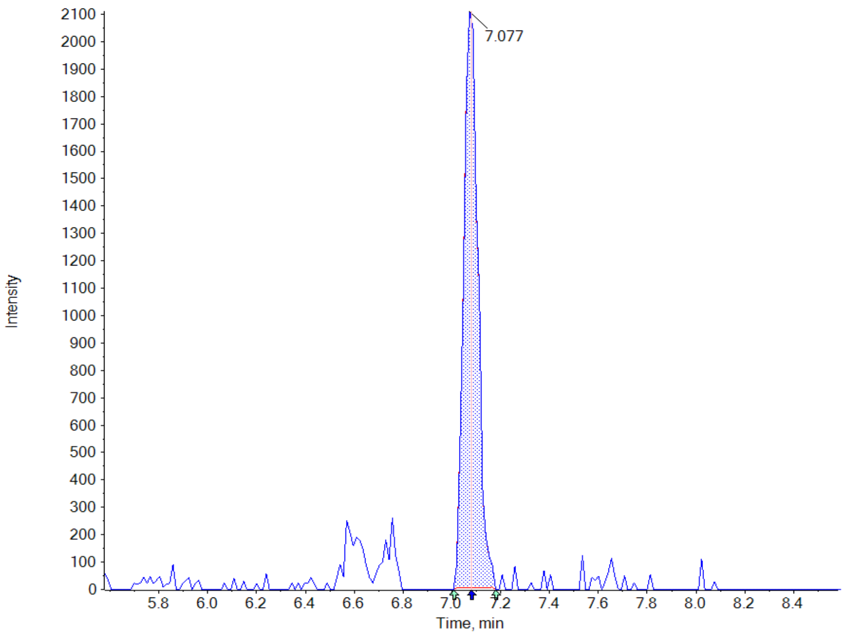

Acquired / Library MSMS

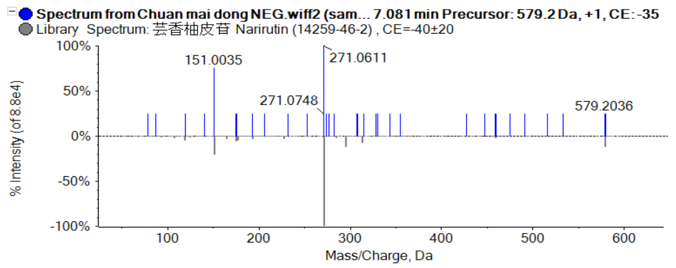

Acquired / Theoretical MS

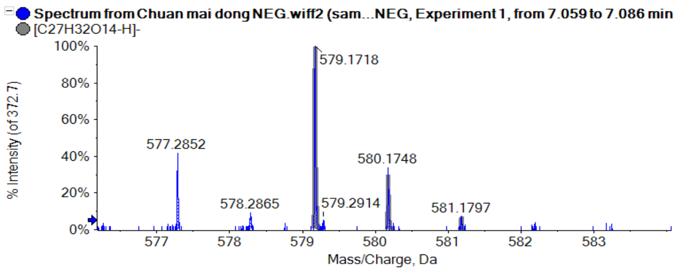

新橙皮苷Neohesperidin

**Retention Time:** 7.50 minutes	**Exp RT:** 7.49 minutes
**Precursor m/z :** 609.1825	**Analyte Name:**
**Fit (%)** 97.7% **RFit (%)** 97.3%	新橙皮苷Neohesperidin

Collision Energy = 35 ± 15 eV

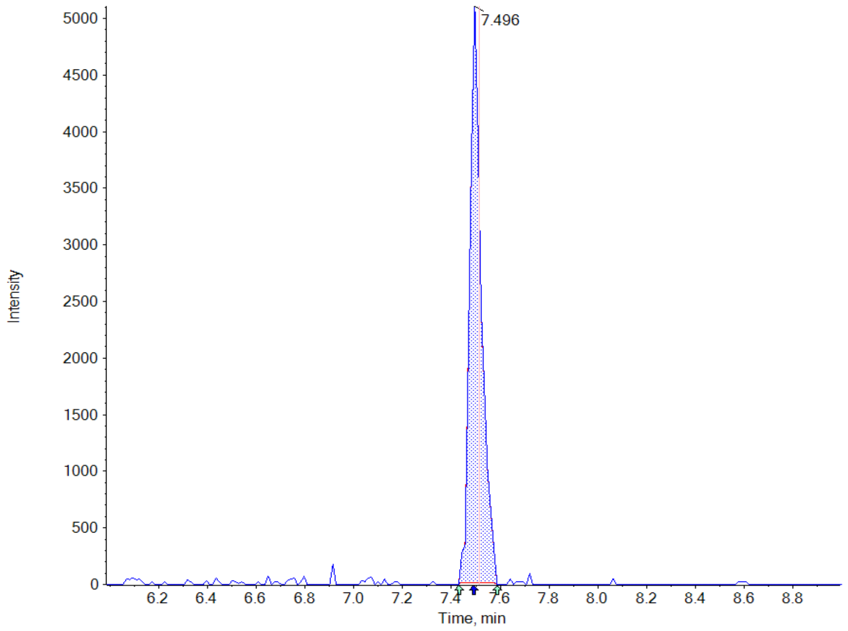

Acquired / Library MSMS

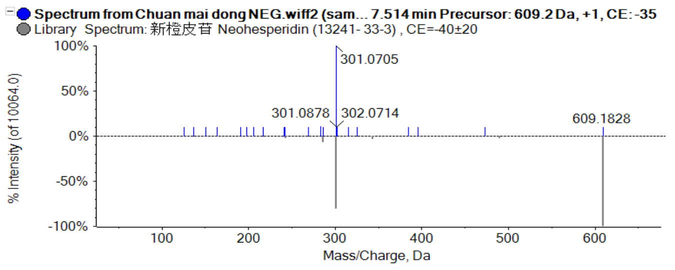

Acquired / Theoretical MS

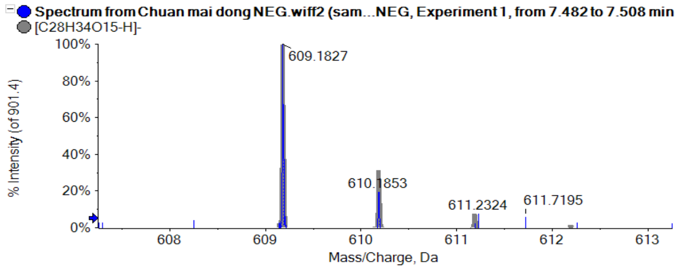

椴树苷Tiliroside

Retention Time: 8.92 minutes
Precursor m/z : 593.1301
Fit (%) 90.1% **RFit (%)** 100.0%

Exp RT: 8.93 minutes
Analyte Name:
椴树苷Tiliroside

Collision Energy = 35 ± 15 eV

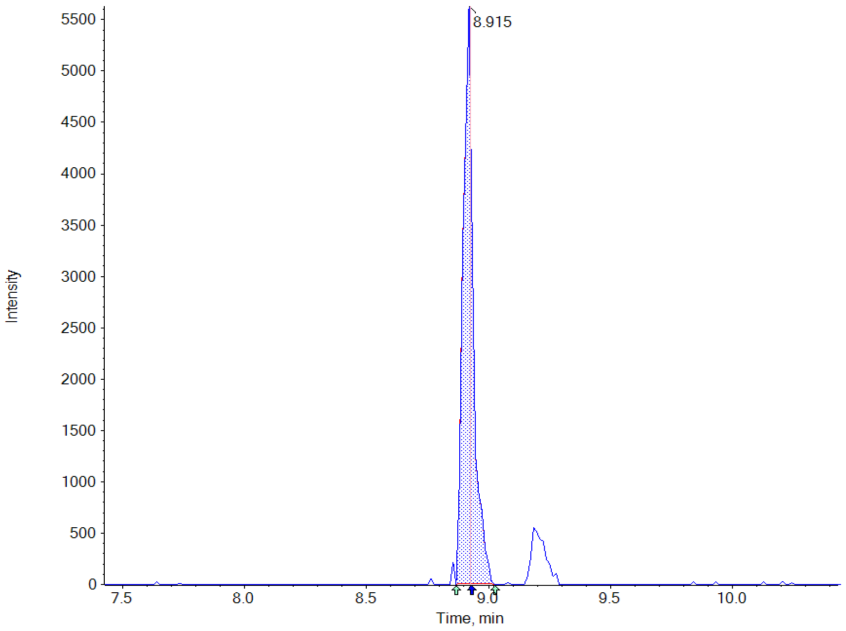

Acquired / Library MSMS

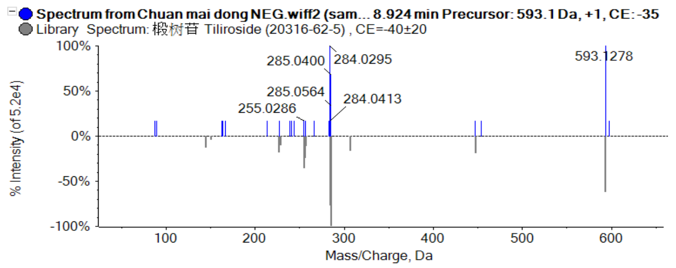

Acquired / Theoretical MS

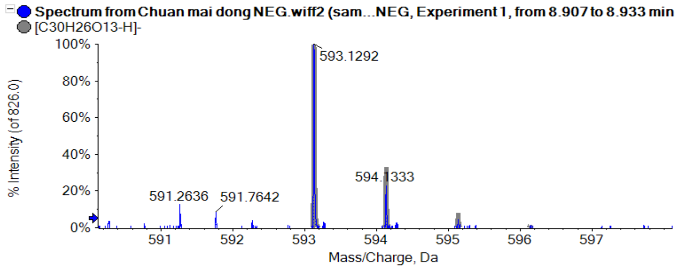

毛蕊异黄酮苷Calycosin-7-o-glucoside

Retention Time: 9.43 minutes
Precursor m/z : 491.1195
Fit (%) 100.0% **RFit (%)** 95.2%

Exp RT: 9.42 minutes
Analyte Name:
毛蕊异黄酮苷Calycosin-7-o-glucoside
+HCOOH

Collision Energy = 35 ± 15 eV

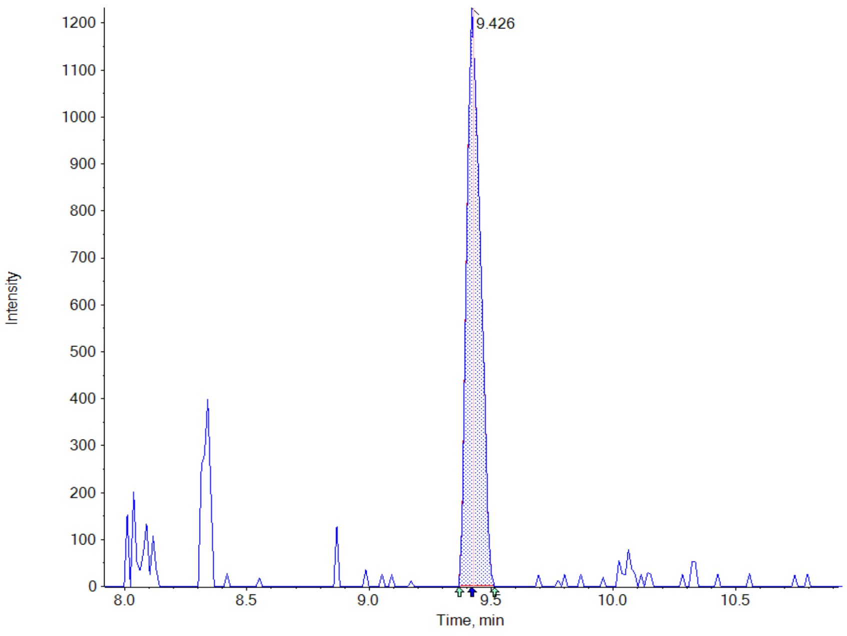

Acquired / Library MSMS

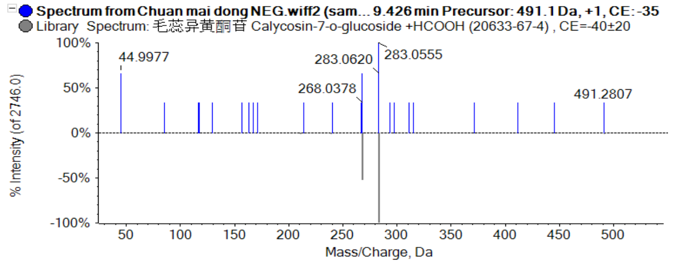

Acquired / Theoretical MS

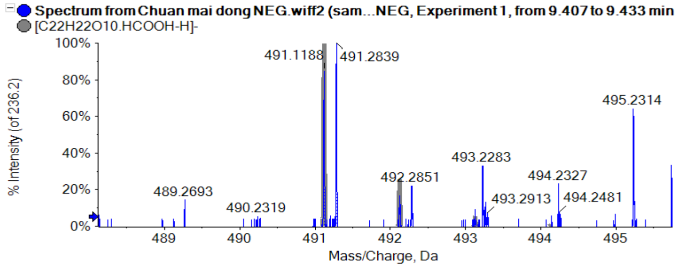

纤细薯蓣皂苷Gracillin

Retention Time: 10.78 minutes **Exp RT:** 10.77 minutes

Precursor m/z : 929.4752 **Analyte Name:**

Fit (%) 95.7% **RFit (%)** 96.9% 纤细薯蓣皂苷Gracillin +HCOOH

Collision Energy = 35 ± 15 eV

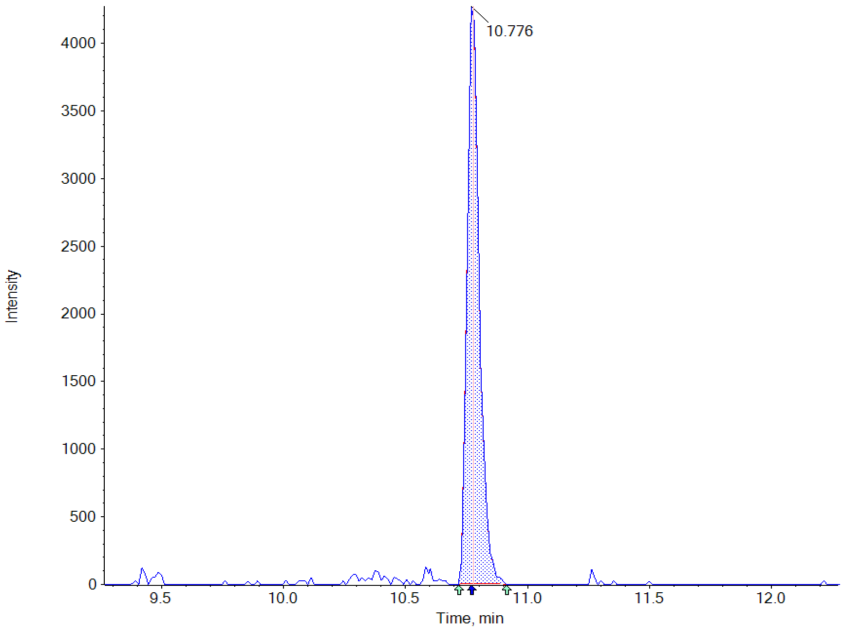

Acquired / Library MSMS

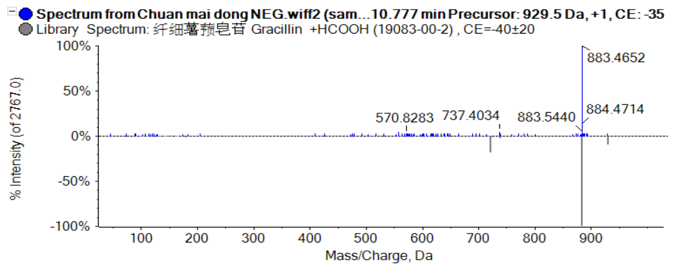

Acquired / Theoretical MS

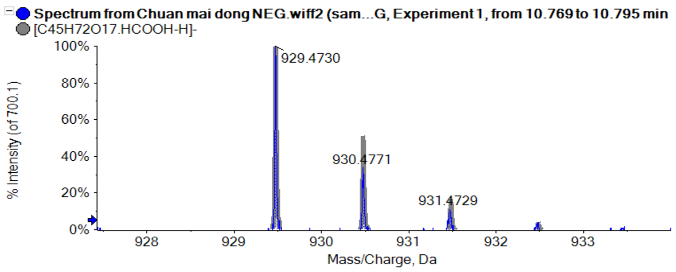

短葶山麦冬皂苷C Liriope muscari baily saponins C

Retention Time: 13.46 minutes
Precursor m/z : 869.4540
Fit (%) 99.4% **RFit (%)** 98.4%

Exp RT: 13.50 minutes
Analyte Name:
短葶山麦冬皂苷C Liriope muscari baily saponins C

Collision Energy = 35 ± 15 eV

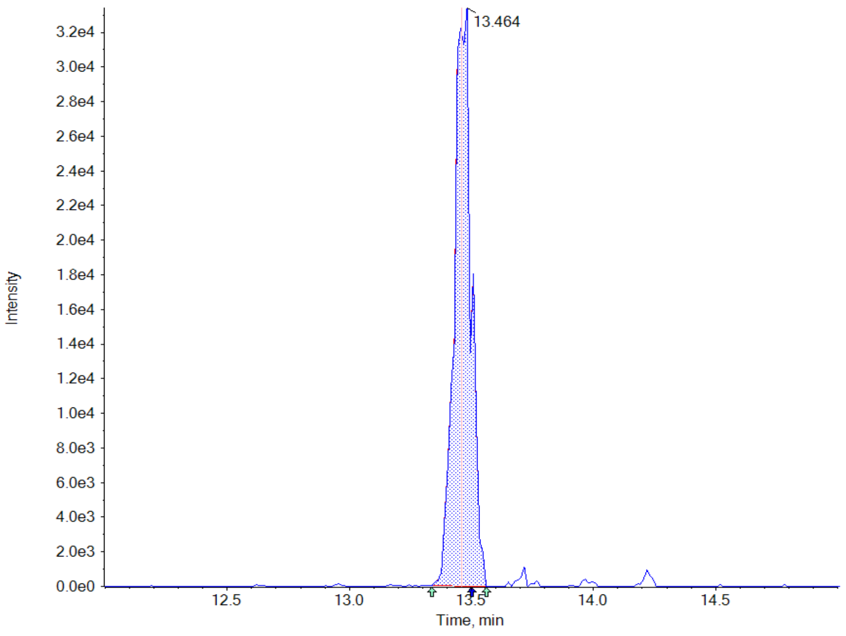

Acquired / Library MSMS

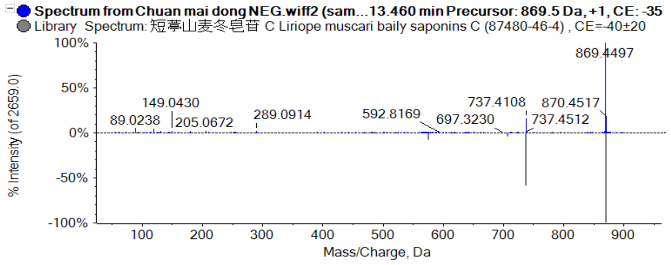

Acquired / Theoretical MS

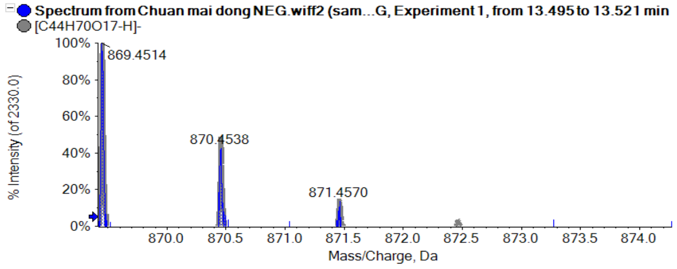

甲基麦冬高异黄酮A Methylophiopogonone A

Retention Time: 14.71 minutes **Exp RT:** 14.71 minutes
Precursor m/z : 339.0874 **Analyte Name:**
Fit (%) 98.7% **RFit (%)** 97.9% 甲基麦冬高异黄酮A Methylophiopogonone A

Collision Energy = 35 ± 15 eV

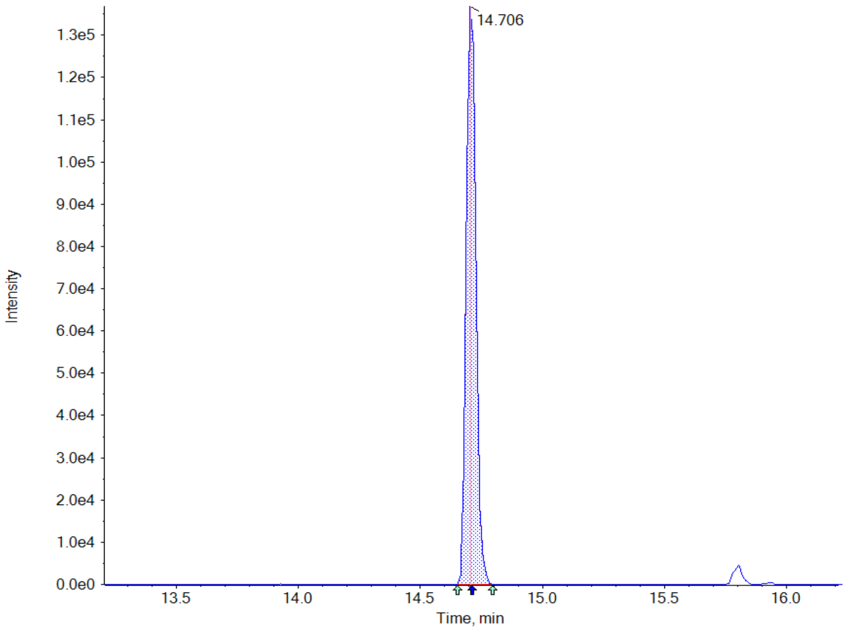

Acquired / Library MSMS

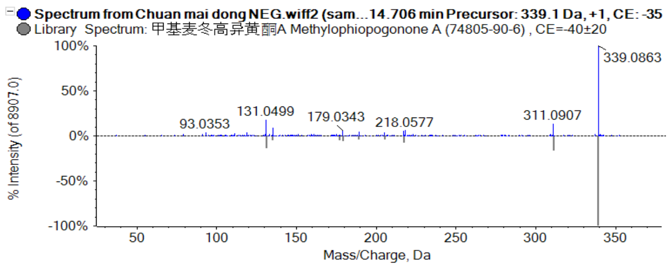

Acquired / Theoretical MS

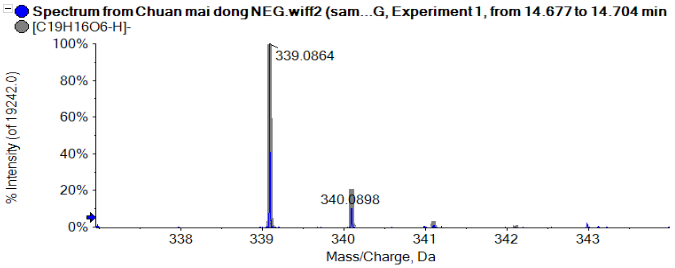

麦冬皂苷D Ophiopogonin D

Retention Time: 14.83 minutes **Exp RT:** 14.83 minutes
Precursor m/z : 899.4646 **Analyte Name:**
Fit (%) 100.0% **RFit (%)** 84.1% 麦冬皂苷D Ophiopogonin D +HCOOH

Collision Energy = 35 ± 15 eV

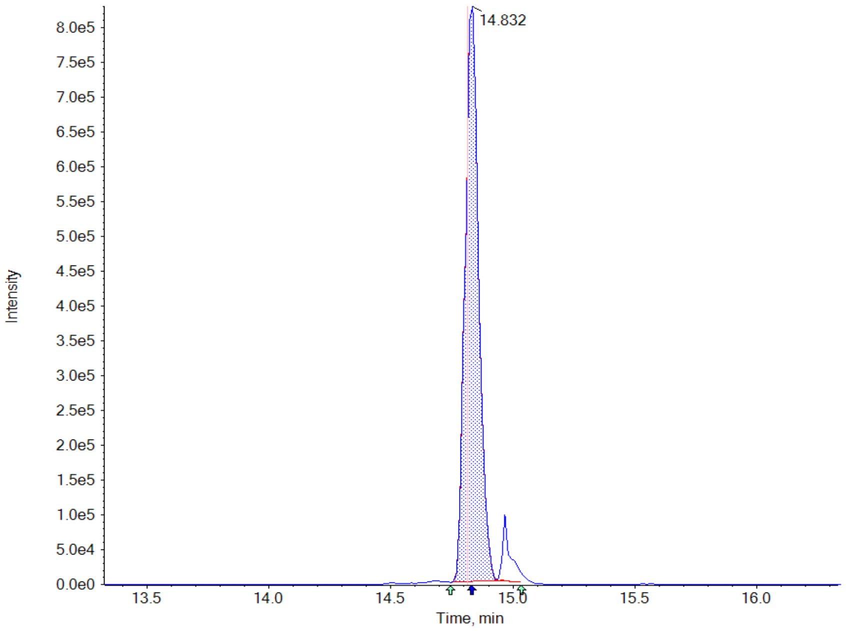

Acquired / Library MSMS

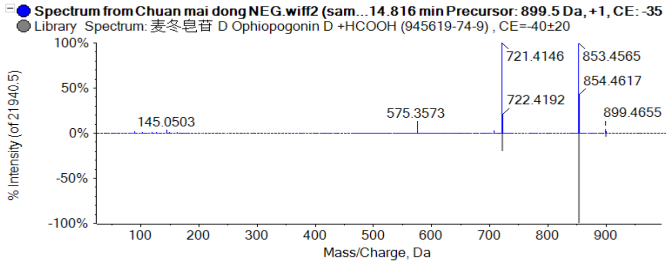

Acquired / Theoretical MS

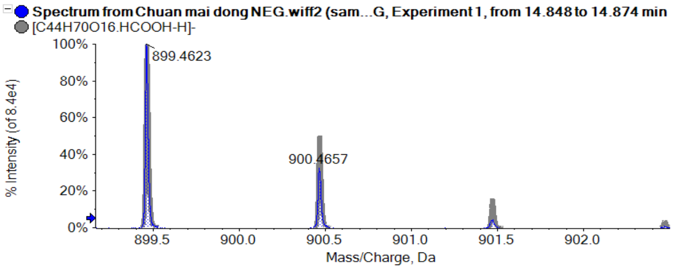

甲基麦冬二氢高异黄酮B Methylophiopogonanone B

Retention Time: 14.99 minutes
Precursor m/z : 327.1238
Fit (%) 90.0% **RFit (%)** 99.9%

Exp RT: 14.98 minutes
Analyte Name:
甲基麦冬二氢高异黄酮B Methylophiopogonanone B

Collision Energy = 35 ± 15 eV

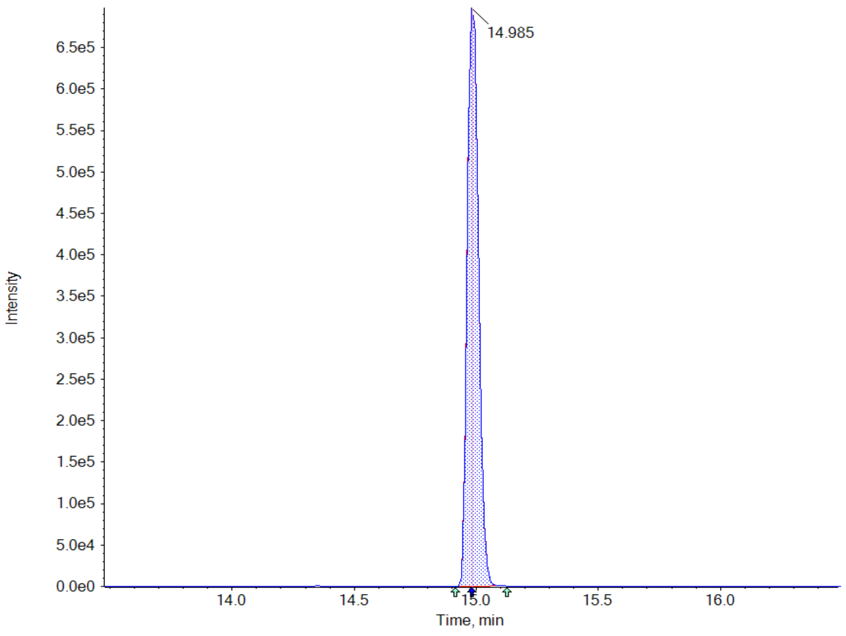

Acquired / Library MSMS

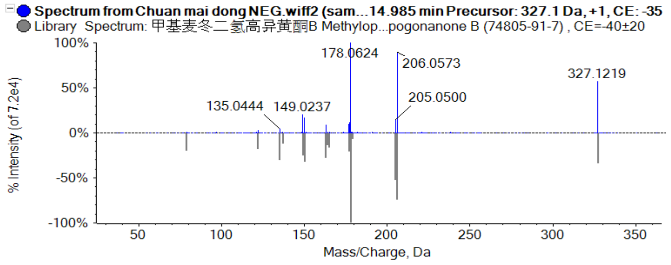

Acquired / Theoretical MS

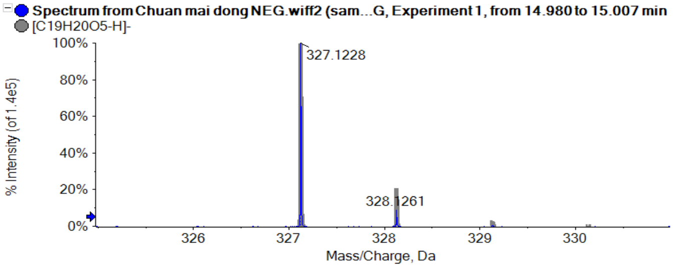

山麦冬皂苷B Liriopesides B

Retention Time: 15.12 minutes **Exp RT:** 15.12 minutes

Precursor m/z : 767.4223 **Analyte Name:**

Fit (%) 95.3% **RFit (%)** 100.0% 山麦冬皂苷B Liriopesides B +HCOOH

Collision Energy = 35 ± 15 eV

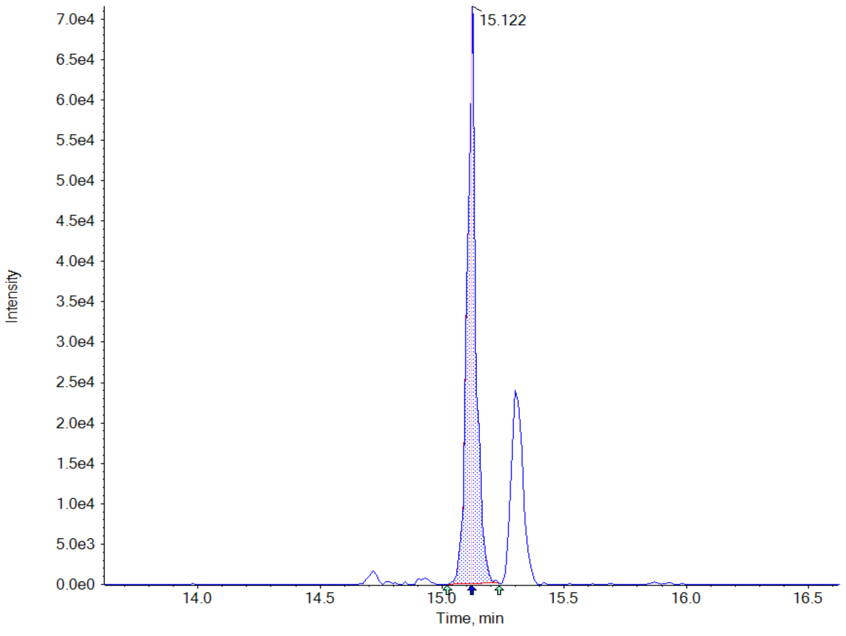

Acquired / Library MSMS

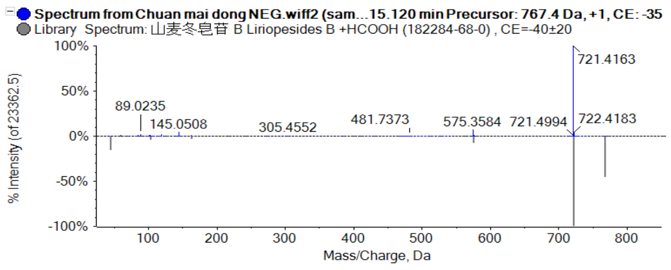

Acquired / Theoretical MS

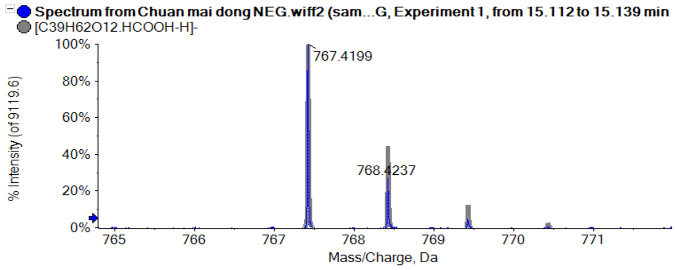

姜糖脂B Gingerglycolipid B

Retention Time: 15.20 minutes
Precursor m/z : 723.3809
Fit (%) 98.3% **RFit (%)** 100.0%

Exp RT: 15.21 minutes
Analyte Name:
姜糖脂B Gingerglycolipid B +HCOOH

Collision Energy = 35 ± 15 eV

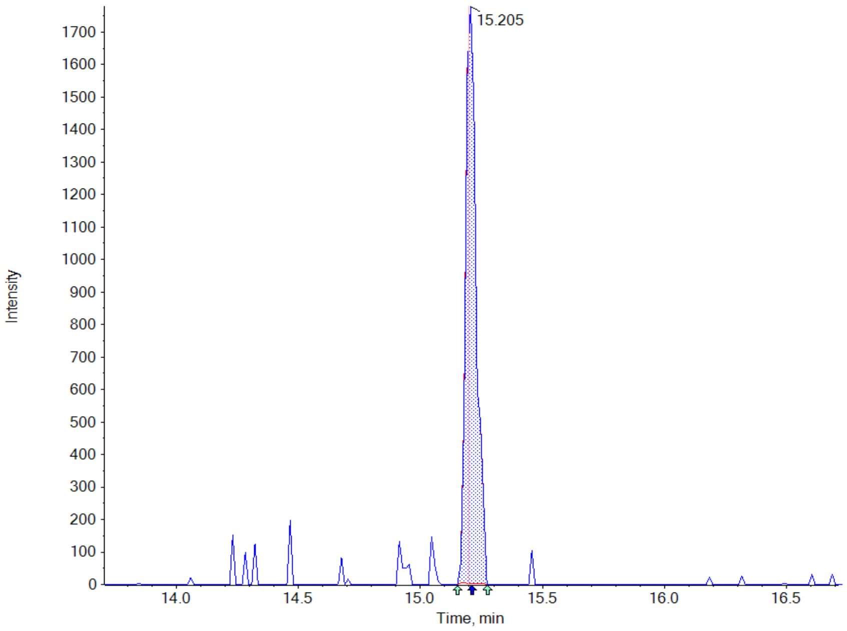

Acquired / Library MSMS

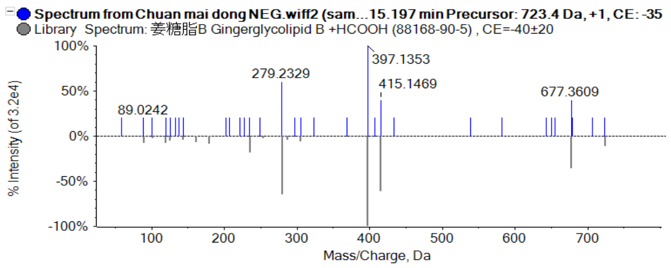

Acquired / Theoretical MS

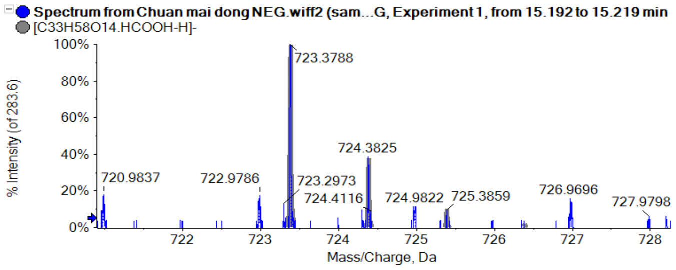

科罗索酸Corosolic acid

**Retention Time:** 15.55 minutes	**Exp RT:** 15.55 minutes
**Precursor m/z :** 471.3480	**Analyte Name:**
**Fit (%)** 100.0% **RFit (%)** 100.0%	科罗索酸Corosolic acid

Collision Energy = 35 ± 15 eV

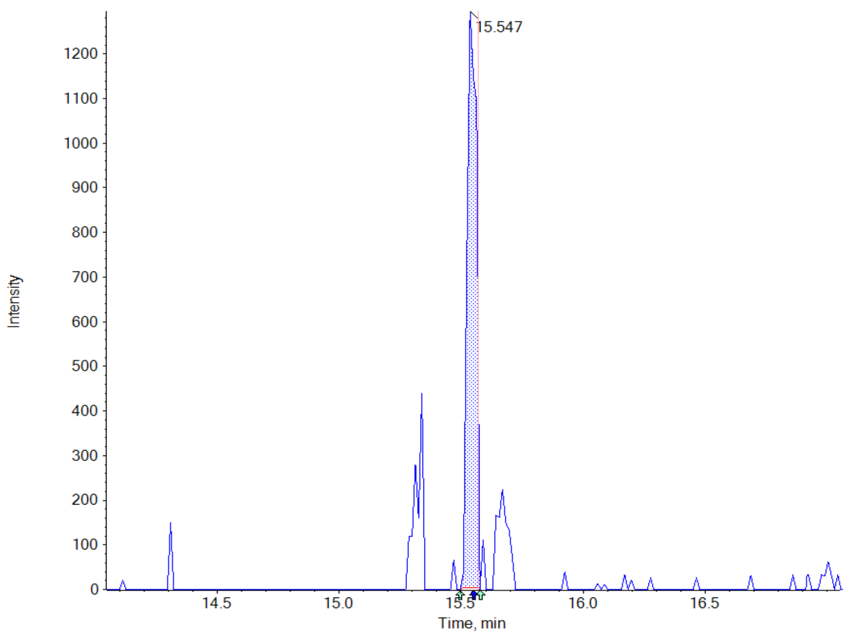

Acquired / Library MSMS

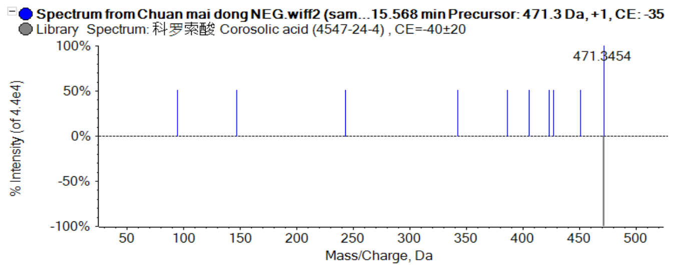

Acquired / Theoretical MS

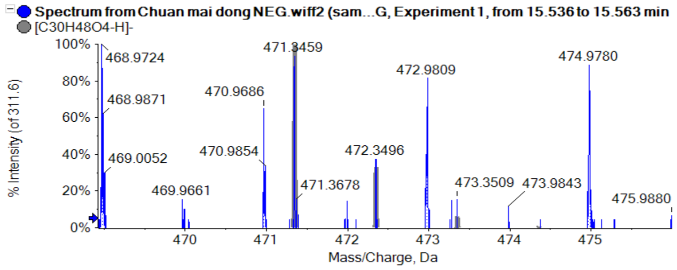

麦冬黄烷酮C Ophiopogonanone C

Retention Time: 15.72 minutes
Precursor m/z : 355.0823
Fit (%) 93.1% **RFit (%)** 99.0%

Exp RT: 15.72 minutes
Analyte Name:
麦冬黄烷酮C Ophiopogonanone C

Collision Energy = 35 ± 15 eV

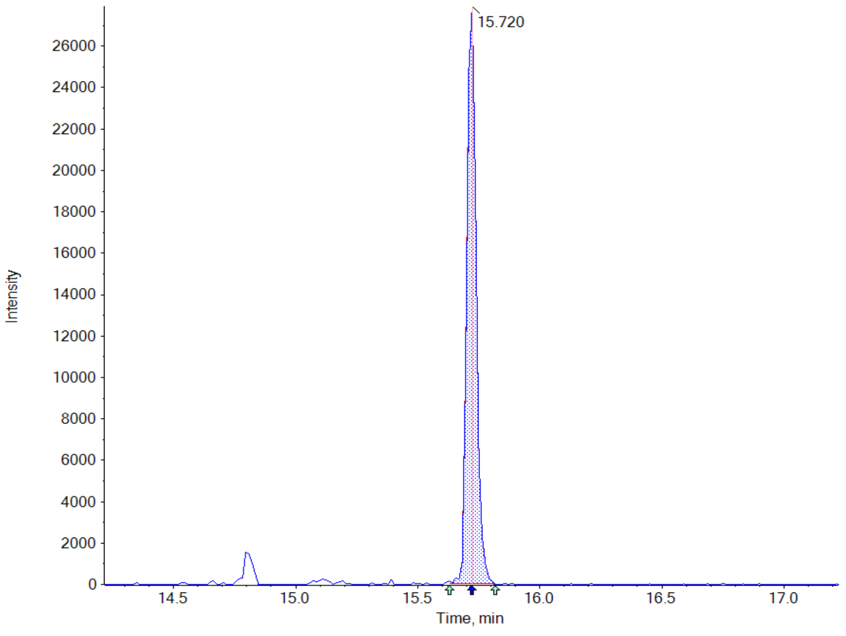

Acquired / Library MSMS

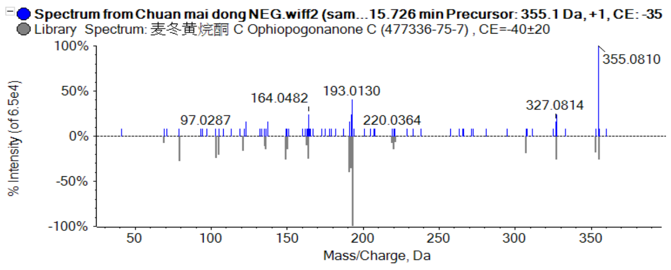

Acquired / Theoretical MS

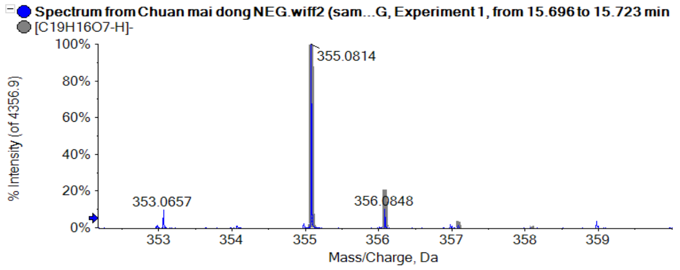

Table S2 MS fragmentation pathways for representative compounds of ZMD in UPLC-Q/TOF-MS analysis

MS fragmentation pathways for different chemical constituents of ZMD in positive ion mode

精氨酸L(+)-Arginine

Retention Time: 1.11 minutes
Precursor m/z : 175.1190
Fit (%) 100.0% **RFit (%)** 100.0%

Exp RT: 1.12 minutes
Analyte Name:
精氨酸L(+)-Arginine

Collision Energy = 35 ± 15 eV

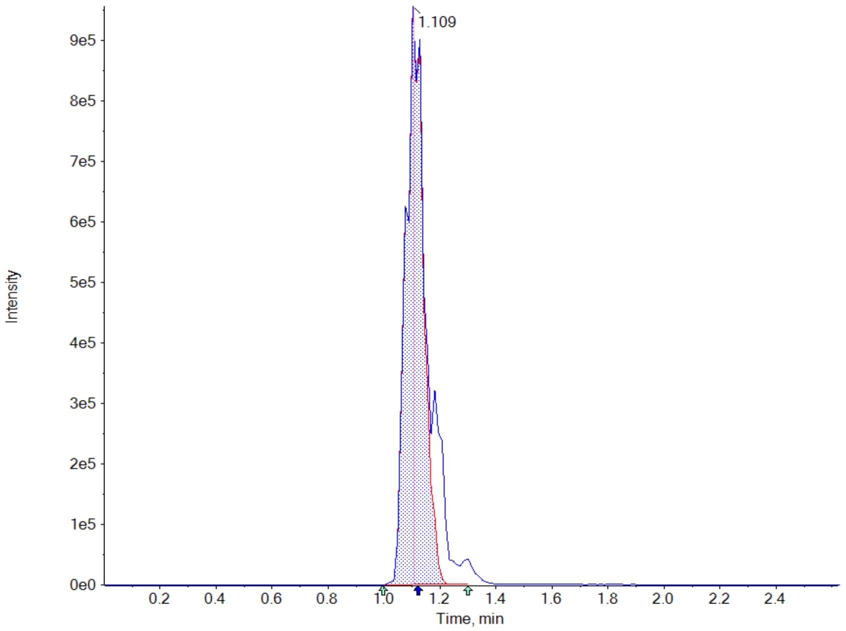

Acquired / Library MSMS

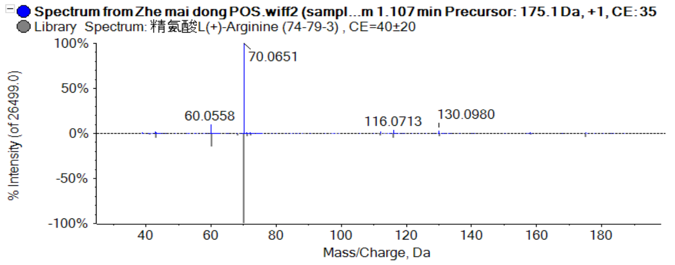

Acquired / Theoretical MS

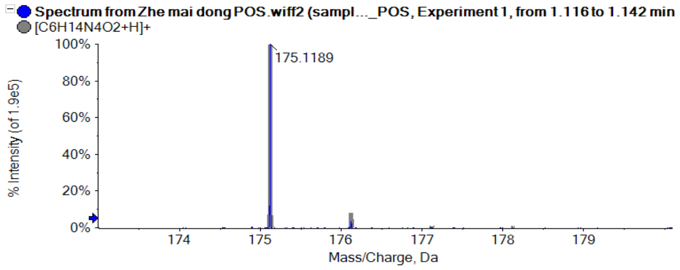

胡芦巴碱Trigonelline

Retention Time: 1.20 minutes
Precursor m/z : 138.0550
Fit (%) 96.8% **RFit (%)** 97.4%

Exp RT: 1.20 minutes
Analyte Name:
胡芦巴碱Trigonelline

Collision Energy = 35 ± 15 eV

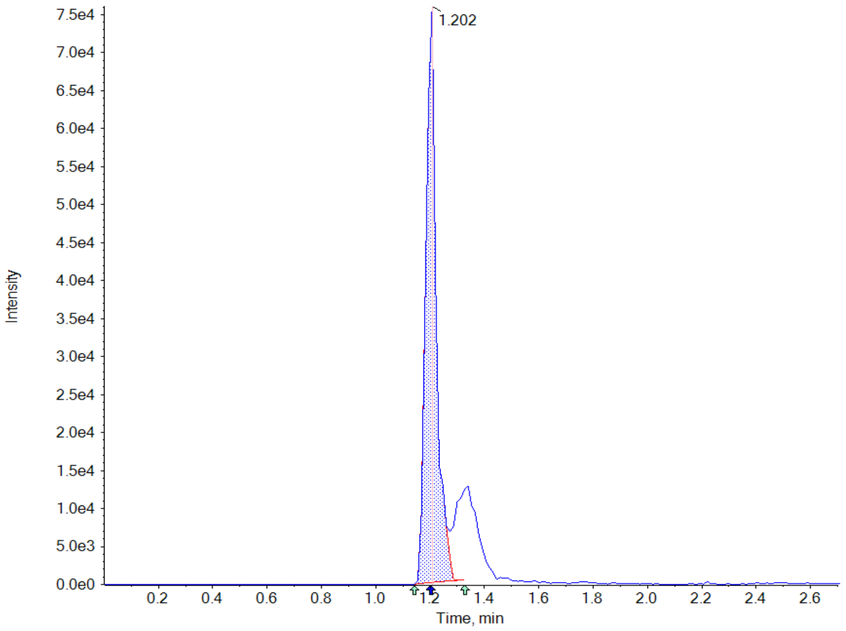

Acquired / Library MSMS

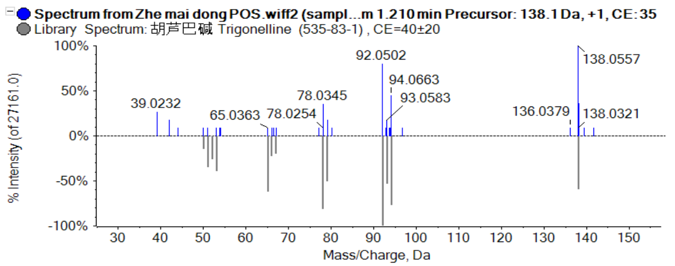

Acquired / Theoretical MS

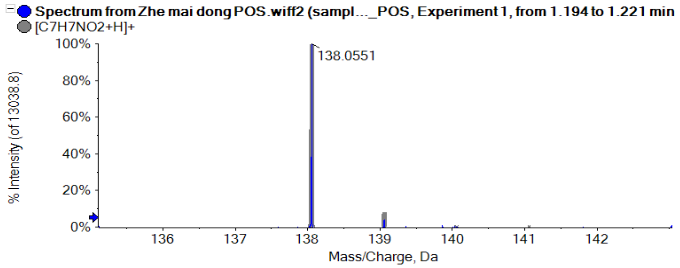

脯氨酸Proline

Retention Time: 1.22 minutes
Precursor m/z : 116.0706
Fit (%) 98.5% **RFit (%)** 100.0%

Exp RT: 1.22 minutes
Analyte Name:
脯氨酸Proline

Collision Energy = 35 ± 15 eV

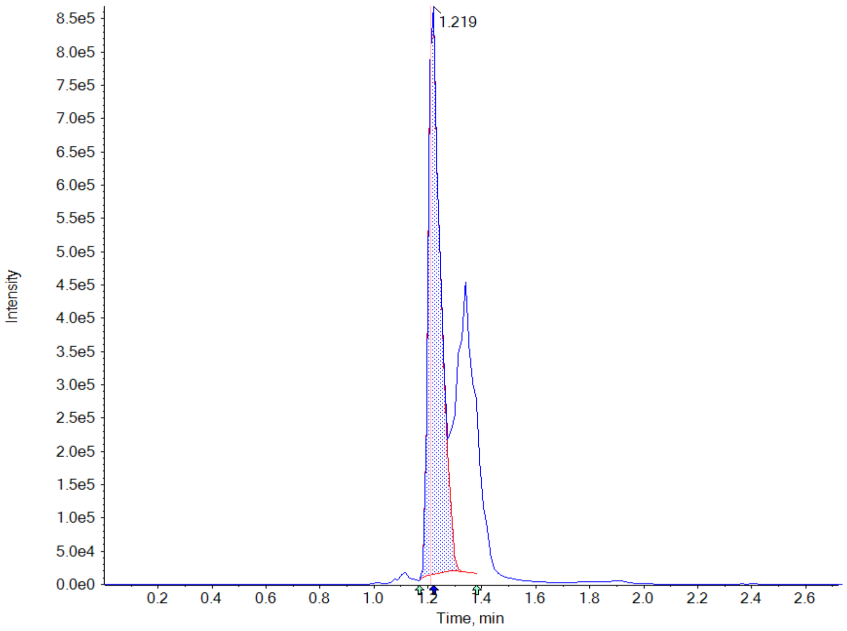

Acquired / Library MSMS

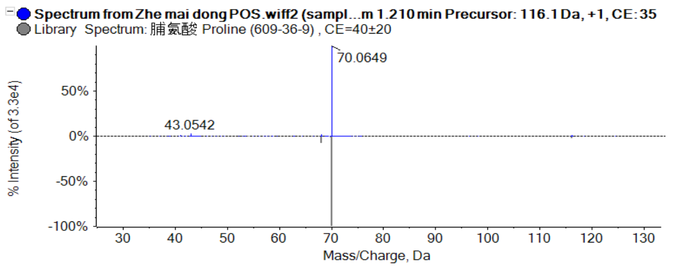

Acquired / Theoretical MS

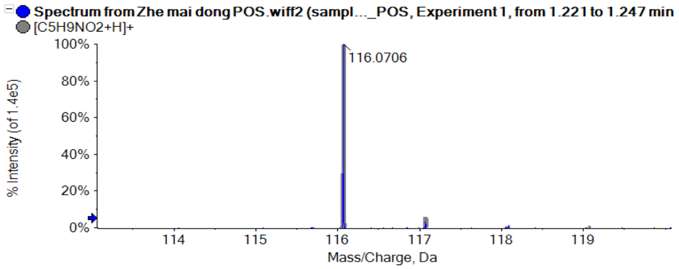

烟酸Nicotinic acid

Retention Time: 1.72 minutes
Precursor m/z : 124.0393
Fit (%) 97.5% **RFit (%)** 100.0%

Exp RT: 1.73 minutes
Analyte Name:
烟酸Nicotinic acid

Collision Energy = 35 ± 15 eV

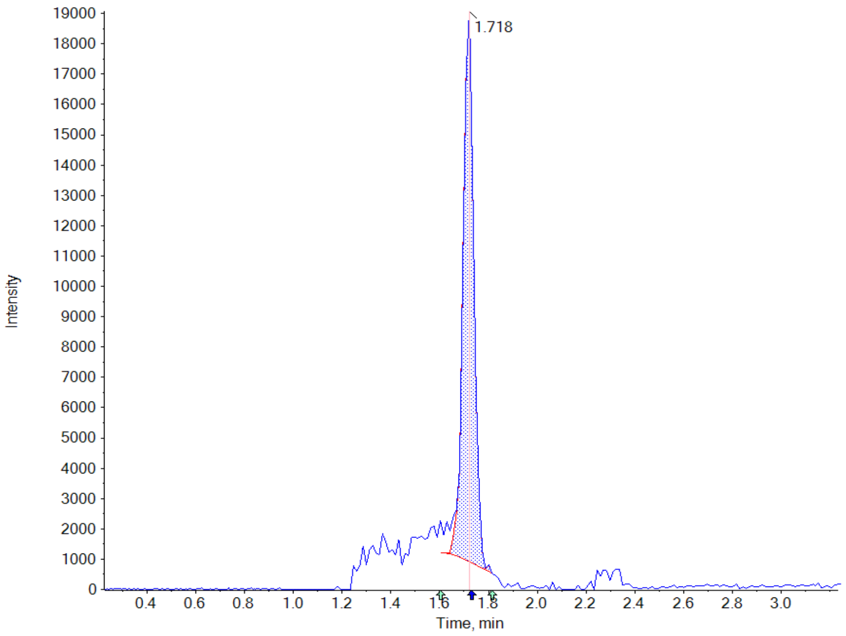

Acquired / Library MSMS

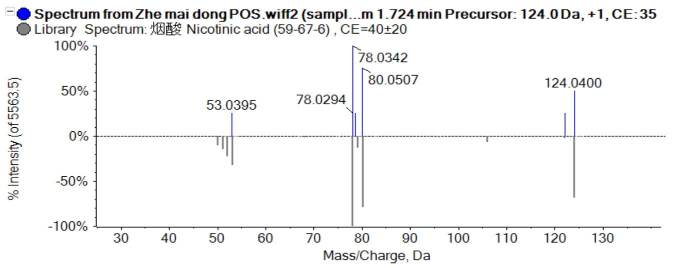

Acquired / Theoretical MS

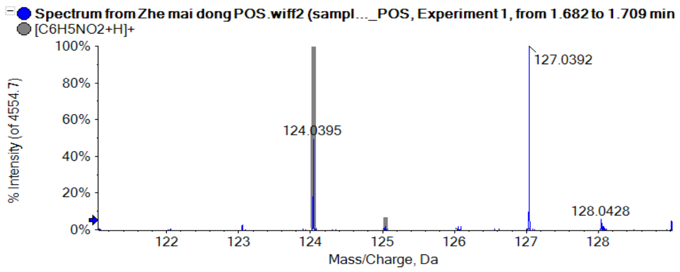

烟酰胺Nicotinamide

Retention Time: 1.80 minutes
Precursor m/z : 123.0553
Fit (%) 99.6% **RFit (%)** 100.0%

Exp RT: 1.79 minutes
Analyte Name:
烟酰胺Nicotinamide

Collision Energy = 35 ± 15 eV

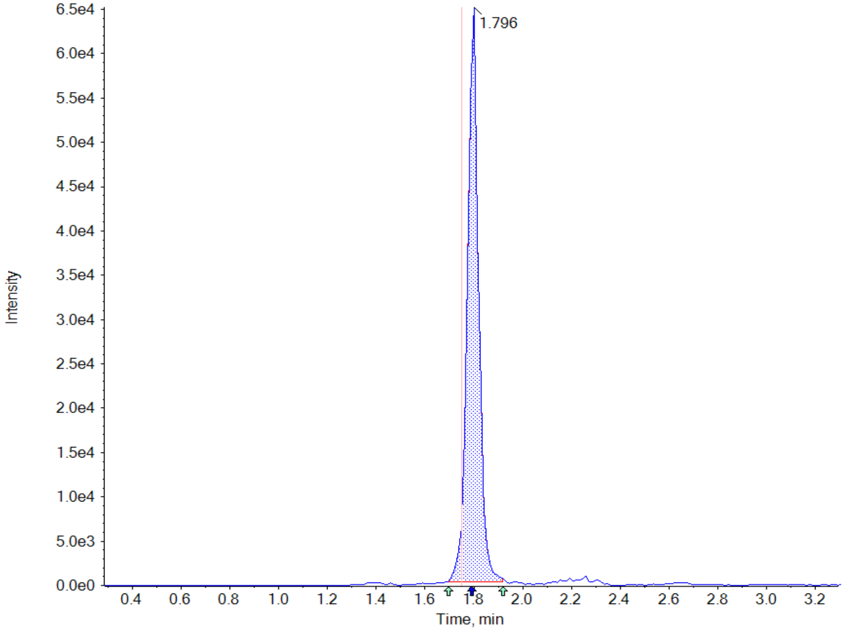

Acquired / Library MSMS

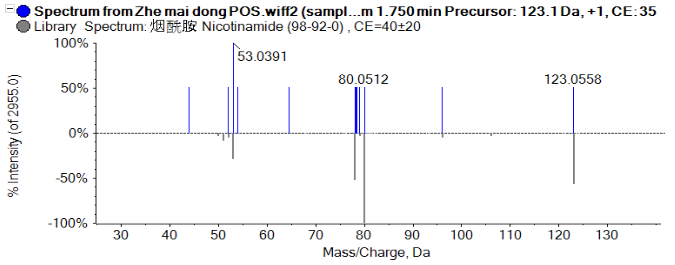

Acquired / Theoretical MS

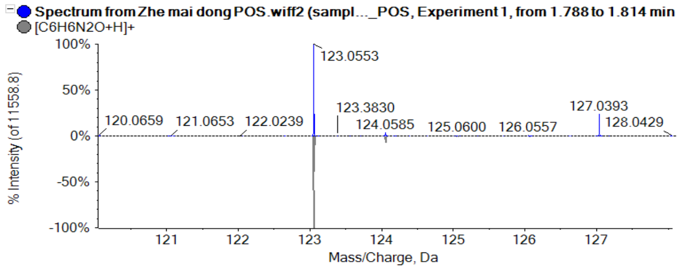

腺苷Adenosine

Retention Time: 2.35 minutes
Precursor m/z : 268.1040
Fit (%) 98.0% **RFit (%)** 100.0%

Exp RT: 2.42 minutes
Analyte Name:
腺苷Adenosine

Collision Energy = 35 ± 15 eV

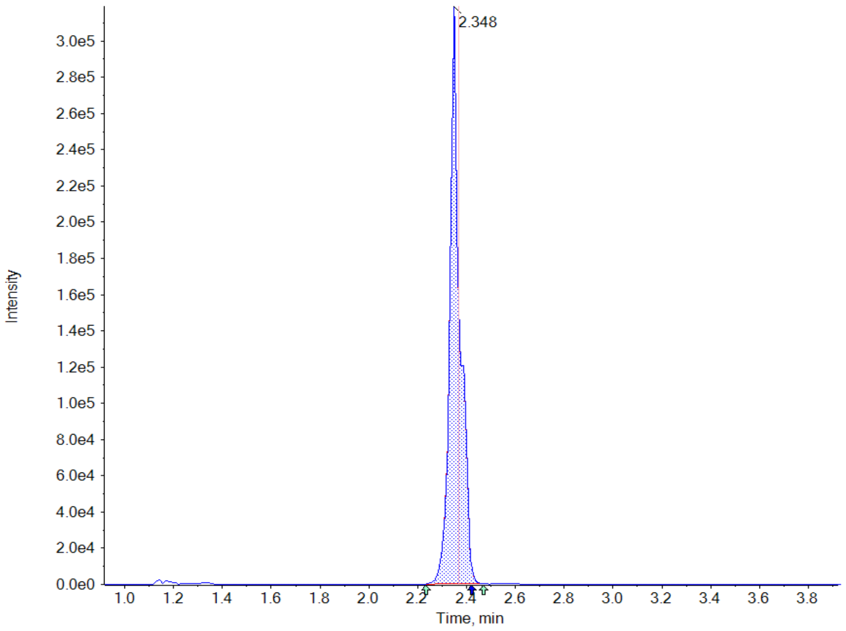

Acquired / Library MSMS

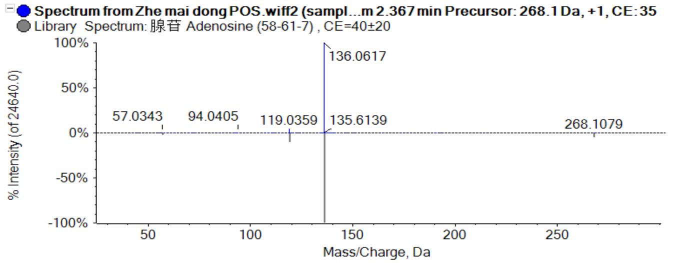

Acquired / Theoretical MS

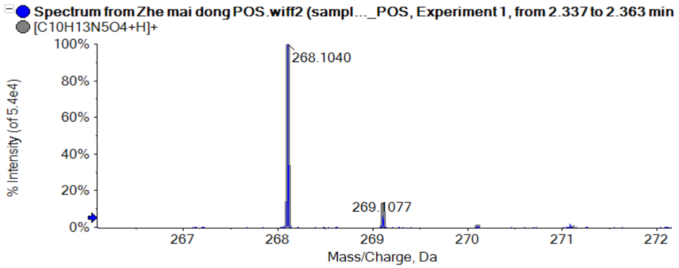

虫草素Cordycepin

Retention Time: 2.42 minutes
Precursor m/z : 252.1091
Fit (%) 100.0% **RFit (%)** 99.8%

Exp RT: 2.42 minutes
Analyte Name:
虫草素Cordycepin

Collision Energy = 35 ± 15 eV

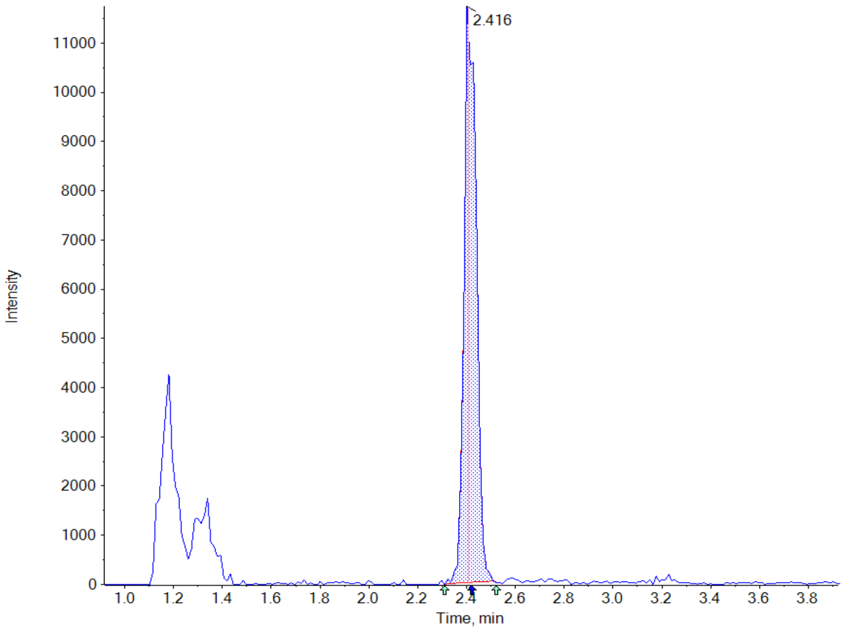

Acquired / Library MSMS

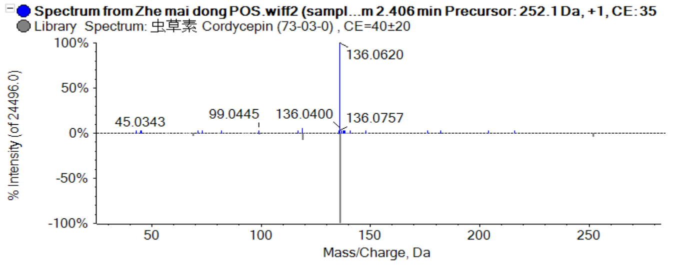

Acquired / Theoretical MS

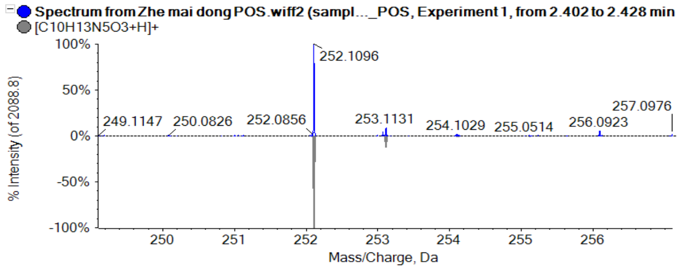

异亮氨酸Isoleucine

Retention Time: 2.46 minutes
Precursor m/z : 132.1019
Fit (%) 95.8% **RFit (%)** 89.2%

Exp RT: 2.47 minutes
Analyte Name:
异亮氨酸Isoleucine

Collision Energy = 35 ± 15 eV

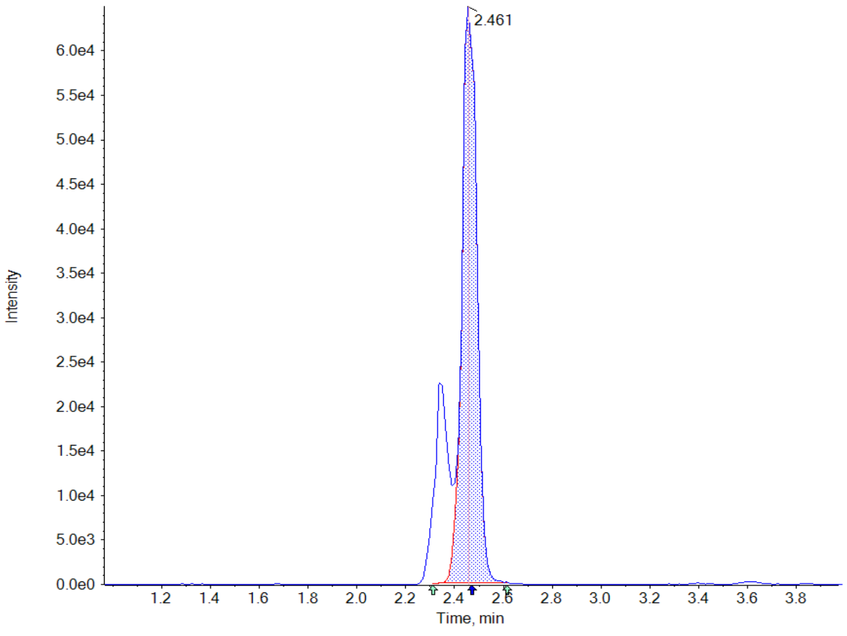

Acquired / Library MSMS

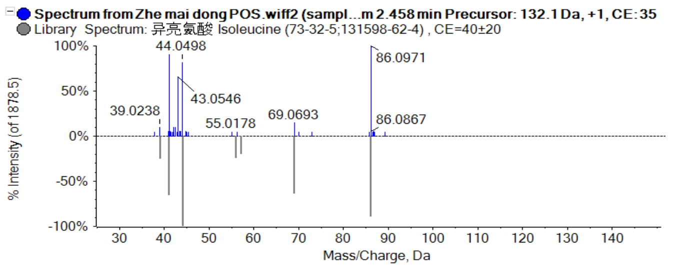

Acquired / Theoretical MS

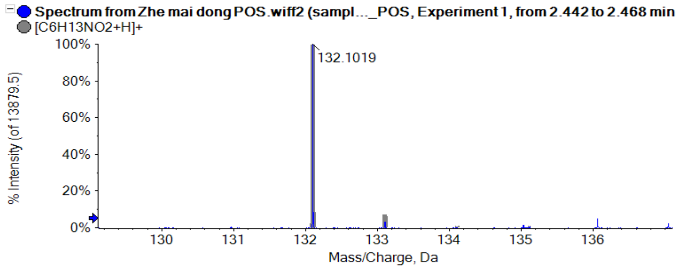

鸟苷Guanosine

Retention Time: 2.47 minutes
Precursor m/z : 284.0989
Fit (%) 99.1% **RFit (%)** 100.0%

Exp RT: 2.56 minutes
Analyte Name:
鸟苷Guanosine

Collision Energy = 35 ± 15 eV

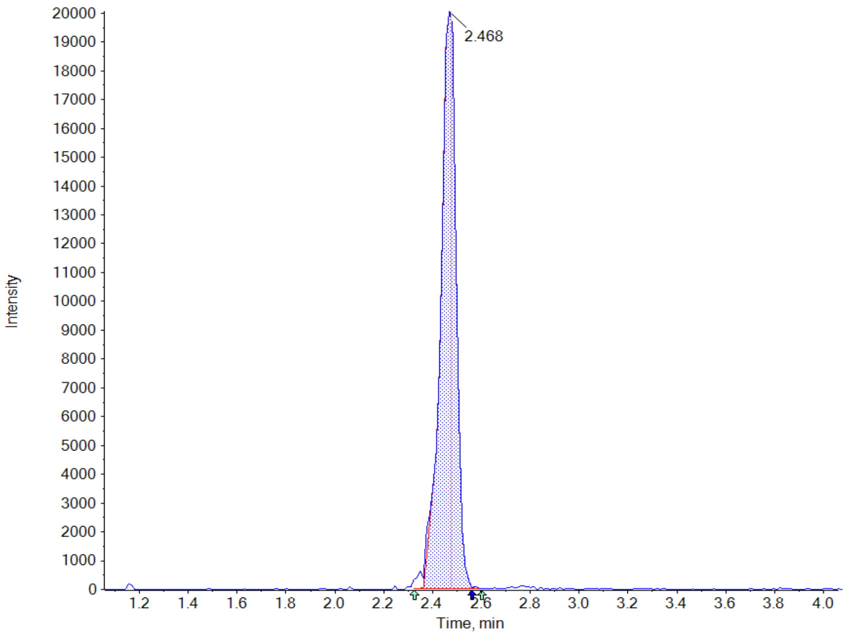

Acquired / Library MSMS

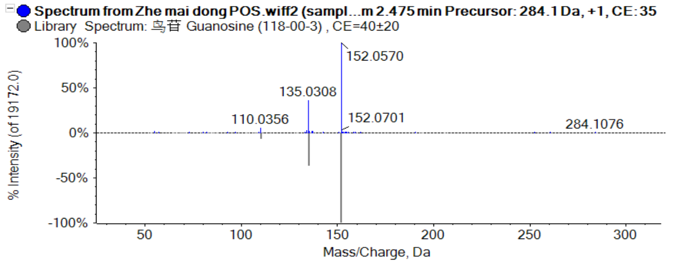

Acquired / Theoretical MS

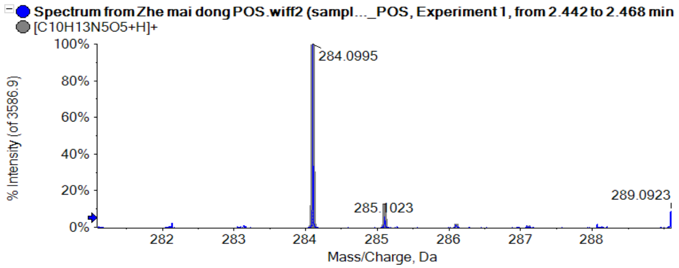

苯丙氨酸Phenprobamate

Retention Time: 3.17 minutes
Precursor m/z : 166.0863
Fit (%) 96.9% **RFit (%)** 100.0%

Exp RT: 3.31 minutes
Analyte Name:
苯丙氨酸Phenprobamate

Collision Energy = 35 ± 15 eV

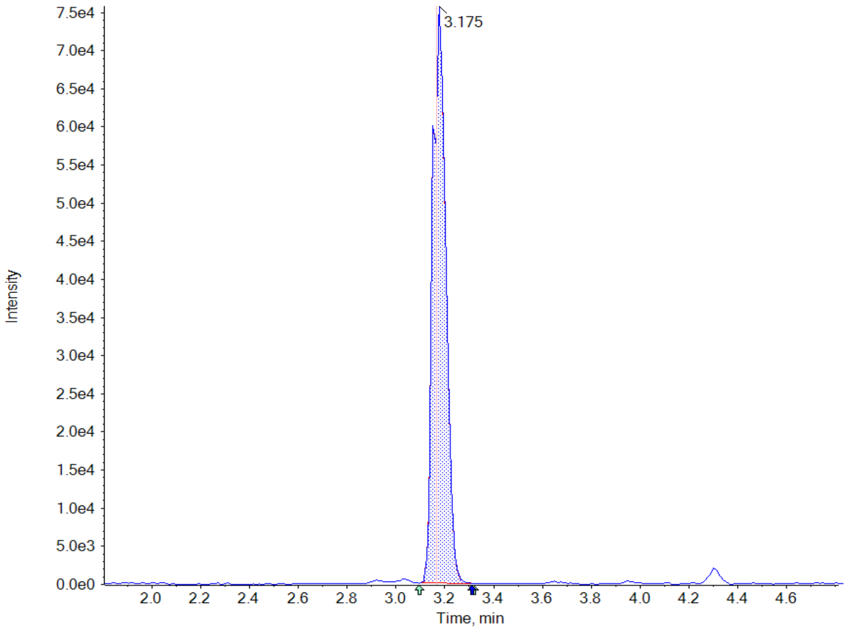

Acquired / Library MSMS

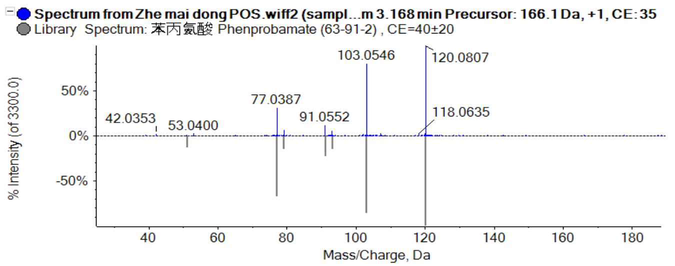

Acquired / Theoretical MS

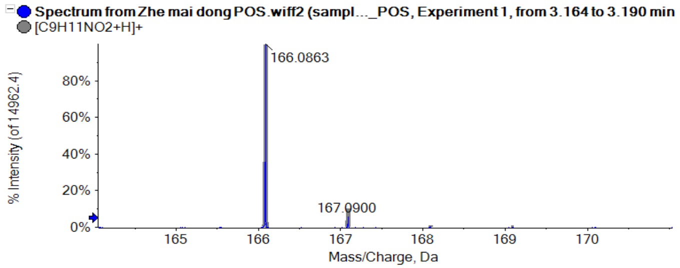

绿原酸Chlorogenic acid

Retention Time: 4.64 minutes

Precursor m/z : 355.1024

Fit (%) 96.5% **RFit (%)** 97.3%

Exp RT: 4.64 minutes

Analyte Name:

绿原酸Chlorogenic acid

Collision Energy = 35 ± 15 eV

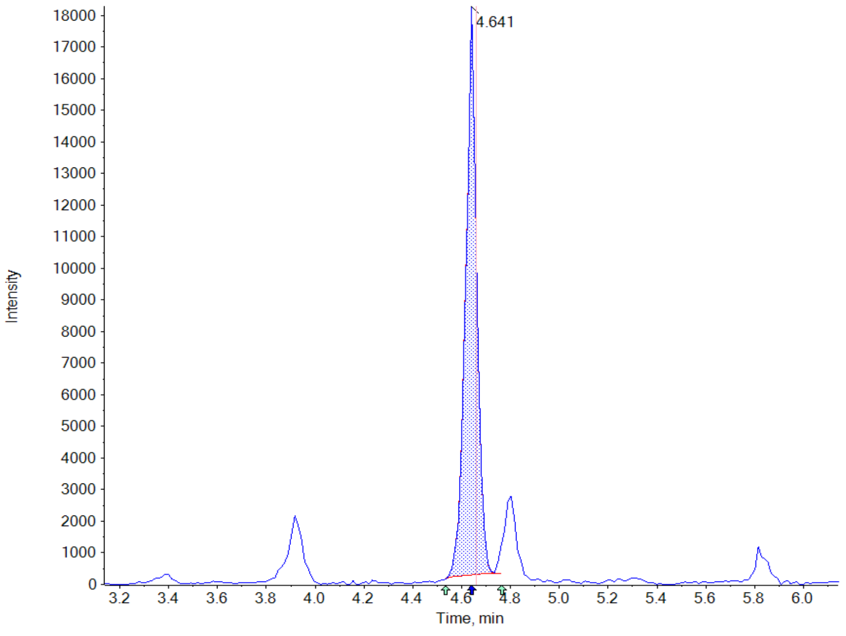

Acquired / Library MSMS

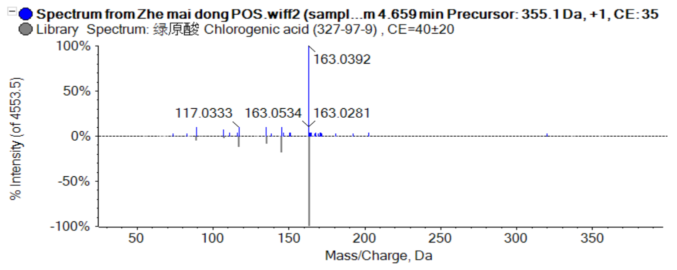

Acquired / Theoretical MS

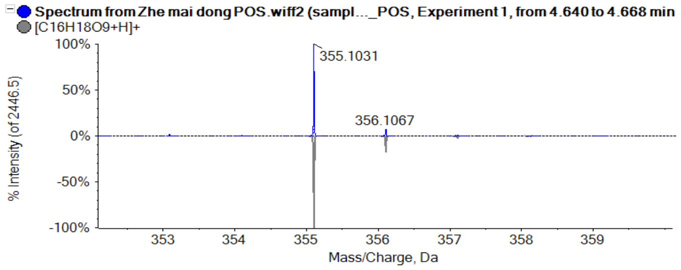

对羟基苯甲酸4-Hydroxybenzoic acid

Retention Time: 4.73 minutes
Exp RT: 4.81 minutes
Precursor m/z : 139.0390
Analyte Name: 对羟基苯甲酸4-Hydroxybenzoic acid
Fit (%) 92.6% **RFit (%)** 99.4%

Collision Energy = 35 ± 15 eV

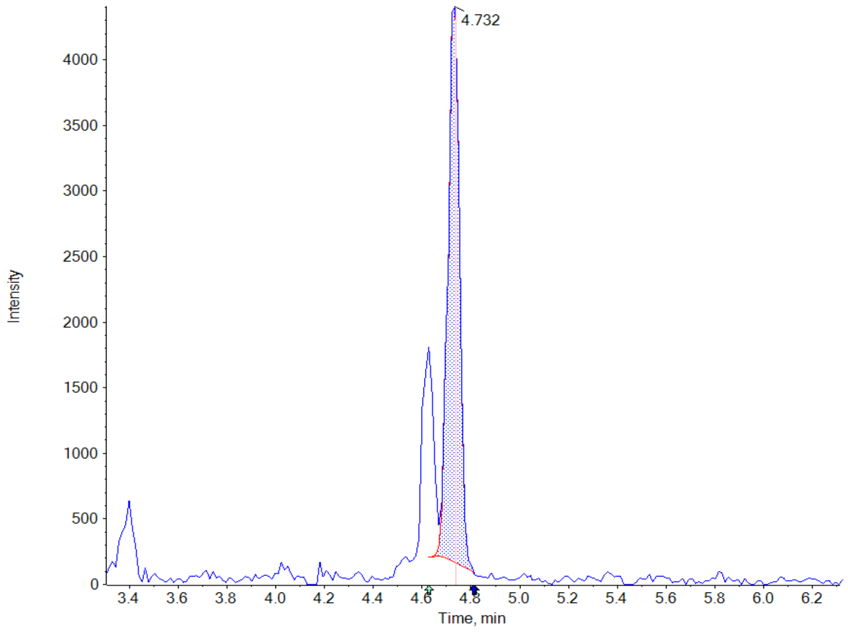

Acquired / Library MSMS

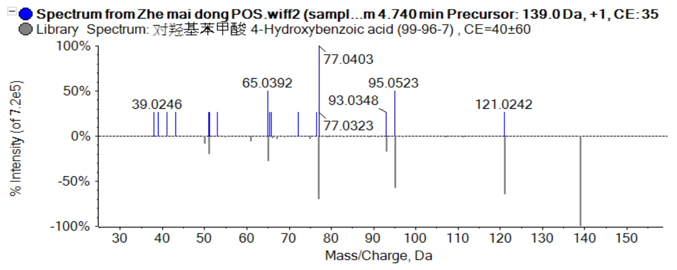

Acquired / Theoretical MS

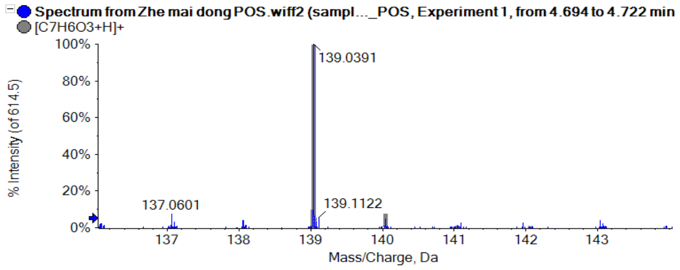

瑞香素Daphnetin

Retention Time: 5.13 minutes **Exp RT:** 5.14 minutes

Precursor m/z : 177.0193 **Analyte Name:**

Fit (%) 92.9% **RFit (%)** 89.3% 瑞香素Daphnetin

Collision Energy = 35 ± 15 eV

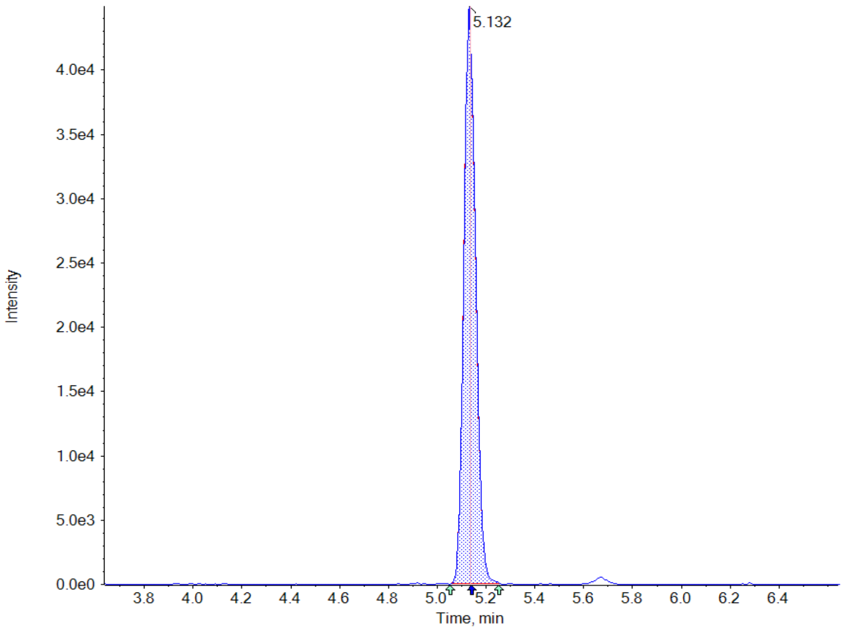

Acquired / Library MSMS

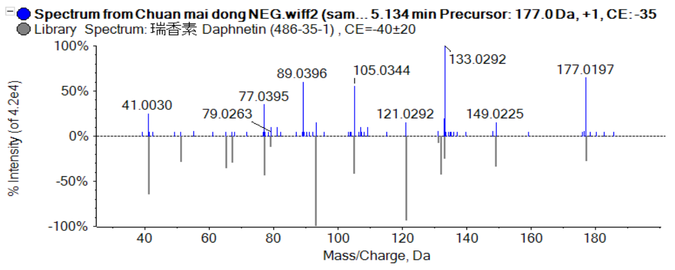

Acquired / Theoretical MS

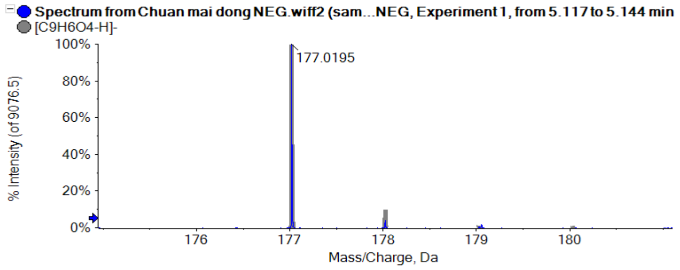

秦皮乙素Esculetin

Retention Time: 5.13 minutes
Precursor m/z : 179.0339
Fit (%) 86.6% **RFit (%)** 99.2%

Exp RT: 5.13 minutes
Analyte Name:
秦皮乙素Esculetin

Collision Energy = 35 ± 15 eV

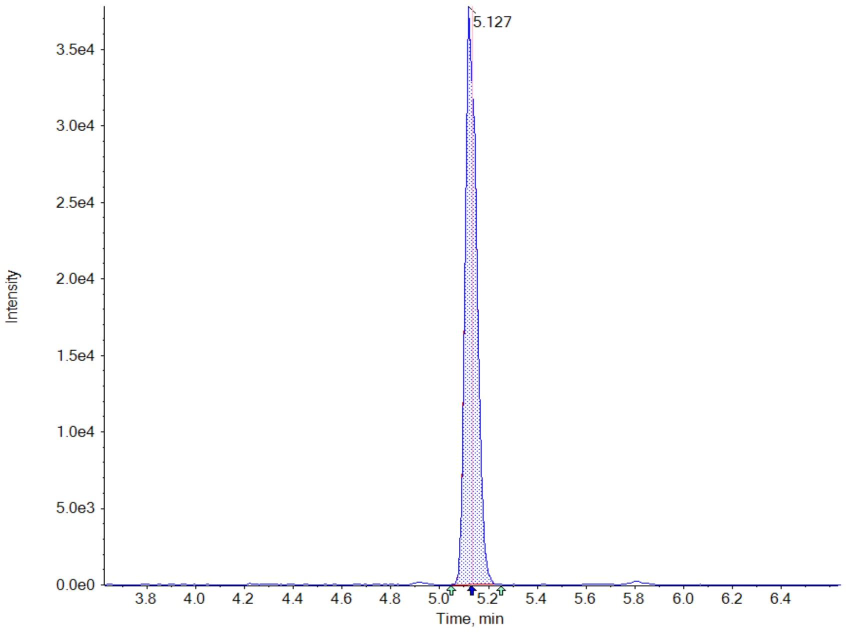

Acquired / Library MSMS

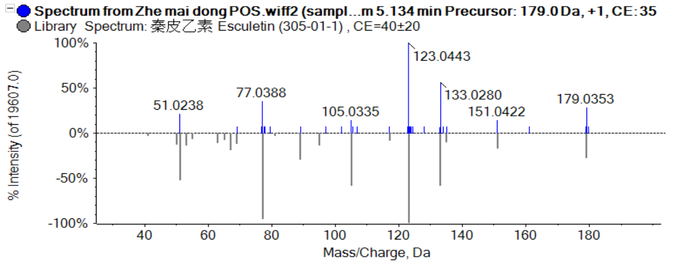

Acquired / Theoretical MS

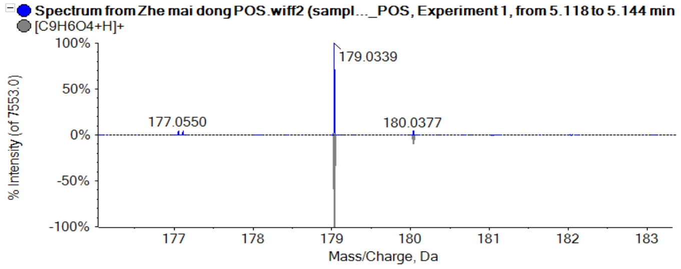

芦丁Rutin

Retention Time: 6.30 minutes
Precursor m/z : 609.1461
Fit (%) 93.3% **RFit (%)** 99.9%

Exp RT: 6.30 minutes
Analyte Name:
芦丁Rutin

Collision Energy = 35 ± 15 eV

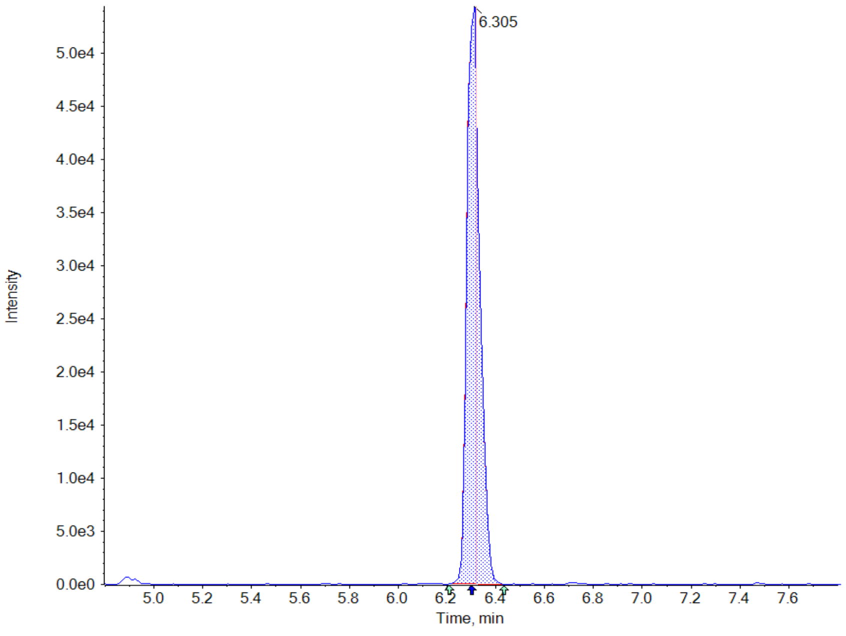

Acquired / Library MSMS

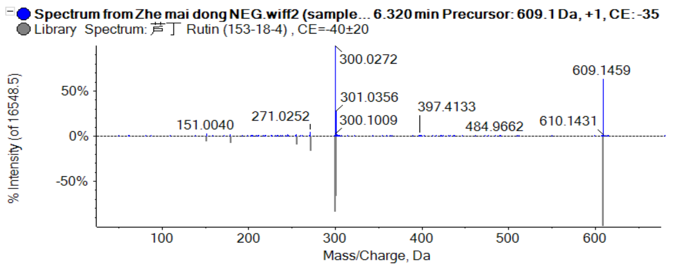

Acquired / Theoretical MS

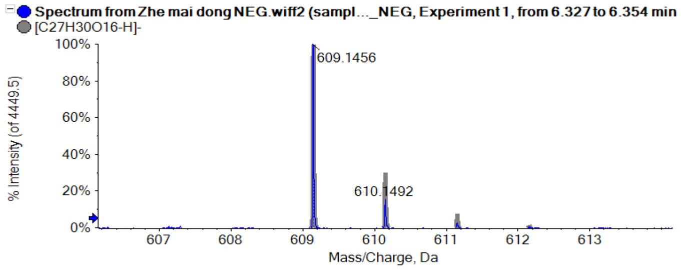

金丝桃苷Hyperin

Retention Time: 6.51 minutes
Precursor m/z : 463.0882
Fit (%) 98.4% **RFit (%)** 100.0%

Exp RT: 6.50 minutes
Analyte Name:
金丝桃苷Hyperin

Collision Energy = 35 ± 15 eV

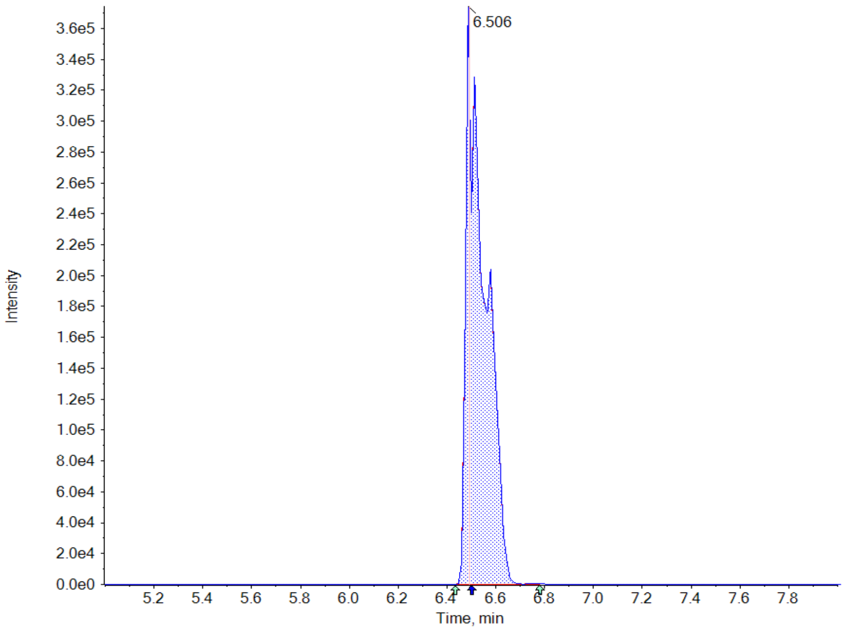

Acquired / Library MSMS

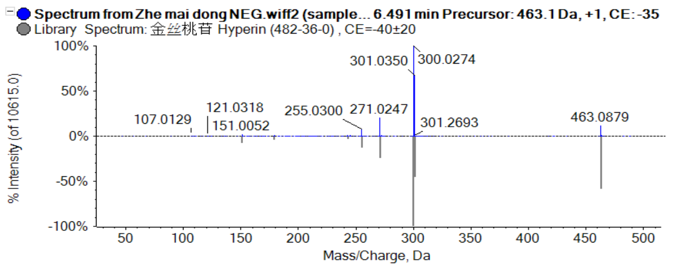

Acquired / Theoretical MS

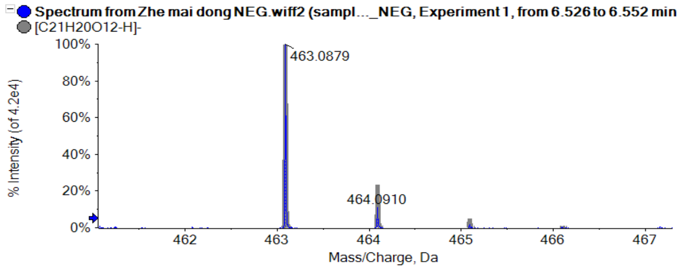

6-羟基-7-甲氧基香豆素

Retention Time: 6.69 minutes **Exp RT:** 6.67 minutes

Precursor m/z : 193.0495 **Analyte Name:**

Fit (%) 98.8% **RFit (%)** 100.0% 6-羟基-7-甲氧基香豆素 Isoscopoletin

Collision Energy = 35 ± 15 eV

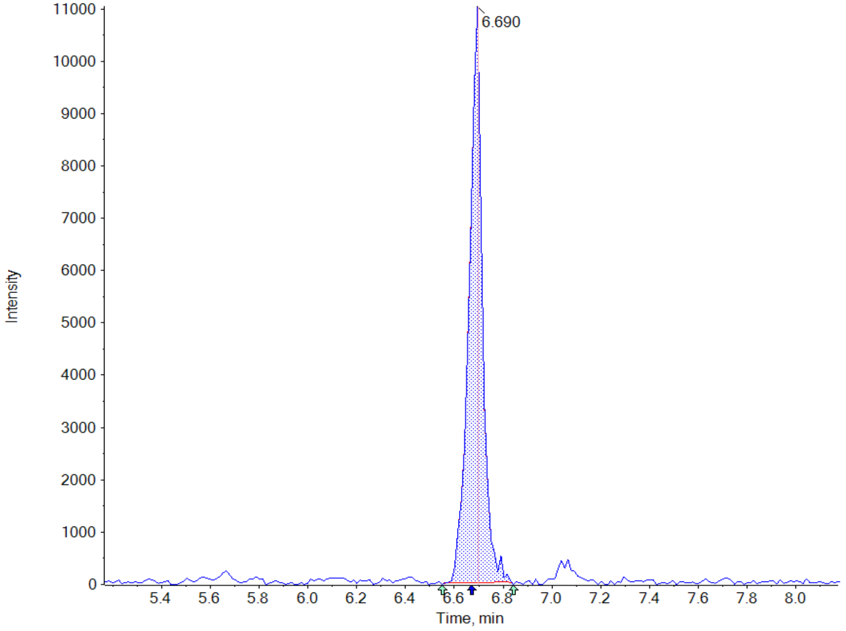

Acquired / Library MSMS

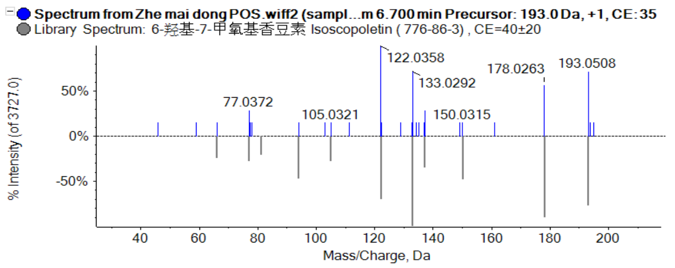

Acquired / Theoretical MS

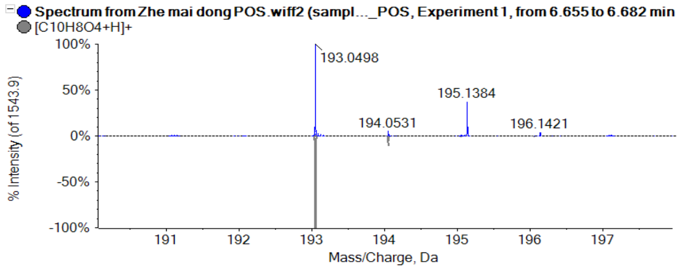

异阿魏酸Isoferulic acid

Retention Time: 6.74 minutes
Precursor m/z : 195.0652
Fit (%) 92.3% **RFit (%)** 100.0%

Exp RT: 6.75 minutes
Analyte Name:
异阿魏酸Isoferulic acid

Collision Energy = 35 ± 15 eV

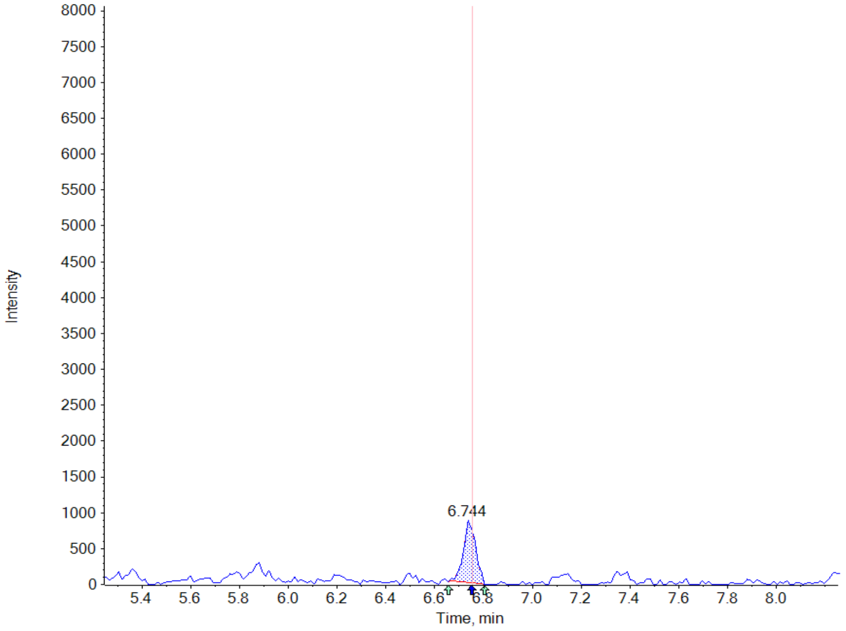

Acquired / Library MSMS

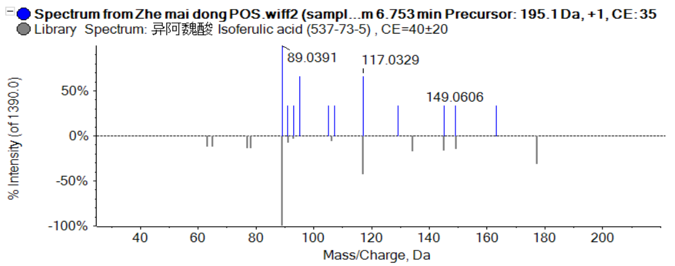

Acquired / Theoretical MS

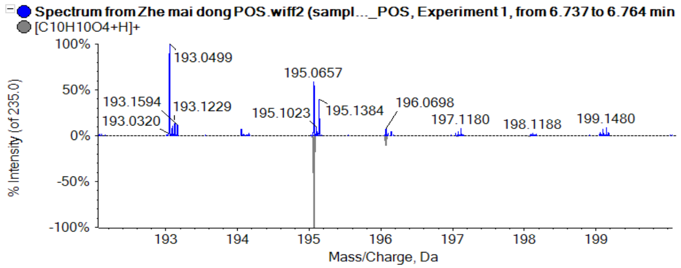

木犀草苷Luteoloside (Mass/FragMass/RT/Isotope/Library/Formula/Ion Ratio)

Retention Time: 6.96 minutes **Exp RT:** 6.91 minutes

Precursor m/z : 449.1078 **Analyte Name:**

Fit (%) 100.0% **RFit (%)** 100.0% 木犀草苷Luteoloside

Collision Energy = 35 ± 15 eV

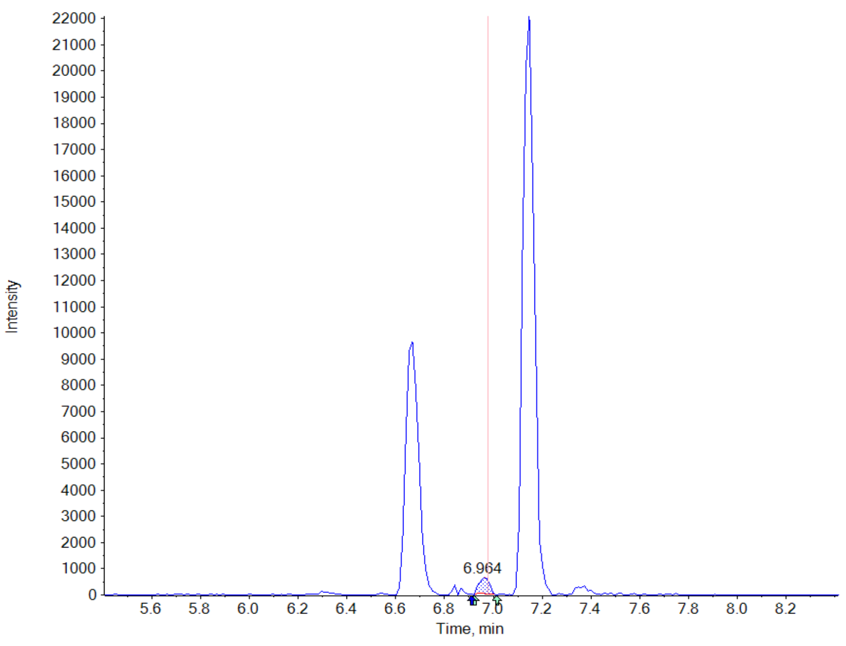

Acquired / Library MSMS

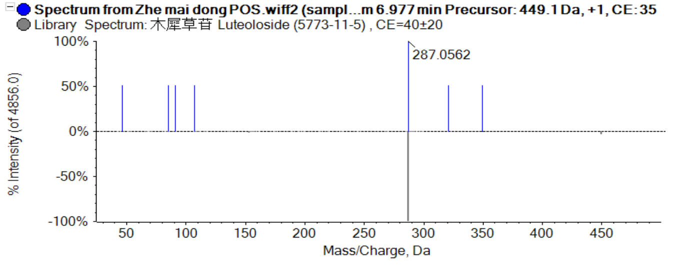

Acquired / Theoretical MS

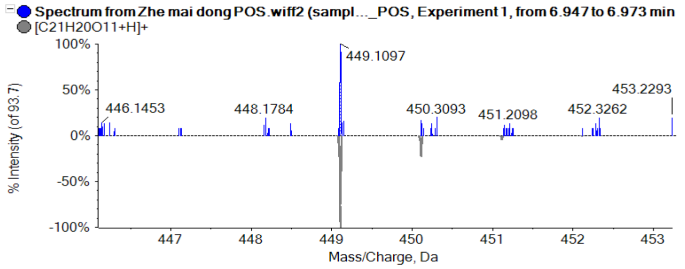

芸香柚皮苷Narirutin

Retention Time: 7.08 minutes
Precursor m/z : 581.1865
Fit (%) 93.0% **RFit (%)** 99.9%

Exp RT: 7.10 minutes
Analyte Name:
芸香柚皮苷Narirutin

Collision Energy = 35 ± 15 eV

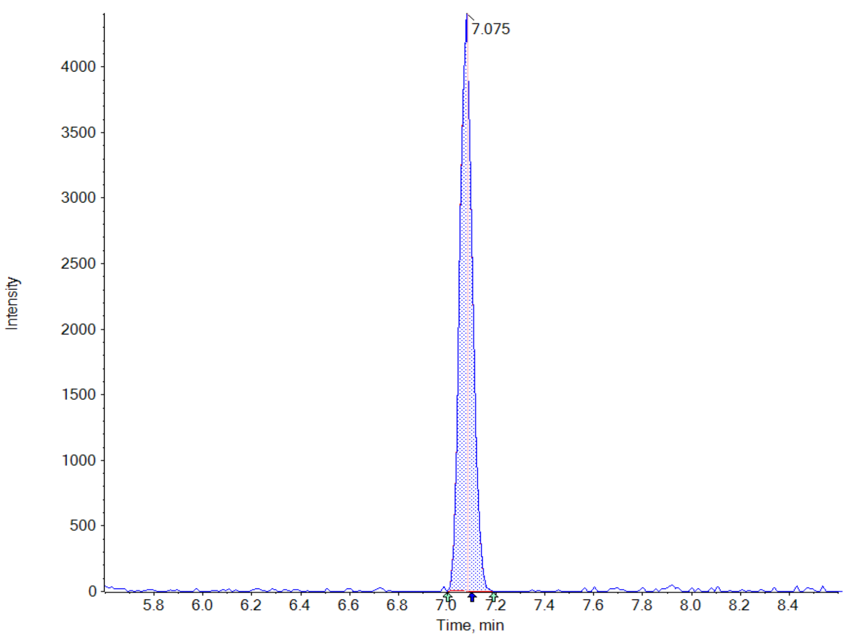

Acquired / Library MSMS

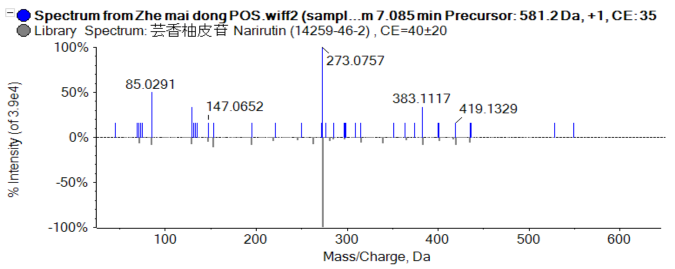

Acquired / Theoretical MS

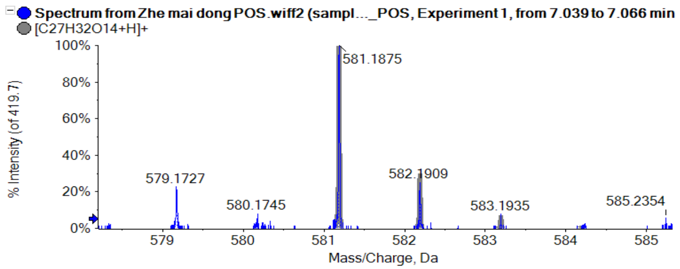

木犀草素Luteolin

Retention Time: 7.14 minutes **Exp RT:** 7.15 minutes
Precursor m/z : 287.0550 **Analyte Name:**
Fit (%) 100.0% **RFit (%)** 90.7% 木犀草素Luteolin

Collision Energy = 35 ± 15 eV

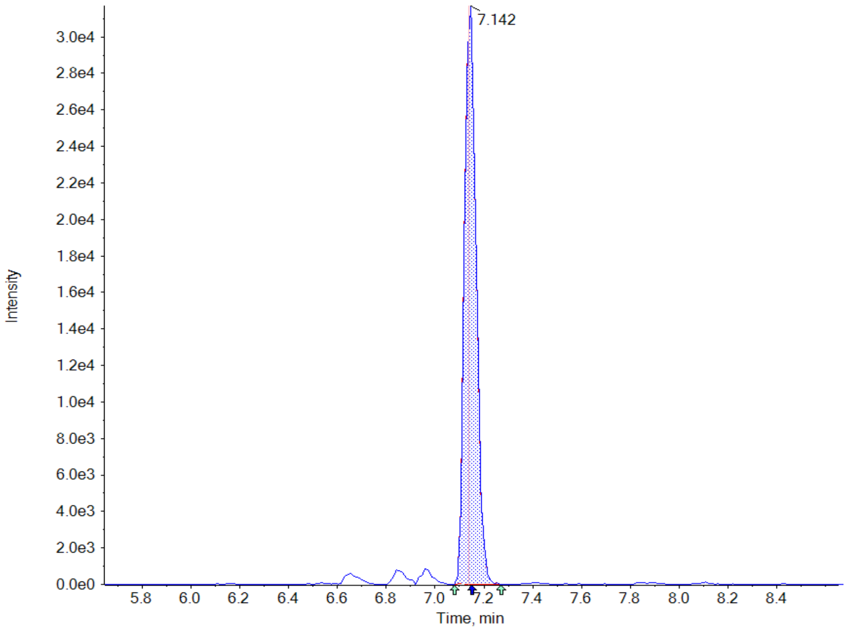

Acquired / Library MSMS

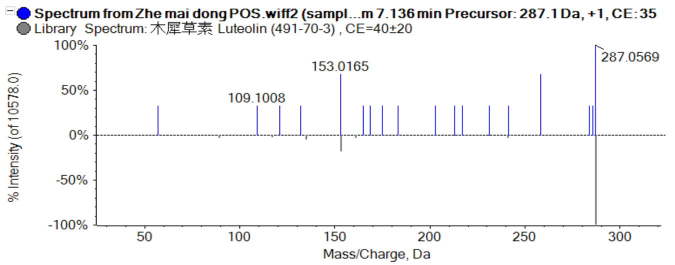

Acquired / Theoretical MS

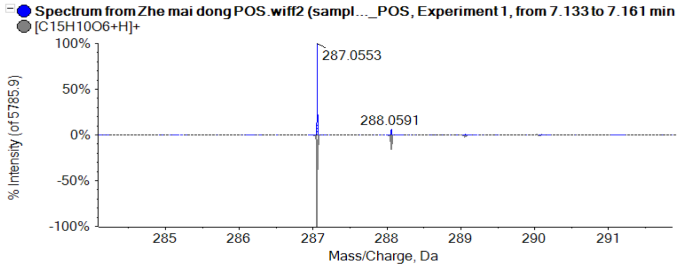

染料木苷Genistin

Retention Time: 7.38 minutes
Precursor m/z : 433.1129
Fit (%) 95.7% **RFit (%)** 97.8%

Exp RT: 7.38 minutes
Analyte Name: 染料木苷Genistin

Collision Energy = 35 ± 15 eV

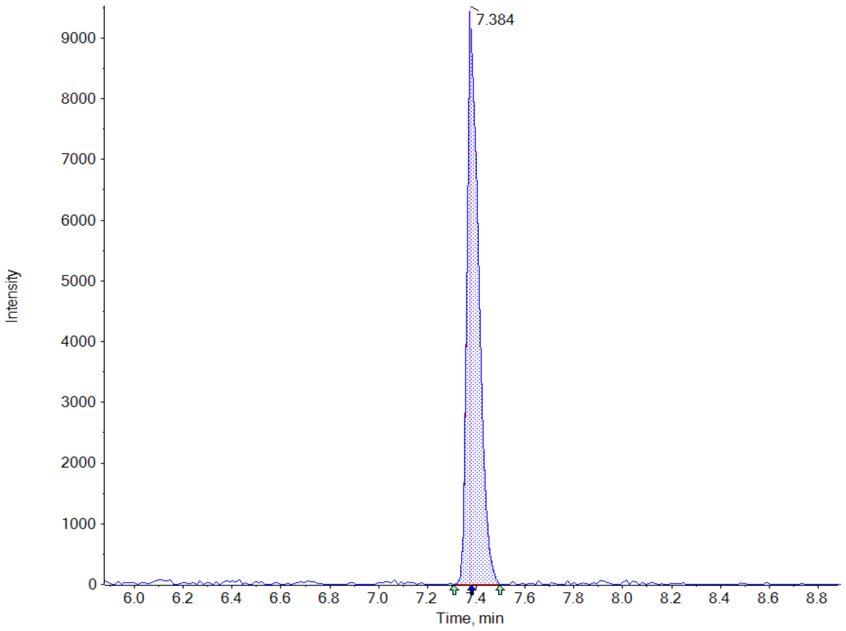

Acquired / Library MSMS

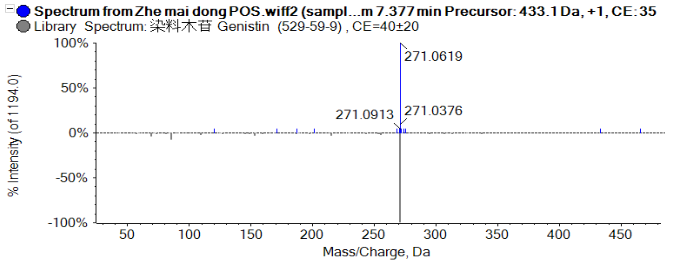

Acquired / Theoretical MS

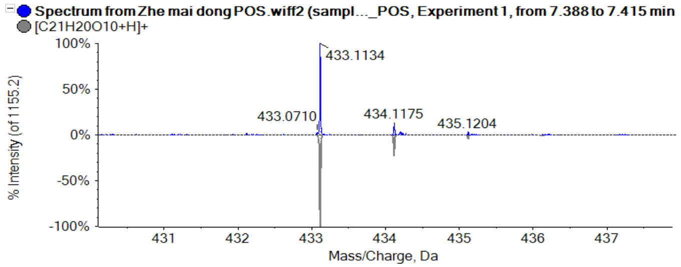

橙皮苷Hesperidin

**Retention Time:** 7.49 minutes	**Exp RT:** 7.49 minutes
**Precursor m/z :** 609.1825	**Analyte Name:**
**Fit (%)** 98.1% **RFit (%)** 100.0%	橙皮苷Hesperidin

Collision Energy = 35 ± 15 eV

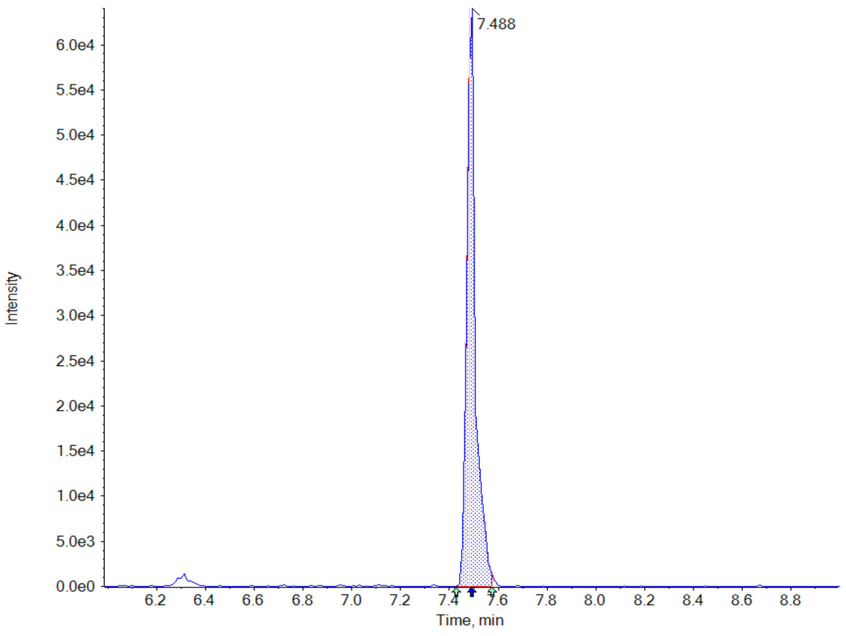

Acquired / Library MSMS

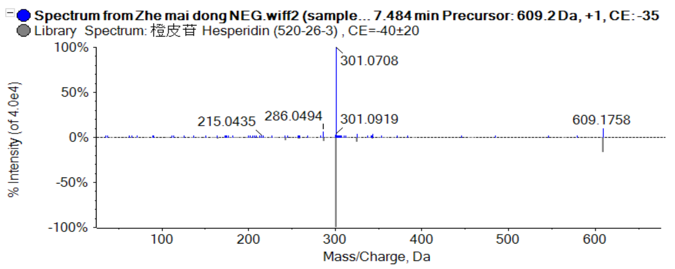

Acquired / Theoretical MS

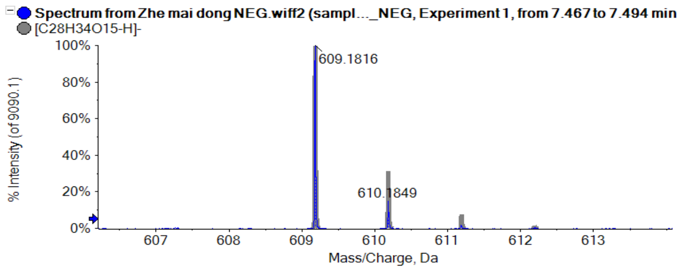

红车轴草素7-O-D-葡萄糖苷Pratensein-7-O-glucoside

Retention Time: 7.70 minutes
Precursor m/z : 463.1235
Fit (%) 94.5% **RFit (%)** 100.0%

Exp RT: 7.70 minutes
Analyte Name:
红车轴草素-7-O-D-葡萄糖苷
Pratensein-7-O-glucoside

Collision Energy = 35 ± 15 eV

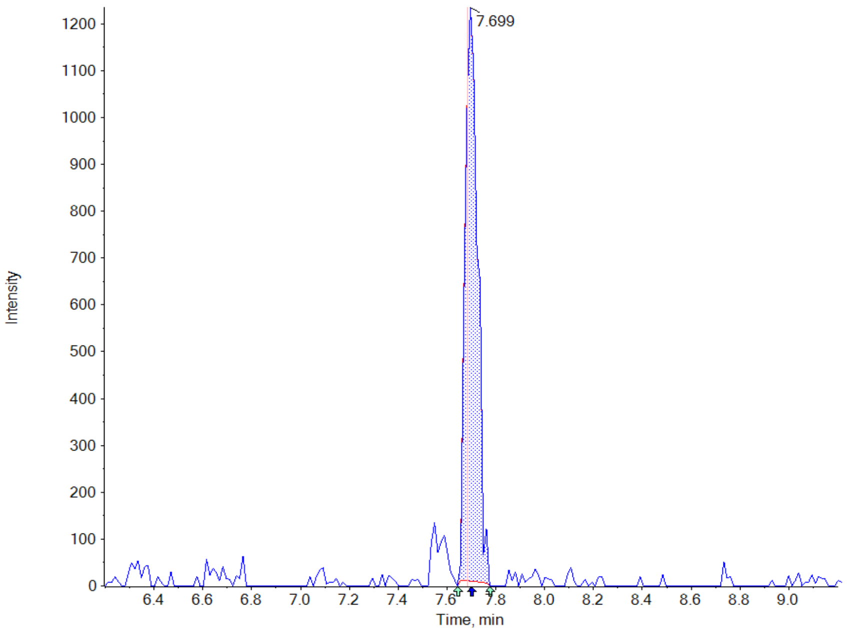

Acquired / Library MSMS

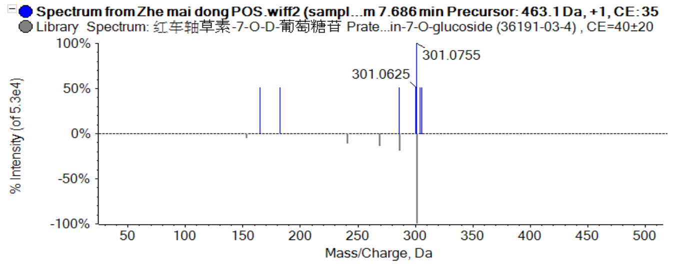

Acquired / Theoretical MS

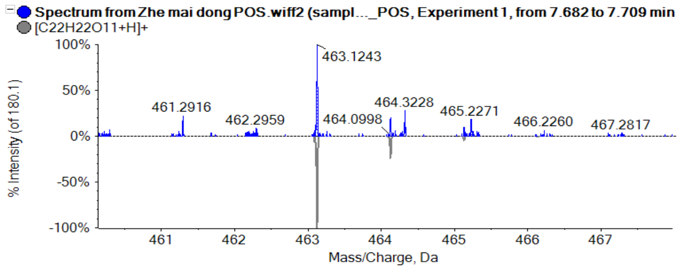

椴树苷Tiliroside

**Retention Time:** 8.91 minutes<br>**Precursor m/z :** 595.1446<br>**Fit (%)** 79.0% **RFit (%)** 100.0%	**Exp RT:** 8.90 minutes<br>**Analyte Name:**<br>椴树苷Tiliroside	
	**Collision Energy = 35 ± 15 eV**	

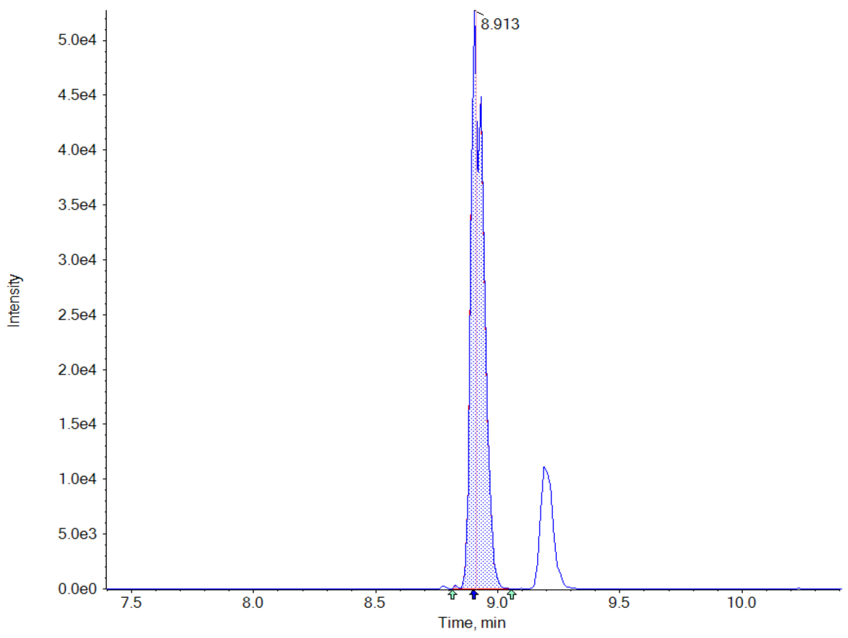

Acquired / Library MSMS

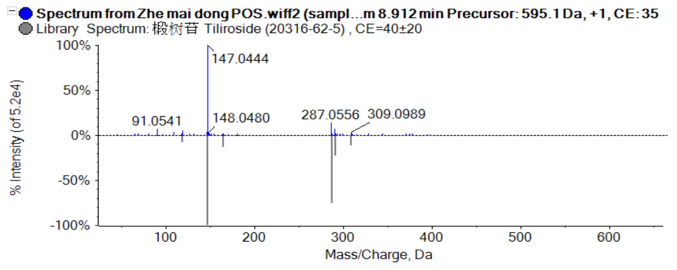

Acquired / Theoretical MS

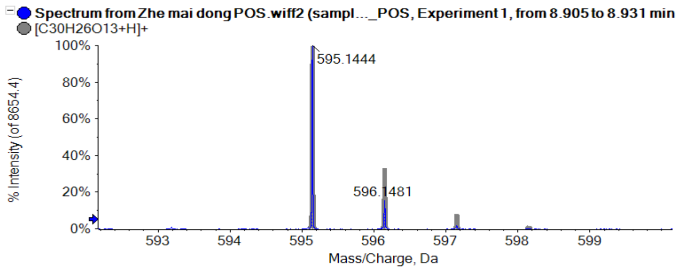

毛蕊异黄酮苷Calycosin-7-O-glucoside

Retention Time: 9.42 minutes
Precursor m/z : 447.1286
Fit (%) 98.2% **RFit (%)** 100.0%

Exp RT: 9.42 minutes
Analyte Name:
毛蕊异黄酮苷Calycosin-7-O-glucoside

Collision Energy = 35 ± 15 eV

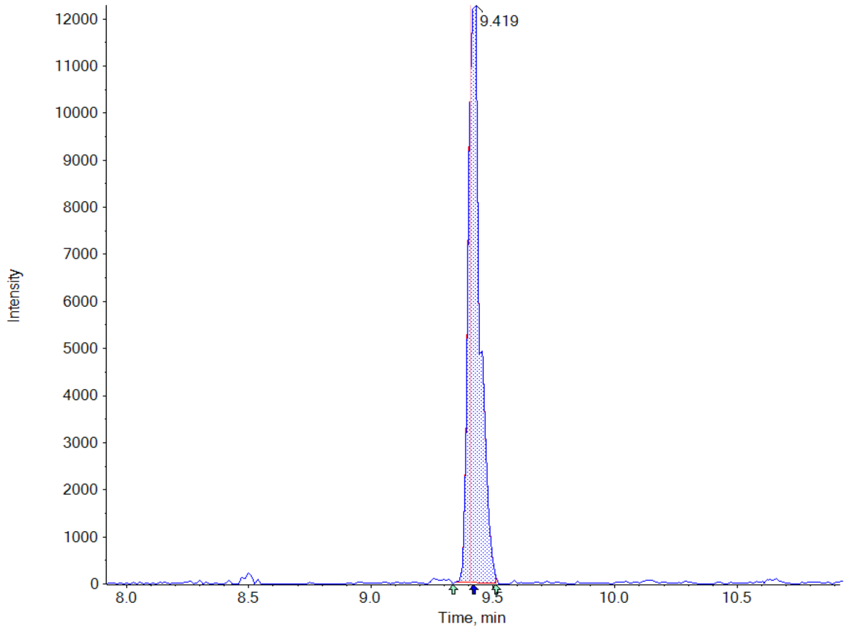

Acquired / Library MSMS

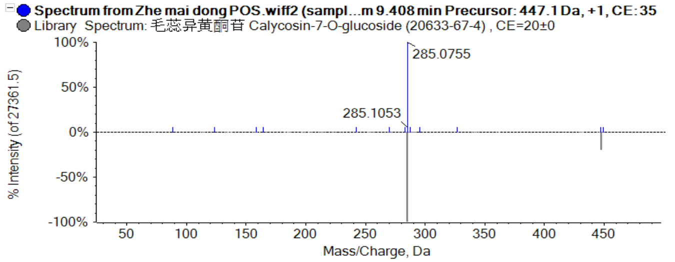

Acquired / Theoretical MS

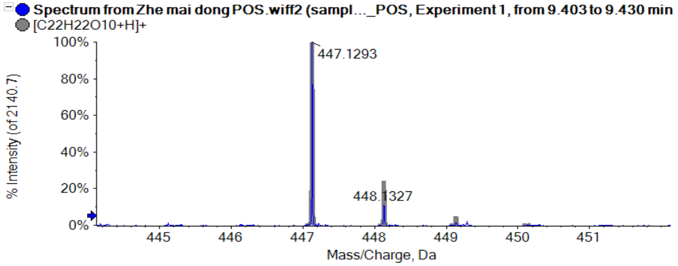

杜鹃素Farrerol

Created with SCIEX OS 1.3.1

Printed:1/11/2021 4:55:13 PM

Retention Time: 10.35 minutes **Exp RT:** 10.35 minutes
Precursor m/z : 301.1071 **Analyte Name:**
Fit (%) 76.7% **RFit (%)** 100.0% 杜鹃素Farrerol

Collision Energy = 35 ± 15 eV

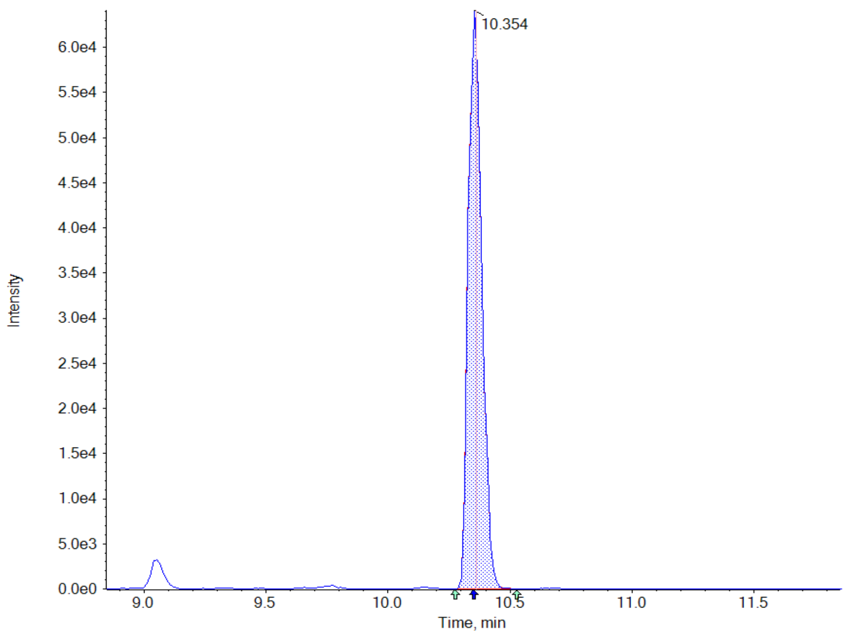

Acquired / Library MSMS

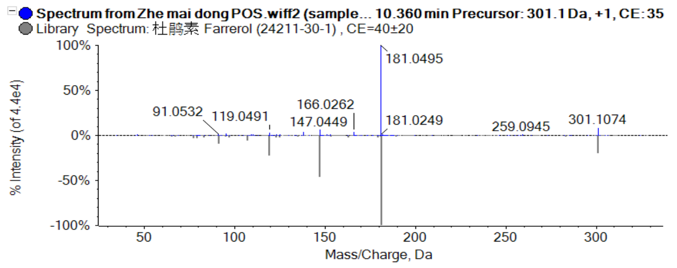

Acquired / Theoretical MS

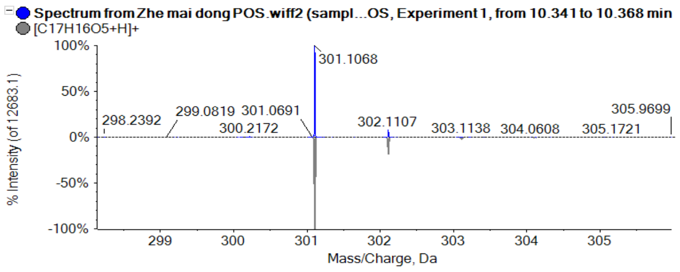

百秋李醇Patchouli alcohol

Created with SCIEX OS 1.3.1

Printed:1/11/2021 4:55:13 PM

Retention Time: 12.24 minutes **Exp RT:** 12.25 minutes

Precursor m/z : 205.1951 **Analyte Name:**

Fit (%) 97.6% **RFit (%)** 99.5% 百秋李醇Patchouli alcohol (loss H20)

Collision Energy = 35 ± 15 eV

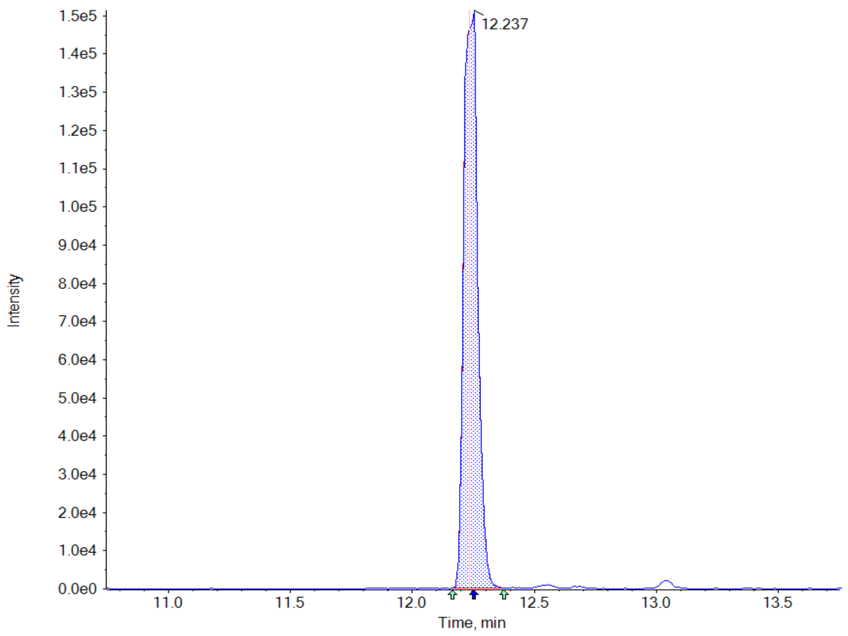

Acquired / Library MSMS

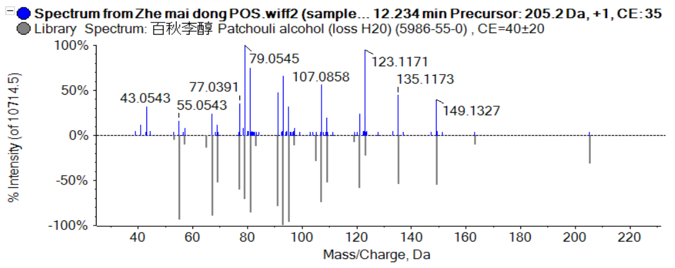

Acquired / Theoretical MS

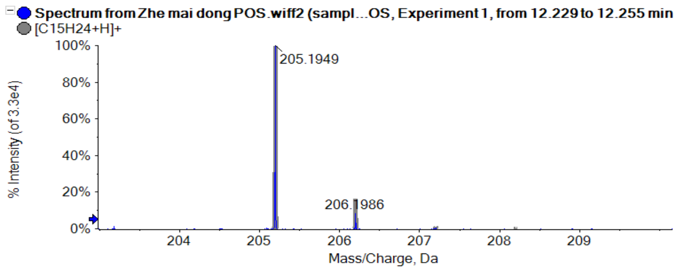

川陈皮素Nobiletin

Retention Time: 12.77 minutes
Precursor m/z : 403.1387
Fit (%) 98.1% **RFit (%)** 98.6%

Exp RT: 12.77 minutes
Analyte Name:
川陈皮素Nobiletin

Collision Energy = 35 ± 15 eV

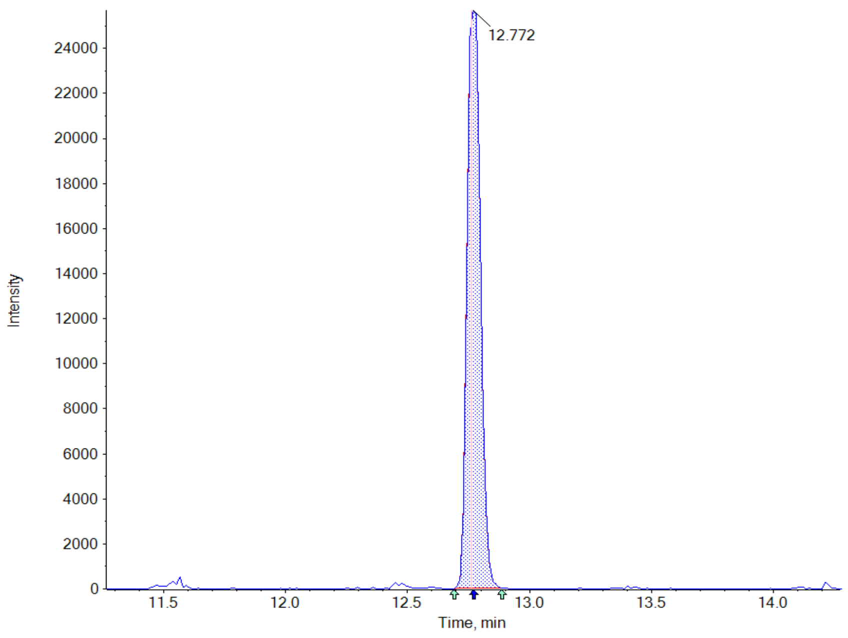

Acquired / Library MSMS

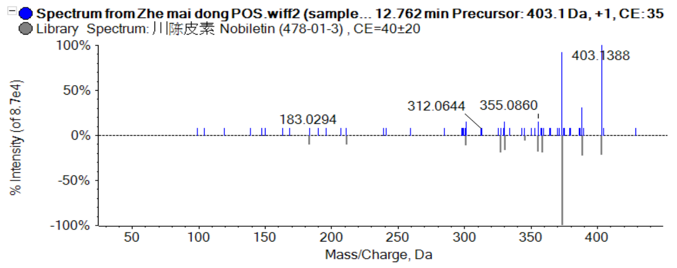

Acquired / Theoretical MS

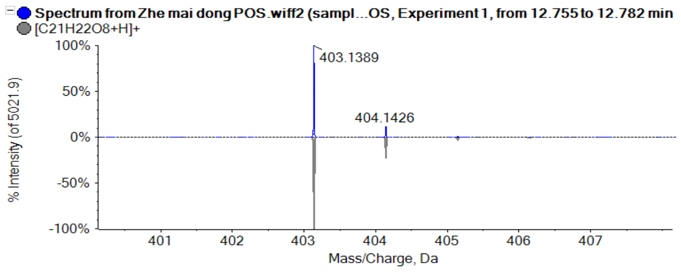

薯蓣皂苷元Diosgenin

Retention Time: 14.75 minutes
Precursor m/z : 415.3207
Fit (%) 89.4% **RFit (%)** 98.7%

Exp RT: 14.75 minutes
Analyte Name:
薯蓣皂苷元Diosgenin

Collision Energy = 35 ± 15 eV

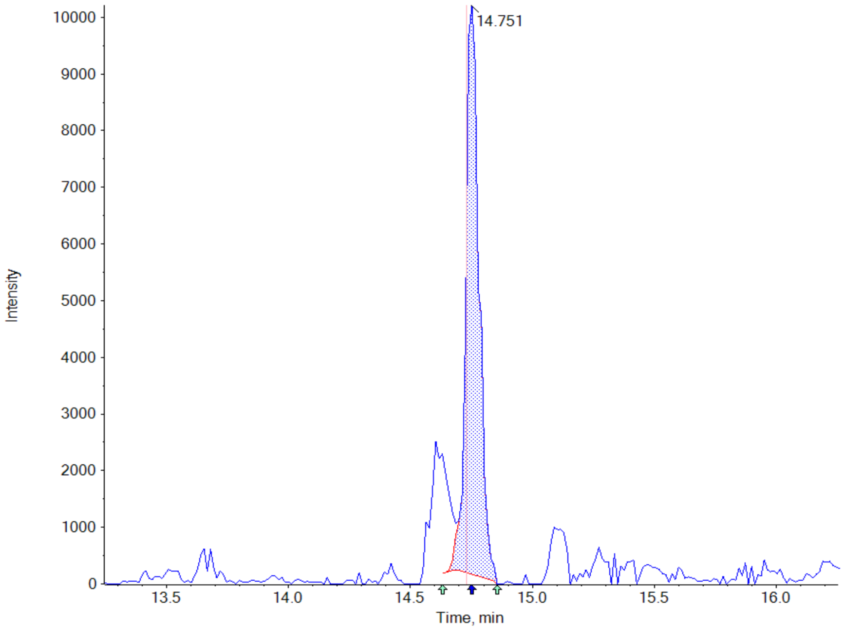

Acquired / Library MSMS

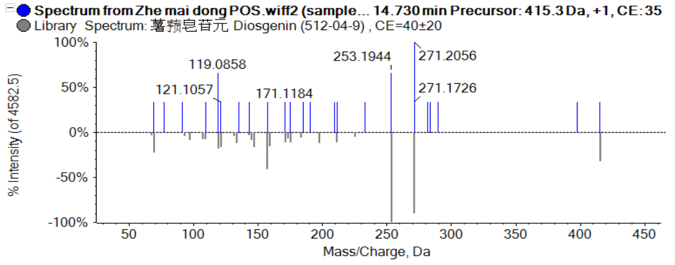

Acquired / Theoretical MS

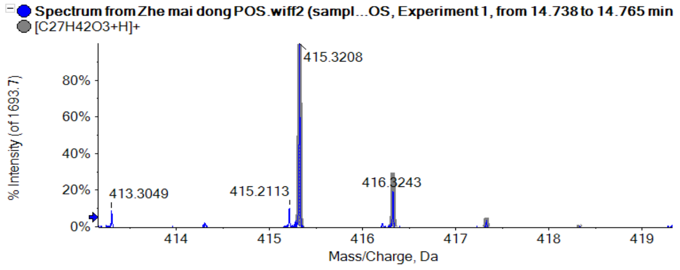

鲁斯可皂苷元Ruscogenin

Retention Time: 14.83 minutes **Exp RT:** 14.85 minutes
Precursor m/z : 431.3156 **Analyte Name:**
Fit (%) 70.5% **RFit (%)** 100.0% 鲁斯可皂苷元Ruscogenin

Collision Energy = 35 ± 15 eV

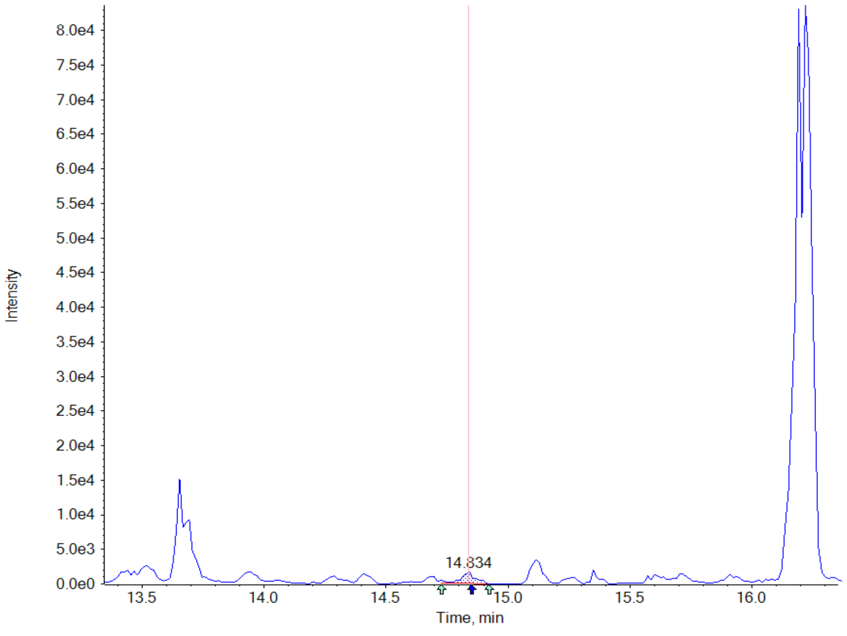

Acquired / Library MSMS

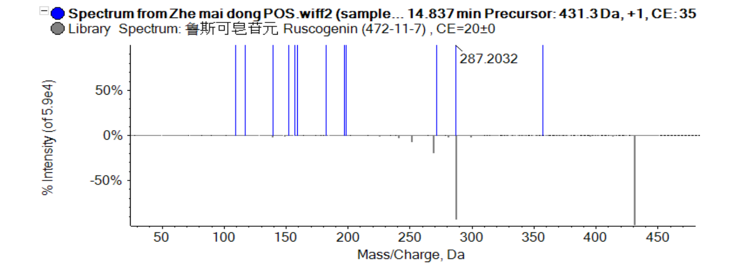

Acquired / Theoretical MS

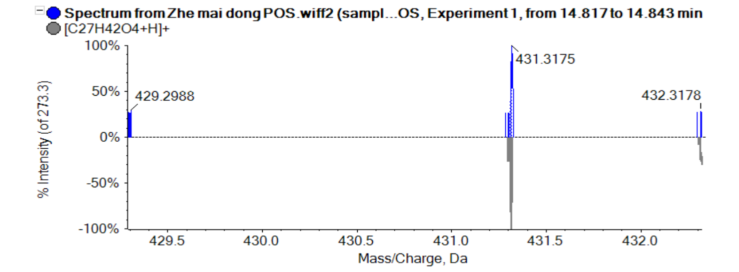

山麦冬皂苷B Liriopesides B

Retention Time: 15.11 minutes **Exp RT:** 15.12 minutes
Precursor m/z : 723.4314 **Analyte Name:**
Fit (%) 92.4% **RFit (%)** 98.1% 山麦冬皂苷B Liriopesides B

Collision Energy = 35 ± 15 eV

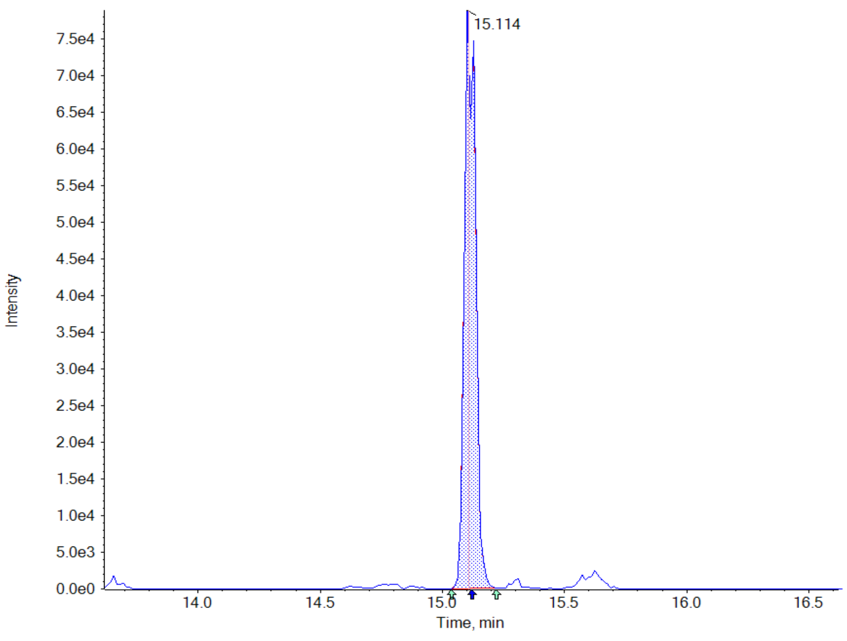

Acquired / Library MSMS

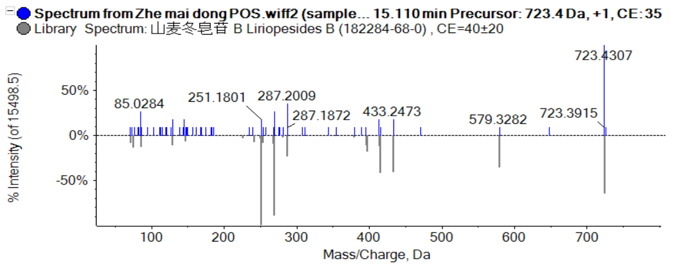

Acquired / Theoretical MS

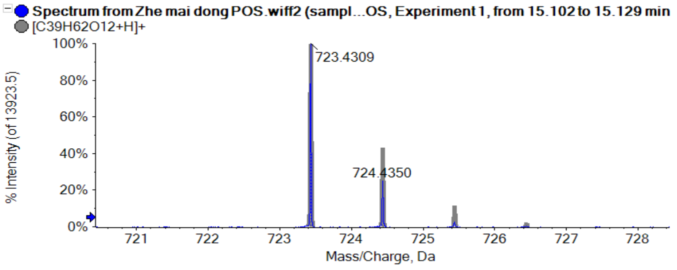

MS fragmentation pathways for different chemical constituents of ZMD in negative ion mode

组氨酸Histidine

Retention Time: 1.08 minutes
Precursor m/z : 154.0622
Fit (%) 84.0% **RFit (%)** 100.0%

Exp RT: 1.09 minutes
Analyte Name: 组氨酸Histidine

Collision Energy = 35 ± 15 eV

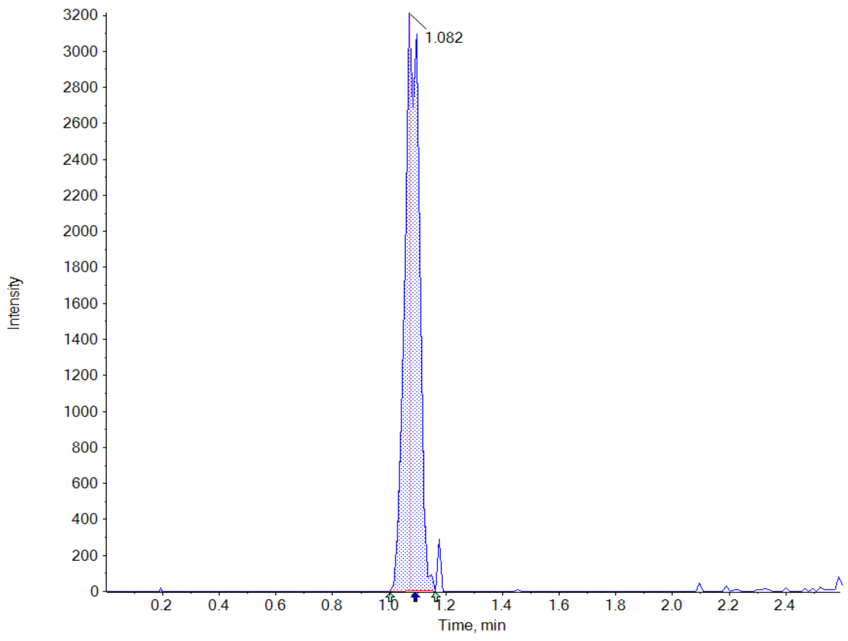

Acquired / Library MSMS

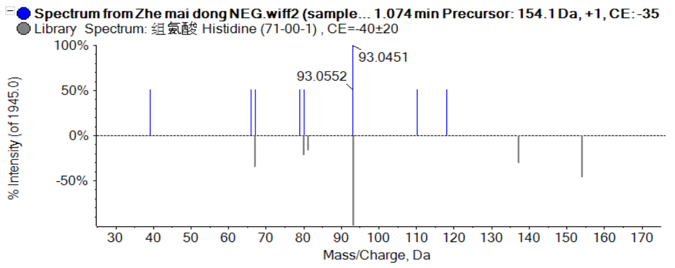

Acquired / Theoretical MS

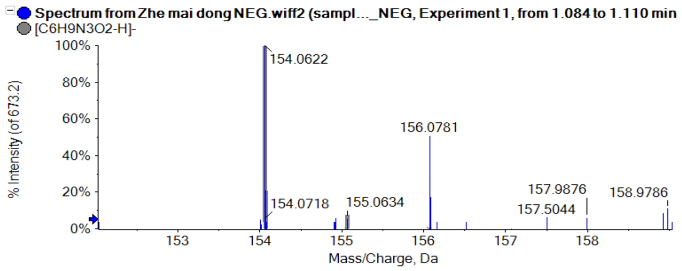

精氨酸L(+)-Arginine

Retention Time: 1.09 minutes
Precursor m/z : 173.1044
Fit (%) 100.0% **RFit (%)** 98.9%

Exp RT: 1.09 minutes
Analyte Name:
精氨酸L(+)-Arginine

Collision Energy = 35 ± 15 eV

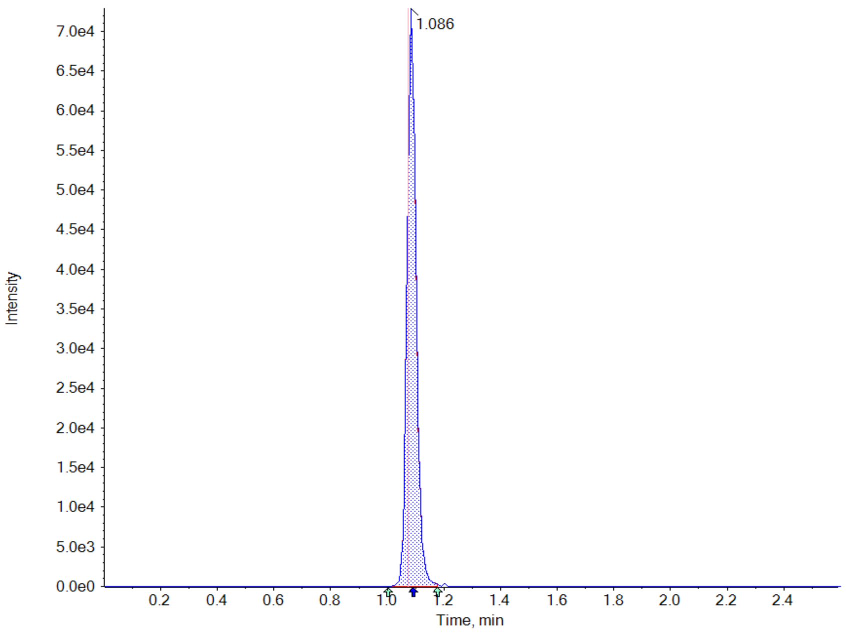

Acquired / Library MSMS

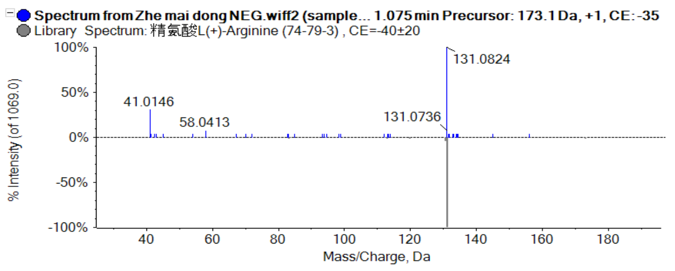

Acquired / Theoretical MS

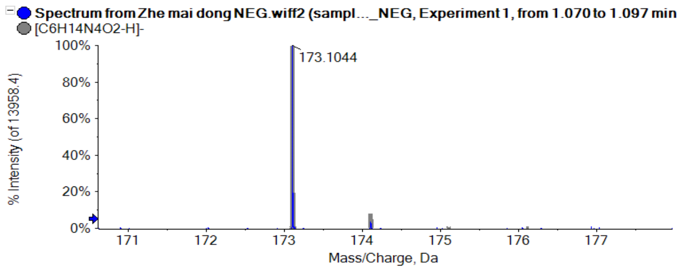

D-甘露糖D-(+)-Mannose

Retention Time: 1.19 minutes
Precursor m/z : 179.0561
Fit (%) 97.6% **RFit (%)** 95.6%

Exp RT: 1.21 minutes
Analyte Name:
D-甘露糖D-(+)-Mannose

Collision Energy = 35 ± 15 eV

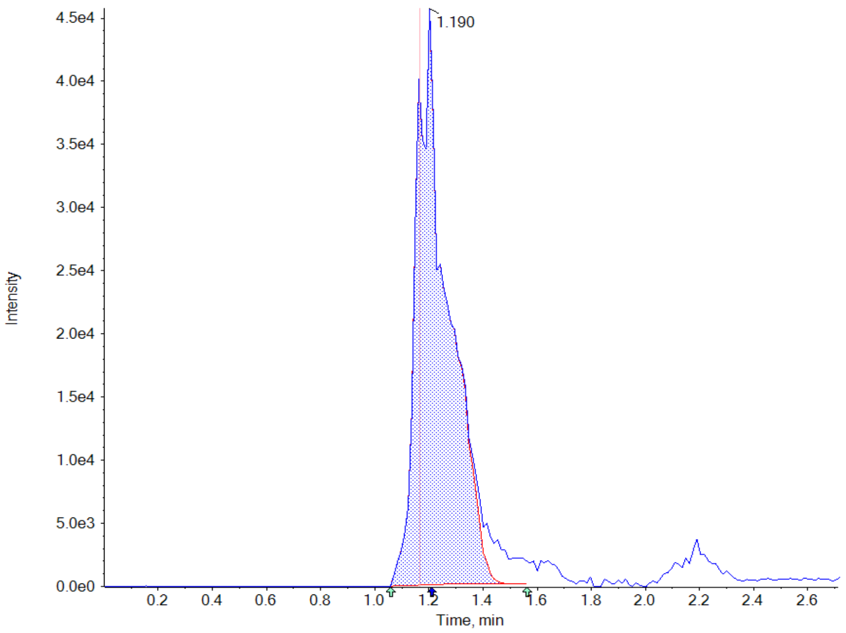

Acquired / Library MSMS

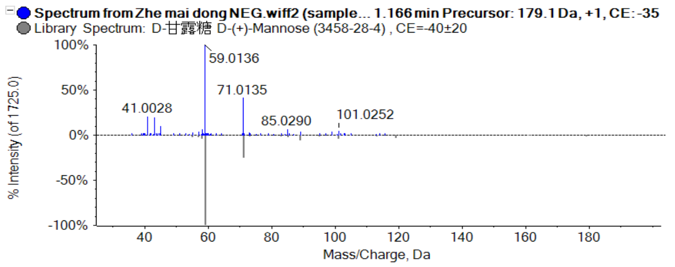

Acquired / Theoretical MS

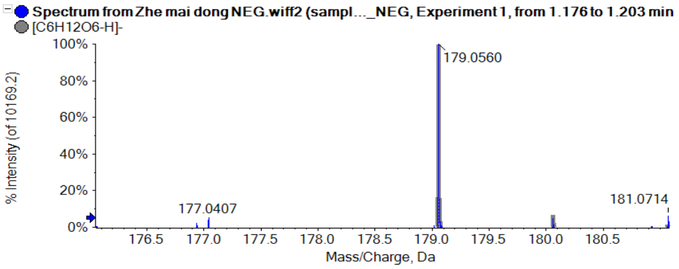

L-苹果酸L-Malic acid

**Retention Time:** 1.35 minutes<br>**Precursor m/z :** 133.0142<br>**Fit (%)** 95.6% **RFit (%)** 86.3%	**Exp RT:** 1.34 minutes<br>**Analyte Name:**<br>L-苹果酸L-Malic acid

	Collision Energy = 35 ± 15 eV	
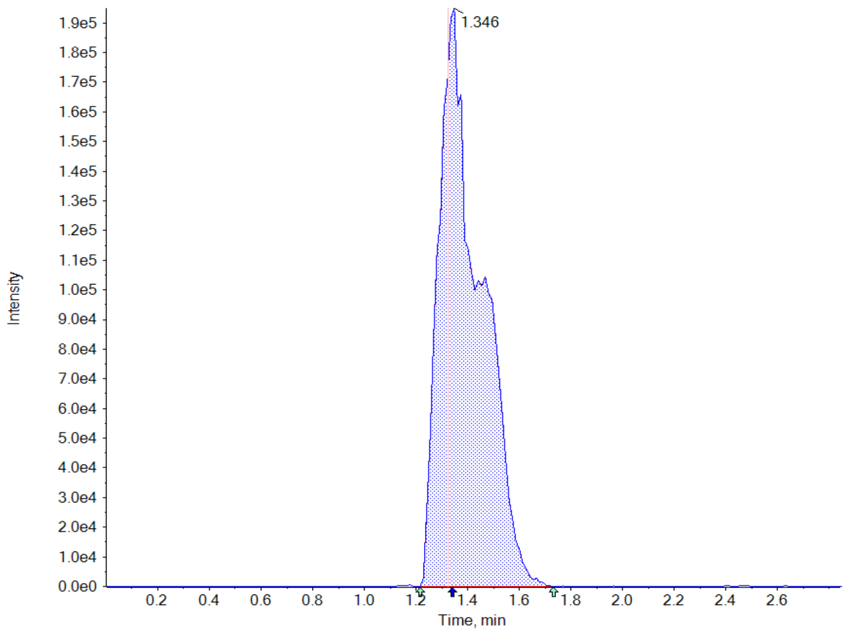	**Acquired / Library MSMS**	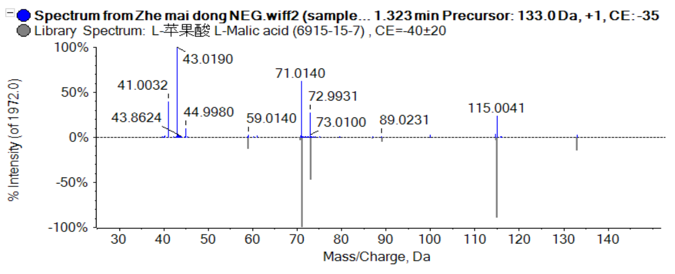
	**Acquired / Theoretical MS**	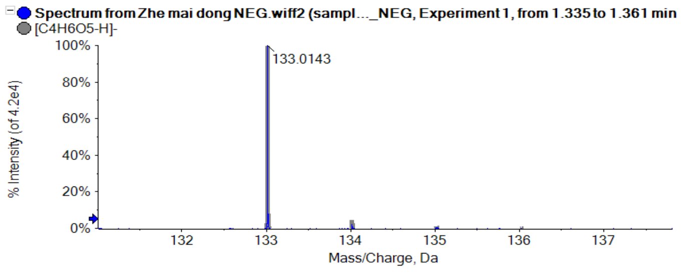

耐斯糖Fungitetraose

**Retention Time:** 1.79 minutes	**Exp RT:** 1.74 minutes
**Precursor m/z :** 665.2146	**Analyte Name:**
**Fit (%)** 93.1% **RFit (%)** 89.4%	耐斯糖Fungitetraose

Collision Energy = 35 ± 15 eV

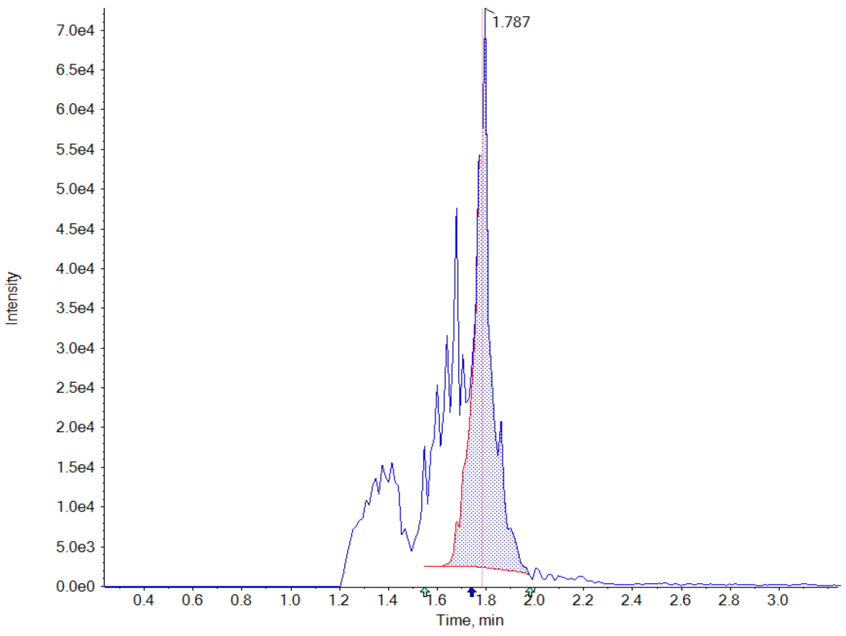

Acquired / Library MSMS

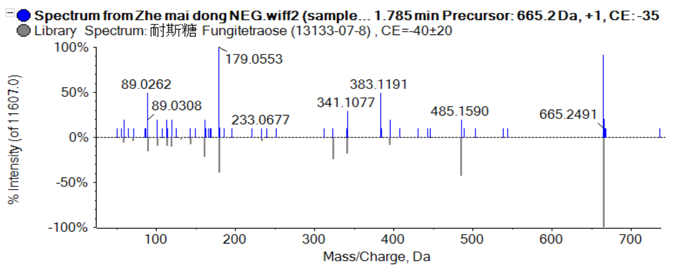

Acquired / Theoretical MS

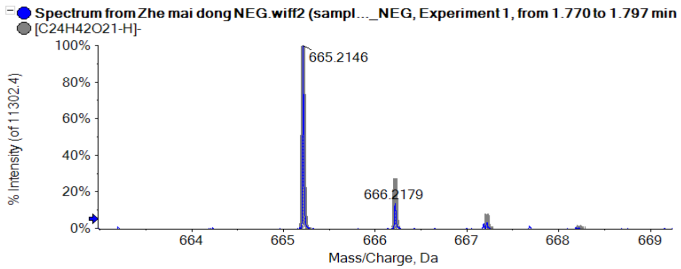

柠檬酸Citric acid

Retention Time: 1.94 minutes
Precursor m/z : 191.0197
Fit (%) 99.2% **RFit (%)** 99.7%

Exp RT: 1.94 minutes
Analyte Name:
柠檬酸Citric acid

Collision Energy = 35 ± 15 eV

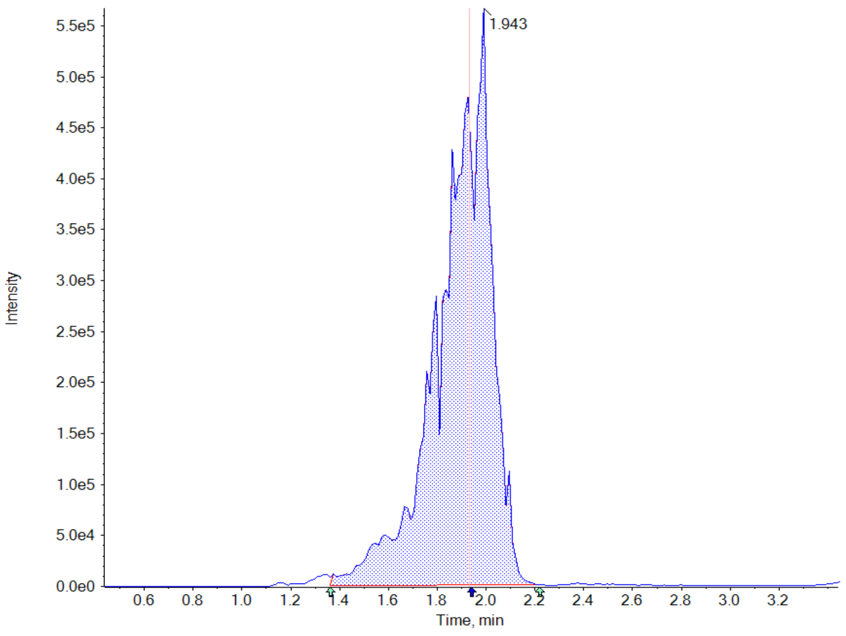

Acquired / Library MSMS

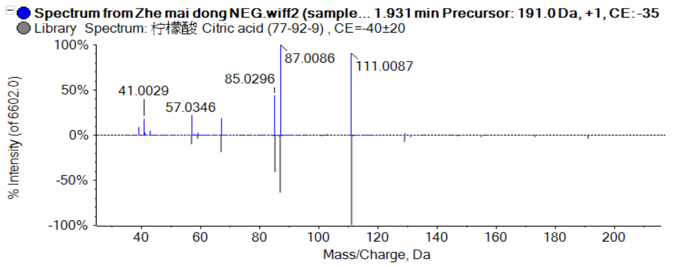

Acquired / Theoretical MS

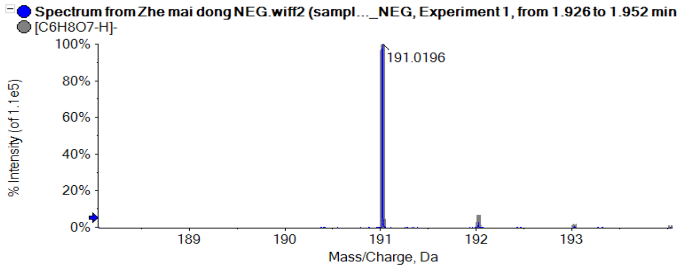

琥珀酸Succinic acid

Retention Time: 2.31 minutes

Precursor m/z : 117.0193

Fit (%) 100.0% **RFit (%)** 95.7%

Exp RT: 2.30 minutes

Analyte Name: 琥珀酸Succinic acid

Collision Energy = 35 ± 15 eV

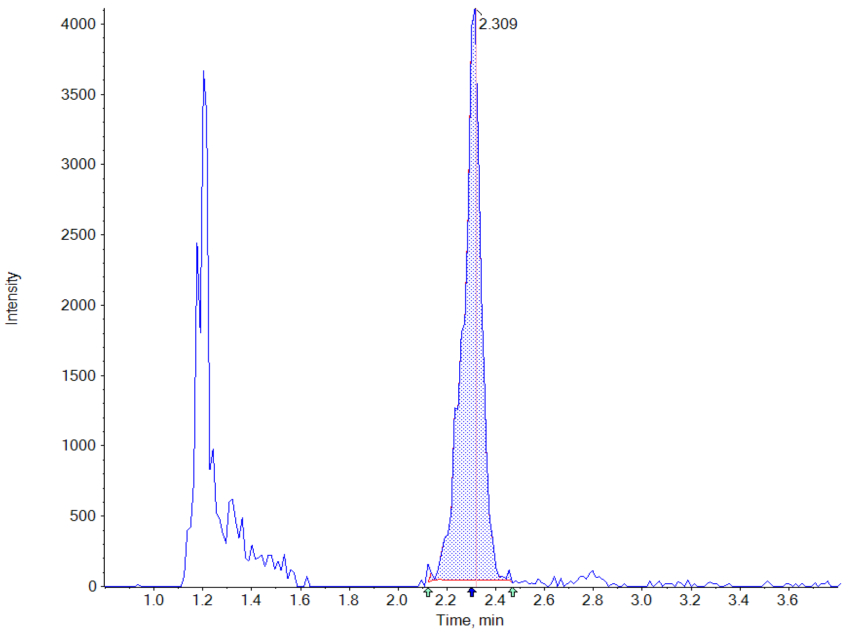

Acquired / Library MSMS

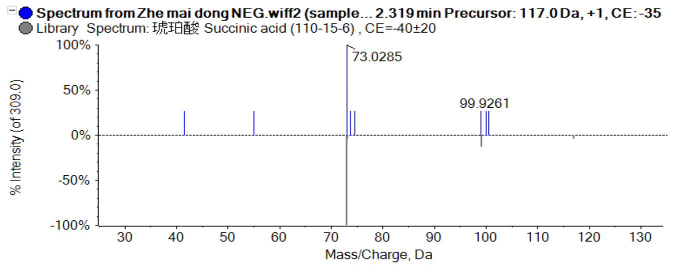

Acquired / Theoretical MS

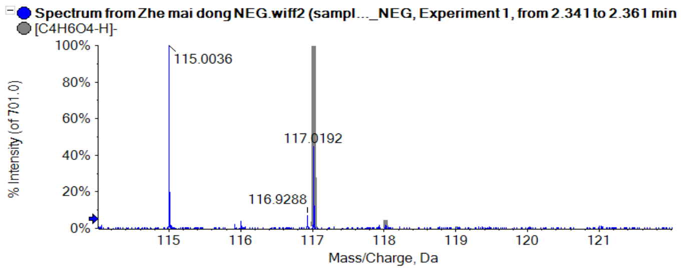

腺嘌呤Adenine

Retention Time: 2.37 minutes **Exp RT:** 2.37 minutes
Precursor m/z : 134.0472 **Analyte Name:**
Fit (%) 86.3% **RFit (%)** 100.0% 腺嘌呤Adenine

Collision Energy = 35 ± 15 eV

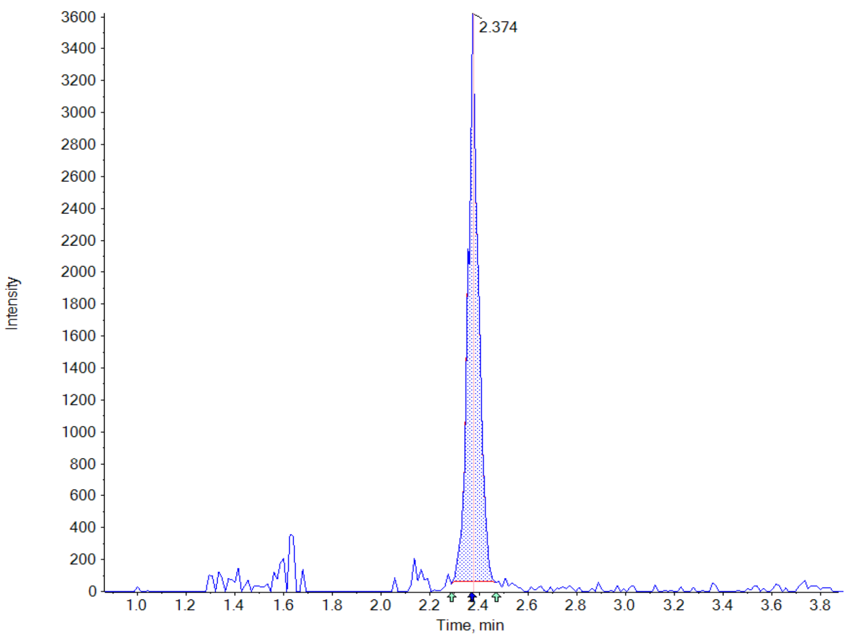

Acquired / Library MSMS

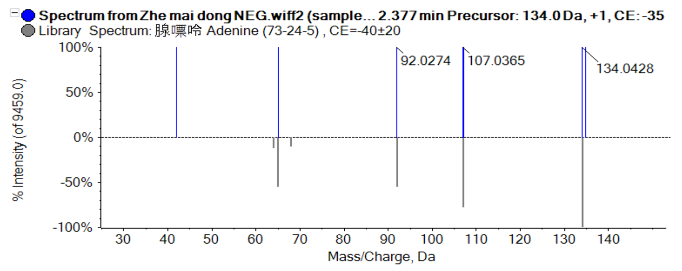

Acquired / Theoretical MS

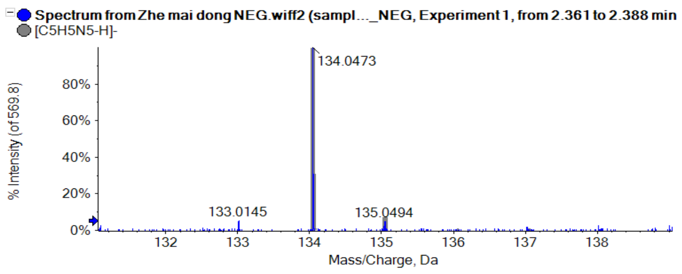

鸟苷Guanosine

Retention Time: 2.48 minutes **Exp RT:** 2.48 minutes

Precursor m/z : 282.0844 **Analyte Name:**

Fit (%) 99.3% **RFit (%)** 100.0% 鸟苷Guanosine

Collision Energy = 35 ± 15 eV

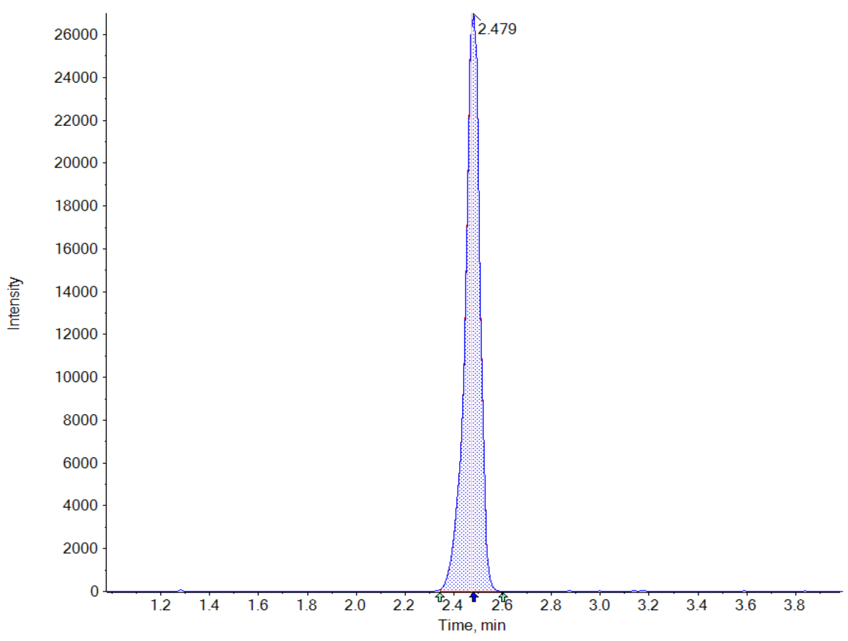

Acquired / Library MSMS

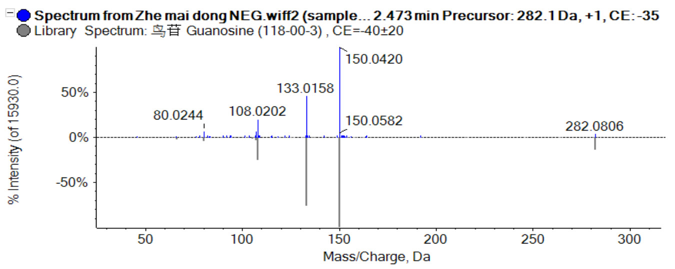

Acquired / Theoretical MS

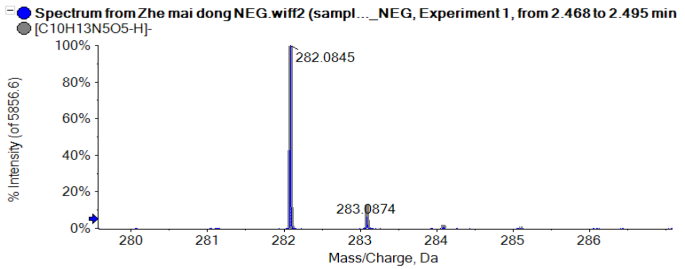

Created with SCIEX OS 1.3.1

Printed:1/11/2021 4:55:13 PM

没食子酸Gallic acid

Retention Time: 2.66 minutes **Exp RT:** 2.66 minutes
Precursor m/z : 169.0142 **Analyte Name:**
Fit (%) 94.2% **RFit (%)** 98.4% 没食子酸Gallic acid

Collision Energy = 35 ± 15 eV

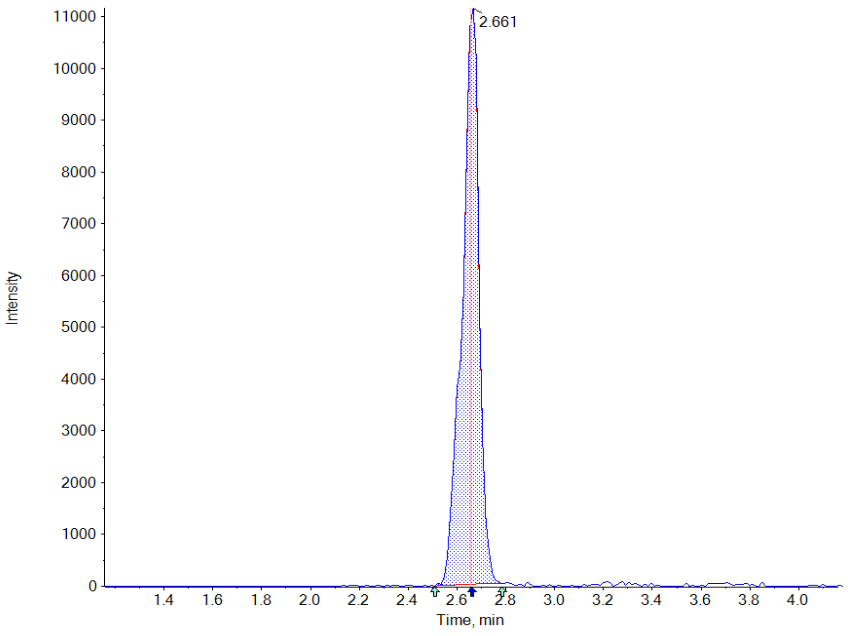

Acquired / Library MSMS

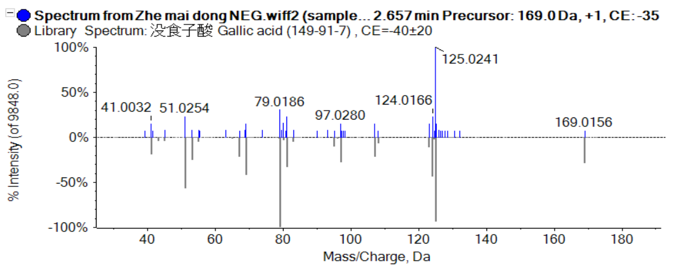

Acquired / Theoretical MS

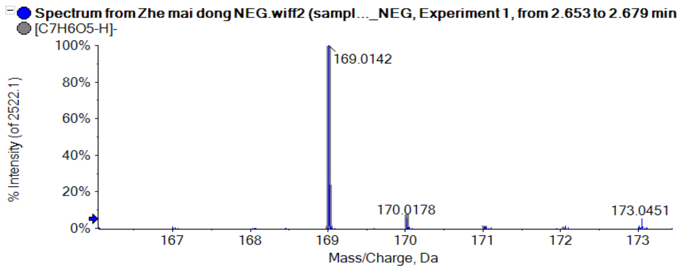

苯丙氨酸Phenprobamate

**Retention Time:** 3.19 minutes	**Exp RT:** 3.18 minutes
**Precursor m/z :** 164.0717	**Analyte Name:**
**Fit (%)** 98.2% **RFit (%)** 95.5%	苯丙氨酸Phenprobamate

Collision Energy = 35 ± 15 eV

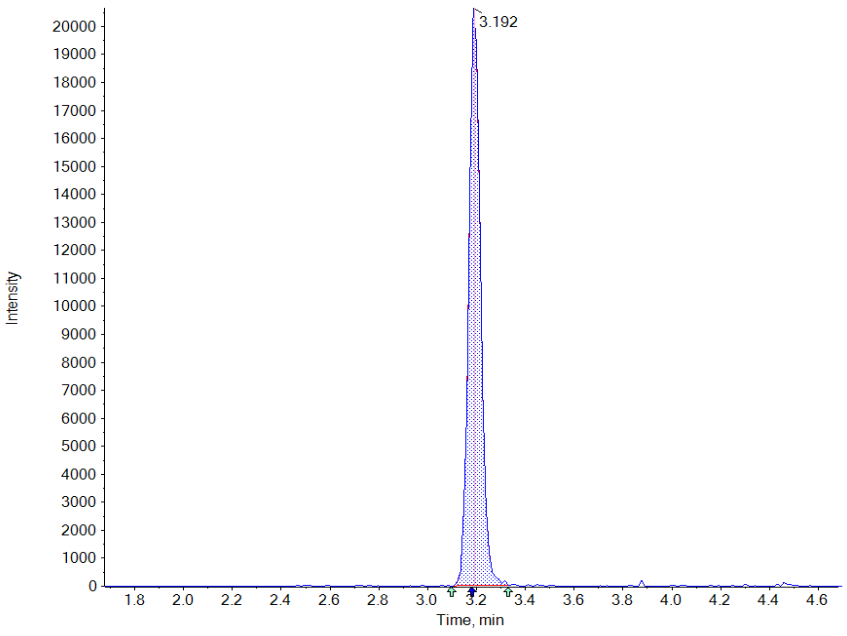

Acquired / Library MSMS

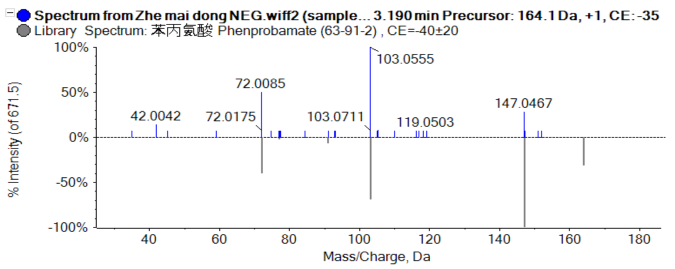

Acquired / Theoretical MS

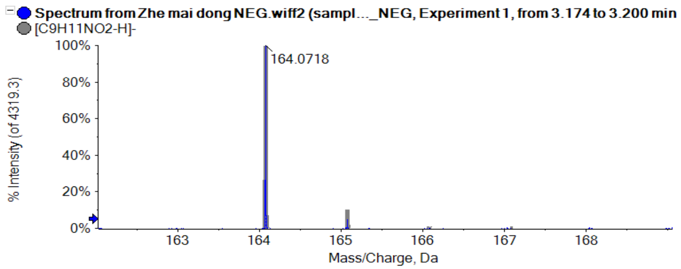

香草酸Vanillic acid

Retention Time: 3.37 minutes **Exp RT:** 3.37 minutes
Precursor m/z : 167.0350 **Analyte Name:**
Fit (%) 71.6% **RFit (%)** 100.0% 香草酸Vanillic acid

Collision Energy = 35 ± 15 eV

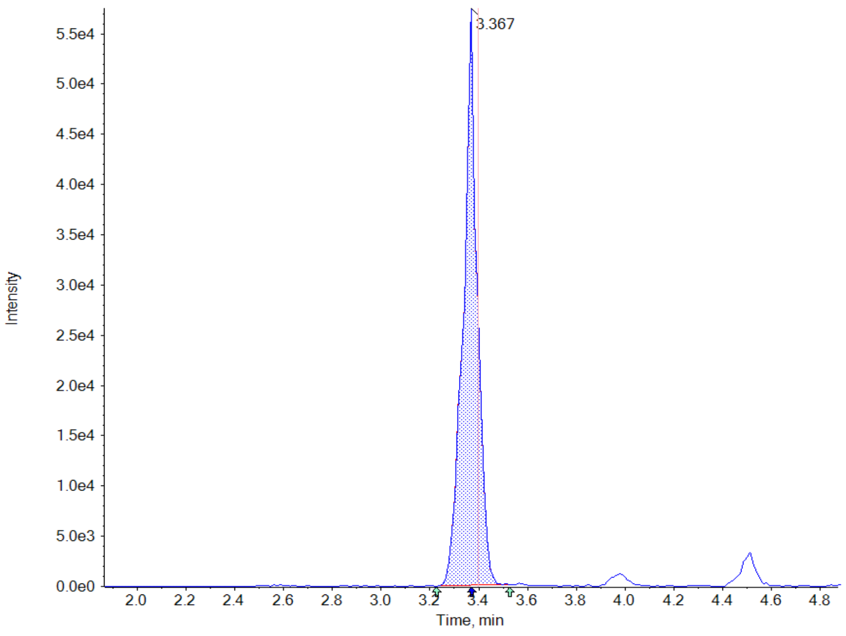

Acquired / Library MSMS

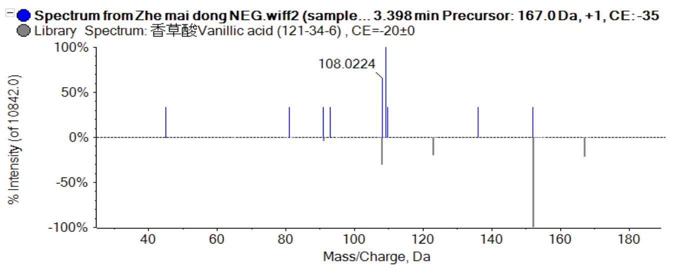

Acquired / Theoretical MS

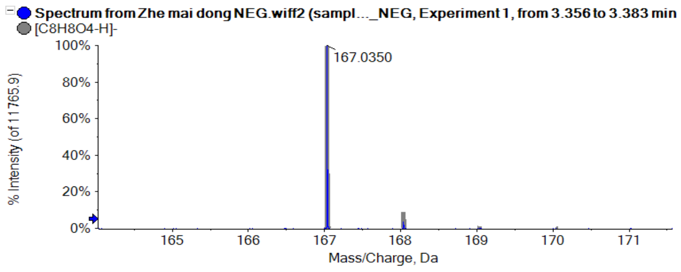

Created with SCIEX OS 1.3.1

Printed:1/11/2021 4:55:13 PM

羟基酪醇Hydroxytyrosol

Retention Time: 3.69 minutes
Precursor m/z : 153.0557
Fit (%) 93.1% **RFit (%)** 100.0%

Exp RT: 3.69 minutes
Analyte Name: 羟基酪醇Hydroxytyrosol

Collision Energy = 35 ± 15 eV

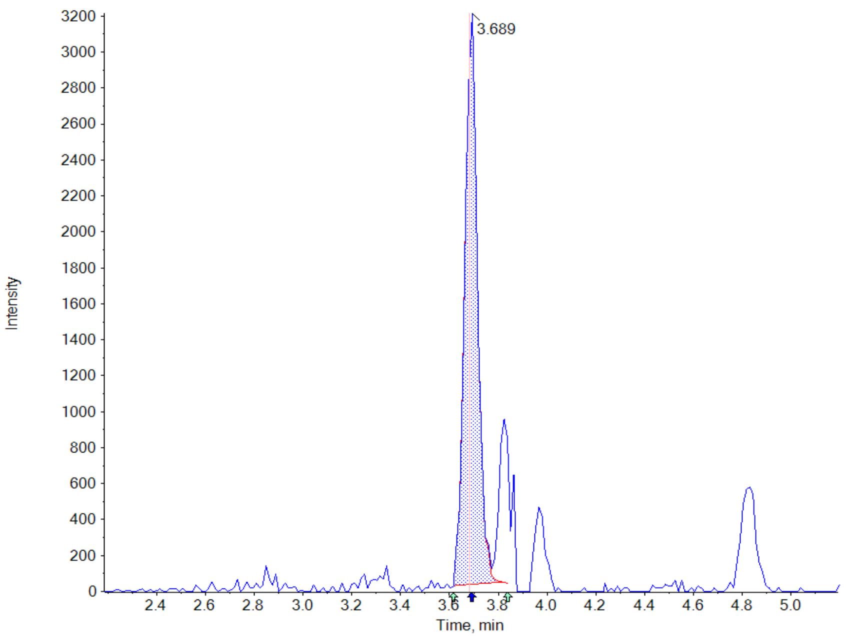

Acquired / Library MSMS

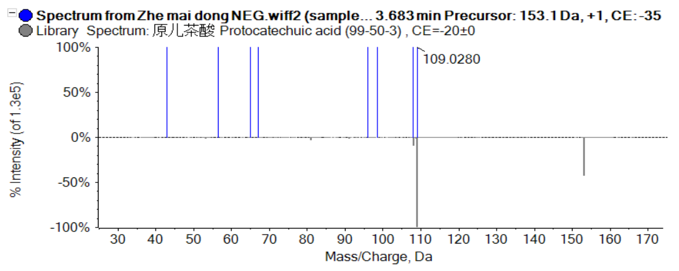

Acquired / Theoretical MS

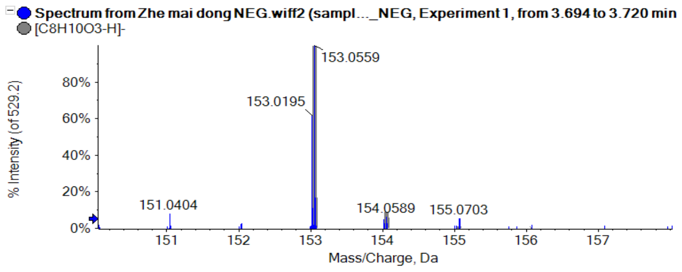

色氨酸L-Tryptophan

Retention Time: 4.16 minutes
Precursor m/z : 203.0826
Fit (%) 97.2% **RFit (%)** 96.2%

Exp RT: 4.16 minutes
Analyte Name:
色氨酸L-Tryptophan

Collision Energy = 35 ± 15 eV

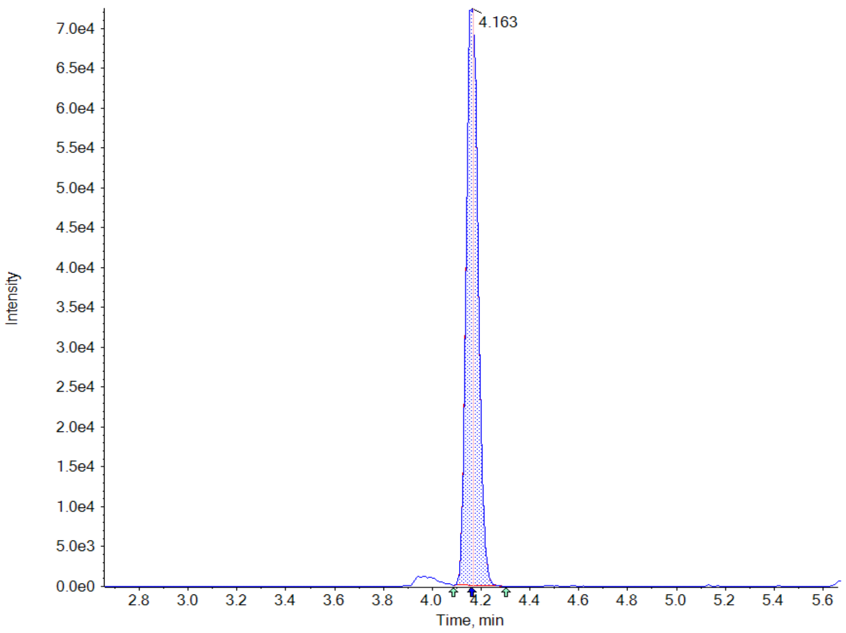

Acquired / Library MSMS

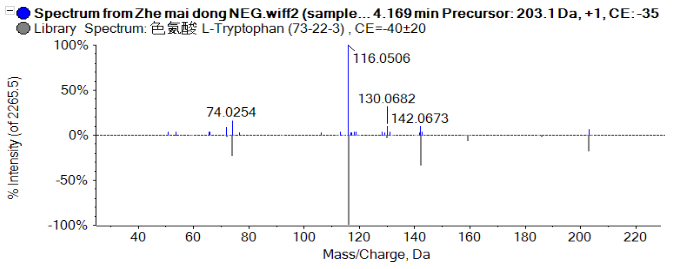

Acquired / Theoretical MS

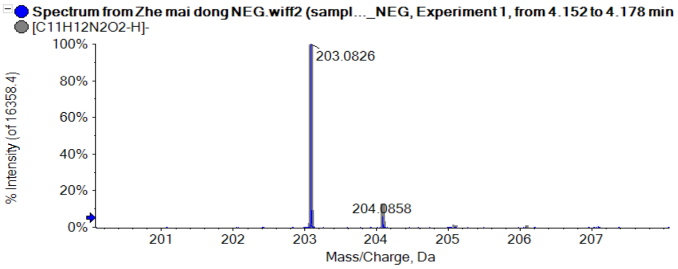

红景天苷Salidroside

Retention Time: 4.17 minutes
Precursor m/z : 299.1136
Fit (%) 90.1% **RFit (%)** 99.5%

Exp RT: 4.18 minutes
Analyte Name:
红景天苷Salidroside

Collision Energy = 35 ± 15 eV

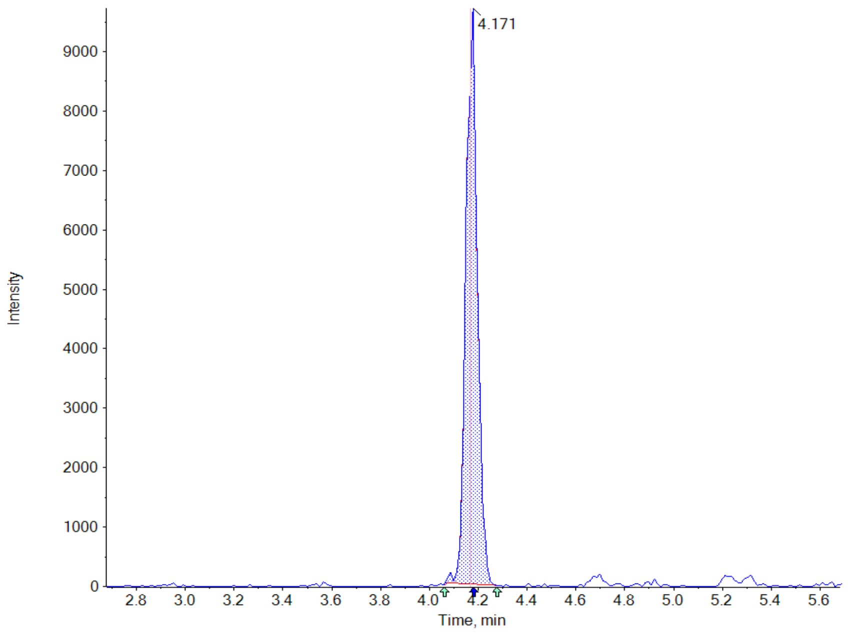

Acquired / Library MSMS

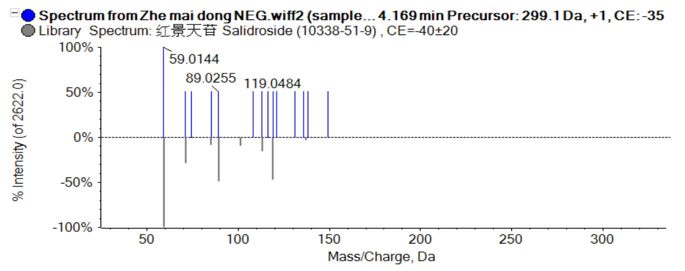

Acquired / Theoretical MS

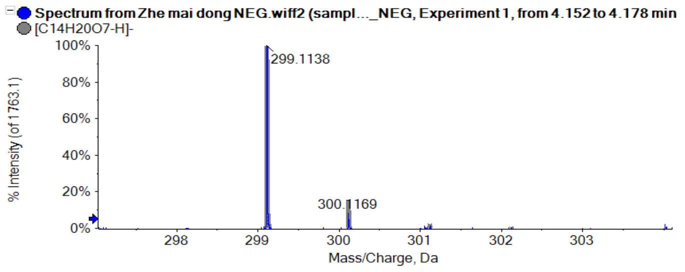

对羟基苯甲酸4-Hydroxybenzoic acid

Retention Time: 4.63 minutes **Exp RT:** 4.63 minutes

Precursor m/z : 137.0244 **Analyte Name:**

Fit (%) 99.1% **RFit (%)** 99.0% 对羟基苯甲酸4-Hydroxybenzoic acid

Collision Energy = 35 ± 15 eV

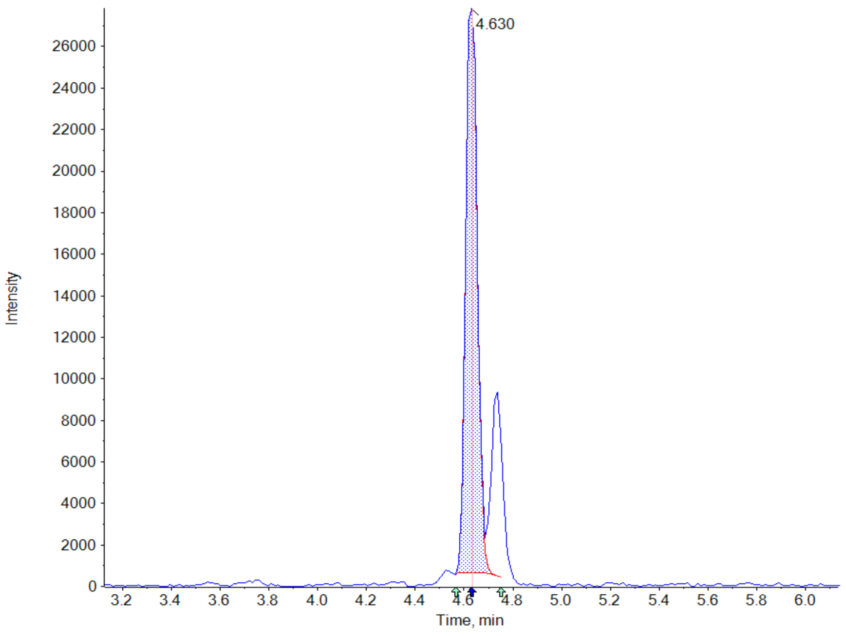

Acquired / Library MSMS

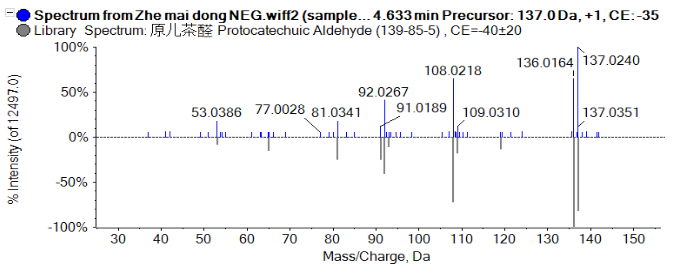

Acquired / Theoretical MS

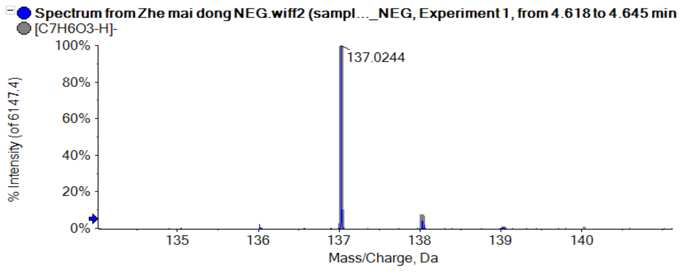

隐绿原酸(5-咖啡酰奎宁酸) 4-O-Caffeoyl Quinic acid

Retention Time: 4.80 minutes
Precursor m/z : 353.0878
Fit (%) 95.8% **RFit (%)** 95.5%

Exp RT: 4.80 minutes
Analyte Name:
隐绿原酸(5-咖啡酰奎宁酸) 4-O-Caffeoyl Quinic acid

Collision Energy = 35 ± 15 eV

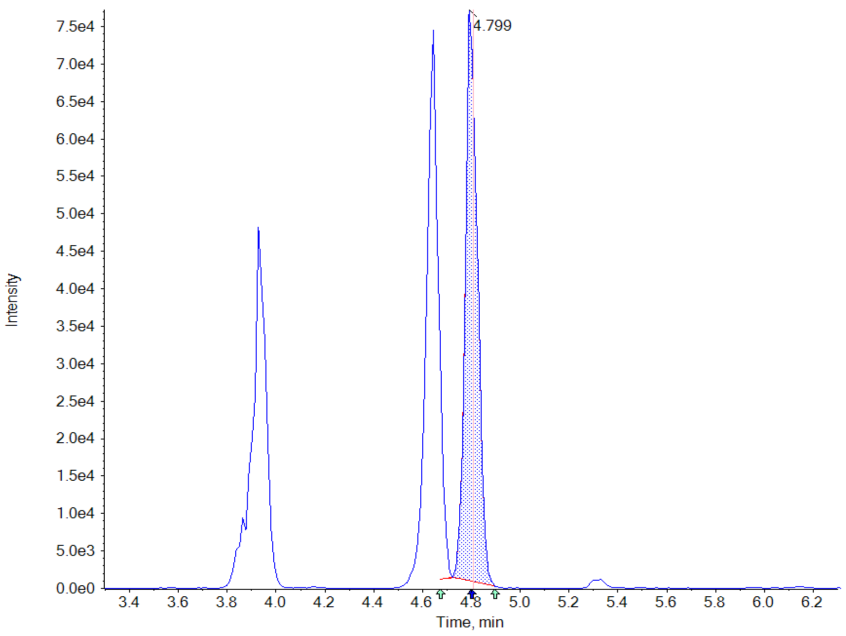

Acquired / Library MSMS

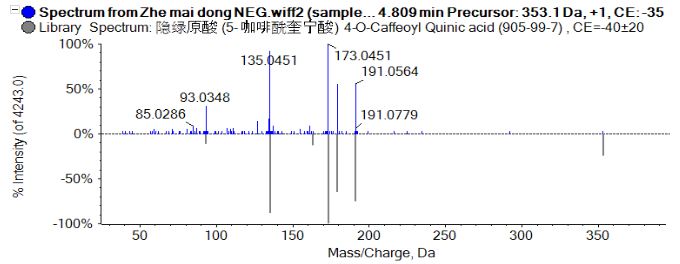

Acquired / Theoretical MS

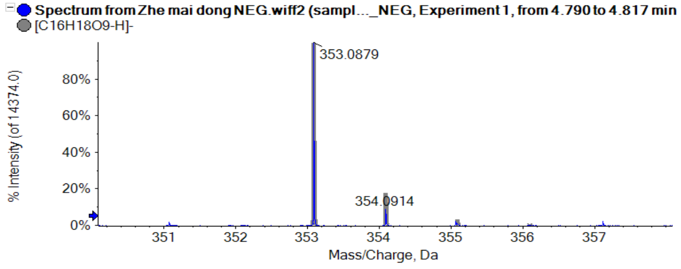

秦皮乙素Esculetin

**Retention Time:** 5.14 minutes	**Exp RT:** 5.14 minutes
**Precursor m/z :** 177.0193	**Analyte Name:**
**Fit (%)** 96.0% **RFit (%)** 99.9%	秦皮乙素Esculetin

Collision Energy = 35 ± 15 eV

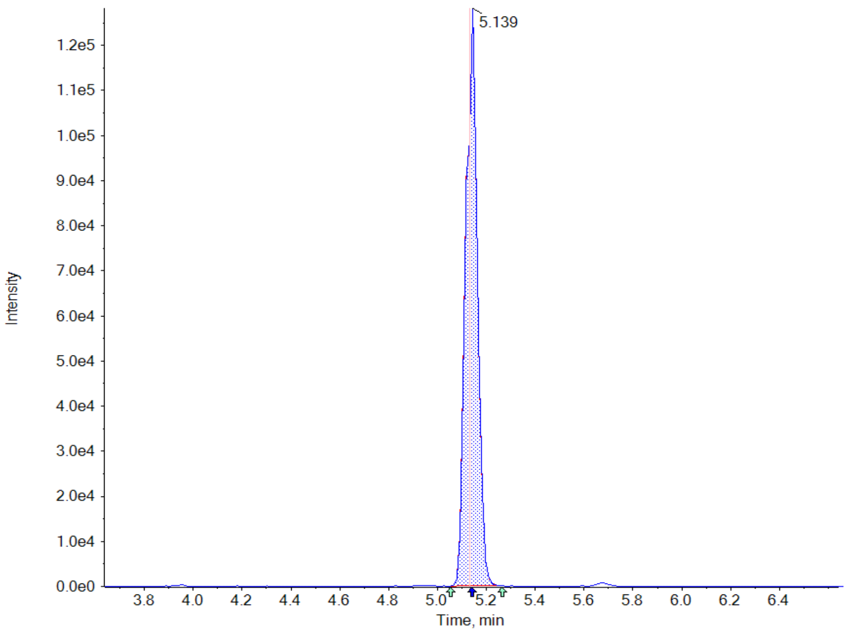

Acquired / Library MSMS

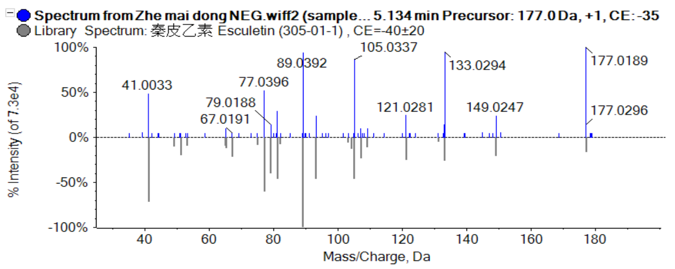

Acquired / Theoretical MS

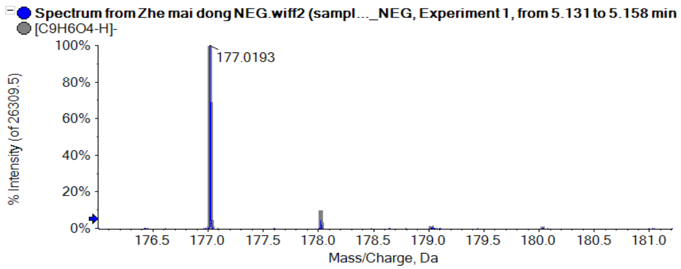

咖啡酸Caffeic acid

Retention Time: 5.21 minutes **Exp RT:** 5.21 minutes
Precursor m/z : 179.0350 **Analyte Name:** 咖啡酸Caffeic acid
Fit (%) 82.9% **RFit (%)** 100.0%

Collision Energy = 35 ± 15 eV

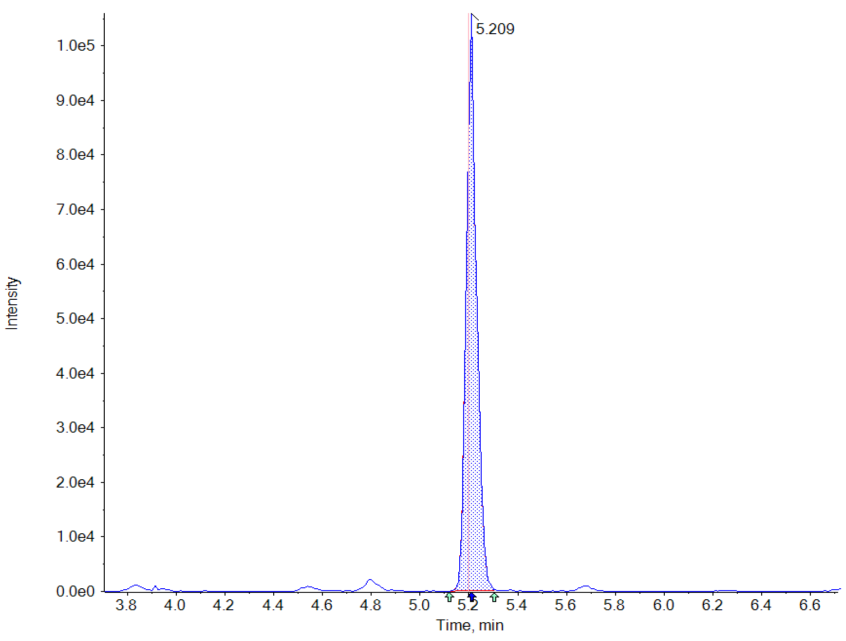

Acquired / Library MSMS

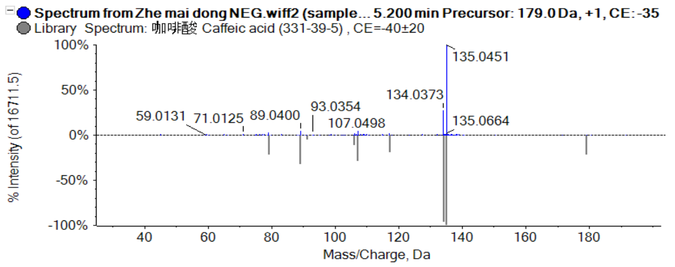

Acquired / Theoretical MS

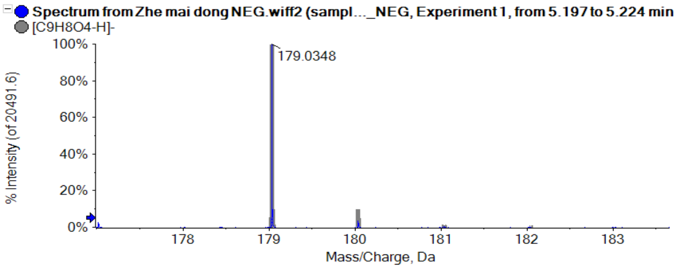

刺五加苷E Eleutheroside E

Retention Time: 5.88 minutes **Exp RT:** 5.88 minutes

Precursor m/z : 787.2666 **Analyte Name:**

Fit (%) 99.5% **RFit (%)** 98.0% 刺五加苷E Eleutheroside E +HCOOH

Collision Energy = 35 ± 15 eV

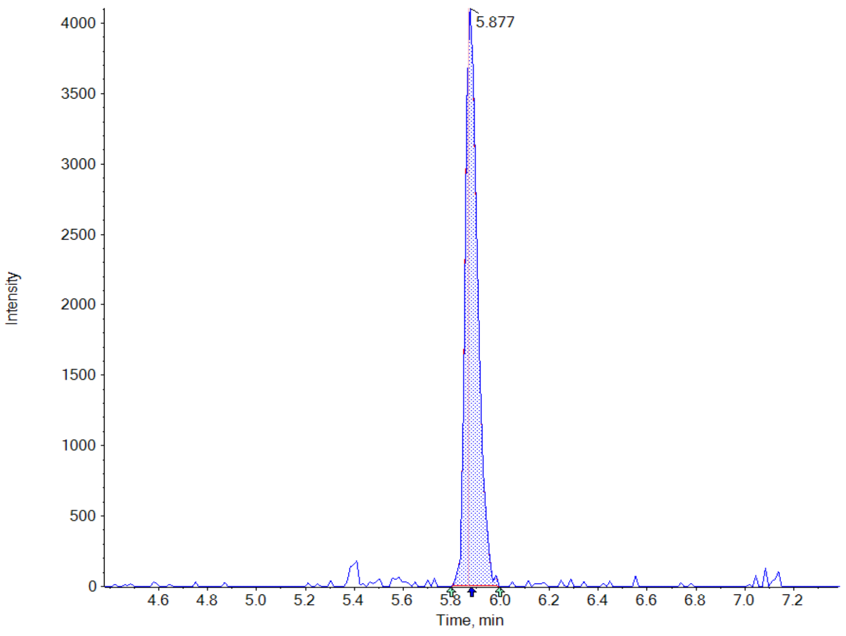

Acquired / Library MSMS

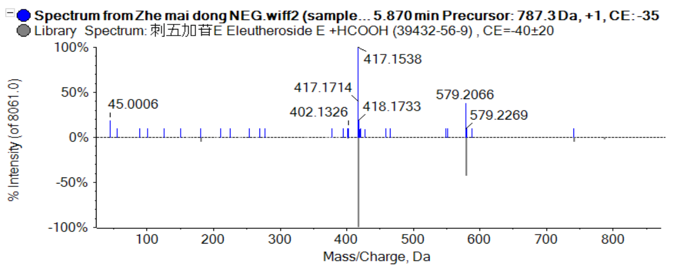

Acquired / Theoretical MS

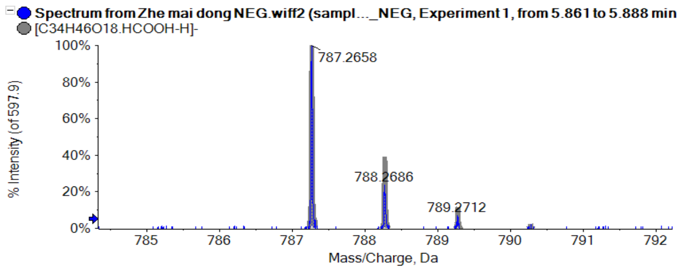

芦丁Rutin

Retention Time: 6.30 minutes
Precursor m/z : 611.1607
Fit (%) 99.6% **RFit (%)** 100.0%

Exp RT: 6.31 minutes
Analyte Name:
芦丁Rutin

Collision Energy = 35 ± 15 eV

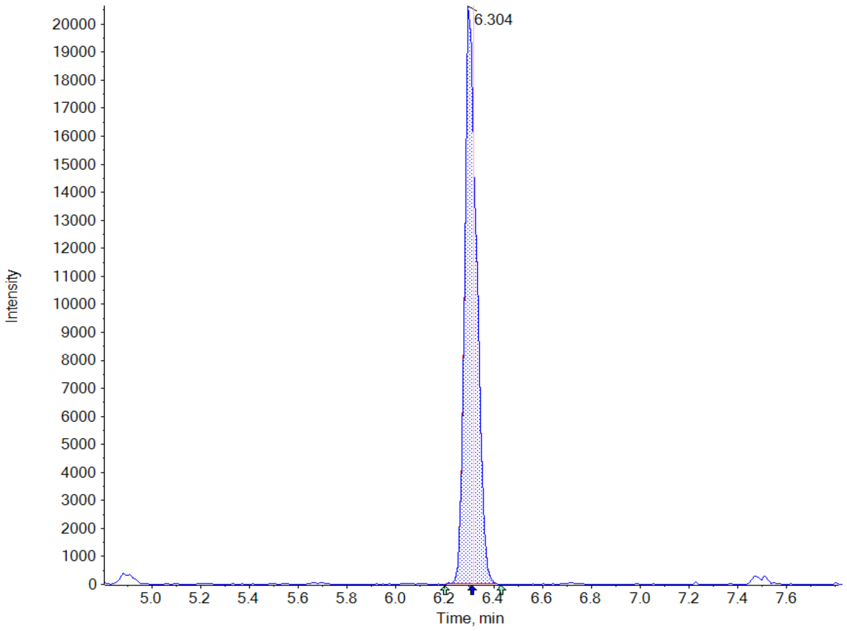

Acquired / Library MSMS

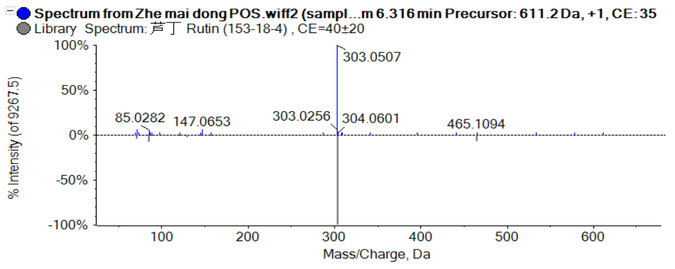

Acquired / Theoretical MS

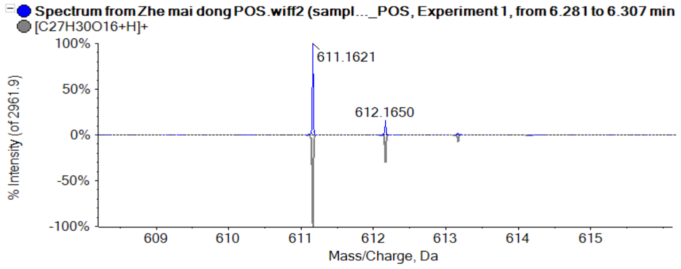

金丝桃苷Hyperin

Retention Time: 6.51 minutes
Precursor m/z : 463.0882
Fit (%) 98.4% **RFit (%)** 100.0%

Exp RT: 6.50 minutes
Analyte Name: 金丝桃苷Hyperin

Collision Energy = 35 ± 15 eV

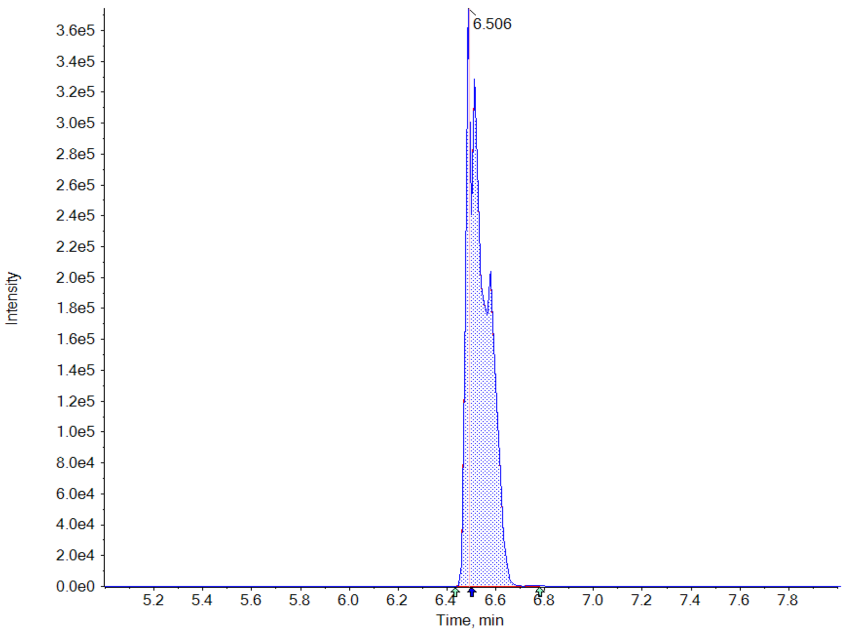

Acquired / Library MSMS

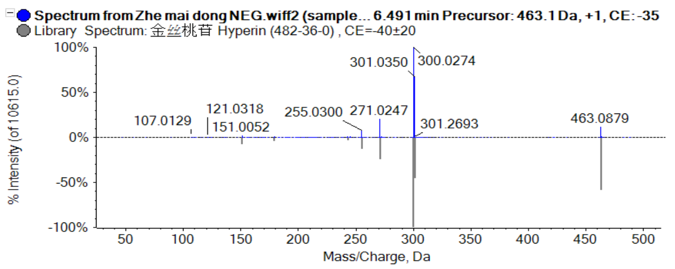

Acquired / Theoretical MS

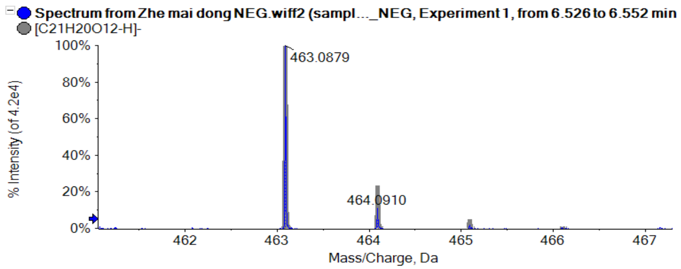

紫云英苷 Astragalin

Retention Time: 6.96 minutes
Precursor m/z : 447.0933
Fit (%) 98.8% **RFit (%)** 100.0%

Exp RT: 6.90 minutes
Analyte Name:
紫云英苷 Astragalin

Collision Energy = 35 ± 15 eV

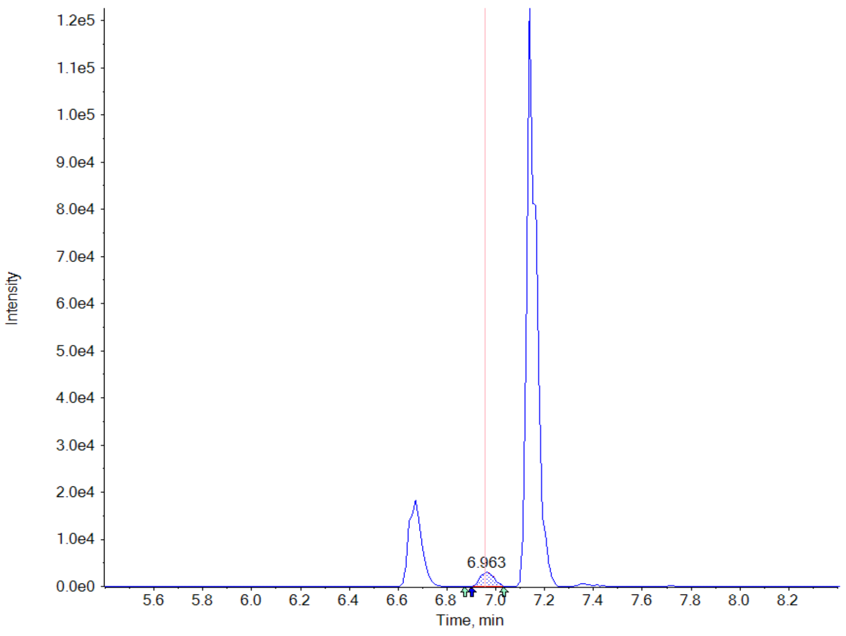

Acquired / Library MSMS

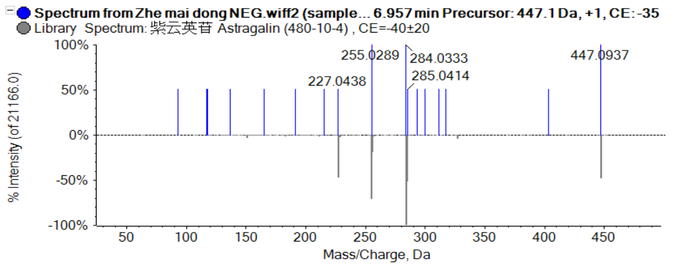

Acquired / Theoretical MS

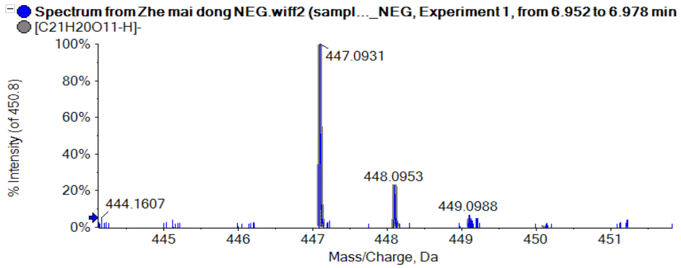

Created with SCIEX OS 1.3.1

Printed:1/11/2021 4:55:13 PM

特女贞苷Specnuezhenide

Retention Time: 7.04 minutes
Precursor m/z : 685.2349
Fit (%) 99.8% **RFit (%)** 99.2%

Exp RT: 7.04 minutes
Analyte Name:
特女贞苷Specnuezhenide

Collision Energy = 35 ± 15 eV

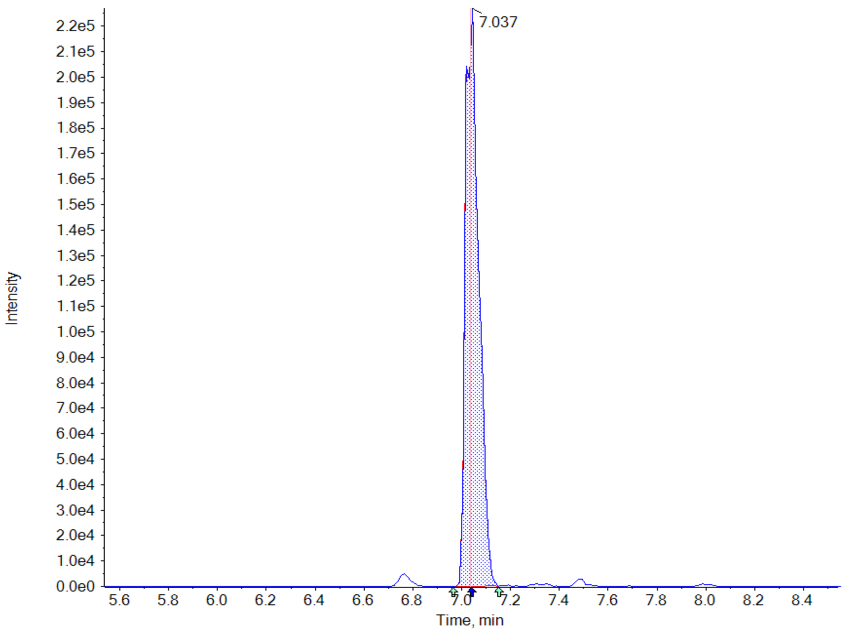

Acquired / Library MSMS

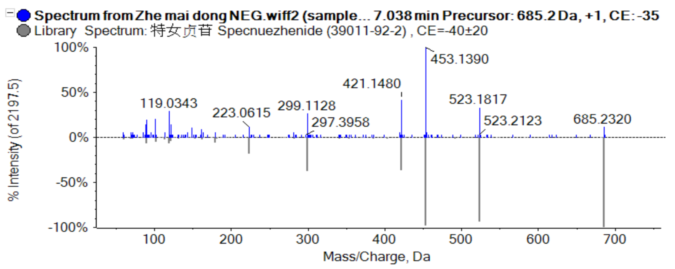

Acquired / Theoretical MS

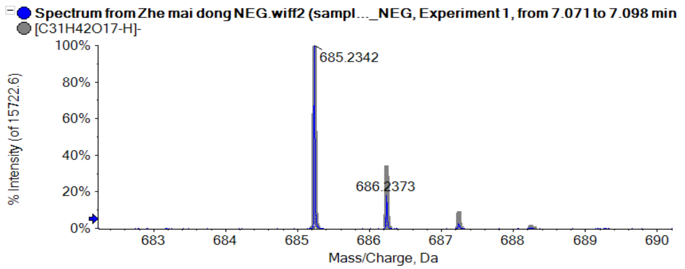

芸香柚皮苷Narirutin

Retention Time: 7.08 minutes
Precursor m/z : 579.1719
Fit (%) 97.2% **RFit (%)** 99.5%

Exp RT: 7.08 minutes
Analyte Name:
芸香柚皮苷Narirutin

Collision Energy = 35 ± 15 eV

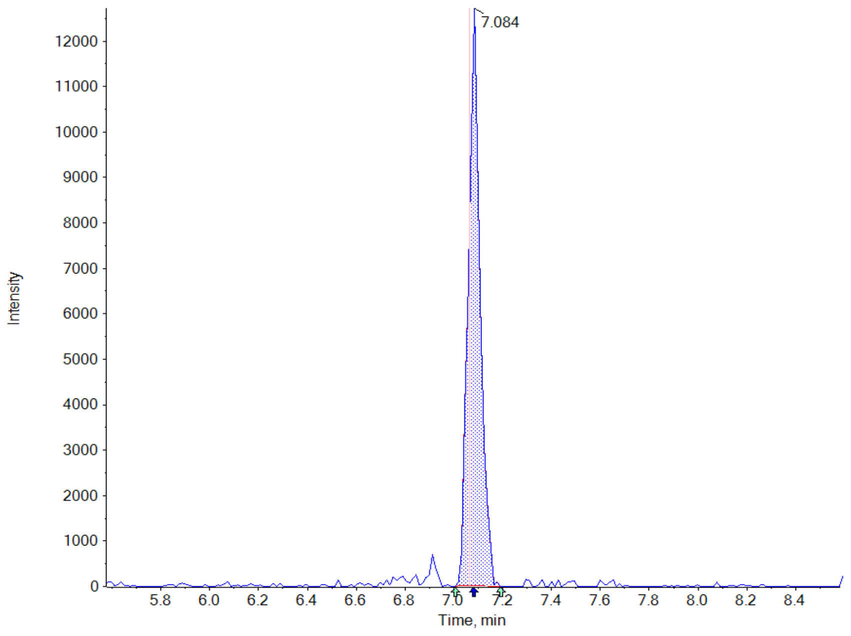

Acquired / Library MSMS

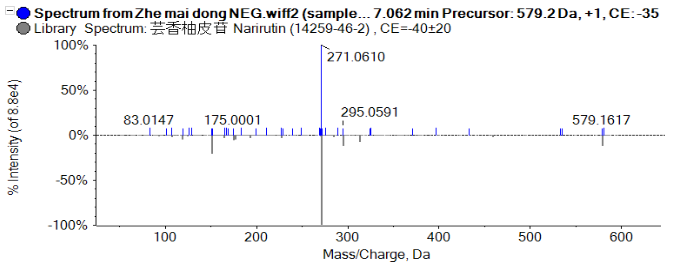

Acquired / Theoretical MS

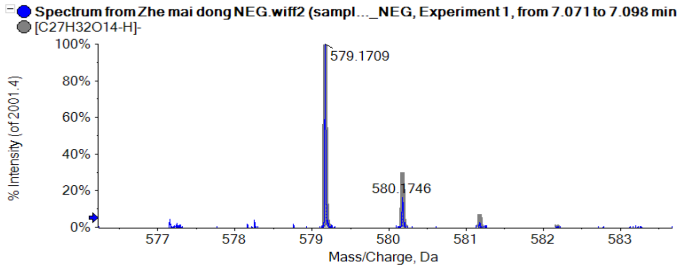

1,5一二咖啡酰奎宁酸1,5-Dicaffeoylquinic acid

**Retention Time:** 7.49 minutes	**Exp RT:** 7.48 minutes
**Precursor m/z :** 515.1195	**Analyte Name:**
**Fit (%)** 94.8% **RFit (%)** 92.6%	1,5一二咖啡酰奎宁酸1,5-Dicaffeoylquinic acid

Collision Energy = 35 ± 15 eV

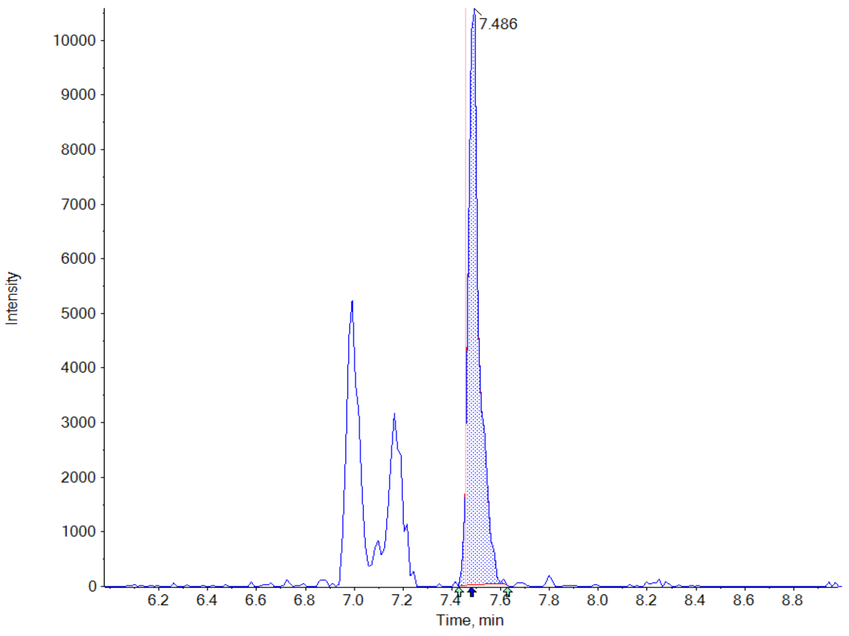

Acquired / Library MSMS

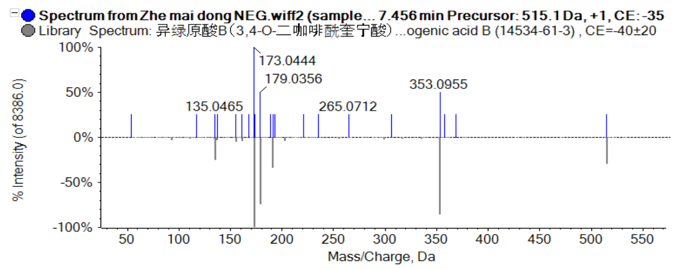

Acquired / Theoretical MS

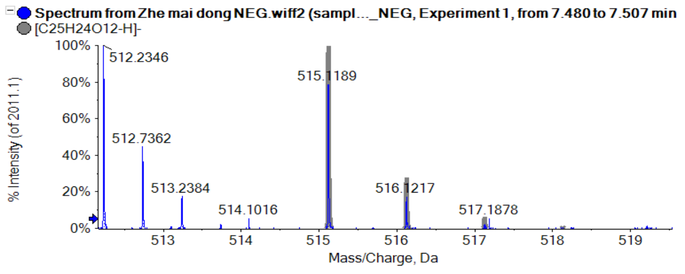

橙皮苷Hesperidin

Retention Time: 7.49 minutes
Precursor m/z : 609.1825
Fit (%) 98.1% **RFit (%)** 100.0%

Exp RT: 7.49 minutes
Analyte Name:
橙皮苷Hesperidin

Collision Energy = 35 ± 15 eV

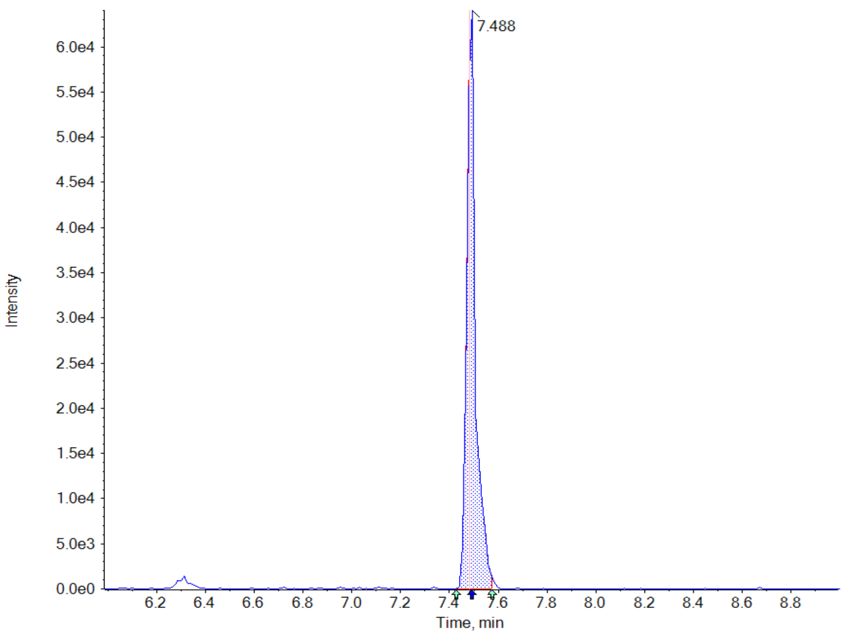

Acquired / Library MSMS

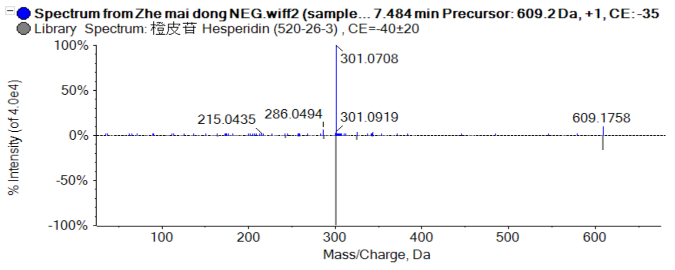

Acquired / Theoretical MS

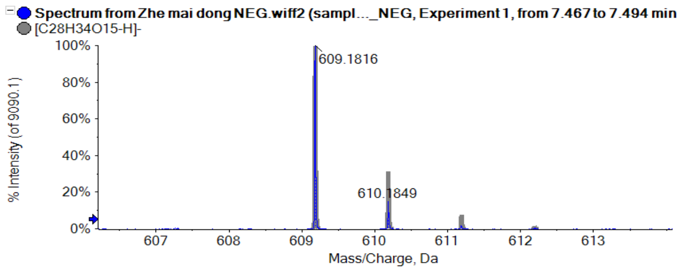

槲皮素Quercetin

Retention Time: 9.10 minutes
Precursor m/z : 301.0354
Fit (%) 95.1% **RFit (%)** 97.9%

Exp RT: 9.09 minutes
Analyte Name:
槲皮素Quercetin

Collision Energy = 35 ± 15 eV

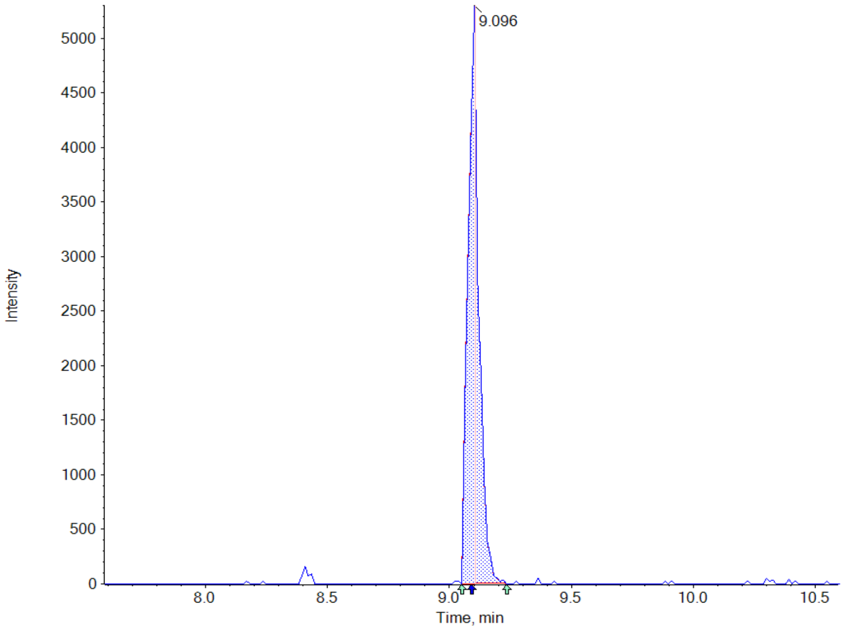

Acquired / Library MSMS

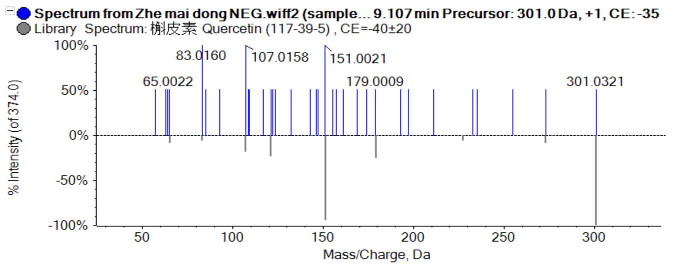

Acquired / Theoretical MS

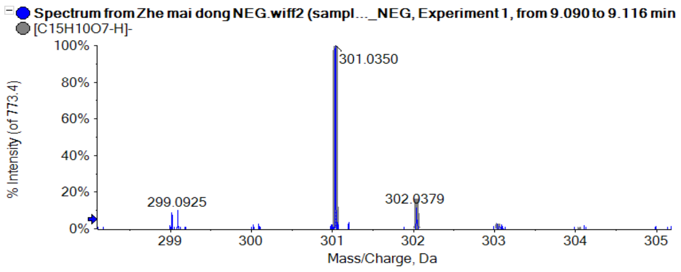

毛蕊异黄酮苷Calycosin-7-o-glucoside

Retention Time: 9.43 minutes
Precursor m/z : 491.1195
Fit (%) 99.7% **RFit (%)** 98.1%

Exp RT: 9.42 minutes
Analyte Name:
毛蕊异黄酮苷Calycosin-7-o-glucoside +HCOOH

Collision Energy = 35 ± 15 eV

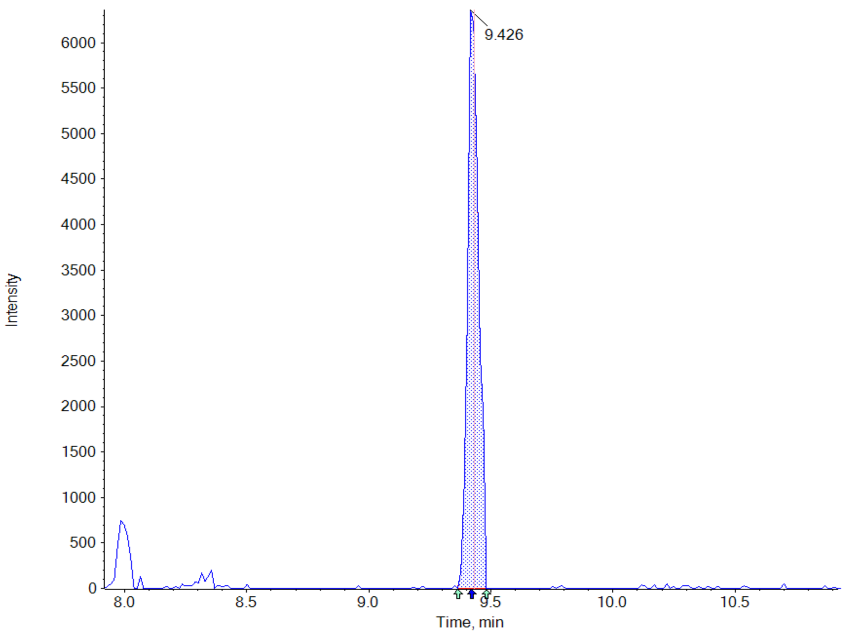

Acquired / Library MSMS

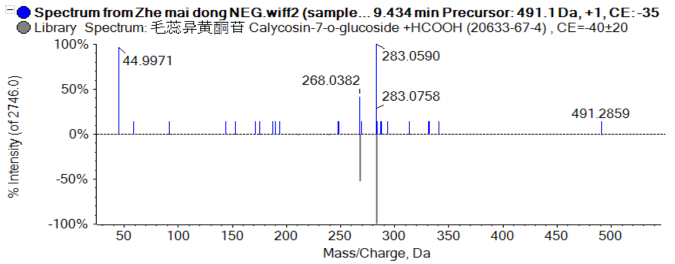

Acquired / Theoretical MS

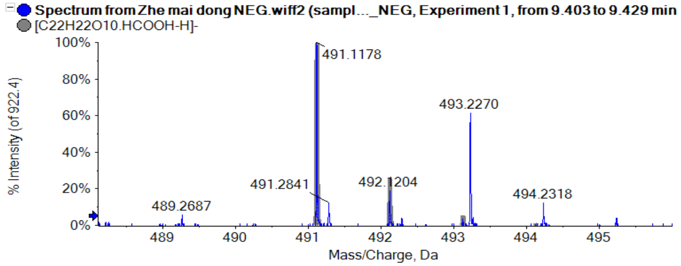

芹菜素Apigenin

**Retention Time:** 10.10 minutes	**Exp RT:** 10.10 minutes
**Precursor m/z :** 269.0455	**Analyte Name:**
**Fit (%)** 94.9% **RFit (%)** 100.0%	芹菜素Apigenin

Collision Energy = 35 ± 15 eV

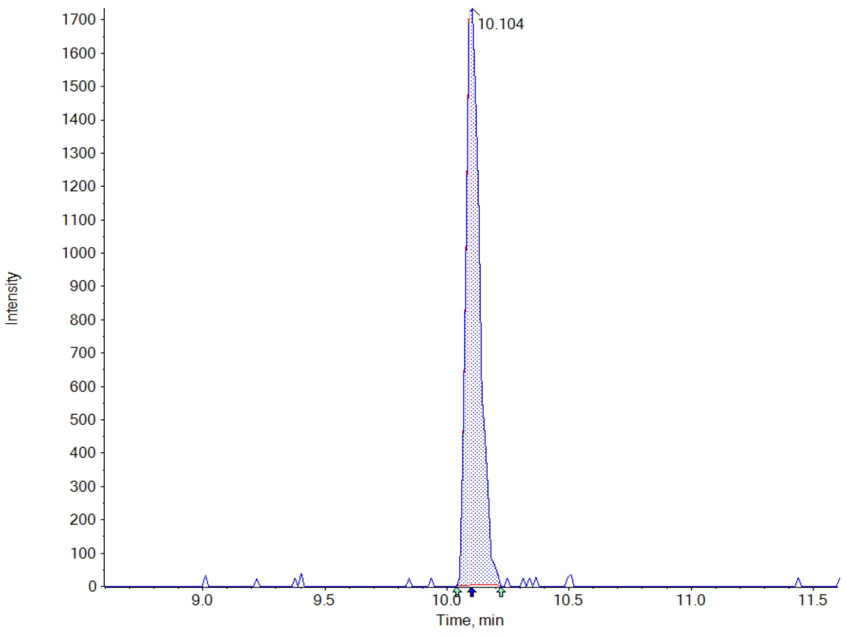

Acquired / Library MSMS

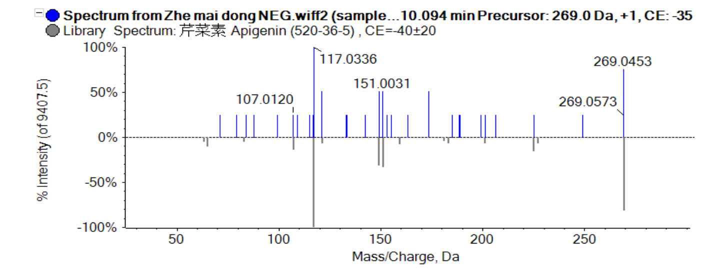

Acquired / Theoretical MS

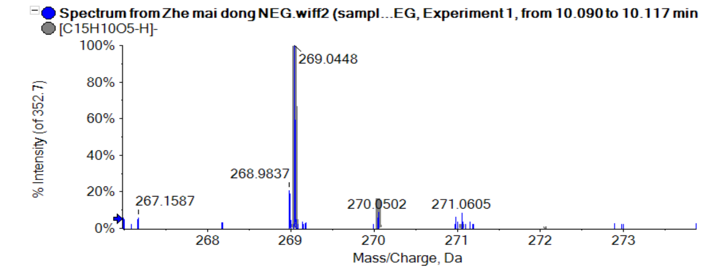

对羟基苯甲酸丁酯Butylparaben

Retention Time: 13.24 minutes **Exp RT:** 12.79 minutes

Precursor m/z : 193.0870 **Analyte Name:**

Fit (%) 95.1% **RFit (%)** 94.7% 对羟基苯甲酸丁酯Butylparaben

Collision Energy = 35 ± 15 eV

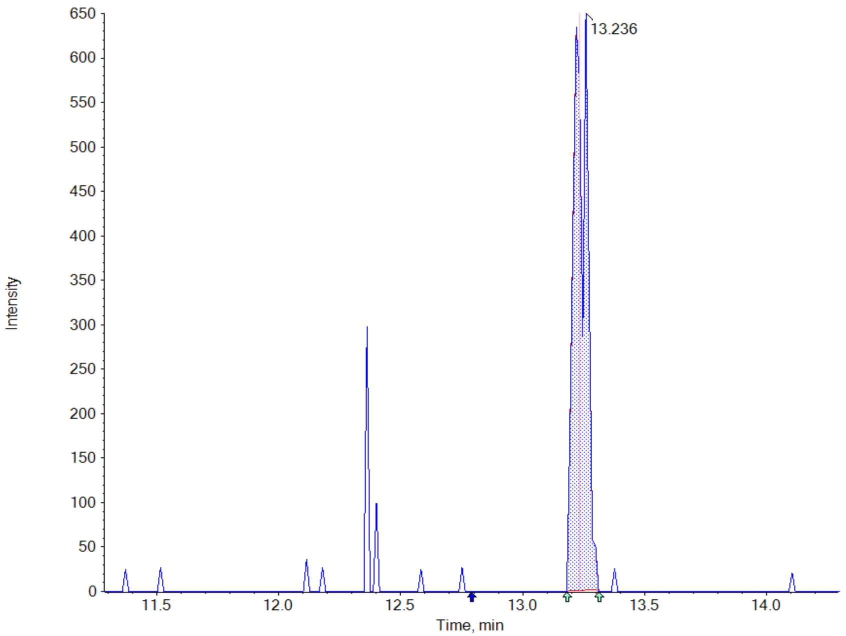

Acquired / Library MSMS

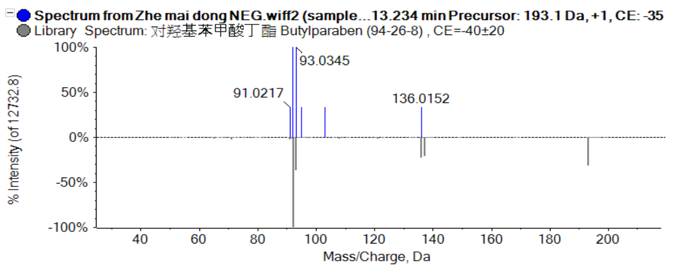

Acquired / Theoretical MS

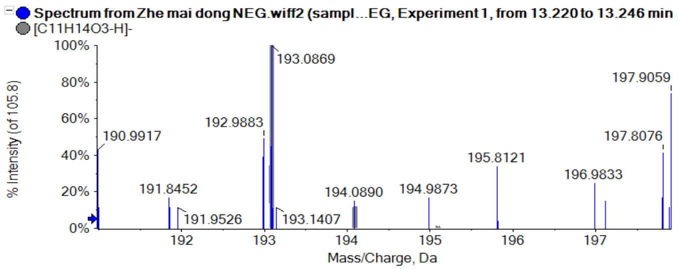

短葶山麦冬皂苷C Liriope muscari baily saponins C

Retention Time: 13.53 minutes
Precursor m/z : 869.4540
Fit (%) 98.9% **RFit (%)** 100.0%

Exp RT: 13.50 minutes
Analyte Name:
短葶山麦冬皂苷C Liriope muscari baily saponins C

Collision Energy = 35 ± 15 eV

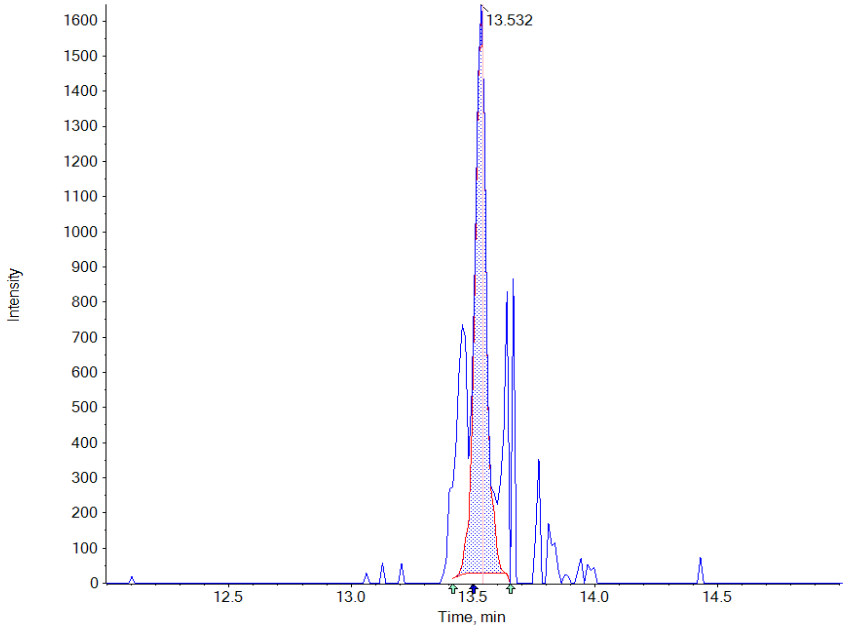

Acquired / Library MSMS

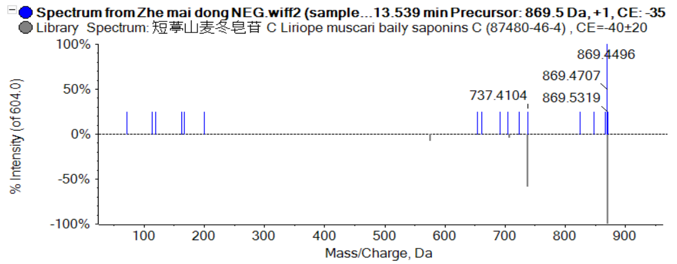

Acquired / Theoretical MS

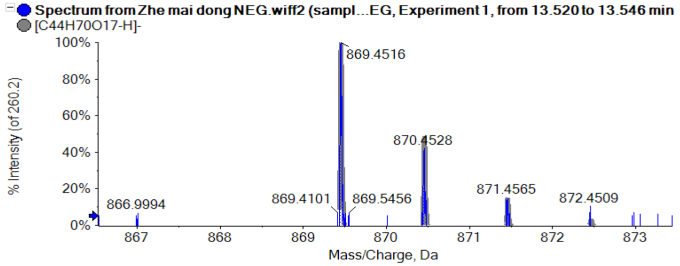

积雪草酸Asiatic acid

Retention Time: 14.06 minutes **Exp RT:** 14.07 minutes

Precursor m/z : 487.3429 **Analyte Name:**

Fit (%) 100.0% **RFit (%)** 91.0% 积雪草酸Asiatic acid

Collision Energy = 35 ± 15 eV

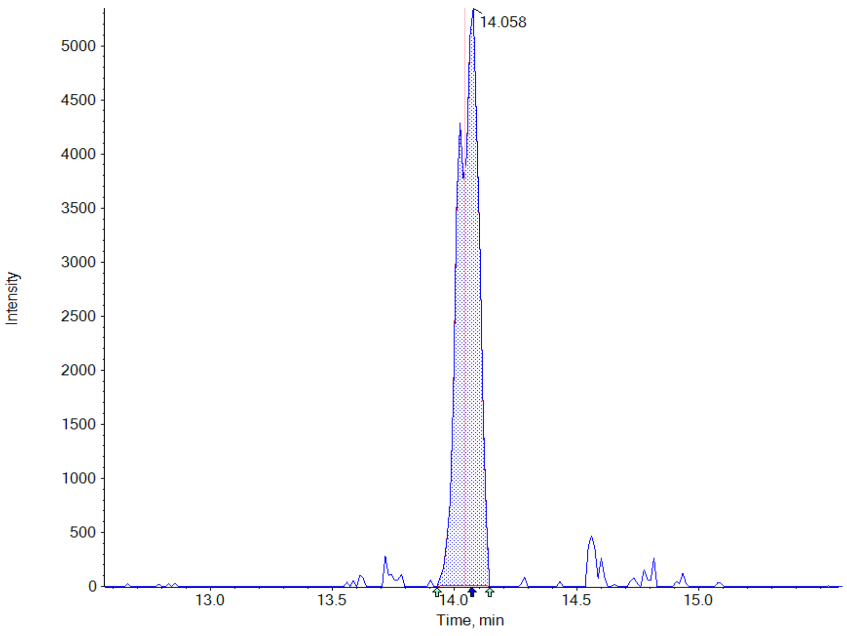

Acquired / Library MSMS

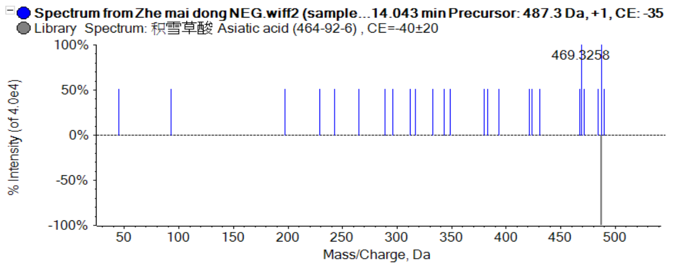

Acquired / Theoretical MS

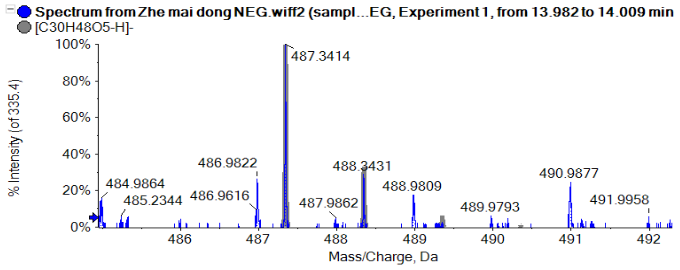

甲基麦冬高异黄酮A Methylophiopogonone A

Retention Time: 14.71 minutes **Exp RT:** 14.71 minutes
Precursor m/z : 339.0874 **Analyte Name:**
Fit (%) 95.9% **RFit (%)** 100.0% 甲基麦冬高异黄酮A Methylophiopogonone A

Collision Energy = 35 ± 15 eV

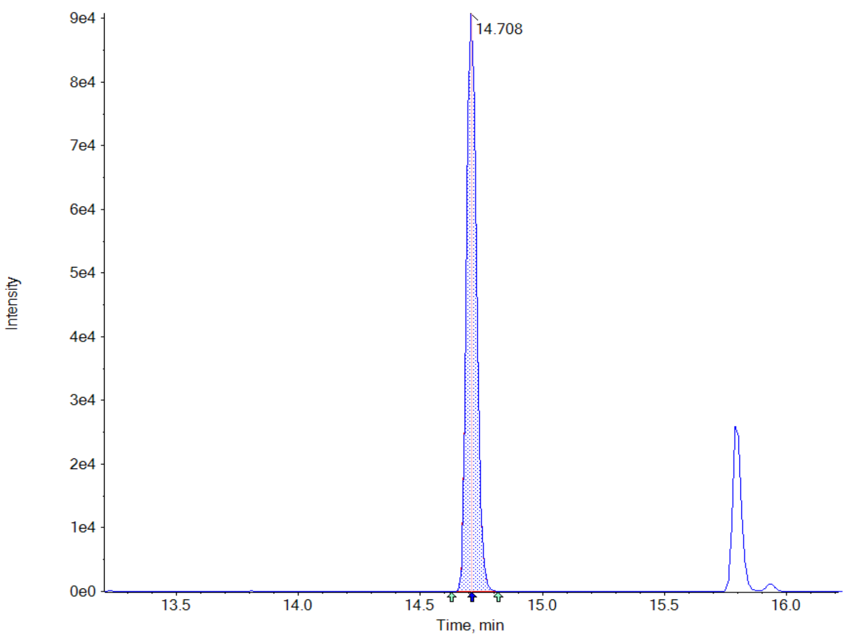

Acquired / Library MSMS

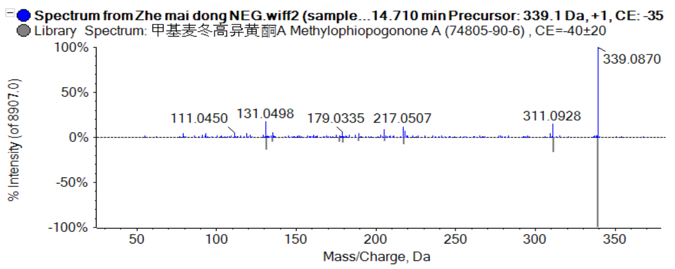

Acquired / Theoretical MS

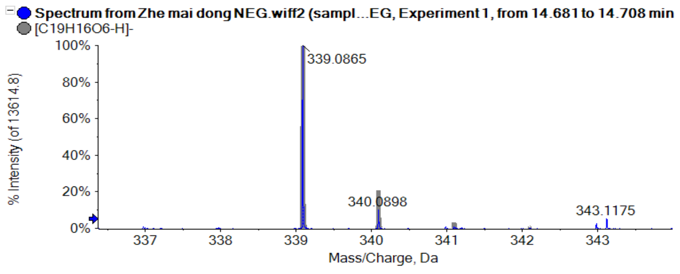

麦冬皂苷D Ophiopogonin D

Retention Time: 14.83 minutes **Exp RT:** 14.83 minutes

Precursor m/z : 899.4646 **Analyte Name:**

Fit (%) 100.0% **RFit (%)** 98.0% 麦冬皂苷D Ophiopogonin D +HCOOH

Collision Energy = 35 ± 15 eV

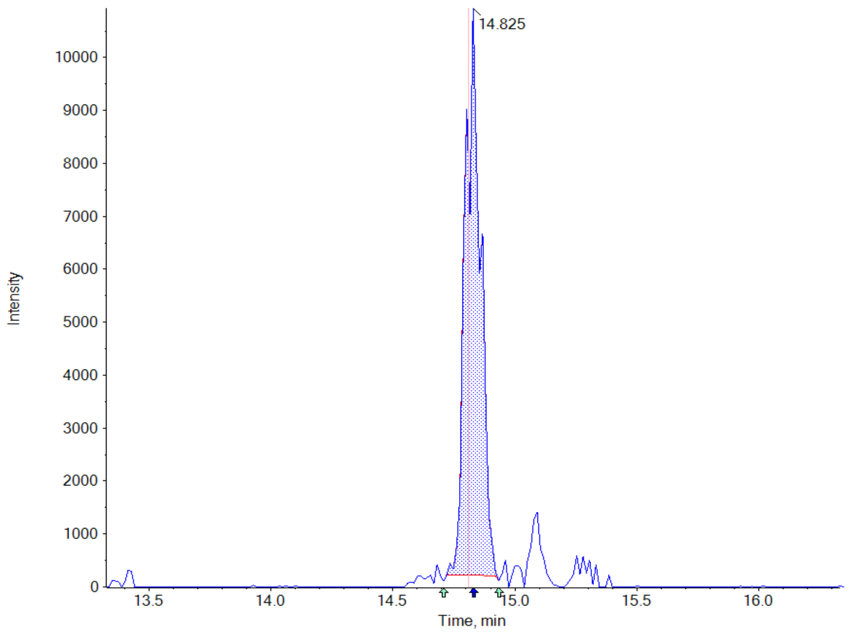

Acquired / Library MSMS

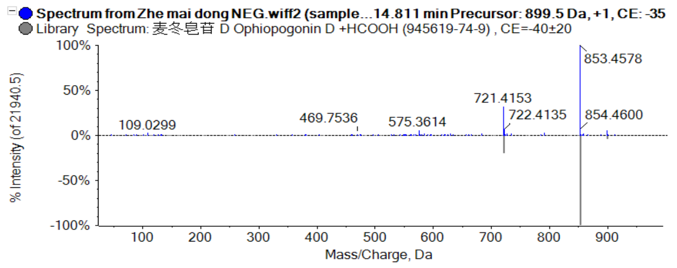

Acquired / Theoretical MS

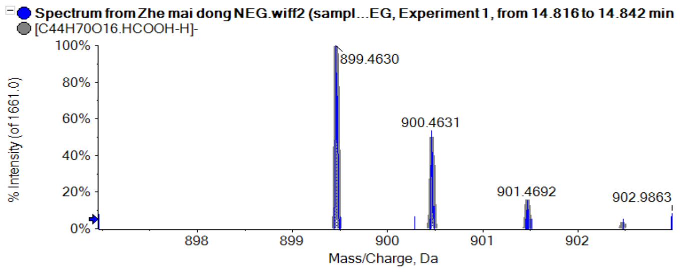

姜糖脂B Gingerglycolipid B

Retention Time: 15.21 minutes **Exp RT:** 15.21 minutes

Precursor m/z : 723.3809 **Analyte Name:**

Fit (%) 98.0% **RFit (%)** 100.0% 姜糖脂B Gingerglycolipid B +HCOOH

Collision Energy = 35 ± 15 eV

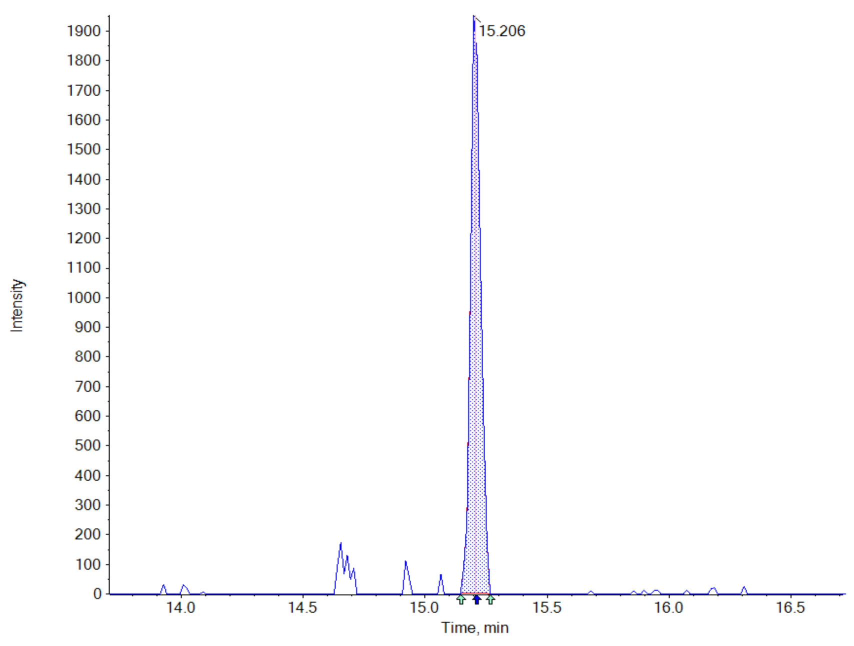

Acquired / Library MSMS

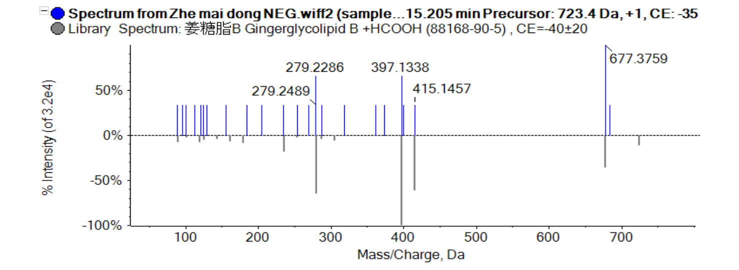

Acquired / Theoretical MS

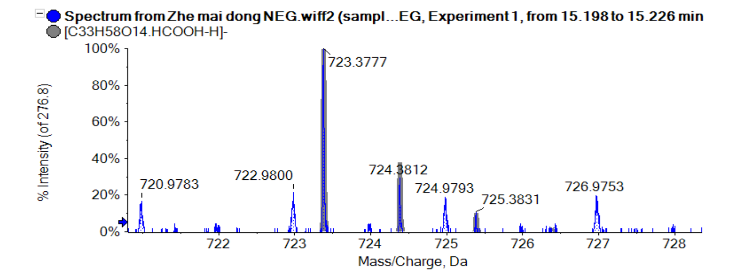

科罗索酸Corosolic acid

**Retention Time:** 15.55 minutes<br>**Precursor m/z :** 471.3480<br>**Fit (%)** 100.0% **RFit (%)** 100.0%	**Exp RT:** 15.55 minutes<br>**Analyte Name:**<br>科罗索酸Corosolic acid

Collision Energy = 35 ± 15 eV

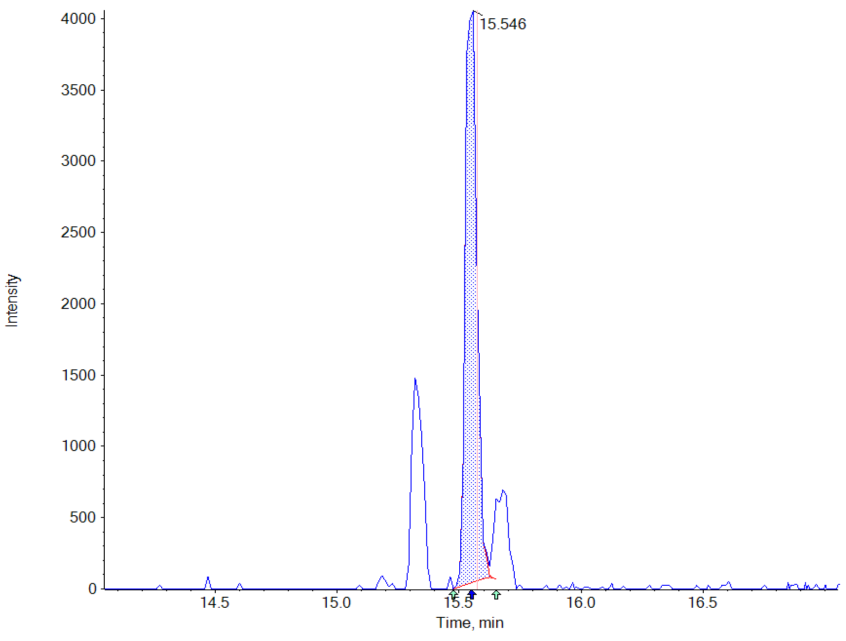

Acquired / Library MSMS

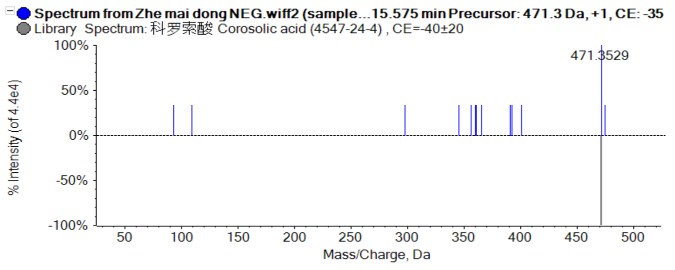

Acquired / Theoretical MS

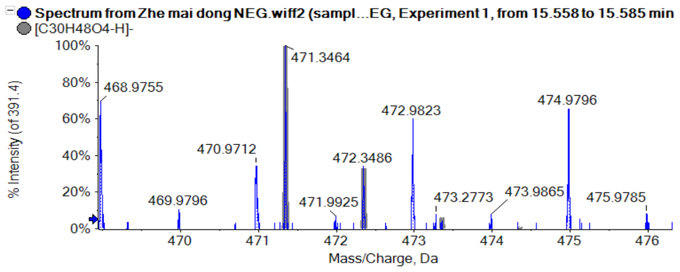

麦冬黄烷酮C Ophiopogonanone C

Retention Time: 15.72 minutes
Precursor m/z : 355.0823
Fit (%) 93.6% **RFit (%)** 98.4%

Exp RT: 15.72 minutes
Analyte Name:
麦冬黄烷酮C Ophiopogonanone C

Collision Energy = 35 ± 15 eV

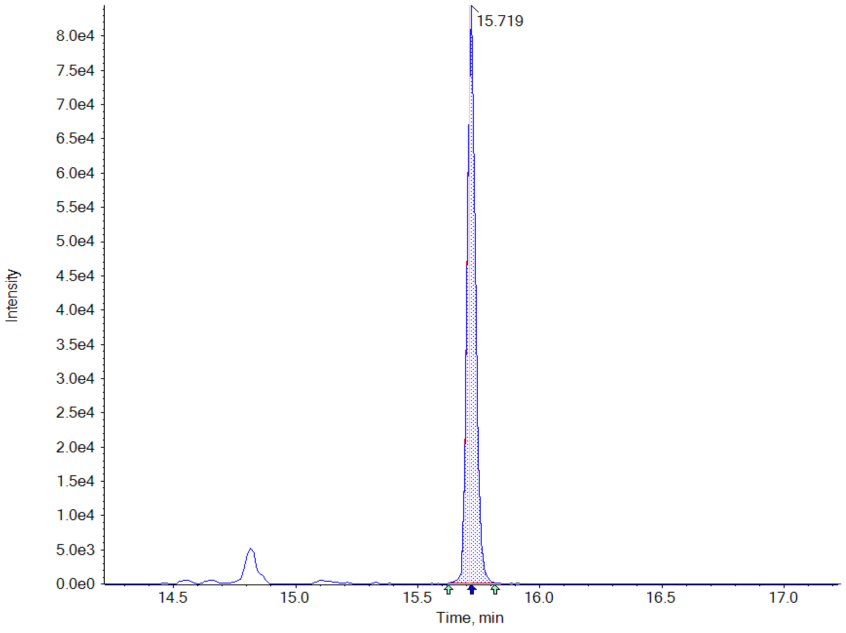

Acquired / Library MSMS

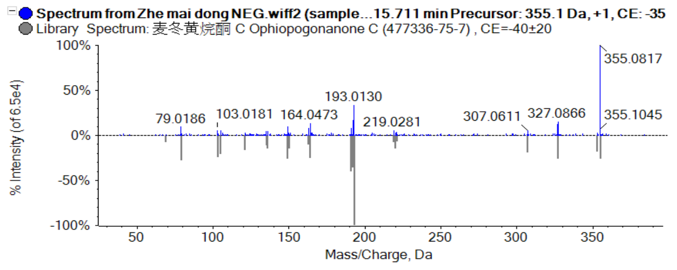

Acquired / Theoretical MS

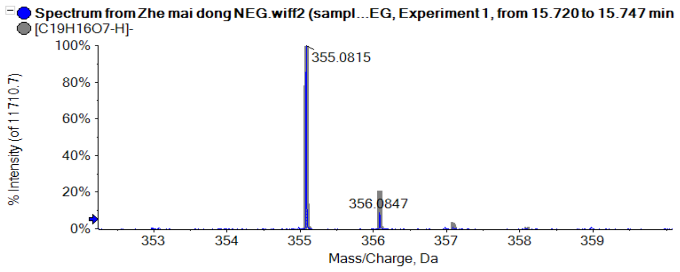

齐墩果酸Oleanolic acid

**Retention Time:** 16.77 minutes<br>**Precursor m/z :** 455.3531<br>**Fit (%)** 98.4% **RFit (%)** 99.3%	**Exp RT:** 16.78 minutes<br>**Analyte Name:**<br>齐墩果酸Oleanolic acid

Collision Energy = 35 ± 15 eV

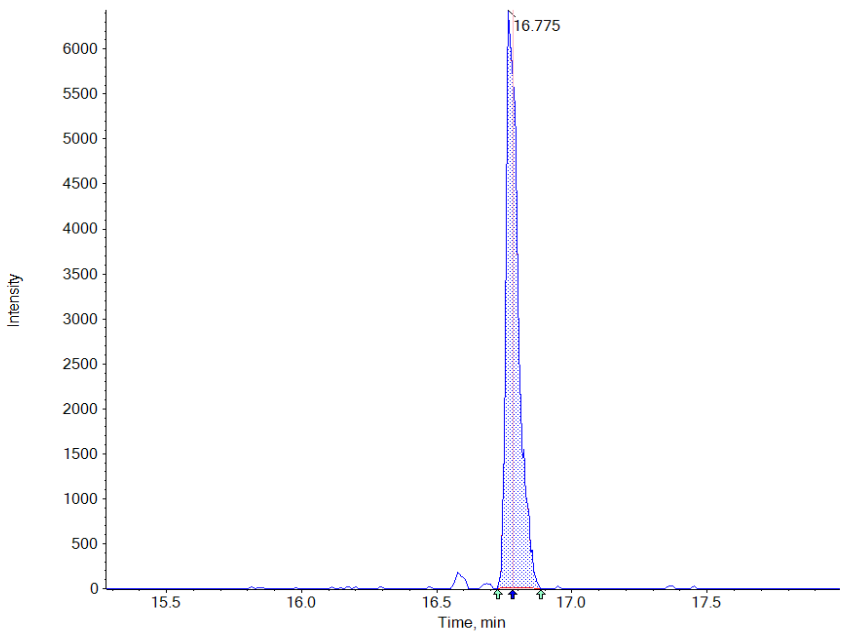

Acquired / Library MSMS

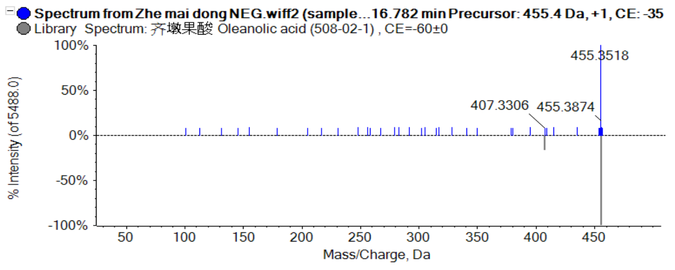

Acquired / Theoretical MS

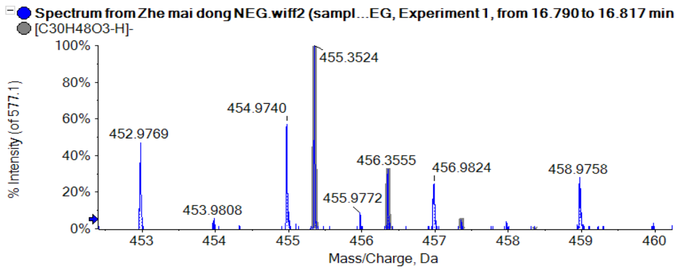

Table S3 The different chemical constituents between CMD and ZMD in GC-MS analysis

NO.	RT(min)	Name	Match	VIP	T-test	Fold change (ZMD/CMD)
1	16.19	D-Fructose	928	1.34	0.031	0.87
2	8.80	glycerol	922	1.92	0.000	1.22
3	11.69	Malic acid	910	1.32	0.034	-1.20
4	26.80	Sucrose	907	1.63	0.004	-1.06
5	9.25	Glycine	894	1.94	0.000	-9.42
6	9.13	L-Proline	888	1.76	0.001	-8.10
7	8.67	Monoethanolamine	882	1.82	0.001	-7.41
8	8.82	Phosphoric acid	873	1.43	0.019	0.51
9	18.86	Inositol	864	1.80	0.001	-2.41
10	16.40	d-Glucose	857	1.46	0.016	0.86
11	16.34	d-Galactose	852	1.31	0.037	0.54
12	10.01	Serine	827	1.35	0.029	-7.38
13	12.35	Threonate	814	1.50	0.012	-2.79
14	12.21	4-Aminobutanoic acid	798	1.68	0.003	-10.95
15	16.28	D-Mannoheptulose	755	1.57	0.007	-0.57
16	20.03	2-(beta-D-Glucosyl)-sn-glycerol	736	1.57	0.007	-2.88
17	26.78	Trehalose	714	1.34	0.031	-0.69

Table S4 The significantly different chemical constituents between CMD and ZMD (ESI+)

NO.	RT (min)	Name	molecular weight	VIP	T-test	Fold change (ZMD/CMD)
1	12.90	Thymidine	242.0903	1.44	0.002	-1.32
2	11.13	Chlorogenic Acid	354.0951	1.20	0.019	5.38
3	8.35	Indole-3-carboxylic acid	161.0477	1.45	0.002	2.39
4	6.79	Pyridoxal Phosphate	247.0246	1.24	0.014	1.18
5	9.74	LysoPC(18:2(9Z,12Z))	519.3325	1.21	0.018	6.69
6	8.90	Traumatic Acid	228.1362	1.26	0.012	2.75
7	8.90	LPA(0:0/18:0)	438.2746	1.22	0.017	1.07
8	13.75	LysoPC(22:2(13Z,16Z))	575.3951	1.54	0.001	2.60
9	1.91	L-Cysteine	121.0197	1.59	0.000	-3.09
10	0.96	alpha-Tocopherol	430.3811	1.69	0.000	-2.96
11	9.85	LysoPE (0:0/18:3(6Z,9Z,12Z))	475.2699	1.05	0.048	-0.88
12	1.23	5-Hydroxyisourate	184.0233	1.47	0.001	-1.82
13	15.00	MG(0:0/22:1(13Z)/0:0)	412.3553	1.31	0.008	-0.73
14	12.69	Dihydroquercetin	304.0583	1.18	0.022	-0.71
15	8.34	Cytidine	243.0855	1.41	0.003	2.80
16	12.16	Glutinosone	220.1463	1.20	0.019	-0.93
17	12.68	Glutathione	307.0838	1.54	0.000	-1.52
18	9.53	L-Cystathionine	222.0674	1.29	0.009	-1.78
19	18.14	L-Arginine	174.1117	1.14	0.028	-0.36
20	8.35	Cadaverine	102.1157	1.30	0.009	3.72
21	11.13	Pyridoxine 5-phosphate	249.0402	1.42	0.003	3.27
22	5.19	Stearoylethanolamide	327.3137	1.34	0.006	-1.60
23	1.19	6-Gingerol	294.1831	1.08	0.041	-1.56
24	15.09	Gibberellin A53	348.1937	1.18	0.022	-1.57
25	7.58	Calystegin A3	159.0895	1.25	0.014	-2.21

Table S5 The significantly different chemical constituents between CMD and ZMD (ESI-)

NO.	RT (min)	Name	molecular weight	VIP	T-test	Fold change (ZMD/CMD)
1	1.17	Geranyl diphosphate	314.0684	1.09	0.049	-1.34
2	1.00	Gibberellin A53	348.1937	1.23	0.021	-1.28
3	8.68	Gibberellin A86	380.1471	1.63	0.000	-5.04
4	4.28	Glutinosone	220.1463	1.23	0.023	2.26
5	18.20	Isovaleric acid	102.0681	1.21	0.024	-0.58
6	7.11	Kynurenic acid	189.0426	1.78	0.000	-2.90
7	6.05	LysoPC(18:4(6Z,9Z,12Z,15Z))	515.3012	1.42	0.005	-3.41
8	6.04	LysoPE(0:0/16:1(9Z))	451.2699	1.32	0.011	-3.56
9	11.60	LysoPE(0:0/22:0)	537.3794	1.09	0.049	1.80
10	12.61	LysoPE(0:0/22:1(13Z))	535.3638	1.37	0.008	1.80
11	14.05	PC(14:0/20:0)	761.5935	1.37	0.008	-0.75
12	10.04	PC(18:0/P-18:0)	773.6298	1.17	0.032	1.90
13	9.43	PC(20:0/24:0)	901.7500	1.51	0.002	3.41
14	14.15	Pelargonidin 3-O-(6-O-malonyl-β-D-glucoside)	518.1060	1.32	0.012	1.21
15	4.77	Pyruvate	88.0160	1.72	0.000	-1.46
16	10.81	Quercetin	302.0427	1.13	0.040	-0.99
17	4.77	Taurine	125.0147	1.78	0.000	-1.51
